# Enantioselective Rh/Cu-Catalyzed Addition of Terminal Alkynes to Allenes for the Synthesis of Chiral 1,4-Enynes

**DOI:** 10.1021/acs.orglett.6c01096

**Published:** 2026-04-07

**Authors:** Martin Daiger, Janika Kromer, Samuel Kaplan, Bernhard Breit

**Affiliations:** Institut für Organische Chemie, 9174 Albert-Ludwigs-Universität Freiburg, Albertstraße 21, 79104 Freiburg im Breisgau, Germany

## Abstract

A regio- and enantioselective rhodium/copper-catalyzed coupling of readily available terminal alkynes and allenes is reported. This method enables direct, atom-economic access to chiral 1,4-enynes, a versatile class of compounds widely employed in the synthesis of natural products and pharmaceuticals. The transformation proceeds in high yields with excellent enantioselectivities and displays broad functional-group tolerance. The synthetic utility of the resulting products is further demonstrated through a range of downstream functionalizations that exploit the orthogonal reactivity of the alkyne and alkene moieties.

## Graphical abstract

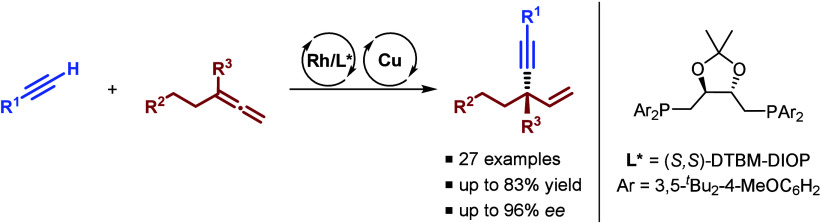

Chiral 1,4-enynes are valuable building blocks in organic synthesis, as they combine two synthetically versatile functional groups in close proximity to a stereogenic center, enabling further derivatization or carbon-skeleton elaboration.^1^ In recent years, several asymmetric allylic alkynylation strategies toward 1,4-enynes have been developed (Scheme 1). In 2011, Hoveyda reported a Cu-catalyzed protocol employing alkynylaluminum reagents.^2^ Subsequently, Carreira^3^ and Breit^4^ described Ir- and Rh-catalyzed approaches based on alkynylboron reagents and alkynyl carboxylic acids, respectively. In contrast, the asymmetric allylation of terminal alkynes remains significantly more challenging, and only a limited number of examples have been reported. In 2014, Sawamura disclosed a Cu-catalyzed asymmetric addition of terminal alkynes to (*Z*)-allylic phosphates.^5^ More recently, Li and co-workers developed a highly regio- and enantioselective synthesis of 1,4-enynes from terminal alkynes and allylic carbonates via Sonogashira-type synergistic Rh/Cu-catalysis.^6^ Despite these advances, existing methods often suffer from drawbacks such as multistep substrate preparation, premetalation of alkynes, the requirement for strongly basic or acidic conditions, or the formation of stoichiometric byproducts.

**Scheme 1:**
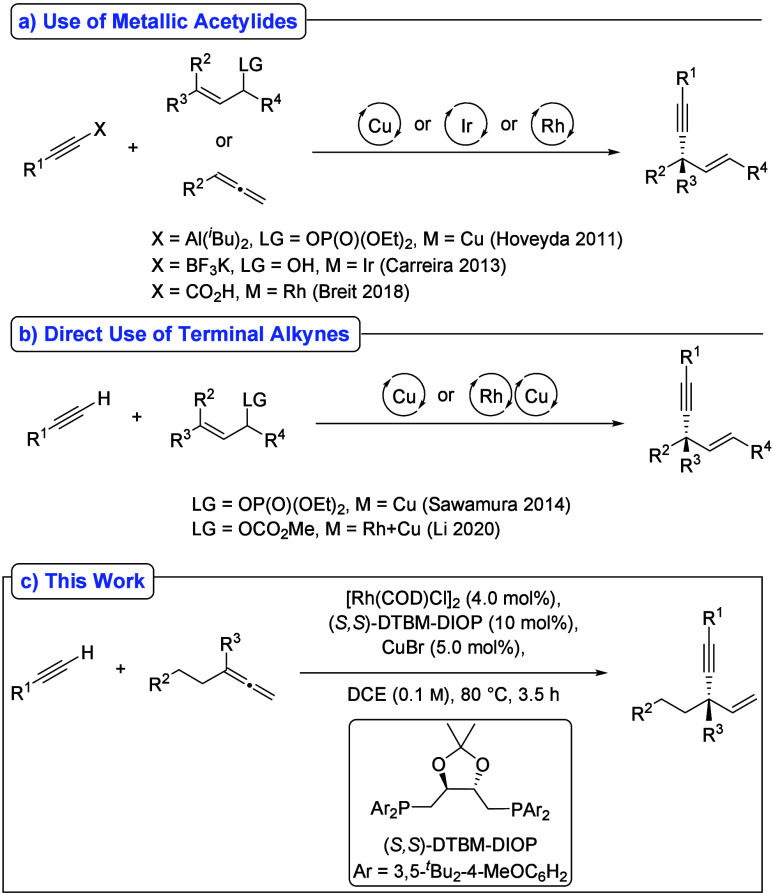
Previous Work on the Asymmetric Allylic Alkynylation and This Work

Over the past several years, our research has focused on the development of Rh-catalyzed chemo-, regio-, and enantioselective allylic additions of carbon,^7^ nitrogen,^8^ oxygen^9^ and sulfur^10^ pronucleophiles to allenes and alkynes, providing atom-efficient alternatives to classical asymmetric allylic substitution reactions.

Building on these studies and inspired by Li’s synergistic Rh/Cu-catalysis, we envisioned that an appropriately tailored chiral catalytic system could enable an atom-economic direct coupling of terminal alkynes and allenes to access chiral 1,4-enynes. Herein, we report a highly branched-selective and enantioselective Rh/Cu-catalyzed allylic alkylation of terminal alkynes with allenes that proceeds with excellent efficiency and selectivity.

The asymmetric allylation of terminal alkynes was investigated using 1-methoxy-4-((penta-3,4-dien-1-yloxy)­methyl)­benzene (**1a**) and ethynylcyclohexane (**2a**) as model substrates (Table 1).

**Table 1:**
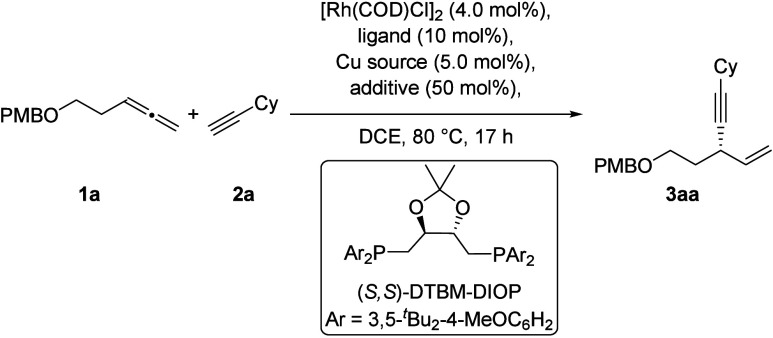
Optimization of Reaction Conditions^a^

Entry	Ligand	Cu Source	Additive	Yield [%]	*ee* [%]
1	dppf	CuI	PPTS	11	nd
2	dppe	CuI	PPTS	nd	–
3	*rac*-BINAP	CuI	PPTS	nd	–
4	(*R,R*)-DIOP	CuI	PPTS	24	87
5	(*S,S*)-DTBM-DIOP	CuI	PPTS	46	89
6	(*S,S*)-DTBM-DIOP	CuBr	PPTS	43	94
7	(*S,S*)-DTBM-DIOP	CuBr	TFA^b^	58	97
8^c^	(*S,S*)-DTBM-DIOP	CuBr	TFA^b^	72	96
9^c^	(*S,S*)-DTBM-DIOP	CuBr	–	78	96
10^c^ ^,^ ^d^	(*S,S*)-DTBM-DIOP	CuBr	–	74^e^	96
11^c^ ^,^ ^d^ ^,^ ^f^	–	CuBr	–	nd	–
12^c^ ^,^ ^d^ ^,^ ^f^	(*S,S*)-DTBM-DIOP	–	–	nd	–
13^c^ ^,^ ^d^ ^,^ ^f^ ^,^ ^g^	(*S,S*)-DTBM-DIOP	CuBr	–	nd	–

a Reaction conditions (unless otherwise specified): **1a** (0.2 mmol), **2a** (0.3 mmol), [Rh­(COD)­Cl_2_ (4.0 mol %), ligand (10 mol %), Cu source (5.0 mol %), additive (50 mol %), in 1,2-dichloroethane (DCE) (1.0 mL), 80 °C, 17 h. The NMR yield of the product was determined by ^1^H NMR spectroscopy using dibromomethane as internal standard. The *ee* values were determined using chiral HPLC. nd = not determined.

b 10 mol % TFA.

c 2.0 mL DCE.

d 3.5 h.

e Isolated yield.

f Ethynylbenzene instead of ethynylcyclohexane.

g No Rh source.

Initial experiments employing 4.0 mol % [Rh­(COD)­Cl_2_, 5.0 mol % CuI, 10 mol % dppf, and 50 mol % PPTS afforded the desired product **3aa** in low yield (11%, entry 1). Ligand screening (entries 1–4, see also Supporting Information) revealed that DIOP-type ligands were most effective. In particular, the sterically demanding (*S,S*)-DTBM-DIOP significantly improved the reaction outcome, delivering **3aa** in 46% yield and 89% *ee* (entry 5). Further enhancement of enantioselectivity was achieved by replacing CuI with CuBr (entry 6). Decreasing the reaction concentration to 0.1 m and omitting the Brønsted-acid additive led to a substantial increase in yield (78%, entry 9). In situ FT-IR monitoring (see Supporting Information) indicated complete consumption of both starting materials within 3 h, allowing the reaction time to be shortened from 17 to 3.5 h without loss of efficiency (entry 10). Control experiments confirmed that the chiral ligand, as well as both Rh- and Cu-catalysts, are essential for productive coupling (entries 11–13).

With the optimized reaction conditions in hand, we next explored the substrate scope of the Rh-catalyzed addition of terminal alkynes to allenes using 1-methoxy-4-((penta-3,4-dien-1-yloxy)­methyl)­benzene (**1a**) as the allene component (Scheme 2, top). The scope evaluation commenced with aryl-substituted terminal alkynes. Phenylacetylene delivered the corresponding 1,4-enyne **3ab** in very good yield and excellent enantioselectivity, and a broad range of substituted aryl alkynes proved to be equally well tolerated under the optimized conditions. Both electron-withdrawing (e.g., fluoro) and electron-donating substituents (e.g., methoxy or amino groups) on aryl alkynes were well tolerated, affording the corresponding chiral 1,4-enynes **3ac**-**3ah** in good yields and uniformly excellent enantioselectivities. Notably, unprotected anilines in the *ortho-* and *meta-*positions reacted chemoselectively at the terminal alkyne carbon. An alkyne with a heteroaromatic substituent, 2-ethynylthiophene, also proved to be a competent substrate, delivering product **3ai** in 63% yield and 94% *ee*.

**Scheme 2:**
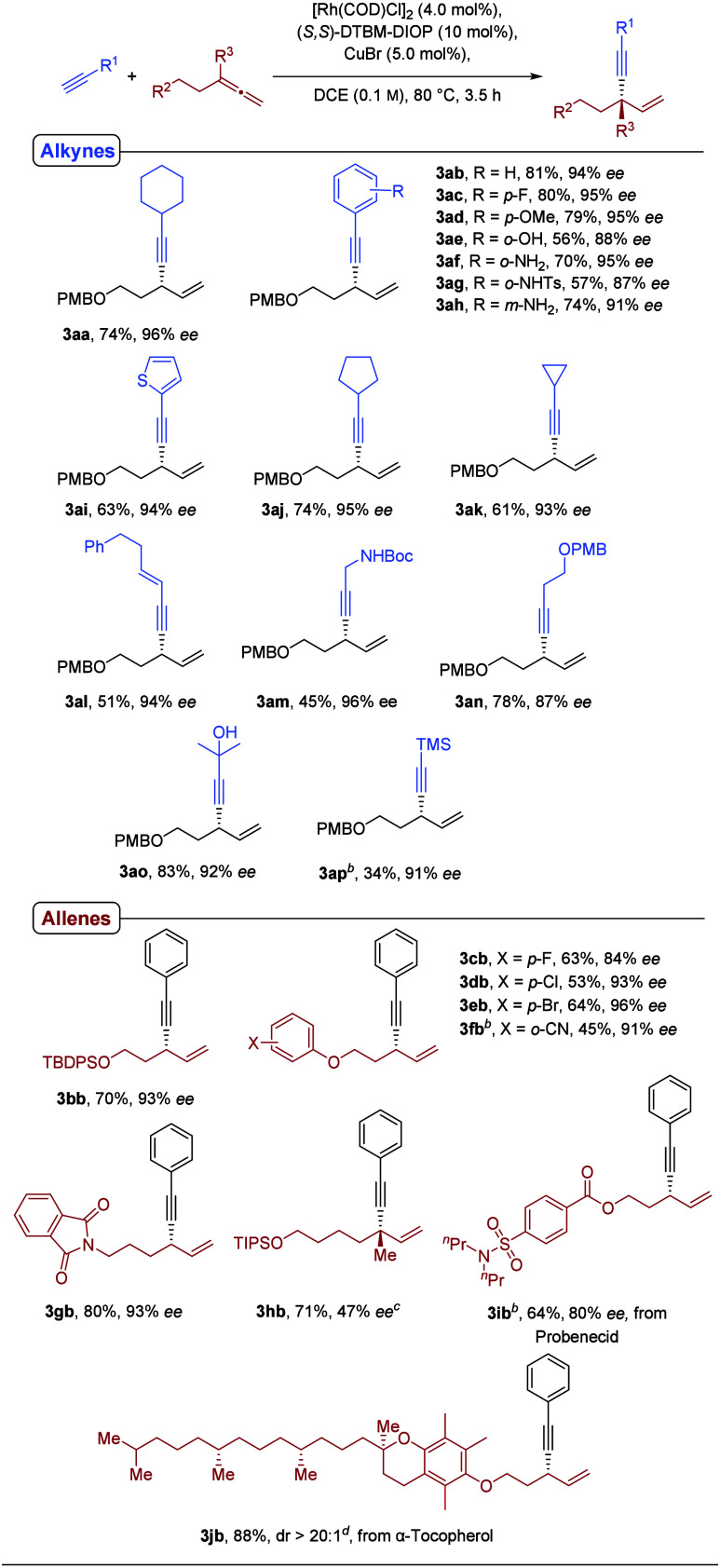
Scope of Different Terminal Alkynes (Top) and Allenes (Bottom)^a^

a All reactions were carried out on a 0.2 mmol scale. All yields are isolated yields. The *ee* values were determined using chiral HPLC. Absolute configuration assigned by analogy to substrate **4**.
b 17 h.
c Determined after removal of the TIPS-group.
d Determined via ^1^H NMR spectrum of the crude product.

The reaction was subsequently extended to aliphatic alkynes. Cyclopropyl- and cyclopentyl-substituted alkynes reacted efficiently, while a conjugated enyne furnished product **3al** in moderate yield but with excellent enantioselectivity. Boc-protected propargylamine underwent coupling with high enantioselectivity, albeit in diminished yield (45%). *Para*-methoxy-protected butynol smoothly afforded product **3an** and the method further tolerated unprotected hydroxy groups, as demonstrated by **3ao**. In contrast, TMS-acetylene delivered product **3ap** in only low yield (34%), even upon extended reaction time, likely reflecting its reduced terminal alkyne acidity.

The scope of the allene coupling partner was next examined using phenylacetylene (**2b**) as the alkyne component (Scheme 2, bottom). Allenes bearing substituted aryl ether side chains were readily engaged, providing products **3cb**–**3eb** in moderate yields and high to excellent enantioselectivities. Introduction of an *ortho*-cyano substituent (**3fb**) required an extended reaction time (17 h) and resulted in a moderate yield with good enantioselectivity. A TBDPS-protected allene was converted efficiently, affording **3bb** in 70% yield and excellent *ee*. Likewise, a phthalimide-substituted allene could be employed, delivering the corresponding product in good yield and selectivity upon prolonged reaction time. Gratifyingly, a 1,1-disubstituted allene also underwent productive coupling to give **3hb** in good yield, albeit with reduced enantioselectivity.

To further demonstrate the synthetic utility of the method, complex-molecule-derived allenes were evaluated. An allene-functionalized derivative of the gout medication probenecid underwent coupling with phenylacetylene to afford **3ib** in 64% yield and 80% *ee* after 17 h. Moreover, an α-tocopherol-derived allene furnished product **3jb** in excellent yield (88%) and outstanding diastereoselectivity (>20:1), highlighting the applicability of the protocol to late-stage functionalization of complex bioactive molecules.

To determine the absolute configuration of the enyne products, **2e** was reacted with **2f** to afford 1,4-enyne **3ef** in 95% *ee*. Acylation of the amine group with 3,5-dinitrobenzoyl chloride furnished single crystals of **4** suitable for X-ray diffraction analysis, which established the (*S*)-configuration for the major enantiomer (Scheme 3, top).

**Scheme 3:**
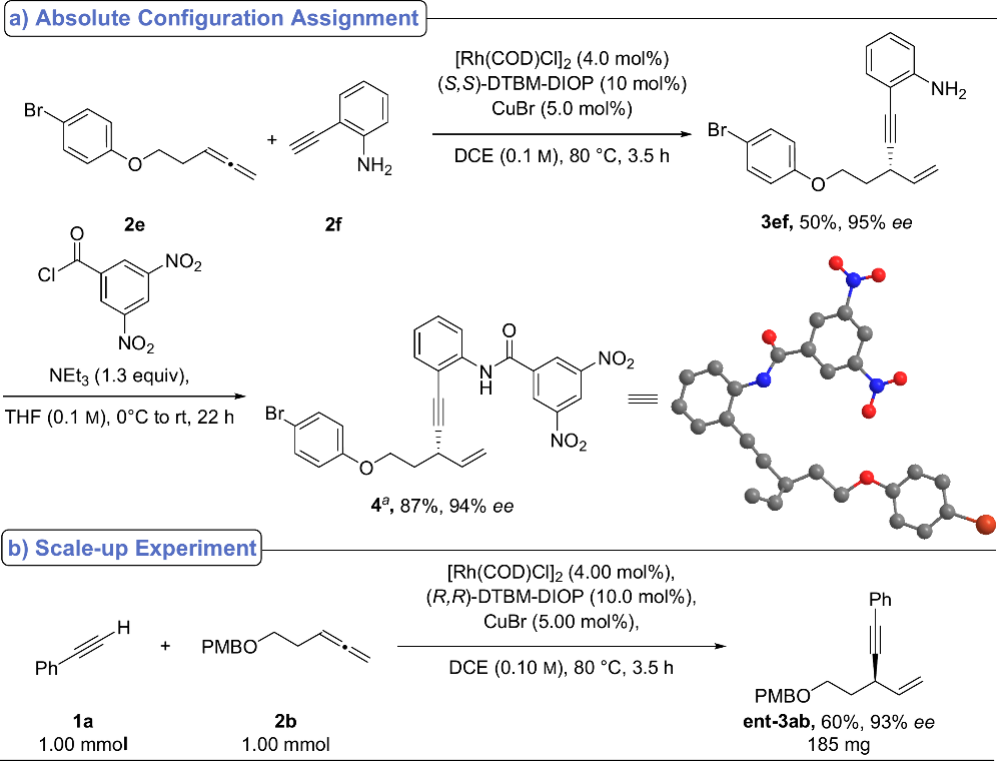
Determination of Absolute Configuration via X-ray Diffraction of 4 (Top) and Scale-up Experiment (Bottom)

a For clarity, hydrogen atoms have been omitted from the X-ray crystal structure.

A scale-up experiment was carried out using **1a** and **2b** (1.0 mmol each) under the standard reaction conditions in the presence of (*R,R*)-DTBM-DIOP (Scheme 3, bottom). The product, **ent-3ab**, was isolated in 60% yield (185 mg).

The synthetic utility of the obtained enynes was illustrated through several transformations. TMS-substituted enyne **3ap** was converted to the terminal alkyne **5**, which could undergo further functionalization. For example, a Cu­(I)-catalyzed azide–alkyne cycloaddition (“Click” reaction) produced 1,4-substituted triazole **6**, a motif widely employed in drug discovery (Scheme 4a).^11^ The *N*-benzyl-protected ortho-alkynyl aniline **2q** reacted with allene **1k** to yield enyne **3kq**, which upon Au-catalyzed cyclization afforded indole **7** (Scheme 4b). To demonstrate the orthogonal reactivity of the alkyne and alkene moieties, 2-substituted phenol **3ae** underwent Cu-catalyzed cyclization to form benzofuran **8**. Subsequent Rh-catalyzed hydroformylation of the remaining alkene delivered the linear aldehyde **9** with excellent regioselectivity (Scheme 4c). In all cases, these follow-up transformations proceeded without loss of optical purity.

**Scheme 4:**
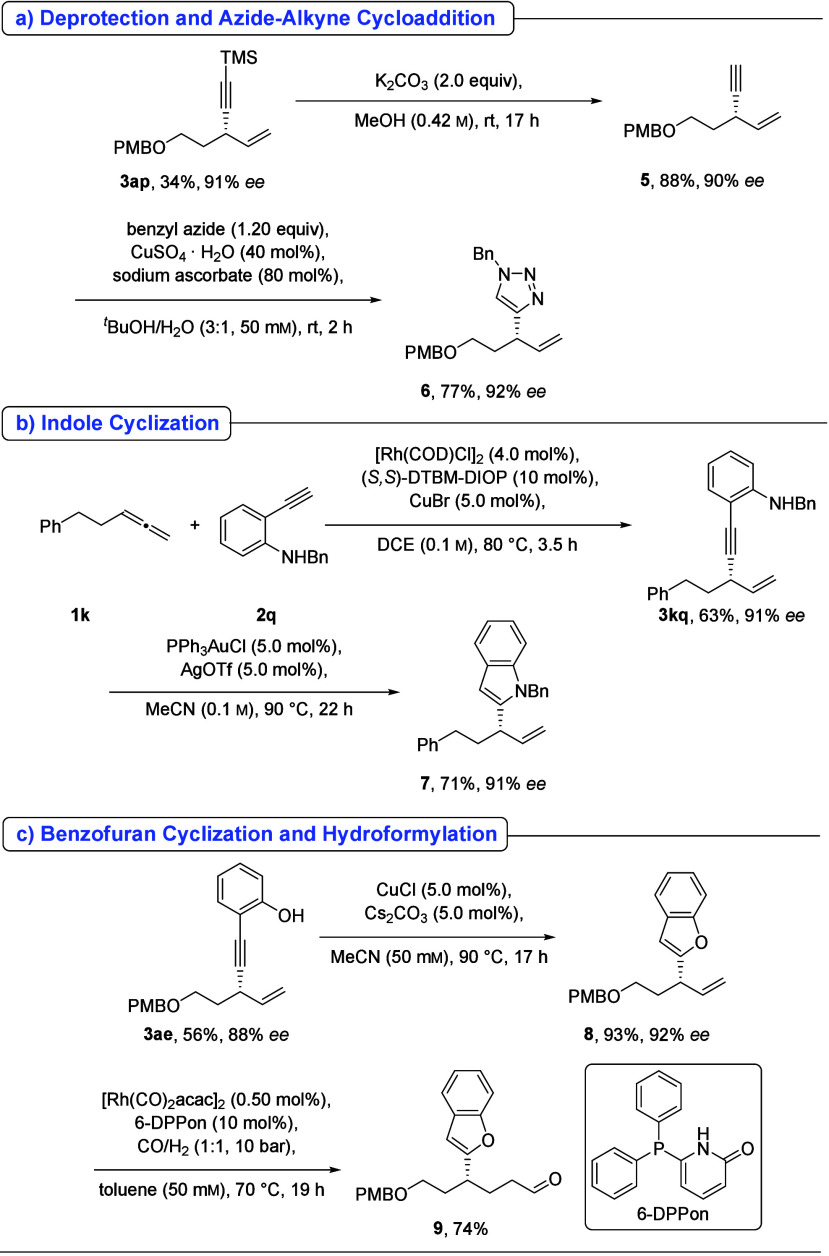
Follow-up Reactions^a^

a All yields are isolated. The *ee* values were determined using chiral HPLC. Absolute configuration assigned by analogy to substrate **4**.

A deuterium-labeling experiment was conducted to probe the reaction mechanism (Scheme 5a). The predominant incorporation of deuterium at the central carbon of the allyl unit suggests that the mechanistic pathway proceeds via a π/σ-allyl Rh-intermediate (**F** in Scheme 5c). Deuteration at the terminal position of the allyl unit may result from competitive hydrometalation of the allene, leading to a vinyl–rhodium intermediate (**E**). The possibility of a postsynthetic H–D exchange in the product can be excluded under the applied reaction conditions (see Supporting Information).

**Scheme 5:**
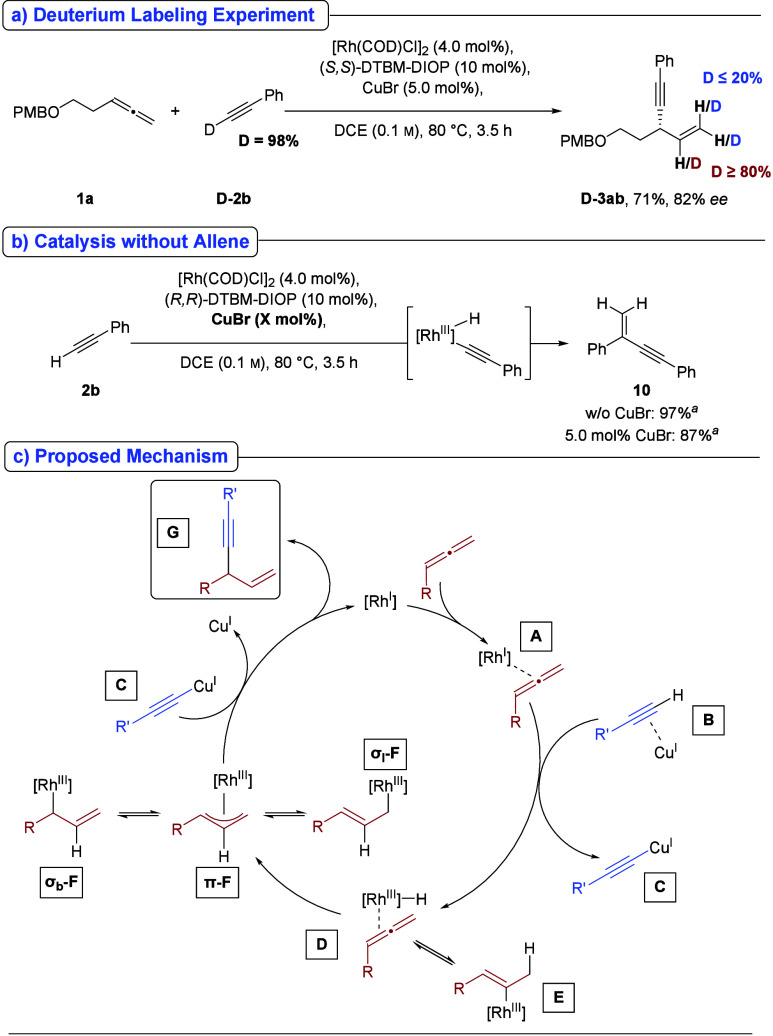
Mechanistic Experiments and Proposed Mechanism

a NMR yield was determined by ^1^H NMR spectroscopy using dibromomethane as internal standard.

However, the role of copper, which is essential for product formation (Table 1, entry 12), remains unclear. Previous DFT studies suggest that lateral coordination of a Cu­(I)-species to an alkyne can increase the acidity of the terminal C–*H* by up to 9.8 p*K*_a_ units,^12^ potentially facilitating oxidative addition to a Rh­(I)-complex to generate a Rh­(III)-alkynylhydride intermediate. Subsequent hydrometalation of the allene and reductive elimination would then furnish the product.

Interestingly, a recent report by Esteruals (2024) described Rh/xantphos-catalyzed homocoupling of terminal alkynes at 80 °C without copper salts.^13^ Consistent with this, control experiments omitting allene under our conditions led to near-quantitative alkyne dimerization, regardless of copper presence (Scheme 5b, see also Supporting Information for the use of **D-2b**). This observation challenges the proposed mechanistic sequence and indicates that the precise role of copper in the present transformation remains unresolved.

Accordingly, an alternative mechanism is proposed (Scheme 5c). Coordination of Cu­(I) to the alkyne in intermediate **B** increases the acidity of the terminal C–*H*, facilitating protonation of species **A**. This generates Cu­(I)-acetylide **C** and Rh–H species **D**, which can undergo hydrometalation to form intermediate **E** or lead to the π-allyl Rh-complex π-**F**, which exists in equilibrium with its branched (σ_b_) and linear (σ_l_) σ-isomers. Subsequent reaction of Cu­(I)-acetylide **C** with π-**F** furnishes the final product **G**, with concurrent regeneration of both the Cu- and Rh-catalysts.

In conclusion, we have developed an efficient method for the intermolecular hydroalkynylation of allenes. A diverse array of chiral 1,4-enynes bearing various functional groups was obtained with excellent regioselectivity and high enantioselectivity. Both aliphatic and aromatic terminal alkynes proved suitable, highlighting the broad substrate scope. The synthetic utility of the enyne products was demonstrated through multiple downstream transformations, particularly underscoring the orthogonal reactivity of the alkyne and alkene moieties.

This methodology represents a valuable addition to our ongoing program aimed at expanding the strategic allylation of allenes with diverse pronucleophiles and exploring applications in target-oriented synthesis.

## Supplementary Material



## Data Availability

The data underlying this study are available in the published article and its Supporting Information.

## Notes

Materials and measurements, substrate synthesis, reaction optimization, experimental details, X-ray crystallography data of **4**, spectral data and additional data. (PDF)
