# Supplementary material for: Enantioselective Rh/Cu-Catalyzed Addition of Terminal Alkynes to Allenes for the Synthesis of Chiral 1,4-Enynes
Source: Org Lett. 2026 Apr 7;28(15):4981–6. doi: 10.1021/acs.orglett.6c01096 (PMC13097249; doi:10.1021/acs.orglett.6c01096)
Supplement: Supplementary file 1 [file ol6c01096_si_001.pdf]

Enantioselective Rh/Cu-Catalyzed Addition of Terminal Alkynes to Allenes for the Synthesis of Chiral 1,4-Enynes

Martin Daiger+,[a] Janika Kromer+,[a] Samuel Kaplan[a] and Bernhard Breit*[a]

[a] Dr. M. T. Daiger, J. Kromer, S. Kaplan, Prof. Dr. B. Breit
Institut für Organische Chemie, Albert-Ludwigs-Universität Freiburg
Albertstraße 21, 79104 Freiburg im Breisgau, Germany
E-Mail: bernhard.breit@chemie.uni-freiburg.de

[+] These authors contributed equally to this work.

Table of contents

1 General Information **1**

2 Reaction Optimization **4**

3 Substrate Synthesis **17**

3.1 General Procedures 17

3.2 Alkynes 19

3.3 Monosubstituted Allenes 23

3.4 Geminal Disubstituted Allenes 29

3.5 Ligands 34

4 Catalysis Results **44**

5 Mechanistic and Control Experiments **63**

5.1 Scale-up Experiment 63

5.2 Deuterium Labeling Experiments 63

5.3 FT-IR Spectroscopy 64

5.4 Control Experiments 65

6 Follow-Up Chemistry **68**

6.1 Absolute Configuration Assignment 68

6.2 Deprotection of 3ap and Azide-Alkyne Cycloaddition 69

6.3 Indole Cyclization of 3kq 70

6.4 Benzofuran Cyclization of 3ae and Hydroformylation 71

7 Literature **72**

8 Appendix **74**

8.1 Abbreviations 74

8.2 NMR Spectra 75

8.3 HPLC Chromatograms 146

8.4 X-Ray Crystallography Data of 4 179

1 General Information

Experimental Techniques

All reactions were run in flame-dried glassware under argon atmosphere (Argon 5.0, Sauerstoffwerk Friedrichshafen). For this, the glassware was flame-dried under oil pump vacuum (0.1 mbar), cooled to room temperature and backfilled with argon. An excess of argon pressure was maintained throughout the reaction time, unless otherwise noted. For the addition of solvents and reagents, syringes and cannula were flushed with argon three times prior to use. Reactions were heated using silicone oil baths. Reaction temperatures were measured in degrees Celsius (°C) and the pressure during distillation was measured in mbar. Reaction times were recorded in minutes (min), hours (h) or days (d). All yields are isolated yields, unless noted differently. The conversion and ratios given for catalytic reactions were determined from ^{1}H-NMR spectra.

Drying and Degassing and Purification of Solvents

Solvents were purchased in analytical grade (p.a. quality) and used without further purification. For air- or moisture-sensitive reactions, AcroSeal® solvents from Thermo Fisher Scientific were used. Flasks for absolute solvents were flame-dried three times under oil pump vacuum and backfilled with argon. For use in catalytic reactions, certain solvents were further purified as described below:

1,2-Dichlorethane (DCE):	Distilled over CaH_2.
Dichlormethane:	Degassed by purging with argon.
Toluene:	Distilled over K, indication via benzophenone.
Acetonitrile:	Degassed by purging with argon.
Ethanol:	Degassed by purging with argon.
Tetrahydrofuran:	Distilled over K.

Evaporation

Reaction mixtures were evaporated at 40 °C on a Laborata 4001 efficient from Heidolph Instruments GmbH & Co. KG. Reaction mixtures containing involatile compounds were further concentrated at a rotary vane pump (pressure ≥ 5 mbar) from vacuubrand GmbH & Co. KG.

Thin Layer Chromatography

For analytical TLC, silica gel coated (0.25 mm) 60 F_{254} glass plates from MACHEREY-NAGEL GmbH & Co. KG were used. The TLC plates were visualized by the quenching of UV fluorescence (λ = 254 nm), and/or by staining with the mixture below followed by heating:

$KMnO_4$ stain: $KMnO_4$ (3.0 g), Na_2CO_3 (20 g), aq NaOH solution (5 wt %, 5.0 mL) in H_2O (300 mL).

Flash Column Chromatography (FCC)

Column Chromatography separations were carried out on silica gel 60 (0.04 - 0.063 mm, 230 - 240 mesh ASTM) from MACHEREY-NAGEL GmbH & Co. KG. The columns were made of glass and were packed (from bottom to top) with a cotton plug, a layer of sea sand, the silica gel layer and another layer of sea sand.

Reversed phase column chromatography was performed on a puriFlash 430 from INTERCHIM using a PF-30C18HP-F0025 column with a flow rate of 15 mL/min. Following procedure was applied:

- column equilibration (95% H_2O) for 10 min
- sample injection
- isocratic regime of 95% H_2O for 5 min
- linear gradient from 95% H_2O to pure MeCN over 45 min
- pure MeCN for 5 min

Nuclear Magnetic Resonance Spectroscopy (NMR)

All NMR experiments were performed on BRUKER Acance spectrometer in the analytic department of the Institute of Organic Chemistry at the University of Freiburg. All ^{1}HNMR spectra are reported in parts per million (ppm) downfield of TMS and were measured relative to the ^{1}H-NMR signals of the deuterated solvent. All ^{13}C spectra were reported in ppm relative to the ^{13}C-NMR signal of the solvent and were obtained with ^{1}H decoupling.

Mass Spectrometry

Mass spectra were recorded in the Analytical Department of the Institute of Organic Chemistry at the University of Freiburg. Experiments were performed using a Thermo Fisher Scientific Exactive mass spectrometer equipped with an Orbitrap mass analyzer. Samples were introduced either by direct infusion or via an LC/MS setup. Ionization was achieved by electrospray ionization (ESI; needle voltage 2.5-5.0 kV, ion transfer tube temperature 250 °C, sheath and auxiliary gas: N_2) or atmospheric pressure chemical ionization (APCI; corona needle current 5-10 µA, vaporizer temperature 50-400 °C, sheath gas: N_2, auxiliary gas: N_2/NH_3). High-resolution mass spectrometry (HRMS) data are reported as follows:

chemical formula, [ion]^{charge}, ionization method (ESI or APCI, positive or negative mode): calculated value, and found value.

High Pressure Liquid Chromatography (HPLC)

Chiral HPLC measurements were performed on a MERCK HITACHI HPLC apparatus (pump: L-7100, UV detector: L-7400, auto sampler: L-7200, oven: L-7360), a VARIAN Pro Star (pump: 230, UV detector: 310, auto sampler: 410, oven: 510) and a HITACHI Primade (pump: 1100, DAD detector: 1430, auto sampler: 1210, oven: 1310).

The racemic substrates for the HPLC measurements were prepared as follows:

- According to **GP 4** using *rac*-DIOP (10.0 mol%) as the ligand: **3aa**, **3ab**, **3ac**, **3ad**, **3ae**, **3ag**, **3ah**, **3ai**, **3aj**, **3ak**, **3al**, **3am**, **3an**, **3ao**, **3ap**, **3bb**, **3cb**, **3db**, **3eb**, **3fb**, **3gb**, **3hb**, **3ib**, **3kq**
- According to **GP 4** using *rac*-DTBM-DIOP (10.0 mol%) as the ligand: **3ef**, **4**, **7**
- According to **GP 5**: **ent-3ab**, **D-3ab**, **3af**, **3ap**, **5**, **6**

Optical Rotation

The angle of rotation of optically active substances was measured with a P8000-T polarimeter from A. KRÜSS OPTRONIC GMBH.

React Infrared Spectroscopy

React IR experiments were performed on a REACTIR™-45m-spectrometer from METTLER-TOLEDO. The measurement is carried out using the ATR method (*Attenuated Total Reflection*).

Crystal Structure

X-ray structures are provided by Dr. Burkhard Butschke, Krossing Group, Inorganic Chemistry Department, Albert-Ludwigs-University Freiburg (see appendix for more information).

Purchased Chemicals

Unless noted otherwise, all commercially available chemicals were purchased from common suppliers and used without further purification.

2 Reaction Optimization

Substrate Screening

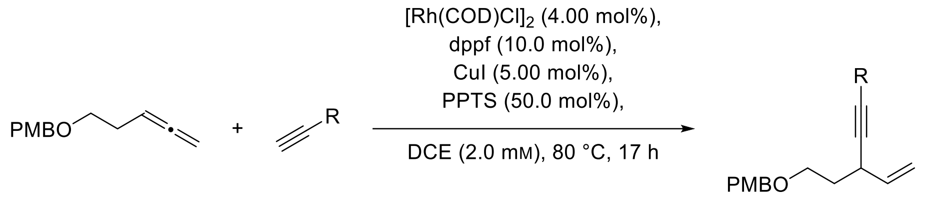

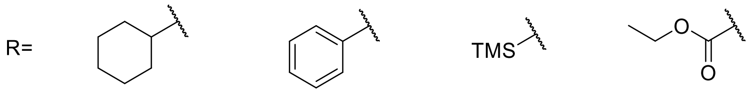

**3aa**	**3ab**	**3ap**	**S1**
13%	0%	0%	0%

Reaction conditions: Allene (200 µmol) and alkyne (300 µmol) in DCE (1.0 mL). ^{1}H-NMR yields with DBM as internal standard.

Ligand Screening

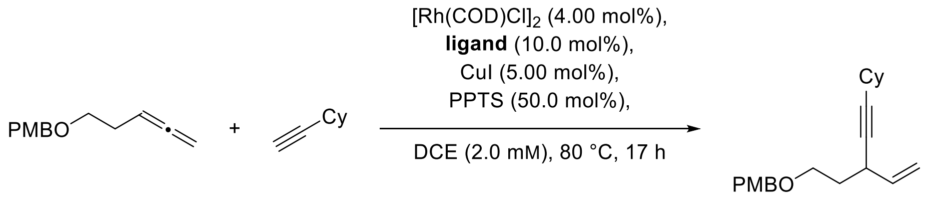

1a **2a** **3aa**

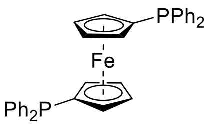

dppf
L1

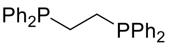

dppe
L2

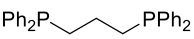

dppp
L3

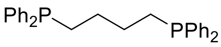

dppb
L4

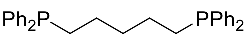

dpppe
L5

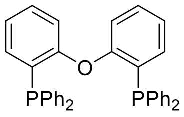

DPEphos
L6

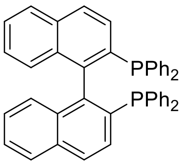

rac-BINAP
L7

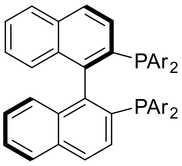

(*R*)-Tol-BINAP
Ar = 4-Me-Ph
L8

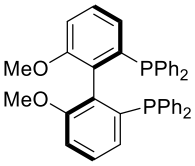

(*R*)-MeOBIPHEP
SL-A101-1
L9

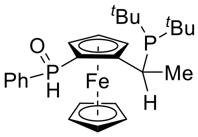

rac-JoSPOphos
SL-J688
L10

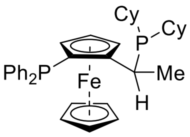

rac-Josiphos SL-J001
L11

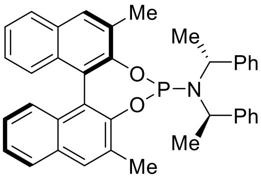

(*R*,*R*,*R*)-MeSimplePhos (Feringa)
L12

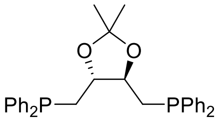

(*R*,*R*)-DIOP
L13

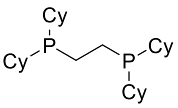

dCyPe
L14

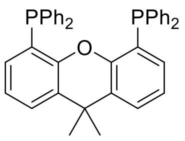

Xantphos
L15

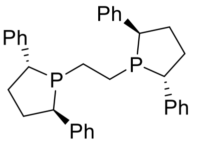

(*R*,*R*)-Ph-BPE
L16

entry[a]	ligand	yield	*ee*
1	dppf (**L1**)	11%	-
2	dppe (**L2**)	-	-
3	dppp (**L3**)	-	-
4	dppb (**L4**)	4%	-
5	dpppe (**L5**)	-	-
6	DPEphos (**L6**)	6%	-
7	*rac*-BINAP (**L7**)	-	-
8	(*R*)-Tol-BINAP (**L8**)	-	-
9	(*R*)-MeOBIPHEP (**L9**)	-	-
10	*rac*-JoSPOphos J688-1 (**L10**)	-	-
11	*rac*-Josiphos J001-1 (**L11**)	-	-
12	Feringa (**L12**)	-	-
13	(*R*,*R*)-DIOP (**L13**)	24%	87%
14	dCyPe (**L14**)	-	-
15	Xantphos (**L15**)	-	-
16	(*R*,*R*)-Ph-BPE (**L16**)	15%	43%

[a] Reaction conditions (unless otherwise specified): **1a** (200 µmol), **2a** (300 µmol), Rh source (4.00 mol%), ligand (10.0 mol%), Cu source (5.00 mol%), additive (50.0 mol%) in solvent (1.0 mL), 80 °C, 17 h. NMR yield of the product was determined by ^{1}H NMR spectroscopy using DBM as internal standard. Enantiomeric ratio determined by chiral HPLC.

DIOP Ligand Screening

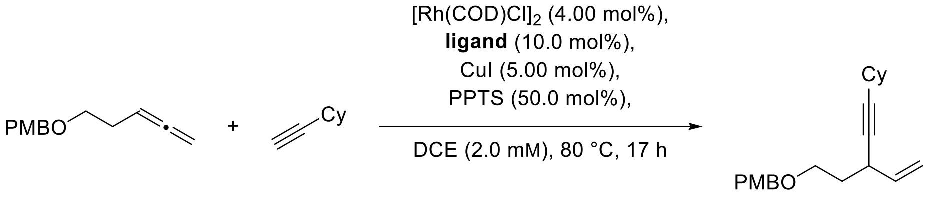

1a **2a** **3aa**

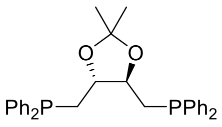

(*R*,*R*)-DIOP
L13

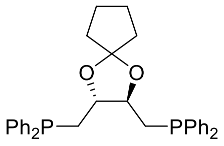

(*R*,*R*)-Cp-DIOP
L17

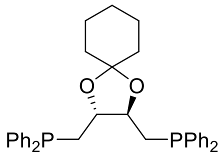

(*R*,*R*)-Cy-DIOP
L18

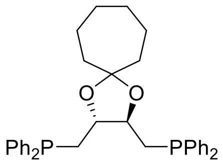

(*R*,*R*)-Chept-DIOP
L19

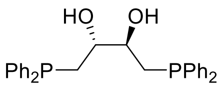

(*R*,*R*)-DIOP-Diol
L20

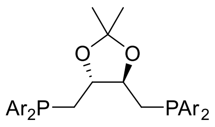

(*R*,*R*)-*p*-Me-DIOP
Ar = 4-Me-Ph
L21

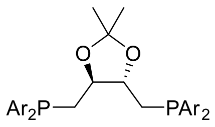

(*S*,*S*)-3,5-CF_3-DIOP
Ar = 3,5-CF_3-Ph
L22

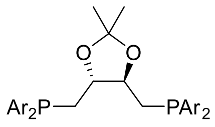

(*R*,*R*)-Naph-DIOP
Ar = Nap
L23

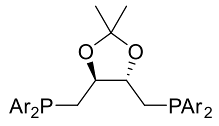

(*S*,*S*)-DTBM-DIOP
Ar = 3,5-^{t}Bu-4-MeO-Ph
L24

entry[a]	ligand	yield	*ee*
1	(*R*,*R*)-DIOP (**L13**)	24%	87%
2	(*R*,*R*)-Cp-DIOP (**L17**)	27%	87%
3	(*R*,*R*)-Cy-DIOP (**L18**)	40%	87%
4	(*R*,*R*)-Chept-DIOP (**L19**)	37%	90%
5	(*R*,*R*)-DIOP-Diol (**L20**)	-	-
6	(*R*,*R*)-*p*-Me-DIOP (**L22**)	25%	88%
7	(*S*,*S*)-3,5-CF_3-DIOP (**L23**)	-	-
8	(*R*,*R*)-Naph-DIOP (**L24**)	11%	-
9	(*S*,*S*)-DTBM-DIOP (**L25**)	46%	89%

[a] Reaction conditions (unless otherwise specified): **1a** (200 µmol), **2a** (300 µmol), Rh source (4.00 mol%), ligand (10.0 mol%), Cu source (5.00 mol%), additive (50.0 mol%) in solvent (1.0 mL), 80 °C, 17 h. NMR yield of the product was determined by ^{1}H NMR spectroscopy using DBM as internal standard. Enantiomeric ratio determined by chiral HPLC.

Rhodium Source Screening

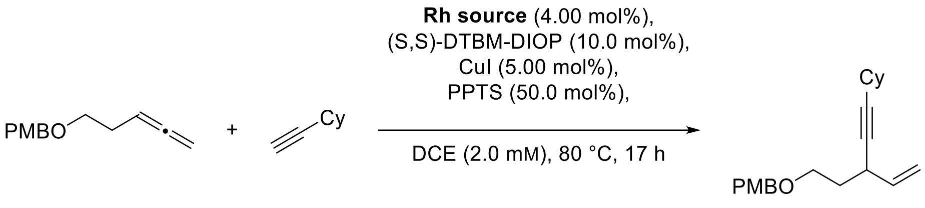

1a **2a** **3aa**

entry[a]	Rh source	yield	*ee*
1	$[Rh(COD)Cl]_2$	46%	89%
2	$[Rh(COD)_2]OTf$	35%	83%
3	$[Rh(nbd)_2]ClO_4$	21%	56%
4	$[Rh(COD)_2]BF_4$	31%	80%
5	$[Rh(OH)(COD)]_2$	34%	81%
6	[Rh(nbd)(acac)]	32%	81%
7	$Rh(COD)_2]SbF_6$	12%	82%
8	$[Rh(COD)(OAc)]_2$	42%	81%

[a] Reaction conditions (unless otherwise specified): **1a** (200 µmol), **2a** (300 µmol), Rh source (4.00 mol%), ligand (10.0 mol%), Cu source (5.00 mol%), additive (50.0 mol%) in solvent (1.0 mL), 80 °C, 17 h. NMR yield of the product was determined by ^{1}H NMR spectroscopy using DBM as internal standard. Enantiomeric ratio determined by chiral HPLC.

Copper Source Screening

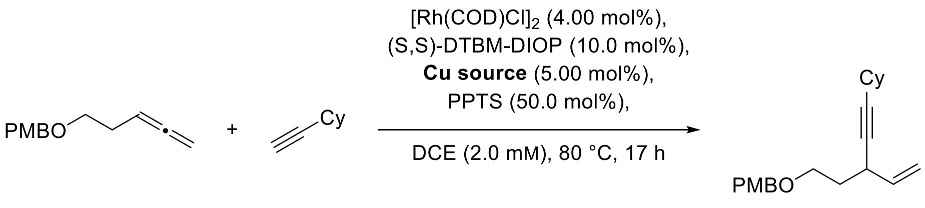

1a **2a** **3aa**

entry[a]	Cu source	yield	*ee*
1	CuI	46%	89%
2	CuCl	38%	93%
3	CuBr	43%	94%
4	$CuBr \cdot SMe_2$	49%	81%
5	$Cu(OTf)_2$	8%	-
6	$[CuBr(PPh)_3]$	45%	93%
7	$[Cu(COD)Cl]_2$	31%	91%
8	$Cu(MeCN)_4BF_4$	26%	92%
9	CuTC	31%	81%

[a] Reaction conditions (unless otherwise specified): **1a** (200 μmol), **2a** (300 μmol), Rh source (4.00 mol%), ligand (10.0 mol%), Cu source (5.00 mol%), additive (50.0 mol%) in solvent (1.0 mL), 80 °C, 17 h. NMR yield of the product was determined by ^{1}H NMR spectroscopy using DBM as internal standard. Enantiomeric ratio determined by chiral HPLC.

Additive Screening

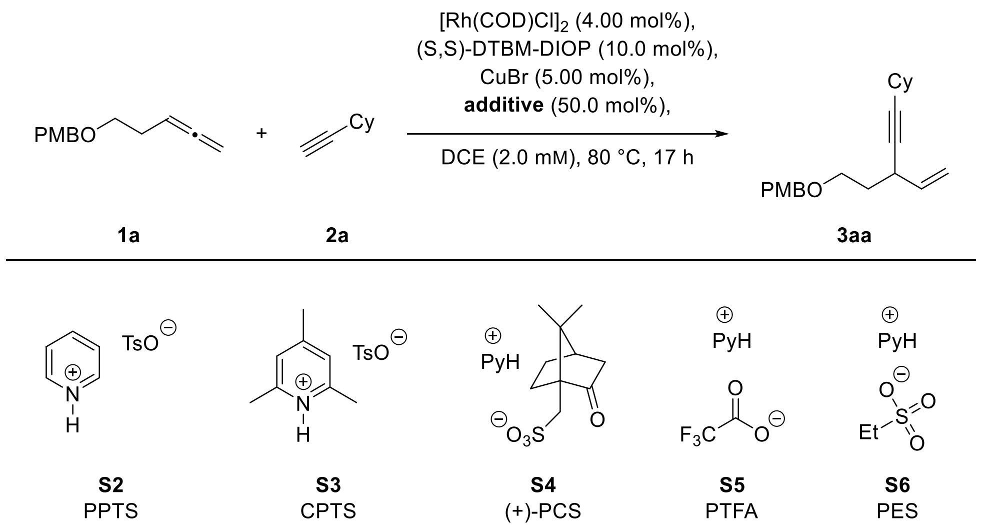

entry[a]	additive	yield	*ee*
1	PPTS (**S2**)	43%	94%
2	CPTS (**S3**)	39%	94%
3	(+)-PCS (**S4**)	44%	94%
4	PTFA (**S5**)	-	-
5	PES (**S6**)	17%	-
6	TFA	23%	-
7	$(PhO)_2PO_2H$	17%	-
8	K_2HPO_4	18%	-
9	NaH_2PO_4 × H2O	-	-
10	*t*-BuOK	-	-
11	Pyridin	-	-
12	NEt_3	-	-
13	Piperidin	-	-
14	DIPEA	16%	-
15	K_2CO_3	10%	-
16	NH_4HCO_3	-	-
17	$(Bu)_4NHSO_4$	27%	-
18	$NH_4[PF_6]$	46%	90%
19	$KHCO_3$	-	-
20	$NaHCO_3$	57%	94%
21	$NaHCO_3$ (20 mol%)	10%	-
22	$KHSO_4$	44%	91%
23	$NaHSO_4$	51%	95%
24	TFA (20 mol%)	48%	96%
25	TFA (10 mol%)	58%	97%

[a] Reaction conditions (unless otherwise specified): **1a** (200 µmol), **2a** (alkin) (300 µmol), Rh source (4.00 mol%), ligand (10.0 mol%), Cu source (5.00 mol%), additive (X mol%) in solvent (1.0 mL), 80 °C, 17 h. NMR yield of the product was determined by ^1H NMR spectroscopy using DBM as internal standard. Enantiomeric ratio determined by chiral HPLC.

Solvent Screening

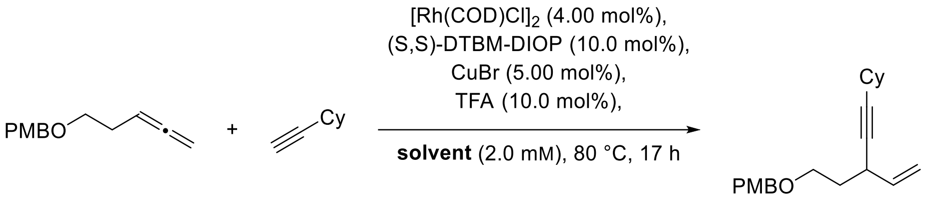

1a **2a** **3aa**

entry[a]	solvent	yield	*ee*
1	DCE	58%	97%
2	DCM	54%	96%
3	EtOH	39%	95%
4	DCM:EtOH (3:1)	50%	96%
5	THF	27%	-
6	Toluol	15%	-
7	MeCN	11%	-

[a] Reaction conditions (unless otherwise specified): **1a** (200 µmol), **2a** (300 µmol), Rh source (4.00 mol%), ligand (10.0 mol%), Cu source (5.00 mol%), additive (10.0 mol%) in solvent (1.0 mL), 80 °C, 17 h. NMR yield of the product was determined by ^{1}H NMR spectroscopy using DBM as internal standard. Enantiomeric ratio determined by chiral HPLC.

Temperature Screening

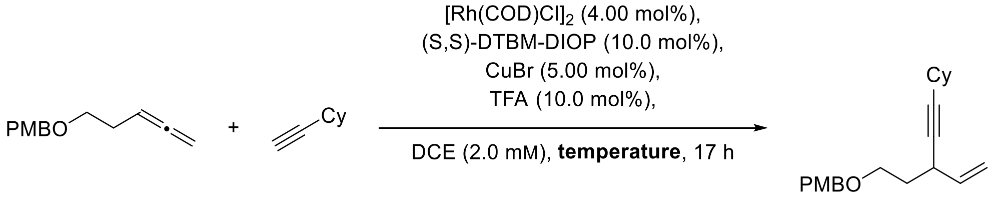

1a **2a** **3aa**

entry[a]	temperature	yield	*ee*
1	60 °C	36%	97%
2	70 °C	37%	96%
3	80 °C	58%	97%
4	90 °C	56%	96%
5	100 °C	37%	94%

[a] Reaction conditions (unless otherwise specified): **1a** (200 µmol), **2a** (300 µmol), Rh source (4.00 mol%), ligand (10.0 mol%), Cu source (5.00 mol%), additive (10.0 mol%) in solvent (1.0 mL), 17 h. NMR yield of the product was determined by ^{1}H NMR spectroscopy using DBM as internal standard. Enantiomeric ratio determined by chiral HPLC.

Ligand Stoichiometry Screening

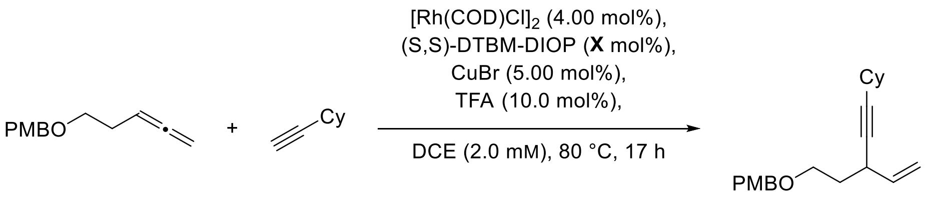

1a **2a** **3aa**

entry[a]	stoichiometry	yield
1	8.00 mol%	49%
2	10.0 mol%	58%
3	15.0 mol%	-
4	20.0 mol%	-

[a] Reaction conditions (unless otherwise specified): **1a** (200 µmol), **2a** (300 µmol), Rh source (4.00 mol%), ligand (X mol%), Cu source (5.00 mol%), additive (10.0 mol%) in solvent (1.0 mL), 80 °C, 17 h. NMR yield of the product was determined by ^{1}H NMR spectroscopy using DBM as internal standard. Enantiomeric ratio determined by chiral HPLC.

Concentration Screening

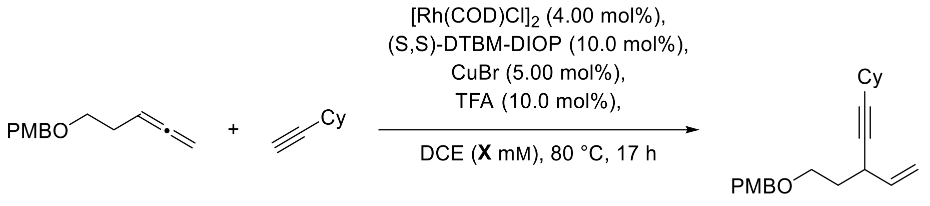

1a **2a** **3aa**

entry[a]	concentration	yield	*ee*
1	0.40 M	48%	-
2	0.20 M	58%	97%
3	0.10 M	72%	96%
4	0.05 M	58%	-
5	0.01 M	58%	-

[a] Reaction conditions (unless otherwise specified): **1a** (200 µmol), **2a** (300 µmol), Rh source (4.00 mol%), ligand (10.0 mol%), Cu source (5.00 mol%), additive (10.0 mol%) in solvent (X mL), 80°C, 17 h. NMR yield of the product was determined by ^{1}H NMR spectroscopy using DBM as internal standard. Enantiomeric ratio determined by chiral HPLC.

Ligand Screening with Geminal Disubstituted Allenes

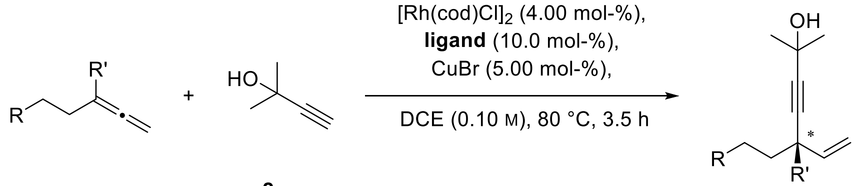

2o

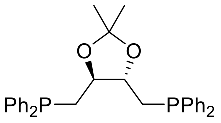

(*S*,*S*)-DIOP
ent-L13

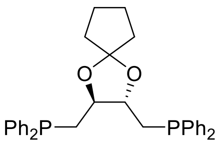

(*S*,*S*)-Cp-DIOP
ent-L17

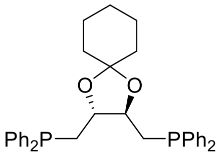

(*R*,*R*)-Cy-DIOP
L18

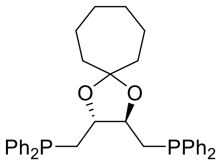

(*S*,*S*)-Chept-DIOP
L19

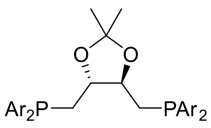

(*R*,*R*)-Naph-DIOP
Ar = Nap
L23

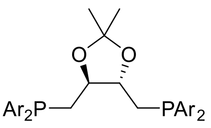

(*S*,*S*)-DTBM-DIOP
Ar = 3,5-^{t}Bu-4-MeO-Ph
L24

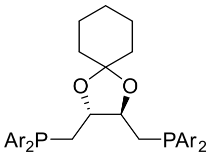

(*R*,*R*)-Cy-DTBM-DIOP
Ar = 3,5-^{t}Bu-4-MeO-Ph
L25

entry[a]	allene	ligand	yield	*ee*
1		*rac*-DIOP (**rac-L13**)	61%	-
2		(*S*,*S*)-DIOP (**ent-L13**)	74%	18%
3		(*S*,*S*)-Cp-DIOP (**ent-L17**)	75%	15%
4	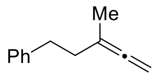 **S7**	(*R*,*R*)-Cy-DIOP (**L18**)	60%	17%
5		(*S*,*S*)-Chept-DIOP (**L19**)	73%	18%
6		(*R*,*R*)-Naph-DIOP (**L23**)	46%	23%
7		(*S*,*S*)-DTBM-DIOP (**L24**)	69%	62%
8		(*R*,*R*)-Cy-DTBM-DIOP (**L25**)	58%	60%
9	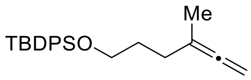 **S8**	*rac*-DIOP (**rac-L13**)	55%	-
10		(*S*,*S*)-DTBM-DIOP (**L24**)	38%	59%
11		*rac*-DIOP (**rac-L13**)	41%	-
12	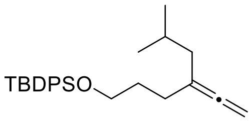 **S9**	(*S*,*S*)-DIOP (**ent-L13**)	52%	28%
13		(*S*,*S*)-DTBM-DIOP (**L24**)	13%	14%
14		*rac*-DIOP (**rac-L13**)	40%	-
15	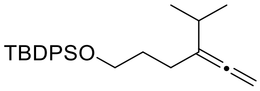 **S10**	(*S*,*S*)-DIOP (**ent-L13**)	46%	20%
16		(*S*,*S*)-DTBM-DIOP (**L24**)	0%	-
17	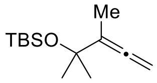 **S11**	*rac*-DIOP (**rac-L13**)	5%[b]	-
18		(*S*,*S*)-DTBM-DIOP (**L24**)	0%	-

[a] Reaction conditions (unless otherwise specified): Allene (200 µmol), alkyne (200 µmol), Rh source (4.00 mol%), ligand (10.0 mol%), Cu source (5.00 mol%) in solvent (2.0 mL), 80 °C, 3.5 h. The yield shown is the isolated yield. Enantiomeric ratio determined by chiral HPLC. [b] NMR yield of the product was determined by ^{1}H NMR spectroscopy using DBM as internal standard.

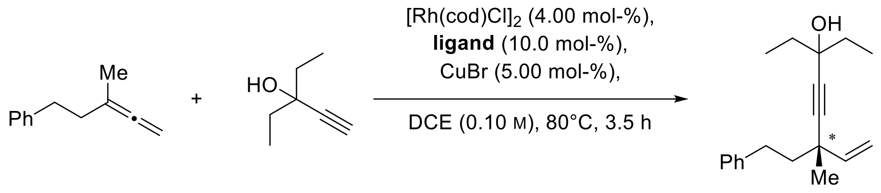

S7 **S12** **S13**

rac-DIOP (**rac-L13**):	63%
(*S,S*)-DTBM-DIOP (**L24**):	66%<br>*ee* could not be detemined

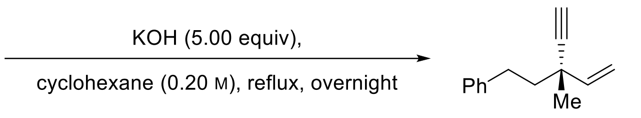

S14, 70%, 58% *ee*

3 Substrate Synthesis

3.1 General Procedures

GP 1: Deprotection of TMS alkyne

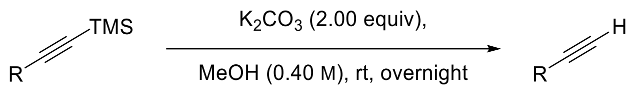

To a stirred solution of TMS alkyne (1.00 equiv) in MeOH (0.40 M) was added K_2CO_3 (2.00 equiv). The suspension was stirred at rt overnight. Then, water (0.40 M) was added and the mixture was extracted with EtOAc (3 × 0.40 M). The combined organic extracts were washed with brine (3 × 0.40 M), dried over Na_2SO_4 and the solvent was removed under reduced pressure. The residue was purified by column chromatography.

GP 2: MITSUNOBU reaction

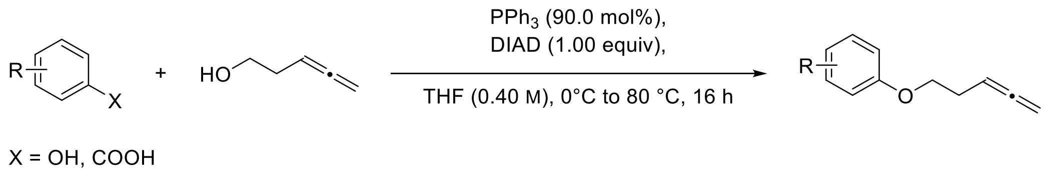

A solution of PPh_3 (0.900 equiv), electrophile (1.20 equiv) and nucleophile (1.00 equiv) in THF (0.40 M) was cooled to 0 °C. DIAD (1.00 equiv) was added dropwise and the reaction mixture was heated to 80 °C for 16 h. The reaction mixture was allowed to come to rt and the solvent was removed under reduced pressure. The crude was purified by flash column chromatography.

GP 3: Synthesis of geminal disubstituted allenes

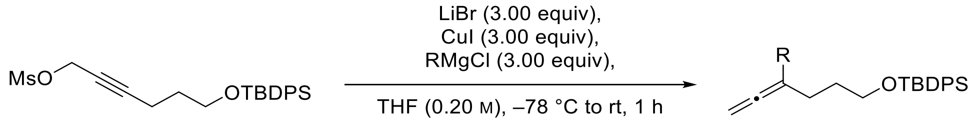

LiBr (0.650 g, 7.50 mmol, 3.00 equiv) was activated under vacuum with a heat gun. After cooling down to rt, CuI (1.43 g, 7.50 mmol, 3.00 equiv) and THF (15 mL) were added. The resulting suspension was cooled to −78 °C and RMgCl (3.00 equiv) was added dropwise. The reaction mixture was stirred at −78 °C for 2 h. Afterwards, a solution of 6-((*tert*-butyldiphenylsilyl)oxy)hex-2-yn-1-yl methanesulfonate

(1.08 g, 2.50 mmol, 1.00 equiv) in THF (5.0 mL) was added. The reaction mixture was stirred at −78 °C for 1 h, until it was allowed to warm up to rt. An aqueous saturated solution of NH_4Cl (15 mL) and NH_4OH (15 mL) were added. The layers were separated, and the aqueous layer was further extracted with Et_2O (3 × 15 mL). The combined organic phases were dried over Na_2SO_4, filtered and concentrated under reduced pressure. The crude product was purified by flash column chromatography.

GP 4: Hydroalkynylation of Allenes

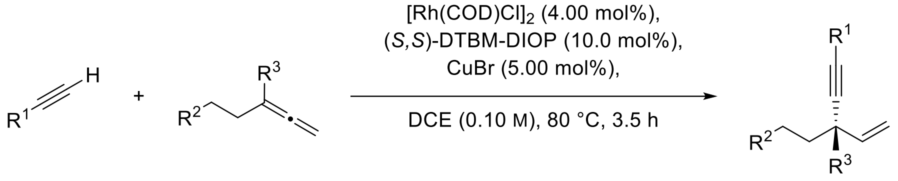

In a SCHLENK-tube $[Rh(COD)Cl]_2$ (3.95 mg, 8.00 µmol, 4.00 mol%), (*S,S*)-DTBM-DIOP (21.4 mg, 20.0 µmol. 10.0 mol%) and CuBr (1.45 mg, 10.0 µmol, 5.00 mol%) were dissolved in DCE (2 mL, 0.1 M). The mixture was stirred for 20 min. The allene (200 µmol, 1.00 equiv) was added to the solution, followed by the addition of the alkyne (200 µmol, 1.00 equiv). The reaction mixture was heated to 80 °C for 3.5 h. After cooling to rt, the solvent was removed under reduced pressure and the residue was purified by flash column chromatography.

GP5: Synthesis of Racemic 1,4-Enynes for HPLC Analysis

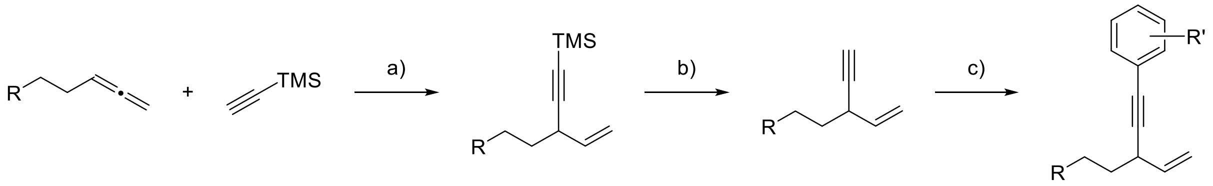

a) Dichlorido(2,9-bis(2,4,6-triisopropylphenyl)-1,10-phenanthroline)cobalt(II) (3.00 mol%) was dissolved in THF (0.25 M) and EtMgBr (3.0 M in Et_2O, 24.0 mol%) and a solution of allene (1.00 equiv) and alkyne (1.20 equiv) in THF (0.17 M) were added. The mixture was stirred at 70 °C overnight, quenched with a saturated aqueous NH_4Cl solution and the aqueous phase was extracted with EtOAc. The combined organic extracts were washed with water and brine, dried over Na_2SO_4 and the solvent was removed under reduced pressure. The residue was purified by column chromatography.

This procedure is based on a previously reported one.[1]

b) To a stirred solution of TMS alkyne (1.00 equiv) in MeOH (0.40 M) was added K_2CO_3 (2.00 equiv). The suspension was stirred at rt overnight. Then, water (0.40 M) was added and the mixture was extracted with EtOAc. The combined organic extracts were washed with brine, dried over Na_2SO_4 and the solvent was removed under reduced pressure. The residue was purified by column chromatography.

c) A mixture of CuI (10.0 mol%), $Pd(PPh_3)_2Cl_2$ (5.00 mol%) and NEt_3 (0.17 M) were stirred at rt for 15 min. Aryliodide (1.10 equiv), alkyne (1.00 equiv) and NEt_3 (0.17 M) were added and the mixture was stirred at rt overnight. Then, the suspension was filtered through a pad of celite and diluted with Et_2O and aqueous HCl solution (1.0 M). The aqueous phase was extracted with Et_2O, the combined organic extracts were dried over Na_2SO_4 and the solvent was removed under reduced pressure. The residue was purified by flash column chromatography.

This procedure is based on a previously reported one.[2]

3.2 Alkynes

1-((But-3-yn-1-yloxy)methyl)-4-methoxybenzene (2n)

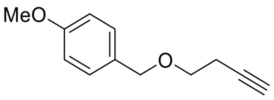

2n

NaH (360 mg, 9.00 mmol, 1.80 equiv) was added to a solution of but-3-yn-1-ol (378 µL, 350 mg, 5.00 mmol, 1.00 equiv) in dry THF (14 mL) at 0 °C. *n*-Bu_4NI (24.0 mg, 65.0 µmol, 10.0 mol%) and *para*-methoxy benzyl chloride (1.02 mL, 1.18 g, 1.50 equiv) were added and the solution was allowed to warm up to rt and stirred for 15 h. The reaction was quenched with aqueous saturated NH_4Cl solution (6.00 mL) and extracted with Et_2O (3 × 6.0 mL). The combined organic extracts were washed with H_2O and brine, dried over Na_2SO_4 and the solvent was removed under reduced pressure. The residue was purified by flash column chromatography [SiO_2, *n*-pentane/Et_2O 15:1 v/v, R_f = 0.30 (*n*-pentane/Et_2O 15:1 v/v)] to obtain the product (640 mg, 3.60 mmol, 72%) as a colorless oil.

^{1}H-NMR (500 MHz, $CDCl_3$) δ = 7.36 – 7.23 (m, 2H), 6.95 – 6.86 (m, 2H), 4.49 (s, 2H), 3.80 (s, 3H), 3.58 (t, J = 7.0 Hz, 2H), 2.49 (td, J = 7.0, 2.7 Hz, 3H), 2.00 (t, J = 2.7 Hz, 1H) ppm.

^{13}C-NMR (126 MHz, $CDCl_3$) δ = 159.4, 130.2, 129.4, 113.9, 81.4, 72.7, 69.4, 67.9, 55.3, 20.0 ppm.

HR-MS ($C_{12}H_{14}O_2^+$; $[M]^+$, pos. APCI): calcd: 190.0988, found: 190.0989.

The analytical data corresponded to the reported literature values.[3]

2-Erthynylphenol (2e)

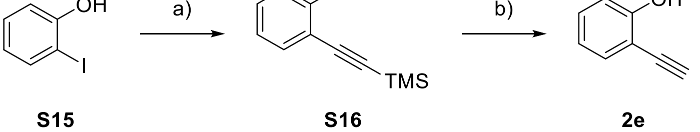

S15 **S16** **2e**

a) Trimethylsilylacetylene (3.33 mL, 2.36 g, 24.0 mmol, 1.20 equiv), CuI (98%, 194 mg, 1.00 mmol, 5.00 mol%) and $PdCl_2(PPh_3)_2$ (143 mg, 200 µmol, 1.00 mol%) were dissolved in THF (30 mL). NEt_3 (6.97 mL, 5.06 g, 50.0 mmol, 2.50 equiv) was added slowly. After stirring the reaction mixture at rt for 30 min, a solution of 2-iodophenol (98%, 4.49 g, 20.0 mmol, 1.00 equiv) in THF (10 mL) was added dropwise. The reaction mixture was stirred at reflux overnight. After cooling to rt, water (20 mL) and aqueous HCl solution (2 M, 20 mL) were added. The aqueous layer was separated and the organic phase was extracted with EtOAc (3 × 50 mL). The combined organic extracts were washed with and brine (4 × 25 mL), dried over Na_2SO_4 and the solvent was removed under reduced pressure. The residue was purified by column chromatography [SiO_2, *n*-pentane/Et_2O 99:1 to 95:1 v/v, R_f = 0.53 (*n*-pentane/Et_2O 95:1 v/v)] to obtain the product **S16** (3.24 g, 17.0 mmol, 85%) as a brown oil.

Analytical data are in accordance with literature.[4]

b) To a stirred solution of **S16** (2.91 g, 15.3 mmol, 1.00 equiv) in MeOH (50 mL) was added K_2CO_3 (2.11 g, 15.3 mmol, 1.00 equiv). The suspension was stirred at rt overnight. Then, aqueous saturated NH_4Cl solution (50 mL) was added and the mixture was extracted with Et_2O (3 × 50 mL). The combined organic extracts were washed with water (2 × 50 mL), dried over Na_2SO_4 and the solvent was removed under reduced pressure. The residue was purified by column chromatography [SiO_2, *n*-pentane/Et_2O 9:1 v/v, R_f = 0.33 (*n*-pentane/Et_2O 9:1 v/v)] to obtain the product **2e** (1.67 g, 14.1 mmol, 92%) as a brown oil.

^{1}H-NMR (400 MHz, $CDCl_3$) δ = 7.39 (dd, *J* = 7.7, 1.7 Hz, 1H), 7.28 (ddd, *J* = 8.2, 7.4, 1.6 Hz, 1H), 6.96 (ddd, *J* = 8.2, 1.1, 0.4 Hz, 1H), 6.88 (td, *J* = 7.5, 1.1 Hz, 1H), 5.81 (s, 1H), 3.47 (s, 1H) ppm.

^{13}C-NMR (101 MHz, $CDCl_3$) δ = 157.4, 132.1, 131.0, 120.4, 114.9, 108.3, 84.4, 78.4 ppm.

HR-MS ($C_8H_7O^+$; $[M+H]^+$, pos. APCI): calcd: 119.0497, found: 119.0491.

Analytical data are in accordance with literature.[5]

2-Ethynylaniline (2f), *N*-(2-ethynylphenyl)-4-methylbenzenesulfonamide (2g) and *N*-benzyl-2-ethynylaniline (2q)

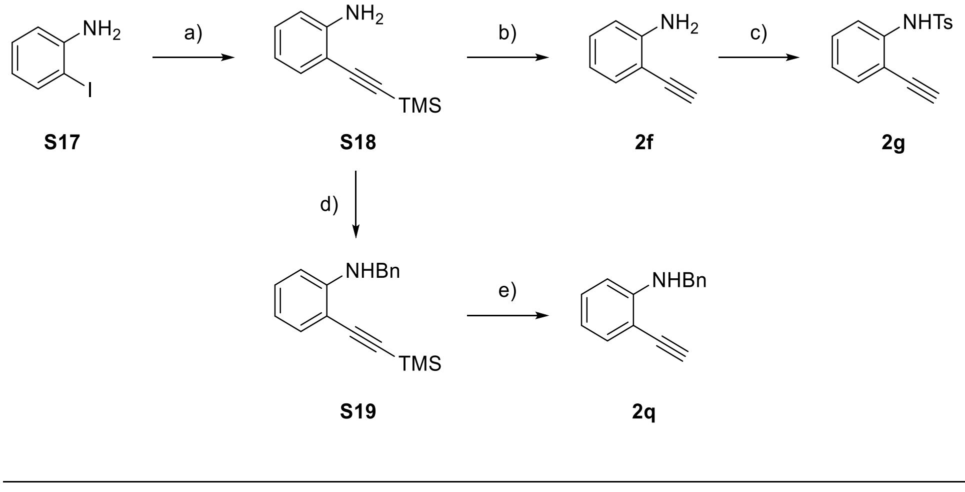

a) 2-Iodoaniline (4.38 g, 20.0 mmol, 1.00 equiv), trimethylsilylacetylene (4.16 mL, 2.95 g, 30.0 mmol, 1.50 equiv), $PdCl_2(PPh_3)_2$ (143 mg, 200 µmol, 1.00 mol%) and CuI (190 mg, 1.00 mmol, 5.00 mol%) were suspended in NEt_3 (50 mL). The mixture was stirred at rt overnight. Then, the solvent was removed under reduced pressure and the residue was purified by column chromatography [SiO_2, *n*-pentane/EtOAc 19:1 v/v, R_f = 0.34 (*n*-pentane/Et_2O 19:1 v/v)] to obtain the product **S18** (3.70 g, 19.5 mmol, 98%) as a yellow oil.

This procedure is based on a previously reported one.[6] Analytical data are in accordance with literature.[7]

b) Synthesis according to **GP 1** by the addition of K_2CO_3 (2.19 g, 15.8 mmol, 2.00 equiv) to a solution of **S18** (1.50 g, 7.92 mmol, 1.00 equiv) in MeOH (20 mL, 0.40 M). Purification by flash column chromatography [SiO_2, *n*-pentane/Et_2O 4:1 v/v, R_f = 0.33 (*n*-pentane/Et_2O 4:1 v/v)] gave the title compound **2f** (776 mg, 6.62 mmol, 84%) as a yellow oil.

^{1}H-NMR (500 MHz, $CDCl_3$) δ = 7.33 (dd, *J* = 7.7, 1.6 Hz, 1H), 7.18 – 7.11 (m, 1H), 6.72 – 6.66 (m, 2H), 4.24 (s, 2H), 3.39 (s, 1H) ppm.

^{13}C-NMR (126 MHz, $CDCl_3$) δ = 148.6, 132.7, 130.2, 117.9, 114.4, 106.7, 82.5, 80.7 ppm.

HR-MS ($C_8H_8N^+$; $[M+H]^+$, pos. APCI): calcd: 118.0657, found: 118.0651.

Analytical data are in accordance with literature.[7]

c) TsCl (1.89 g, 9.93 mmol, 1.50 equiv) and pyridine (801 µL, 785 mg, 9.93 mg, 1.50 equiv) were added to a solution of **2f** (776 mg, 6.62 mmol, 1.00 equiv) in CH_2Cl_2 (30 mL). The mixture was stirred at rt overnight. Then, the solution was poured into an aqueous saturated NH_4Cl solution (100 mL) and extracted with CH_2Cl_2 (3 × 30 mL). The combined organic extracts were washed with brine (60 mL), dried over Na_2SO_4 and the solvent was removed under reduced pressure. The residue was purified by flash column chromatography [SiO_2, *n*-pentane/Et_2O 9:1 to 4:1 v/v, R_f = 0.22 (*n*-pentane/Et_2O 4:1 v/v)] to obtain the product **2g** (1.75 g, 6.45 mmol, 97%) as a colorless solid.

This procedure is based on a previously reported one.[8]

^{1}H-NMR (500 MHz, $CDCl_3$) δ = 7.69 (d, *J* = 8.5 Hz, 2H), 7.59 (dd, *J* = 8.3, 1.2 Hz, 1H), 7.34 (dd, *J* = 7.7, 1.6 Hz, 1H), 7.31 – 7.26 (m, 1H), 7.23 – 7.20 (m, 2H), 7.01 (td, *J* = 7.6, 1.1 Hz, 1H), 3.37 (s, 1H), 2.37 (s, 3H) ppm.

^{13}C-NMR (126 MHz, $CDCl_3$) δ = 144.2, 138.6, 136.0, 132.6, 130.3, 129.7, 127.4, 124.2, 119.3, 112.7, 84.5, 78.7, 21.6 ppm.

HR-MS ($C_{15}H_{14}NO_2S^+$; $[M+H]^+$, pos. APCI): calcd: 272.0745, found: 272.0738.

Analytical data are in accordance with literature.[9]

d) Benzylaldehyde (2.29 mL, 2.41 g, 22.7 mmol, 1.20 equiv) was added to a solution of **S18** (3.57 mg, 18.9 mmol, 1.00 equiv) in AcOH (47 mL). The reaction was stirred at rt for 1 h and cooled to 0 °C. $NaBH_4$ (1.43 g, 37.8 mmol, 2.00 equiv) was added, the mixture was stirred at rt for 1 h and quenched with aqueous NaOH solution (2 M, 50 mL). The mixture was extracted with EtOAc (3 × 100 mL), the combined organic extracts were dried over Na_2SO_4 and the solvent was removed under reduced pressure. The target compound **S19** (5.27 g, 18.9 mmol, quant) was obtained as a yellow oil and used in the next step without further purification.

This procedure is based on a previously reported one.[10] Analytical data are in accordance with literature.[11]

e) Synthesis according to **GP 1** by the addition of K_2CO_3 (5.22 g, 37.8 mmol, 2.00 equiv) to a solution of **S19** (5.27 g, 18.9 mmol, 1.00 equiv) in MeOH (47 mL). Purification by flash column chromatography [SiO_2, *n*-pentane/Et_2O 49:1 to 29:1 v/v, R_f = 0.46 (*n*-pentane/Et_2O 19:1 v/v)] gave the title compound **2q** (3.43 mg, 16.6 mmol, 88%) as a yellow oil.

^{1}H-NMR (500 MHz, $CDCl_3$) δ = 7.41 – 7.33 (m, 5H), 7.31 – 7.27 (m, 1H), 7.18 (ddd, *J* = 8.6, 7.5, 1.4 Hz, 1H), 6.64 (ddd, *J* = 7.4, 7.4, 0.9 Hz, 1H), 6.58 (d, *J* = 8.4 Hz, 1H), 5.10 (s, 1H), 4.44 (s, 2H), 3.41 (s, 1H) ppm.

^{13}C-NMR (126 MHz, $CDCl_3$) δ = 149.4, 149.4, 139.1, 139.0, 132.7, 130.5, 128.8, 127.3, 127.2, 116.5, 110.0, 106.4, 83.1, 80.9, 47.7 ppm.

HR-MS ($C_{15}H_{14}N^+$; $[M+H]^+$, pos. APCI): calcd: 208.1121, found: 208.1120.

Analytical data are in accordance with literature.[11]

3-Ethylpent-1-yn-3-ol (S12)

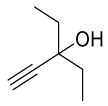

S12

Ethynylmagnesium bromide solution (0.5 M in THF, 30.0 mL, 15.0 mL, 1.50 equiv) was added to a solution of acetone pent-3-one (1.06 mL, 861 mg, 10.0 mmol, 1.00 equiv) in THF (10 mL) at 0 °C. The mixture was allowed to warm up to rt and stirred for 4 h. Aqueous HCl solution (0.5 M, 40 mL) was added, the layers were separated and the aqueous layer was extracted with Et_2O (3 × 80 mL). The combined organic extracts were dried over Na_2SO_4, the solvent was removed under reduced pressure and the residue was purified by column chromatography [SiO_2, *n*-pentane/Et_2O 4:1 v/v, R_f = 0.26 (*n*-pentane/Et_2O 4:1 v/v)] to afford the product (754 mg, 6.72 mmol, 67%) as a yellow oil.

^{1}H-NMR (400 MHz, $CDCl_3$) δ = 2.43 (s, 1H), 1.92 – 1.86 (m, 1H), 1.77 – 1.57 (m, 4H), 1.04 (t, *J* = 0.5 Hz, 6H) ppm.

^{13}**C-NMR** (101 MHz, $CDCl_3$) δ = 86.5, 72.4, 72.1, 34.3, 8.5 ppm.

HR-MS ($C_7H_{13}O^+$; $[M+H]^+$, pos. APCI): calcd: 113.0961, found: 113.0961.

This procedure is based on a previously reported one.[12]

3.3 Monosubstituted Allenes

1-Methoxy-4-((penta-3,4-dien-1-yloxy)methyl)benzene (1a)

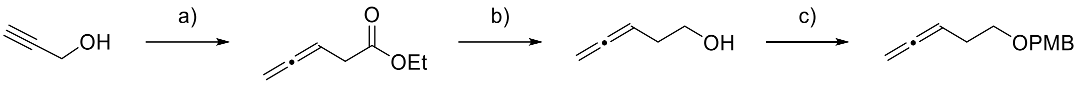

S20 **S21** **S22** **1a**

a) To a solution of propargyl alcohol (9.50 mL, 160 mmol, 0.50 equiv) in triethyl orthoacetate (60.0 mL, 320 mmol, 1.00 equiv) was added dropwise propionic acid (0.960 mL, 12.8 mmol, 4.00 mol%). The mixture was heated to 160 °C and resulting EtOH was continuously distilled off under atmospheric pressure for 1 h. Then another aliquot of propargyl alcohol (9.50 mL, 160 mmol, 0.500 equiv) and propionic acid (0.960 mL, 12.8 mmol, 4.00 mol%) were added dropwise and the mixture was stirred for another 1 h before a third portion of propionic acid (0.960 mL, 12.8mmol, 4.00 mol%) was added. The reaction was stirred for an additional hour and then cooled to rt and quenched by addition of an aqueous HCl solution (2.0 M, 20.0 mL). The aqueous layer was extracted with Et_2O (3 × 100 mL). The combined organic layers were washed with brine (100 mL), dried over Na_2SO_4 and the solvent was removed under reduced pressure. The residue was purified by fractional distillation under reduced pressure (46 mbar, 75 °C). Ethyl penta-3,4 dienoate (**S21**, 31.3 g, 248 mmol, 78%) was obtained as a colorless oil.

This procedure is based on a previously reported one. Analytical data are in accordance with literature.[13]

b) $LiAlH_4$ (2.63 g, 69.3 mmol, 1.25 equiv) was suspended in Et_2O (200 mL) at 0 °C. It was treated dropwise with ethyl penta-3,4 dienoate (7.00 g, 55.5 mmol, 1.00 eq) in Et_2O (80 mL) and then allowed to warm to rt overnight. The reaction mixture was quenched successively by the addition of H_2O (2.60 mL), aqueous NaOH solution (2.0 M, 2.6 mL) and H_2O (7.8 mL). The heterogeneous mixture was dried over Na_2SO_4, filtered over cotton wool and rinsed with Et_2O (40 mL). The solvent was removed under reduced pressure to obtain crude penta-3,4-dien-1-ol (**S22**, 4.67 g, 55.5 mmol, quant) which was used without further purification.

This procedure is based on a previously reported one. Analytical data are in accordance with literature.[14]

c) NaH (856 mg, 21.4 mmol, 1.20 equiv) was added to a solution of penta-3,4-dien-1-ol (1.80 g, 21.4 mmol, 1.20 equiv) in THF (23 mL) at 0 °C. *n*-Bu_4NI (658 mg, 17.8 mmol, 10.0 mol%) and *para*-methoxy benzyl chloride (2.40 mL, 2.79 g, 1.00 equiv) were added and the solution was allowed to warm up to rt and stirred for 15 h. The reaction was quenched with aqueous saturated NH_4Cl solution (7.0 mL) and extracted with Et_2O (3 × 10 mL). The combined organic extracts were washed with H_2O and brine, dried over Na_2SO_4 and the solvent was removed under reduced pressure. The residue was purified by flash column chromatography [SiO_2, *n*-pentane/Et_2O 20:1 v/v, R_f = 0.30 (*n*-pentane/Et_2O 20:1 v/v)] to obtain the product **1a** (2.90 g, 14.2 mmol, 80%) as a colorless oil.

This procedure is based on a previously reported one.[15]

^{1}H-NMR (400 MHz, $CDCl_3$) δ = 7.32 – 7.22 (m, 2H), 6.94 – 6.84 (m, 2H), 5.14 (p, *J* = 6.8 Hz, 1H), 4.68 (dt, *J* = 6.7, 3.1 Hz, 2H), 4.46 (d, *J* = 0.6 Hz, 2H), 3.81 (s, 3H), 3.52 (t, *J* = 6.8 Hz, 2H), 2.32 (qt, *J* = 6.8, 3.1 Hz, 2H) ppm.

^{13}C-NMR (101 MHz, $CDCl_3$) δ = 209.1, 159.4, 130.7, 129.4, 114.0, 86.9, 75.0, 72.7, 69.5, 55.4, 29.0 ppm.

HR-MS ($C_{13}H_{16}O_2^+$; $[M]^+$, pos. APCI): calcd: 204.1145, found: 204.1143.

***Tert*-butyl(penta-3,4-dien-1-yloxy)diphenylsilane (1b)**

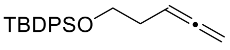

1b

To a stirred solution of allenol (0.380 mL, 336 mg, 4.00 mmol, 1.00 equiv) in CH_2Cl_2 (20 mL) was added imidazole (300 mg, 4.40 mmol, 1.10 equiv) and TBDPSCl (1.14 mL, 1.21 g, 4.40 mmol, 1.10 equiv). The mixture was stirred for 17 h at rt, then quenched with H_2O (5 mL), extracted with DCM (3 × 8 mL) and the solvent removed under reduced pressure. Purification by flash column chromatography [SiO_2, *n*-pentane/Et_2O 50:1 v/v, R_f = 0.50 (*n*-pentane/Et_2O 50:1 v/v)] gave the title compound **1b** (1.28 g, 3.96 mmol, quant) as a colorless oil.

^{1}H-NMR (500 MHz, $CDCl_3$) δ = 7.78 – 7.71 (m, 4H), 7.51 – 7.40 (m, 6H), 5.20 (p, *J* = 7.0 Hz, 1H), 4.69 (dt, *J* = 6.8, 2.9 Hz, 2H), 3.80 (t, *J* = 6.6 Hz, 2H), 2.41 – 2.27 (m, 2H), 1.12 (s, 9H) ppm.

^{13}C-NMR (126 MHz, $CDCl_3$) δ = 209.3, 135.7, 134.1, 129.7, 127.8, 86.9, 74.6, 63.6, 32.1, 27.0, 19.4 ppm.

HR-MS ($C_{21}H_{27}OSi^+$; $[M+H]^+$, pos. APCI): calcd: 323.1826, found: 323.1820.

1-Fluoro-4-(penta-3,4-dien-1-yloxy)benzene (1c)

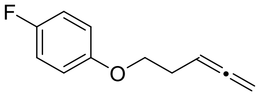

1c

Synthesis according to **GP 2** by the addition of DIAD (0.800 mL, 809 mg, 4.00 mmol. 1.00 equiv) to a solution of PPh_3 (944 mg, 3.60 mmol, 0.90 equiv), 4-fluorophenol (449 mg, 4.00 mmol, 1.00 equiv) and penta-3,4-dien-1-ol (0.450 mL, 404 mg, 4.80 mmol, 1.20 equiv) in THF (10 mL). Purification by flash column chromatography [SiO_2, *n*-pentane/Et_2O 50:1 v/v, R_f = 0.45 (*n*-pentane/Et_2O 50:1 v/v)] gave the title compound **1c** (520 mg, 2.92 mmol, 81%) as a colorless oil.

^{1}H-NMR (500 MHz, $CDCl_3$) δ = 7.04 – 6.92 (m, 2H), 6.88 – 6.80 (m, 2H), 5.21 (p, *J* = 6.8 Hz, 1H), 4.83 – 4.67 (m, 2H), 4.00 (t, *J* = 6.7 Hz, 2H), 2.47 (qt, *J* = 6.7, 3.2 Hz, 2H) ppm.

^{13}C-NMR (126 MHz, $CDCl_3$) δ = 209.2, 157.4 (d, *J* = 238 Hz), 155.2, 115.9, 115.8, 86.3, 75.5, 68.0, 28.4 ppm.

^{19}F-NMR (471 MHz, $CDCl_3$) δ = –124.1 (m_c) ppm.

HR-MS ($C_{11}H_{12}FO^+$; $[M+H]^+$, pos. APCI): calcd: 179.0867, found: 179.0868.

1-Chloro-4-(penta-3,4-dien-1-yloxy)benzene (1d)

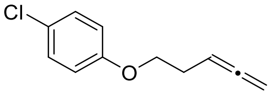

1d

Synthesis according to **GP 2** by the addition of DIAD (0.800 mL, 809 mg, 4.00 mmol. 1.00 equiv) to a solution of PPh_3 (945 mg, 3.60 mmol, 0.90 equiv), 4-chlorophenol (515 mg, 4.00 mmol, 1.00 equiv) and penta-3,4-dien-1-ol (0.450 mL, 404 mg, 4.80 mmol, 1.20 equiv) in THF (10 mL). Purification by flash column chromatography [SiO_2, *n*-pentane/Et_2O 50:1 v/v, R_f = 0.45 (*n*-pentane/Et_2O 50:1 v/v)] gave the title compound **1d** (588 mg, 3.02 mmol, 84%) as a colorless oil.

^{1}H-NMR (400 MHz, $CDCl_3$) δ = 7.25 – 7.19 (m, 2H), 6.89 – 6.76 (m, 2H), 5.21 (p, *J* = 6.8 Hz, 1H), 4.73 (dt, *J* = 6.7, 3.1 Hz, 2H), 4.00 (t, *J* = 6.7 Hz, 2H), 2.48 (dtd, *J* = 9.9, 6.7, 3.1 Hz, 2H) ppm.

^{13}C-NMR (101 MHz, $CDCl_3$) δ = 209.2, 157.7, 129.4, 125.8, 116.1, 86.2, 75.6, 67.7, 28.3 ppm.

HR-MS ($C_{11}H_{12}ClO^+$; $[M+H]^+$, pos. APCI): calcd: 195.0571, found: 195.0574.

The analytical data corresponded to the reported literature values.[16]

1-Bromo-4-(penta-3,4-dien-1-yloxy)benzene (1e)

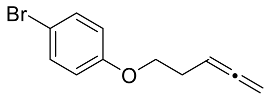

1e

Synthesis according to **GP 2** by the addition of DIAD (0.800 mL, 809 mg, 4.00 mmol. 1.00 equiv) to a solution of PPh_3 (944 mg, 3.60 mmol, 0.900 equiv), 4-bromophenol (693 mg, 4.00 mmol, 1.00 equiv) and penta-3,4-dien-1-ol (0.450 mL, 404 mg, 4.80 mmol, 1.20 equiv) in THF (10 mL). Purification by flash column chromatography [SiO_2, *n*-pentane/Et_2O 50:1 v/v, R_f = 0.45 (*n*-pentane/Et_2O 50:1 v/v)] gave the title compound **1e** (814 mg, 3.40 mmol, 94%) as a colorless oil.

^{1}H-NMR (400 MHz, $CDCl_3$) δ = 7.42 – 7.31 (m, 2H), 6.87 – 6.74 (m, 3H), 5.20 (p, *J* = 6.8 Hz, 1H), 4.72 (dt, *J* = 6.8, 3.1 Hz, 2H), 4.00 (t, *J* = 6.7 Hz, 2H), 2.48 (dtd, *J* = 9.9, 6.7, 3.1 Hz, 2H) ppm.

^{13}C-NMR (101 MHz, $CDCl_3$) δ = 209.2, 158.2, 132.4, 116.6, 113.0, 86.2, 75.6, 67.6, 28.3 ppm.

HR-MS ($C_{11}H_{12}{}^{79}BrO^+$; $[M+H]^+$, pos. APCI): calcd: 239.0066, found: 239.0066.

1-Nitro-4-(penta-3,4-dien-1-yloxy)benzene (S23)

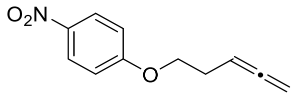

S23

Synthesis according to **GP 2** by the addition of DIAD (0.800 mL, 809 mg, 4.00 mmol, 1.00 equiv) to a solution of PPh_3 (944 mg, 3.60 mmol, 0.90 equiv), 4-nitrophenol (557 mg, 4.00 mmol, 1.00 equiv) and penta-3,4-dien-1-ol (0.450 mL, 404 mg, 4.80 mmol, 1.20 equiv) in THF (10 mL). Purification by flash column chromatography [SiO_2, *n*-pentane/Et_2O 20:1 v/v, R_f = 0.30 (*n*-pentane/Et_2O 20:1 v/v)] gave the title compound **S23** (738 mg, 3.60 mmol, quant) as a colorless oil.

^{1}H-NMR (400 MHz, $CDCl_3$) δ = 8.23 – 8.17 (m, 2H), 6.99 – 6.92 (m, 2H), 5.21 (p, *J* = 6.8 Hz, 1H), 4.74 (dt, *J* = 6.8, 3.2 Hz, 2H), 4.13 (t, *J* = 6.7 Hz, 2H), 2.58 – 2.44 (m, 2H) ppm.

^{13}C-NMR (101 MHz, $CDCl_3$) δ = 209.2, 164.1, 141.7, 126.1, 114.6, 85.8, 75.9, 68.1, 28.1 ppm.

HR-MS ($C_{11}H_{12}NO_3^+$; $[M+H]^+$, pos. APCI): calcd: 206.0812, found: 206.0816.

2-(Penta-3,4-dien-1-yloxy)benzonitrile (1f)

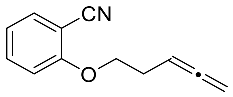

1f

Synthesis according to **GP 2** by the addition of DIAD (0.710 mL, 728 mg, 3.60 mmol. 0.90 equiv) to a solution of PPh_3 (1.05 g, 4.00 mmol, 1.00 equiv), 2-hydroxybenzonitrile (477 mg, 4.00 mmol, 1.00 equiv) and penta-3,4-dien-1-ol (0.450 mL, 404 mg, 4.80 mmol, 1.20 equiv) in THF (10 mL). Purification by flash column chromatography [SiO_2, *n*-pentane/Et_2O 9:1 v/v, R_f = 0.35 (*n*-pentane/Et_2O 9:1 v/v)] gave the title compound **1f** (631 mg, 3.41 mmol, 95%) as a yellowish oil.

^{1}H-NMR (500 MHz, $CDCl_3$) δ = 7.58 – 7.46 (m, 2H), 7.06 – 6.89 (m, 2H), 5.22 (p, *J* = 6.8 Hz, 1H), 4.73 (dt, *J* = 6.7, 3.1 Hz, 2H), 4.13 (t, *J* = 6.7 Hz, 2H), 2.53 (qt, *J* = 6.7, 3.1 Hz, 2H) ppm.

^{13}C-NMR (126 MHz, $CDCl_3$) δ = 209.2, 160.6, 134.4, 133.8, 120.8, 116.4, 112.4, 102.2, 85.7, 75.6, 68.2, 28.0 ppm.

HR-MS ($C_{12}H_{12}NO^+$; $[M+H]^+$, pos. APCI): calcd: 186.0913, found: 186.0911.

Penta-3,4-dien-1-yl 4-(*N*,*N*-dipropylsulfamoyl)benzoate (1i)

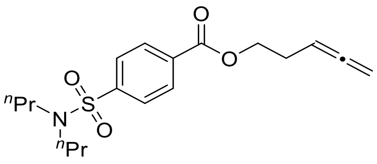

1i

Synthesis according to **GP 2** by the addition of DIAD (0.800 mL, 809 mg, 4.00 mmol. 1.00 equiv) to a solution of PPh_3 (944 mg, 3.60 mmol, 0.900 equiv), probenecid (1.14 g, 4.00 mmol, 1.00 equiv) and penta-3,4-dien-1-ol (0.450 mL, 404 mg, 4.80 mmol, 1.20 equiv) in THF (10 mL). Purification by flash column chromatography [SiO_2, *n*-pentane/Et_2O 4:1 v/v, R_f = 0.30 (*n*-pentane/Et_2O 4:1 v/v)] gave the title compound **1i** (869 mg, 2.47 mmol, 69%) as a colorless oil.

^{1}H-NMR (400 MHz, $CDCl_3$) δ = 8.17 – 8.12 (m, 2H), 7.90 – 7.84 (m, 2H), 5.17 (p, *J* = 6.8 Hz, 1H), 4.72 (dt, *J* = 6.8, 3.1 Hz, 2H), 4.42 (t, *J* = 6.6 Hz, 2H), 3.15 – 3.05 (m, 4H), 2.48 (dtd, *J* = 9.8, 6.7, 3.1 Hz, 2H), 1.63 – 1.48 (m, 4H), 0.99 – 0.63 (m, 6H) ppm.

^{13}C-NMR (101 MHz, $CDCl_3$) δ = 209.4, 165.3, 144.5, 133.8, 130.3, 127.1, 85.9, 75.7, 64.7, 50.1, 27.9, 22.1, 11.3 ppm.

HR-MS ($C_{18}H_{26}NO_4S^+$; $[M+H]^+$, pos. APCI): calcd: 352.1577, found: 352.1579.

(*R*)-2,5,7,8-Tetramethyl-6-(penta-3,4-dien-1-yloxy)-2-((4*R*,8*R*)-4,8,12-trimethyltridecyl)chromane (1j)

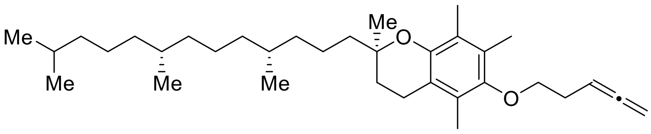

1j

Synthesis according to **GP 2** by the addition of DIAD (0.800 mL, 809 mg, 4.00 mmol. 1.00 equiv) to a solution of PPh_3 (944 mg, 3.60 mmol, 0.90 eq), *α*-Tocopherol (1.72 g, 4.00 mmol, 1.00 equiv) and penta-3,4-dien-1-ol (0.450 mL, 404 mg, 4.80 mmol, 1.20 equiv) in THF (10 mL). Purification by flash column chromatography [SiO_2, *n*-pentane/Et_2O 50:1 v/v, R_f = 0.30 (*n*-pentane/Et_2O 50:1 v/v)] gave the title compound **1j** (771 mg, 1.55 mmol, 43%) as a colorless oil.

^{1}H-NMR (400 MHz, $CDCl_3$) δ = 5.27 (p, *J* = 6.9 Hz, 1H), 4.71 (dt, *J* = 6.7, 3.1 Hz, 2H), 3.72 (t, *J* = 6.8 Hz, 2H), 2.57 (t, *J* = 6.8 Hz, 2H), 2.50 (qt, *J* = 6.9, 3.1 Hz, 2H), 2.18 (s, 3H), 2.13 (s, 3H), 2.08 (s, 3H), 1.78 (tq, *J* = 13.3, 6.5 Hz, 2H), 1.63 – 1.00 (m, 21H), 1.23 (s, 3H), 0.96 – 0.81 (m, 12H) ppm.

^{13}C-NMR (101 MHz, CDCl3) δ = 209.2, 148.4, 147.9, 128.0, 126.0, 123.0, 117.7, 86.8, 75.2, 74.9, 72.3, 40.3, 39.6, 37.7, 37.6, 37.6, 37.5, 33.0, 32.9, 31.5, 29.4, 28.1, 25.0, 24.6, 24.1, 22.9, 22.8, 21.2, 20.8, 19.9, 19.8, 13.0, 12.1, 11.9 ppm.

HR-MS ($C_{34}H_{57}O_2^+$; [M+H]$^+$, pos. APCI): calcd: 497.4353, found: 497.4352.

Penta-3,4-dien-1-ylbenzene (1k)

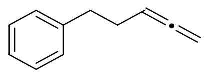

1k

Mg turnings were heated under vacuum while stirring vigorously for 5 min. Then, dry THF (20 mL) and (2-bromoethyl)benzene (2.00 mL, 2.72 g, 14.7 mmol, 0.15 equiv) were added. After the reaction had started, (2-bromoethyl)benzene (11.6 mL, 15.8 g, 85.2 mmol, 0.85 equiv) in dry THF (155 mL, 0.55 M) was added dropwise and the mixture was stirred at 80 °C for 90 min. The mixture was cooled to −78 °C and a solution of freshly distilled propargyl bromide (80% w/v in toluene, 17.9 mL, 14.3 g, 120 mmol, 1.20 equiv) in THF (50 mL, 2.4 M) was added dropwise. The resulting suspension was stirred at room temperature for 15 h. Then, saturated aqueous NH_4Cl solution (250 mL) was added, the suspension was filtered and the phases of the filtrate were separated. The aqueous phase was extracted with EtOAc (3 × 200 mL), the combined organic extracts were dried over Na_2SO_4 and the solvent was removed under reduced pressure. The residue was purified by flash column chromatography [silica gel, *n*-pentane, R_f = 0.35 (*n*-pentane)]. The target molecule **1k** (5.50 g, 38.1 mmol, 38 %) was obtained as a colorless oil.

^{1}H-NMR (400 MHz, $CDCl_3$) δ = 7.34 – 7.27 (m, 2H), 7.23 – 7.16 (m, 3H), 5.16 (ttt, *J* = 6.7, 6.6, 0.9 Hz, 1H), 4.68 (dtd, *J* = 6.6, 3.2, 1.0 Hz, 2H), 2.74 (dd, *J* = 9.0, 6.7 Hz, 2H), 2.37 – 2.29 (m, 2H) ppm.

^{13}C-NMR (101 MHz, CDCl3) δ = 208.7, 141.8, 128.6, 128.4, 126.0, 89.5, 75.2, 35.5, 30.1 ppm.

HR-MS ($C_{11}H_{12}NO^+$; [M+NO]$^+$, pos. APCI): calcd: 174.0919, found: 174.0912.

Analytical data are in accordance with literature.[17]

3.4 Geminal Disubstituted Allenes

(3-Methylpenta-3,4-dien-1-yl)benzene (S7)

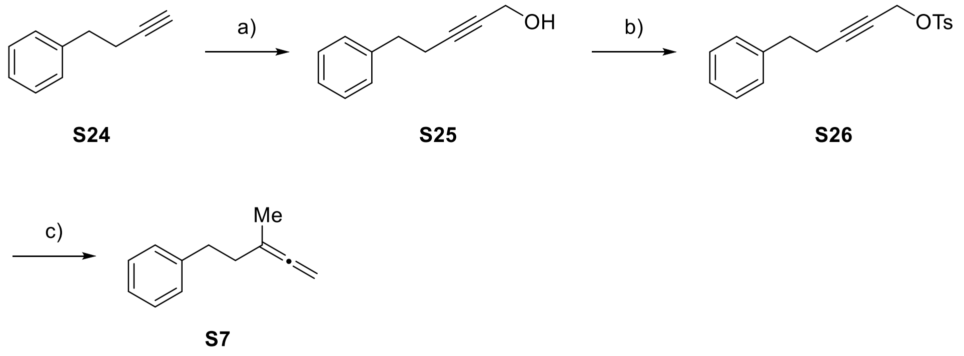

a) *n*-BuLi (2.5 M in hexane, 7.20 mL, 18.0 mmol, 1.20 equiv) was added to a solution of 4-phenyl-1-butyne (97%, 2.17 mL, 2.01 g, 15.0 mmol, 1.00 equiv) in THF (50 mL) at −60 °C and the mixture was stirred for 25 min. The resulting solution was added to a suspension of paraformaldehyde (901 mg, 30.0 mmol, 2.00 equiv) in THF (20 mL) at −50 °C. The mixture was allowed to warm up to rt and stirred for 18 h. The reaction was quenched with water (50 mL) and the solvent was removed under vacuum. The residue was extracted with Et_2O (3 × 50 mL), the combined organic extracts were washed with brine (50 mL) and dried over Na_2SO_4. The solvent was removed under reduced pressure and the residue was purified by column chromatography [SiO_2, *n*-pentane/EtOAc 4:1 v/v, R_f = 0.23 (*n*-pentane/ EtOAc 4:1 v/v)] to afford product **S25** (2.30 g, 14.3 mmol, 96%) as a yellow oil.

This procedure is based on a previously reported one. Analytical data are in accordance with literature.[18]

b) TsCl (98%, 3.35 g, 17.2 mmol, 1.20 equiv) was added to a solution of propargylic alcohol **S25** (2.30 g, 14.4 mmol, 1.00 equiv) in Et_2O (30 mL). The mixture was cooled to 0 °C and freshly pestled KOH (85%, 5.04 g, 76.3 mmol, 5.30 equiv) was added. The solution was allowed to warm up to rt and stirred for 30 min and then poured on ice. The layers were separated and the aqueous phase was extracted with Et_2O (3 × 50 mL). The combined organic extracts were washed with brine (50 mL), dried over $MgSO_4$ and the solvent was removed under reduced pressure. The crude product **S26** (4.20 g, 13.4 mmol, 93%) was used in the next step without further purification.

This procedure is based on a previously reported one. Analytical data are in accordance with literature.[18]

c) To a suspension of CuBr (98%, 196 mg, 1.34 mmol, 10.0 mol%) in THF was added tosylate **S26** (4.20 g, 13.4 mmol, 1.00 equiv) and the mixture was stirred at rt for 5 min. The reaction was cooled to 0 °C, MeMgBr (3.0 M in Et_2O, 5.60 mL, 16.8 mmol, 1.25 equiv) was added dropwise and the mixture was stirred at rt for 3 h. Then, aqueous saturated NH_4Cl solution (40 mL) was added, the layers were separated and the aqueous phase was extracted with Et_2O (3 × 40 mL). The combined organic extracts

were washed with brine (40 mL), dried over Na_2SO_4 and the solvent was removed under reduced pressure. The residue was purified by column chromatography [SiO_2, *n*-pentane, R_f = 0.49 (*n*-pentane)] to afford product **S7** (1.38 g, 8.72 mmol, 65%) as a colorless oil.

^{1}H-NMR (500 MHz, $CDCl_3$) δ = 7.31 – 7.26 (m, 2H), 7.23 – 7.16 (m, 3H), 4.62 (qt, *J* = 3.2, 3.2 Hz, 2H), 2.78 – 2.72 (m, 2H), 2.28 – 2.21 (m, 2H), 1.73 (t, *J* = 3.1 Hz, 3H) ppm.

^{13}C-NMR (126 MHz, CDCl3) δ = 206.3, 142.3, 128.5, 128.3, 125.8, 98.2, 74.7, 35.3, 34.0, 19.0 ppm.

HR-MS ($C_{12}H_{15}^+$; $[M+H]^+$, pos. APCI): calcd: 159.1168, found: 159.1167.

This procedure is based on a previously reported one. Analytical data are in accordance with literature.[19]

tert-Butyl((2,3-dimethylpenta-3,4-dien-2-yl)oxy)dimethylsilane (S11)

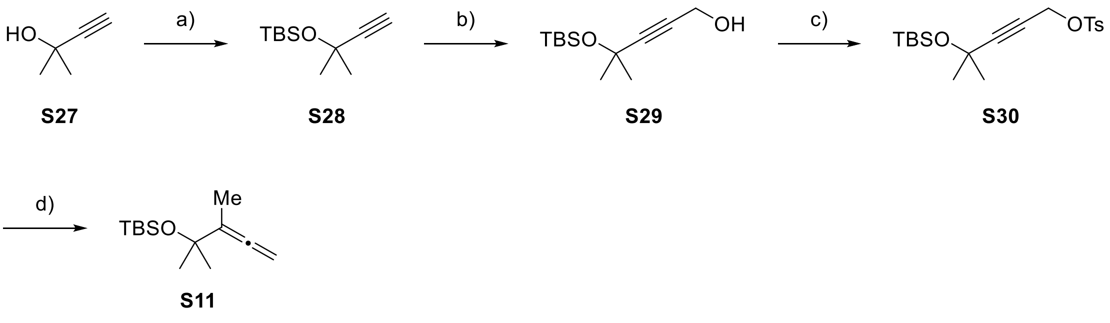

a) To a solution of alcohol **S27** in DMF (10 mL) was added TBSCl (3.00 g, 20.0 mmol, 1.00 equiv), imidazole (1.91 g, 28.0 mmol, 1.40 equiv) and DMAP (244 mg, 2.00 mmol, 10.0 mol%). The mixture was stirred at 65 °C for 19 h, cooled to rt and H_2O (40 mL) and Et_2O (40 mL) were added. The layers were separated and the aqueous phase was extracted with Et_2O (2 × 20 mL). The combined organic extracts were dried over Na_2SO_4, the solvent was removed under reduced pressure and the residue was purified by column chromatography [SiO_2, *n*-pentane, R_f = 0.63 (*n*-pentane)] to afford the product (2.65 g, 13.4 mmol, 67%) as a colorless oil.

This procedure is based on a previously reported one. Analytical data are in accordance with literature.[20]

b) *n*-BuLi (2.5 M in hexane, 6.54 mL, 16.3 mmol, 1.22 equiv) was added dropwise to a solution of alkyne **S28** (2.65 g, 13.4 mmol, 1.00 equiv) in THF at −78 °C. The mixture was allowed to warm up to 0 °C and stirred for 1 h. Then, the solution was cooled to −78 °C and paraformaldehyde (447 mg, 14.9 mmol, 1.20 equiv) was added. The mixture was allowed to warm up to rt and stirred for 19 h. Aqueous saturated NH_4Cl solution (10 mL) was added, the layers were separated and the aqueous phase was extracted with Et_2O (3 × 20 mL). The combined organic extracts were washed with brine (20 mL), dried over Na_2SO_4 and the solvent was removed under reduced pressure. The residue was purified by column

chromatography [SiO_2, *n*-pentane/Et_2O 4:1 v/v, R_f = 0.40 (*n*-pentane/Et_2O 4:1 v/v)] to afford the product (2.36 g, 10.3 mmol, 77%) as a colorless oil.

This procedure is based on a previously reported one. Analytical data are in accordance with literature.[18]

c) TsCl (98%, 2.41 g, 12.4 mmol, 1.20 equiv) was added to a solution of propargylic alcohol **S29** (2.36 g, 10.3 mmol, 1.00 equiv) in Et_2O (20 mL). The mixture was cooled to 0 °C and freshly pestled KOH (85%, 3.64 g, 55.1 mmol, 5.30 equiv) was added. The solution was allowed to warm up to rt and stirred for 30 min and then poured on ice. The layers were separated and the aqueous phase was extracted with Et_2O (3 × 20 mL). The combined organic extracts were washed with brine (20 mL), dried over Na_2SO_4 and the solvent was removed under reduced pressure. The residue was purified by column chromatography [SiO_2, *n*-pentane/CH_2Cl_2 4:1 to 1:1 v/v, R_f = 0.24 (*n*-pentane/ CH_2Cl_2 4:1 v/v)] to afford the product (3.64 g, 9.51 mmol, 92%) as a yellow oil.

This procedure is based on a previously reported one. Analytical data are in accordance with literature.[18]

d) A mixture of CuBr (58.3 mg, 261 µmol, 10.0 mol%) and tosylate **S30** (930 µL, 1.00 g, 2.61 mmol, 1.00 equiv) in THF (10 mL) was cooled to 0 °C and MeMgBr (3.0 M in Et_2O, 1.09 mL, 3.26 mmol, 1.25 equiv) was added dropwise. The solution was stirred at rt for 2 h, quenched by the addition of aqueous saturated NH_4Cl solution (20 mL) and the layers were separated. The aqueous phase was extracted with Et_2O (3 × 20 mL), the combined organic extracts were washed with brine (20 mL) and dried over Na_2SO_4. The solvent was removed under reduced pressure and the product was purified by column chromatography [SiO_2, *n*-pentane, R_f = 0.56 (*n*-pentane)] to afford product **S11** (487 mg, 2.15 mmol, 82%) as a colorless oil.

^{1}H-NMR (400 MHz, $CDCl_3$) δ = 4.63 (q, J = 3.1 Hz, 2H), 1.73 (t, J = 3.1 Hz, 3H), 1.34 (s, 6H), 0.87 (s, 9H), 0.07 (s, 6H) ppm.

^{13}C-NMR (101 MHz, $CDCl_3$) δ = 204.9, 106.5, 75.2, 73.1, 29.9, 26.0, 18.3, 14.4, −2.4 ppm.

HRMS ($C_{13}H_{26}NO_2Si$ $[M+NO]^+$, pos. APCI) m/z : calcd: 257.1750, found 257.1751.

Analytical data are in accordance with literature.[18]

tert-Butyl((4-methylhexa-4,5-dien-1-yl)oxy)diphenylsilane (S8), tert-butyl((6-methyl-4-vinylideneheptyl)oxy)diphenylsilane (S9) and tert-butyl((4-isopropylhexa-4,5-dien-1-yl)oxy)diphenylsilane (S10)

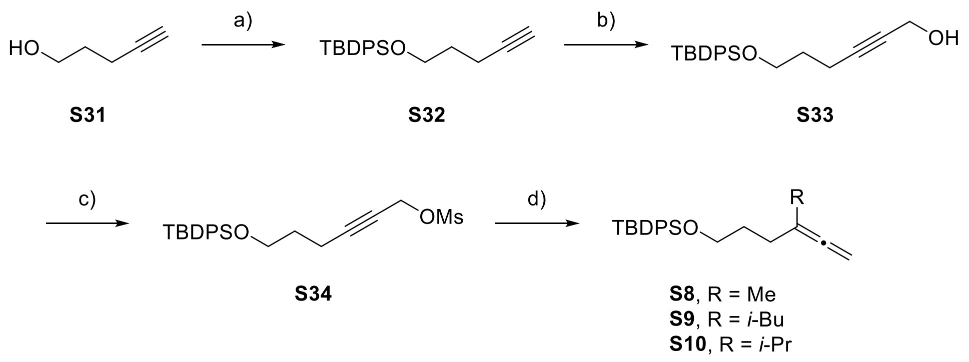

a) *tert*-Butyl(chlor)diphenylsilane (14.3 mL, 15.1 g, 55.0 mmol, 1.10 equiv) and imidazole (8.50 g, 125 mmol, 2.50 equiv) were dissolved in CH_2Cl_2 (125 mL). Pent-4-yn-1-ol (4.65 mL, 4.21 g, 100 mmol, 1.00 equiv) was added. The reaction mixture was stirred at rt for 3 h. Afterwards, it was quenched with water (200 mL). The phases were separated, and the aqueous layer was further extracted with CH_2Cl_2 (2 × 150 mL). The combined organic phases were washed with water, dried over Na_2SO_4, filtered and concentrated by rotary evaporation. The crude residue was purified by flash column chromatography chromatography [silica gel, *n*-pentane, R_f = 0.29 (*n*-pentane)] to obtain the title compound (14.8 g, 46.0 mmol, 91%) as a colorless oil.

Analytical data is in accordance with literature.[21]

b) *tert*-Butyl(pent-4-yn-1yloxy)diphenylsilane (14.8 g, 46.0 mmol, 1.00 equiv) was dissolved in THF (185 mL) and the solution was cooled to −78 °C. *n*-BuLi (2.5 M in *n*-hexane, 22.0 mL, 55.1 mmol, 1.20 equiv) was added dropwise. The reaction mixture was stirred at −78 °C for 1 h until it was transferred to a suspension of $(CH_2O)_n$ (3.45 g, 115 mmol, 2.50 equiv) in THF (110 mL) at 0 °C. The reaction mixture was warmed to rt and stirred overnight. A saturated aqueous solution of NH_4Cl (300 mL) was added and the aqueous layer was extracted with EtOAc (3 × 185 mL). The combined organic layers were washed with brine (185 mL), dried over Na_2SO_4, filtered and concentrated in vacuo. The crude residue was purified by flash column chromatography [SiO_2, *n*-pentane/EtOAc 5:1 v/v, R_f = 0.40 (*n*-pentane/EtOAc 5:1 v/v)] to obtain the title compound (13.3 g, 37.7 mmol, 81%) as a colorless oil.

Analytical data is in accordance with literature.[21]

c) 6-((*tert*-Butyldiphenylsilyl)oxy)hex-2-yn-1-ol (13.4 g, 37.7 mmol, 1.00 equiv) was dissolved in THF (380 mL) and the solution was cooled to 0 °C. NEt_3 (5.78 mL, 41.5 mmol, 1.10 equiv) and methanesulfonylchloride (3.21 mL, 41.5 mmol, 1.10 equiv) were added. The reaction mixture was stirred for 30 min at 0 °C. Afterwards the solvent was removed by rotary evaporation and the residue was

purified by column chromatography [SiO_2, *n*-pentane/ EtOAc 19:1 to 4/1 v/v, R_f = 0.24 (*n*-pentane/EtOAc 4:1 v/v)] to obtain the title compound (5.87 g, 13.6 mmol, 36%) as a colorless oil.

^{1}H-NMR (700 MHz, $CDCl_3$) *δ* = 7.67 – 7.65 (m, 5H), 7.41 – 7.38 (m, 6H), 4.81 (t, *J* = 2.2 Hz, 2H), 3.73 (t, *J* = 5.9 Hz, 2H), 3.04 (s, 2H), 2.42 (tt, *J* = 7.1, 2.3 Hz, 2H), 1.77 (ddd, *J* = 13.0, 7.1, 5.9 Hz, 2H), 1.06 (s, 9H) ppm.

^{13}C-NMR (176 MHz, $CDCl_3$) *δ* = 135.6, 133.8, 129.8, 127.8, 90.6, 62.2, 58.5, 39.0, 31.1, 26.9, 21.1, 19.3, 15.4, 14.3 ppm.

HRMS ($C_{23}H_{31}O_4SSi^+$, $[M+H]^+$, pos. ESI): m/z:cald: 431.1707, found 431.1700.

d_1) *tert*-Butyl((4-methylhexa-4,5-dien-1-yl)oxy)diphenylsilane (**S34**) was synthesized according to **GP 3**. The crude product was purified by flash column chromatography [SiO_2, *n*-pentane/ EtOAc 9:1 v/v, R_f = 0.40 (*n*-pentane/EtOAc 9:1 v/v)] to obtain the title compound **S8** (0.723 g, 2.06 mmol, 82%) as a viscous oil.

^{1}H-NMR (700 MHz, $CDCl_3$) δ = 7.69 – 7.66 (m, 4H), 7.44 – 7.41 (m, 2H), 7.39 (dd, *J* = 7.1, 1.2 Hz, 4H), 4.55 (h, *J* = 3.2 Hz, 2H), 3.69 (t, *J* = 6.3 Hz, 2H), 2.05 – 2.01 (m, 2H), 1.73 – 1.66 (m, 5H), 1.05 (s, 9H) ppm.

^{13}C-NMR (176 MHz, $CDCl_3$) δ = 206.1, 135.7, 134.2, 129.6, 127.7, 98.2, 74.3, 63.5, 30.5, 29.7, 27.0, 19.3, 19.0 ppm.

D_2) *tert*-Butyl((6-methyl-4-vinylideneheptyl)oxy)diphenylsilane (**S34**) was synthesized according to **GP 3**. The crude product was purified by flash column chromatography [SiO_2, *n*-pentane/ EtOAc 9:1 v/v, R_f = 0.69 (*n*-pentane/EtOAc 9:1 v/v)] to obtain the title compound **S9** (0.761 g, 1.94 mmol, 77%) as a colorless oil.

^{1}H-NMR (700 MHz, $CDCl_3$) δ = 7.67 (ddd, *J* = 8.0, 2.7, 1.4 Hz, 4H), 7.44 – 7.38 (m, 2H), 7.38 (ddt, *J* = 7.5, 6.7, 0.8 Hz, 5H), 4.61 – 4.57 (m, 2H), 3.71 – 3.67 (m, 2H), 2.00 (ddt, *J* = 10.5, 5.7, 2.9 Hz, 2H), 1.83 – 1.66 (m, 6H), 1.05 (s, 9H), 0.90 (dd, *J* = 6.6, 1.4 Hz, 6H) ppm.

^{13}C-NMR (176 MHz, $CDCl_3$) δ = 206.3, 135.7, 134.2, 129.6, 127.7, 101.8, 75.1, 63.6, 42.2, 30.6, 27.0, 26.6, 22.7, 19.3 ppm.

D_3) *tert*-Butyl((4-isopropylhexa-4,5-dien-1-yl)oxy)diphenylsilane (**S34**) was synthesized according to **GP 3**. The crude product was purified by flash column chromatography [SiO_2, *n*-pentane/ EtOAc 9:1 v/v, R_f = 0.60 (*n*-pentane/EtOAc 9:1 v/v)] to obtain the title compound **S10** (0.761 g, 2.01 mmol, 80%) as a slightly yellow oil.

^{1}H-NMR (700 MHz, $CDCl_3$) δ = 7.70 – 7.65 (m, 5H), 7.45 – 7.39 (m, 2H), 7.42 – 7.35 (m, 5H), 4.65 (td, *J* = 3.5, 2.6 Hz, 2H), 3.70 (t, *J* = 6.3 Hz, 2H), 2.12 – 2.01 (m, 4H), 1.74 – 1.68 (m, 2H), 1.06 (s, 9H), 1.02 (d, *J* = 6.8 Hz, 6H) ppm.

^{13}C-NMR (176 MHz, $CDCl_3$) δ = 204.7, 134.2, 129.6, 127.7, 109.5, 77.0, 63.6, 30.8, 30.6, 27.0, 26.4, 21.7, 19.3 ppm.

HRMS ($C_{25}H_{36}OSi$ $[M+H]^+$, pos. ESI) m/z : cald : 379.2452, found 379.2455.

3.5 Ligands

(2*R*,3*R*)-1,4-Bis(diphenylphosphaneyl)butane-2,3-diol (L20)

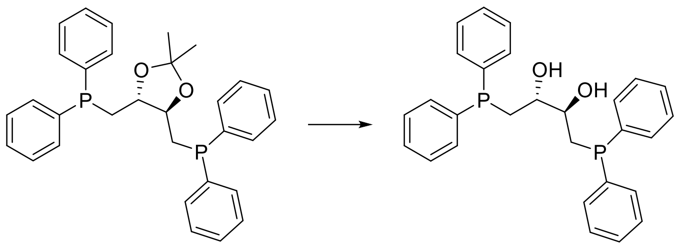

L13 **L20**

A solution of (*R*,*R*)-DIOP (100 mg, 0.200 mmol, 1.00 equiv) and *para*-toluenesulfonic acid monohydrate (7.60 mg, 40.0 µmol, 20.0 mol%) in a degassed EtOH/H_2O mixture (95:5, 2.00 mL) was heated to reflux overnight. The reaction mixture was cooled to rt, the solvent removed under vacuum and the residue filtered through a plug of silica gel (DCM:EtOAc 9:1 (degassed), 150 mL). After removal of the solvent, the ligand **L20** (65.1 mg, 0.140 mmol, 71%) was obtained as a colorless solid.

^{1}H-NMR (700 MHz, C_6D_6) δ = 7.43 – 7.37 (m, 8H), 7.08 – 6.97 (m, 12H), 3.84 – 3.75 (m, 2H), 2.40 – 2.35 (m, 2H), 2.32 – 2.27 (m, 2H) ppm.

^{13}C-NMR (176 MHz, C_6D_6) δ = 139.5 (d, *J* = 13.0 Hz), 139.1 (d, *J* = 13.0 Hz), 133.4 (d, *J* = 19.8 Hz), 133.1 (d, *J* = 19.1 Hz), 128.8, 128.7 (d, *J* = 7.0 Hz), 128.7 (d, *J* = 6.7 Hz), 128.6, 72.4 (dd, *J* = 14.9, 8.0 Hz), 34.2 (d, *J* = 13.7 Hz) ppm.

^{31}P-NMR (283 MHz, C_6D_6): δ = –22.5 ppm.

HR-MS ($C_{28}H_{29}O_2P_2^+$; $[M+H]^+$, pos. ESI): calcd: 459.1637, found: 459.1637.

The analytical data corresponded to the reported literature values.[22]

(2*R*,3*R*)-2,3-Bis((diphenylphosphaneyl)methyl)-1,4-dioxaspiro[4.4]nonane (L17)

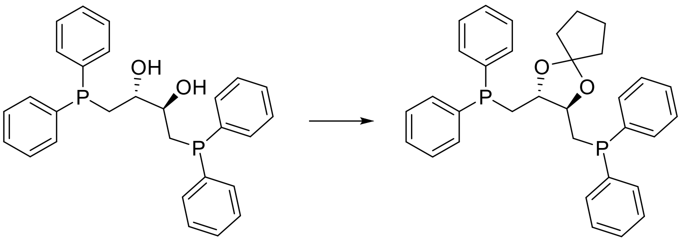

L20 **L17**

A solution of (*R*,*R*)-DIOP-Diol (79.4 mg, 0.17 mmol, 1.00 equiv), cyclopentanone (37.6 µL, 35.6 mg, 0.43 mmol, 2.50 equiv) and *para*-toluenesulfonic acid monohydrate (6.50 mg, 30.0 µmol, 20.0 mol%) in toluene (1.0 mL) was heated to reflux overnight in a SCHLENK tube charged with 4 Å molecular sieve. The reaction mixture was cooled to rt, washed with H_2O (1.0 mL), dried over Na_2SO_4 and the solvent removed under vacuum. After purification by flash column chromatography [SiO_2, cyclohexane/EtOAc 10:1 v/v, R_f = 0.30 (cyclohexane/EtOAc 10:1 v/v)] the ligand **L17** (50.8 mg, 0.100 mmol, 57%) was obtained as a colorless solid.

^{1}H-NMR (500 MHz, C_6D_6) δ = 7.62 – 7.50 (m, 8H), 7.22 – 7.09 (m, 12H), 4.28 – 4.18 (m, 2H), 2.58 – 2.45 (m, 4H), 2.02 – 1.86 (m, 4H), 1.66 – 1.54 (m, 4H) ppm.

^{13}C-NMR (101 MHz, C_6D_6) δ = 139.5 (t, *J* = 14 Hz), 133.28 (dd, *J* = 20, 16 Hz), 129.0 – 128.4 (m), 119.0, 80.3 (m_c), 33.1 (dd, *J* = 17, 3 Hz), 23.7 ppm.

^{31}P-NMR (122 MHz, C_6D_6): δ = –22.5 ppm.

HR-MS ($C_{33}H_{35}O_4P_2^+$; $[M+O_2+H]^+$, pos. ESI): calcd: 557.2005, found: 557.2007.

The analytical data corresponded to the reported literature values.[23]

(2*R*,3*R*)-2,3-Bis((diphenylphosphaneyl)methyl)-1,4-dioxaspiro[4.6]undecane (L19)

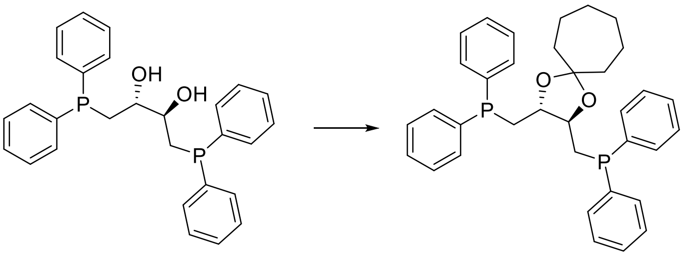

L20 **L19**

A solution of (*R*,*R*)-DIOP-Diol (91.7 mg, 0.200 mmol, 1.00 equiv), cycloheptanone (59.0 µL, 56.1 mg, 500 µmol, 2.50 equiv) and *para*-toluenesulfonic acid monohydrate (7.60 mg, 40.0 µmol, 20.0 mol%) in toluene (1.0 mL) was heated to reflux overnight in a Schlenk tube charged with 4 Å molecular sieve. The reaction mixture was cooled to rt, washed with H_2O (1.0 mL), dried over Na_2SO_4 and the solvent removed under vacuum. After purification by flash column chromatography [SiO_2, cyclohexane/EtOAc

10:1 v/v, R_f = 0.40 (cyclohexane/EtOAc 10:1 v/v)] the ligand **L19** (49.7 mg, 90.0 µmol, 44%) was obtained as a colorless solid.

^{1}H-NMR (500 MHz, C_6D_6) δ = 7.50 – 7.40 (m, 8H), 7.10 – 7.01 (m, 12H), 4.20 – 4.03 (m, 2H), 2.53 – 2.22 (m, 4H), 2.03 – 1.68 (m, 4H), 1.48 – 1.43 (m, 6H), 1.42 – 1.24 (m, 2H) ppm.

^{13}C-NMR (126 MHz, C_6D_6) δ = 139.7 (dd, *J* = 14, 3 Hz), 133.30 (dd, *J* = 20, 5 Hz), 128.8 – 128.5 (m), 113.1, 79.6 (m_c), 40.4, 32.44 (dd, *J* = 17, 4 Hz), 28.8, 22.4 ppm.

^{31}P-NMR (122 MHz, $CDCl_3$): δ = –22.2 ppm.

HR-MS ($C_{35}H_{39}O_4P_2^+$; $[M+O_2+H]^+$, pos. ESI): calcd: 585.2318, found: 585.2318.

The analytical data corresponded to the reported literature values.[23]

((2*S*,3*S*)-1,4-Dioxaspiro[4.5]decane-2,3-diyl)bis(methylene) bis(4-methylbenzenesulfonate) (S38)

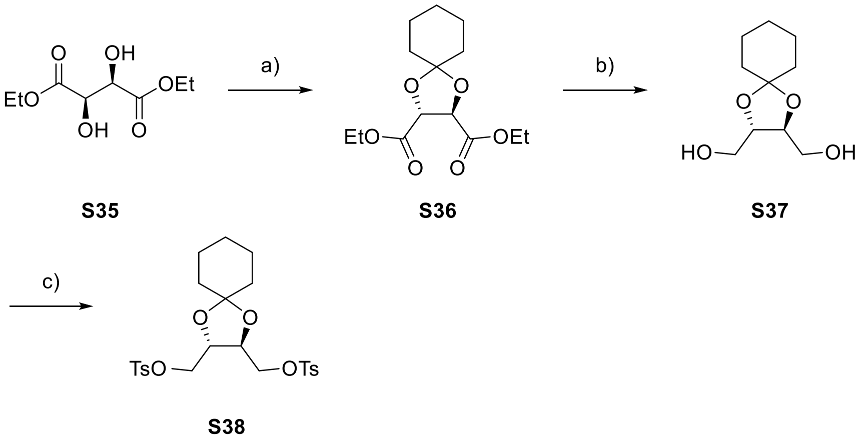

a) Cyclohexanone (7.75 mL, 7.36 g, 75.0 mmol, 1.50 equiv) was added to a solution of diethyl L-tartrate (98%, 9.05 mL, 10.9 g, 50.0 mmol, 1.0 equiv), $ZnCl_2$ (293 mg, 2.00 mmol, 4.00 mol%) and *p*-TsOH × H_2O (285 mg, 1.50 mmol, 3.00 mol%) in toluene (185 mL) and the reaction mixture refluxed overnight with DEAN-STARK removal of H_2O. After cooling to room temperature, $NaHCO_3$ (1.24 g) was added and the mixture was stirred for 15 min. Water (75 mL) was added and the organic layer was separated. The aqueous layer was extracted with EtOAc (2 × 90 mL). The combined organic extracts were washed with water (90 mL), brine (90 mL), dried over Na_2SO_4 and concentrated to give the product **S36** (12.7 g, 44.2 mmol, 88%) as a yellow oil. The crude product obtained was used for subsequent reaction without further purification.

This procedure is based on a previously reported one.[24]

b) A suspension of $LiAlH_4$ (1.90 g, 50.0 mmol, 2.50 equiv) in THF (45 mL) was cooled to 0 °C and a solution of **S36** (5.45 mL, 5.73 g, 20.0 mmol, 1.00 equiv) in THF (45 mL) was slowly added and subsequently heated to reflux for 2 h. H_2O (4.0 mL), an aqueous NaOH solution (6.0 M, 4.0 mL) and H_2O (4.0 mL) were added to the mixture at 0 °C. After stirring for 30 min, the white suspension was

filtered over a pad of silica and eluted with Et_2O. The filtrate was dried over Na_2SO_4 and concentrated under reduced pressure. The product (3.43 g, 17.0 mmol, 85%) was obtained as a colorless oil and used in the next step without further purification.

c) To a stirred solution of **S37** (1.61 g, 7.96 mmol, 1.00 equiv) in Et_2O (15 mL) was added *p*-TsCl (98%, 3.64 g, 19.1 mmol, 2.40 equiv) and the reaction mixture was cooled to 0 °C. Then, crushed KOH (85%, 3.15 g, 47.8 mmol, 6.00 equiv) was added in small portions and stirred for 30 min at 0 °C. The reaction mixture was poured into ice water and transferred to a separatory funnel. After the extraction with Et_2O (3 × 15 mL), the combined organic extracts were washed with brine (15 mL) and dried over Na_2SO_4. The solvent was removed under reduced pressure and the residue was purified by column chromatography [SiO_2, *n*-pentane/EtOAc 4:1 to 3:2 v/v, R_f = 0.25 (*n*-pentane/EtOAc 4:1 v/v)] to afford the product **S38** (1.72 g, 3.38 mmol, 42%) as a colorless solid.

This procedure is based on a previously reported one.[25]

^{1}H-NMR (700 MHz, $CDCl_3$) δ = 7.78 (d, J = 8.3 Hz, 4H), 7.38 – 7.34 (m, 4H), 4.10 – 4.06 (m, 4H), 4.02 – 3.99 (m, 2H), 2.46 (s, 6H), 1.54 – 1.44 (m, 8H), 1.38 – 1.30 (m, 2H) ppm.

^{13}C-NMR (176 MHz, $CDCl_3$) δ = 145.3, 132.6, 130.1, 128.1, 111.6, 74.8, 68.8, 36.4, 24.9, 23.7, 21.8 ppm.

HR-MS ($C_{24}H_{30}O_8NaS_2^+$; $[M+Na]^+$, pos. ESI): calcd: 533.1274, found: 533.1272.

The corresponding (*R*,*R*)-enantiomer of **S38** was obtained analogously starting from diethyl D-tartrate.

((4*R*,5*R*)-2,2-Dimethyl-1,3-dioxolane-4,5-diyl)bis(methylene) bis(4-methylbenzenesulfonate) (S43)

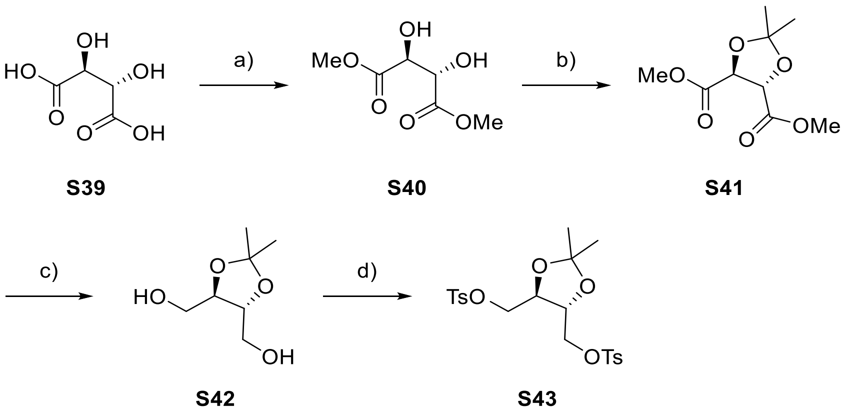

a) Thionyl chloride (48.3 mL, 665 mmol, 5.00 equiv) was added slowly to a solution of D-tartaric acid (19.1 g, 127 mmol, 1.00 equiv) in methanol (60 mL) at 0 °C using a gas wash bottle charged with a saturated aqueous solution of NaOH to neutralize the resulting HCl. The reaction mixture was allowed to warm to rt, stirred for 20 min and then heated to reflux for 3 hours. The mixture was slowly quenched

with a saturated aqueous Na_2CO_3 solution (50 mL) and methanol was removed under vacuum. The mixture was extracted with EtOAc (3 × 80 mL), dried over Na_2SO_4 and the solvent was removed under vacuum. The product (16.3 g, 91.3 mmol, 72%) was obtained as a colorless solid. The crude was used for the next synthetic step without further purification.

b) To a solution of dimethyl D-tartrate (16.0 g, 89.8 mmol, 1.00 equiv) and camphorsulfonic acid (10.4 g, 44.9 mmol, 0.50 equiv) in acetone (112 mL) was added 2,2-dimethoxypropane (24.2 mL, 20.6 g, 198 mmol, 2.20 equiv) at rt and stirred for 20 h. The reaction mixture was diluted with a saturated aqueous solution of $NaHCO_3$ (112 mL). The precipitate was filtered off, the filtrate diluted with H_2O (50 mL) and the aqueous layer was extracted with EtOAc (3 × 100 mL). The combined organic layers were dried over Na_2SO_4 and concentrated under reduced pressure. The residue was purified by fractional distillation (6.6×10^{-1} mbar, 72 °C) to give a yellowish oil (8.00 g, 36.7 mmol, 41%).

c) A suspension of $LiAlH_4$ (3.48 g, 91.8 mmol, 2.50 equiv) in THF (80 mL) was cooled to 0 °C and a solution of (4*S*,5*S*)-2,2-dimethyl-1,3-dioxolan-4,5-dimethanol (8.00 g, 36.7 mmol, 1.00 equiv) in THF (80 mL) was slowly added and subsequently heated to reflux for 4 h. H_2O (7.0 mL) and an aqueous solution of NaOH (6.00 M, 7.0 mL) were added to the mixture at 0 °C. The white suspension was filtered over a pad of silica and eluted with Et_2O. The filtrate was dried over Na_2SO_4 and concentrated under reduced pressure. The product (5.53 g, 34.1 mmol, 93%) was obtained as a colorless oil.

d) Tosyl chloride (16.9 g, 88.7 mmol, 3.10 equiv) was added to a solution of **S42** (4.60 g, 28.6 mmol, 1.00 equiv) in pyridine (36.0 mL) at −18 °C. The reaction mixture was allowed to come to rt and stirred for 20 h. H_2O (300 mL) was added and the mixture was stored at 0 °C to complete crystallization. The product was collected by filtration, washed with H_2O and EtOH and was finally dried in vacuo. The title compound **S43** (7.50 g, 15.9 mmol, 56%) was obtained as colorless solid.

^{1}H-NMR (400 MHz, $CDCl_3$) δ = 7.83 – 7.75 (m, 4H), 7.40 – 7.34 (m, 4H), 4.12 – 4.06 (m, 4H), 4.06 – 4.01 (td, *J* = 2.8, 1.5 Hz, 2H), 2.46 (d, *J* = 0.8 Hz, 6H), 1.30 (s, 6H) ppm.

^{13}C-NMR (101 MHz, $CDCl_3$) δ = 145.4, 132.7, 130.1, 128.2, 111.0, 75.3, 68.6, 26.9, 21.8 ppm.

HR-MS ($C_{21}H_{27}O_8S_2^+$; $[M+H]^+$, pos. APCI): calcd: 471.1142, found: 471.1140.

The analytical data corresponded to the reported literature values.[23]

The corresponding (*S*,*S*)-enantiomer of **S-43** was obtained analogously starting from L-tartaric acid.

(((4*S*,5*S*)-2,2-Dimethyl-1,3-dioxolane-4,5-diyl)bis(methylene))bis(bis(3,5-bis(trifluoromethyl)phenyl)phosphane) (L22)

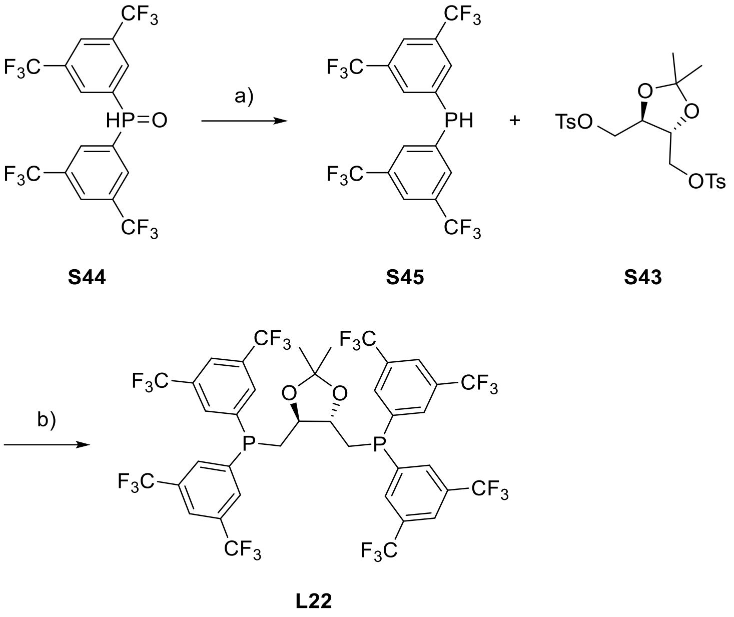

a) To a solution of DIBAL-H in heptane (1.00 M, 14.3 mL, 9.00 mmol, 3.00 equiv) was added **S44** (1.42 g, 4.61 mmol, 1.00 equiv) in THF (11.0 mL) over a period of 15 min. The mixture was stirred for 30 min at rt, MTBE (20.0 mL, degassed) was added over 10 min. After cooling the solution to 0 °C, an aqueous NaOH solution (2.00 M, 11.0 mL) was added over 15 min, followed by saturated aqueous NaCl solution (6.0 mL) over 5 min. The solution was stirred for additional 5 min and warmed to rt. Stirring was subsequently stopped and the layers allowed to separate. The organic layer was then transferred via cannula to a second 100 mL flask charged with Na_2SO_4. After stirring for 10 min, the mixture was filtered and the solvent removed in vacuo to obtain the phosphine (1.48 g, 3.23 mmol, 70%) as a colorless oil.

b) At –78 °C, **S45** (332 mg, 2.10 mmol, 2.10 equiv) in THF (1.75 mL) was added to NaH (88.0 mg, 2.20 mmol, 2.20 equiv) over a period of 10 min. The solution was stirred at –78 °C for an additional 15 min and then allowed to come to rt. After stirring at rt for 10 min, the reaction mixture was cooled to –78 °C and a solution of **S43** (471 mg, 1.00 mmol, 1.00 equiv) in THF (3.0 mL) was slowly added. The resulting mixture was allowed to come to rt and stirred for an additional 12 h. THF was evaporated under reduced pressure and the residue was dissolved in degassed Et_2O (10 mL). The resulting solution was washed with degassed water (3 × 5 mL). The organic layer was separated, dried with Na_2SO_4 and the solvent was evaporated under reduced pressure to give a light-yellow solid. After purification by flash column chromatography [SiO_2, *n*-pentane/CH_2Cl_2 (degassed) 5:1 to 2:1 v/v, R_f = 0.30 (*n*-pentane/CH_2Cl_2 (degassed) 2:1 v/v)] the ligand **L22** (260 mg, 250 µmol, 25%) was obtained as a colorless solid.

^{1}H-NMR (500 MHz, C_6D_6) δ = 7.80 (s, 2H), 7.79 (s, 2H), 7.76 (s, 2H), 7.75 (s, 1H), 7.65 (s, 4H), 3.78 – 3.66 (m, 2H), 1.82 – 1.64 (m, 4H), 1.12 (s, 6H) ppm.

13C-NMR (126 MHz, C_6D_6) δ = 141.06 (dd, J = 20, 13 Hz), 132.4, 132.1 (dd, J = 28, 6 Hz), 128.4 (m_c), 123.7 – 123.2 (m), 109.8, 79.3 (m_c), 30.60 (d, J = 17 Hz), 26.6 ppm.

19F-NMR (471 MHz, C_6D_6): δ = –62.8 (s), –62.8 (s) ppm.

31P-NMR (203 MHz, C_6D_6): δ = –16.5 ppm.

HR-MS ($C_{39}H_{25}F_{24}O_4P_2$; $[M+O_2+H]^+$, pos. ESI): calcd: 1075.0844, found: 1075.0839.

The analytical data corresponded to the reported literature values.[23]

Bis(4-methoxy-3,5-dimethylphenyl)phosphine oxide (S49)

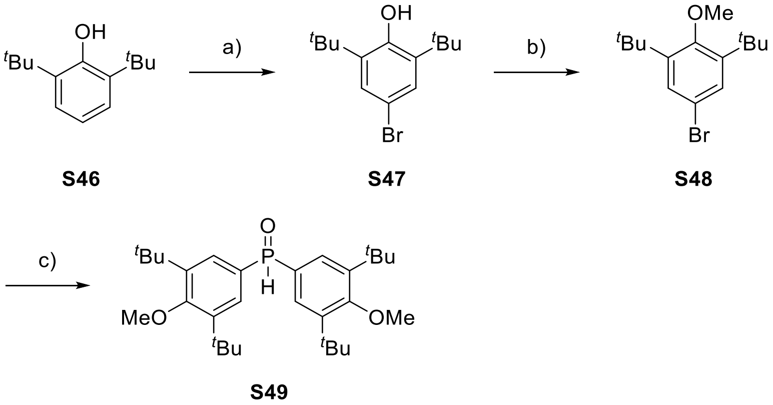

a) *N*-bromosuccinimide (19.6 g, 110 mmol, 1.10 equiv) was added to a solution of 2,6-di-*tert*-butylphenol in MeCN (200 mL). The mixture was heated to reflux overnight. The solvent was evaporated under reduced pressure and the residue solved in EtOAc (500 mL). The organic solution was washed with H_2O (3 × 250 mL) and brine (500 mL). The combined organic layers were dried over Na_2SO_4 and concentrated under reduced pressure. The residue was purified by column chromatography [SiO_2, 100% *n*-pentane, R_f = 0.41 (100% *n*-pentane)] to afford the product (26.9 g, 94.3 mmol, 94%) as a yellow oil.

b) NaH (60% in mineral oil, 5.66 g, 141 mmol, 1.50 equiv) was suspended in THF (400 mL) and cooled down to 0 °C. A solution of 4-bromo-2,6-di-*tert*-butyl phenol (26.9 g, 94.3 mmol, 1.00 equiv) in THF (100 mL) was slowly added. After complete addition the reaction mixture was allowed to come to rt and stirred for 1 h. The mixture was cooled down to 0 °C and dimethyl sulfate (9.77 mL, 13.0 g, 103 mmol, 1.10 equiv) was added. The reaction mixture was heated to reflux overnight. Then, H_2O (70 mL) was carefully added at 0 °C and the mixture was allowed to come to rt. Additional H_2O (100 mL) and Et_2O (400 mL) were added and the organic layer was extracted with Et_2O (2 × 400 mL). The combined organic extracts were washed with H_2O (2 × 150 mL) and brine (150 mL). The solution was dried over Na_2SO_4 and concentrated under reduced pressure. The residue was purified by column chromatography [SiO_2, 100% *n*-pentane, R_f = 0.52 (100% *n*-pentane)] to afford the product (27.6 g, 92.0 mmol, 98%) as a colorless solid.

c) 5-Bromo-1,3-di-*tert*-butyl-2-methoxybenzene (2.00 g, 6.68 mmol, 15 mol%) was added to a stirred suspension of magnesium (4.55 g, 187 mmol, 4.24 equiv) in THF (20 mL). With starting of the reaction, a solution of remaining **S48** (24.4 g, 88.2 mmol, 2.00 equiv) in THF (80 mL) was added quickly to the reaction mixture. The reaction mixture was heated to reflux over 4 h. In the meanwhile, diethyl phosphite (98%, 5.80 mL, 6.21 g, 44.1 mmol, 1.00 equiv) was carefully added to a suspension of NaH (60% in mineral oil, 1.94 g, 48.5 mmol, 1.10 equiv) in THF (68 mL) and heated to reflux. The solution of the deprotonated diethyl phosphite was then slowly added at rt to the Grignard suspension and the reaction mixture heated to reflux for 2 h, allowed to come to RT and stirred overnight. Then, H_2O (60 mL) and aqueous HCl solution (2.0 M, 200 mL) were added. The mixture was filtered and the aqueous layer was extracted with CH_2Cl_2 (3 × 150 mL). The combined organic extracts were washed with brine (2 × 150 mL), dried over Na_2SO_4 and the solvent was removed under reduced pressure. The residue was purified by column chromatography [SiO_2, CH_2Cl_2/EtOAc 19:1 to 4/1 v/v, R_f = 0.50 (CH_2Cl_2/EtOAc 9:1 v/v)] to afford the product **S49** (12.3 g, 25.3 mmol, 57%) as a colorless solid.

^{1}H-NMR (500 MHz, C_6D_6) δ = 7.88 (d, J = 14.0 Hz, 4H), 7.23 (d, J = 7.8 Hz, 1H), 3.27 (s, 6H), 1.34 (s, 36H) ppm.

^{13}C-NMR (126 MHz, C_6D_6) δ = 163.3 (d, J = 3 Hz), 144.9 (d, J = 13 Hz), 129.6 (d, J = 13 Hz), 126.9, 64.2, 36.1, 31.9 ppm.

^{31}P-NMR (203 MHz, C_6D_6): δ = 17.7 ppm.

HR-MS ($C_{30}H_{48}O_3P^+$; [M+H]$^+$, pos. ESI): calcd: 487.3336, found: 487.3333.

The analytical data corresponded to the reported literature values.[23]

(2*R*,3*R*)-2,3-Bis((bis(3,5-di-tert-butyl-4-methoxyphenyl)phosphaneyl)methyl)-1,4-dioxaspiro[4.5]decane (L25)

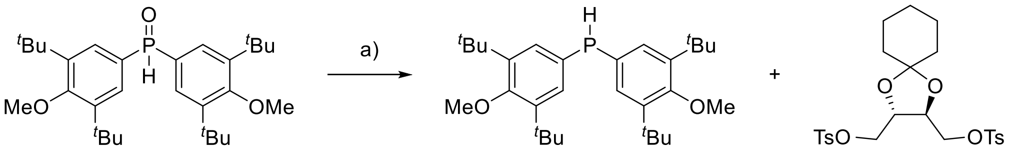

S49 **S50** **S38**

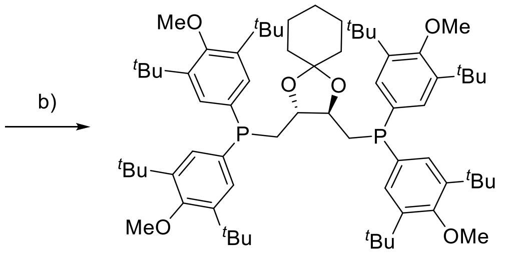

L25

a) To a suspension of $LiAlH_4$ (460 mg, 12.1 mmol, 3.40 equiv) in Et_2O (15 mL) was slowly added **S49** (1.74 g, 3.56 mmol, 1.00 equiv) in Et_2O (20 mL) at 0 °C. The reaction mixture was heated to reflux

overnight. After the suspension was cooled down to 0 °C, degassed H_2O (3.0 mL) was slowly added and stirred at rt for 20 min. Using a SCHLENK frit, the reaction mixture was filtered through a pad of silica and the resulting clear solution was concentrated under reduced pressure to give a white solid (83% purity). Due to its ability to rapid oxidation, the compound was readily used in the next step.

b) Phosphine **S50** (1.68 g, 3.56 mmol, 2.10 equiv) was dissolved in THF (15 mL) cooled down to –78 °C and *n*-BuLi (2.5 M in hexane, 2.24 mL, 5.59 mmol, 3.30 equiv) was carefully added. The reaction mixture was allowed to come to rt and stirred for 2 h. Tosylate **S38** (868 mg, 1.70 mmol, 1.00 equiv) in THF (5 mL) was added and the mixture was stirred at rt overnight. MeOH (10 mL) and H_2O (10 mL) were added and the reaction mixture was concentrated under reduced pressure. The residue was extracted with CH_2Cl_2 (3 × 20 mL), the combined organic extracts were washed with brine (20 mL), dried over Na_2SO_4 and concentrated under reduced pressure. The crude product was purified via flash column chromatography [SiO_2, *n*-pentane/CH_2Cl_2 7:3 to 2/3 v/v, R_f = 0.62 (*n*-pentane/CH_2Cl_2 3:2 v/v)] to afford ligand **L25** (149 mg, 134 µmol, 8%) as a colorless solid.

^{1}H-NMR (400 MHz, C_6D_6) δ = 7.76 – 7.69 (m, 8H), 4.44 – 4.33 (m, 2H), 3.39 (s, 6H), 3.36 (s, 6H), 2.83 – 2.70 (m, 4H), 1.63 (t, *J* = 6.1 Hz, 4H), 1.53 – 1.25 (m, 76H), 1.21 – 1.11 (m, 2H) ppm.

^{13}C-NMR (126 MHz, C_6D_6) *δ* = 160.6, 160.5, 143.8 (m_c), 133.4 (d, *J* = 12.5 Hz), 132.8 (d, *J* = 10.7 Hz), 132.0 (d, *J* = 4.9 Hz), 131.8 (d, *J* = 4.2 Hz), 109.2, 80.3 (m_c), 64.1, 64.0, 37.4, 36.1, 33.8 (d, *J* = 4.6 Hz), 33.6 (d, *J* = 4.4 Hz), 32.3, 25.5, 24.1 ppm.

^{31}P-NMR (162 MHz, C_6D_6): *δ* = –23.5 ppm.

HR-MS ($C_{70}H_{109}O_6P_2^+$; [M+H]$^+$, pos. ESI): calcd: 1107.7694, found: 1107.7694.

(((4*S*,5*S*)-2,2-Dimethyl-1,3-dioxolane-4,5-diyl)bis(methylene))bis(bis(3,5-di-*tert*-butyl-4-methoxyphenyl)phosphane) (L24)

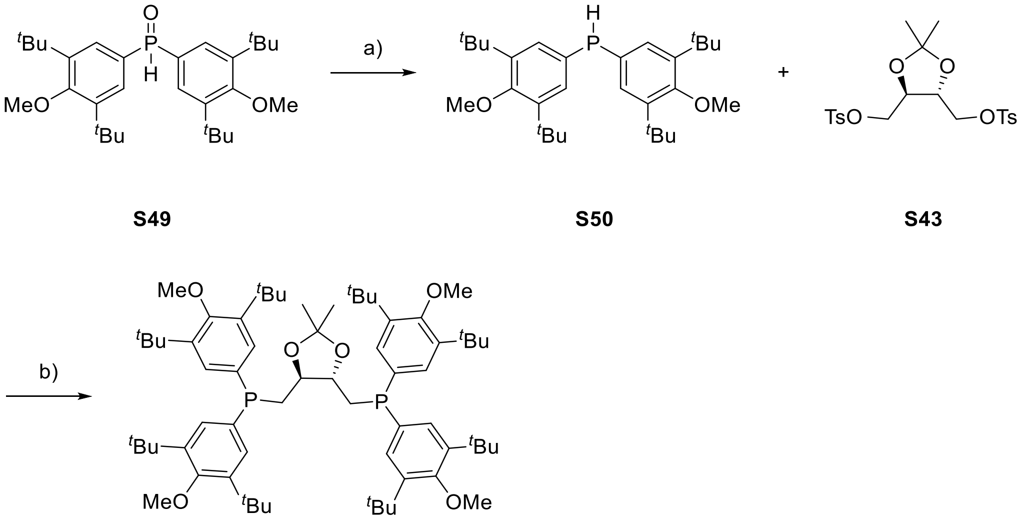

S49 S50 S43

L24

a) To a solution of DIBAL-H (1 M in hexane, 24.7 mL, 24.7 mmol, 3.00 equiv) at 65 °C under argon atmosphere was added a solution of **S49** (4.00 g, 8.22 mmol, 1.00 equiv) in THF (30 mL) over 10 min. After 90 min, the mixture was cooled to rt and a degassed, aqueous NaOH solution (5%, 20 mL) was slowly added. The aqueous phase was removed and the organic phase was transferred into SCHLENK flask and concentrated under vacuum to afford the target product (82% purity) as a white solid. Due to its ability to rapid oxidation, the compound was readily used in the next step.

This procedure is based on a previously reported one.[26]

b) Phosphin **S50** (3.87 g, 8.22 mmol, 2.10 equiv) was solved in THF (40 mL) cooled down to –78 °C and *n*-BuLi (2.5 M in hexane) was added until the mixture turned permanently orange. Then *n*-BuLi (2.5 M in hexane, 5.17 mL, 12.9 mmol, 3.30 equiv) was carefully added. The yellow reaction mixture was allowed to come to rt and stirred for 1 h. **S43** (1.84 g, 3.91 mmol, 1.00 equiv) in THF (20 mL) was added and the mixture was stirred at rt overnight. MeOH (40 mL) and H_2O (40 mL) were added and the reaction mixture was concentrated under reduced pressure. The residue was extracted with CH_2Cl_2 (3 × 60 mL), the combined organic extracts were washed with brine (60 mL), dried over Na_2SO_4 and concentrated under reduced pressure. The crude product was purified via flash column chromatography [SiO_2, *n*-pentane/CH_2Cl_2 2:1 to 1/1 v/v, R_f = 0.27 (*n*-pentane/CH_2Cl_2 2:1 v/v)] to afford the ligand **L24** (1.86 g, 1.75 mmol, 45%) as a colorless solid.

^{1}H-NMR (500 MHz, C_6D_6) δ = 7.74 (d, *J* = 7.7 Hz, 4H), 7.69 (d, *J* = 7.6 Hz, 4H), 4.32 (q, *J* = 5.2 Hz, 2H), 3.38 (d, *J* = 12.5 Hz, 12H), 2.78 (dd, *J* = 13.7, 4.5 Hz, 2H), 2.71 (dd, *J* = 13.7, 6.4 Hz, 2H), 1.45 (d, *J* = 9.4 Hz, 72H), 1.35 (s, 6H) ppm.

^{13}C-NMR (126 MHz, C_6D_6) δ = 160.7, 160.5, 143.9 (m_c), 143.8 (m_c), 133.3 (d, *J* = 12 Hz), 132.7 (d, *J* = 12 Hz), 132.0 (d, *J* = 22 Hz), 131.8 (d, *J* = 21 Hz), 108.7, 80.7 (m_c), 64.2, 64.1, 36.1, 33.6 (d, *J* = 13 Hz), 32.4 (d, *J* = 3 Hz), 32.4, 27.7 ppm.

^{31}P-NMR (203 MHz, C_6D_6): δ = –23.0 ppm.

HR-MS ($C_{67}H_{105}O_6P_2^+$; $[M+H]^+$, pos. APCI): calcd: 1067.7381, found: 1067.7402.

The analytical data corresponded to the reported literature values.[22]

The corresponding (*R*,*R*)-enantiomer of **L24** could be obtained analogously from (*S*,*S*)-**S43**.

4 Catalysis Results

(*S*)-1-(((3-(Cyclohexylethynyl)pent-4-en-1-yl)oxy)methyl)-4-methoxybenzene (3aa)

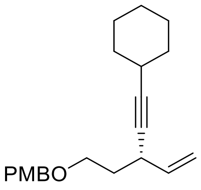

3aa

3aa was synthesized from 1-methoxy-4-((penta-3,4-dien-1-yloxy)methyl)benzene (40.0 µL, 40.9 mg, 200 µmol, 1.00 equiv) and ethynylcyclohexane (26.1 µL, 21.6 mg, 200 µmol, 1.00 equiv) according to **GP 4**. Purification by flash column chromatography [SiO_2, *n*-pentane/Et_2O 20:1 v/v, R_f = 0.30 (*n*-pentane/Et_2O 2:1 v/v)] gave the title compound **3aa** (46.2 mg, 148 µmol, 74%) as a colorless oil. Absolute configuration assigned by analogy.

^{1}H-NMR (500 MHz, $CDCl_3$) δ = 7.29 – 7.24 (m, 2H), 6.90 – 6.85 (m, 2H), 5.78 (ddd, *J* = 16.9, 10.0, 5.9 Hz, 1H), 5.30 (dt, *J* = 16.9, 1.7 Hz, 1H), 5.05 (dt, *J* = 10.0, 1.5 Hz, 1H), 4.45 (s, 2H), 3.80 (s, 3H), 3.66 – 3.43 (m, 2H), 3.26 (dtq, *J* = 9.1, 5.6, 1.7 Hz, 1H), 2.37 (ddd, *J* = 11.6, 7.5, 4.5 Hz, 1H), 1.88 – 1.82 (m, 1H), 1.80 – 1.63 (m, 5H), 1.54 – 1.24 (m, 6H) ppm.

^{13}C-NMR (126 MHz, $CDCl_3$) δ = 159.3, 138.8, 130.9, 129.4, 114.7, 113.9, 88.4, 79.9, 72.9, 67.8, 55.4, 35.8, 33.2, 32.5, 29.3, 26.1, 25.0 ppm.

HR-MS ($C_{21}H_{29}O_2$; $[M+H]^+$, pos. APCI): calcd: 313.2162, found: 313.2155.

HPLC (LC-3, λ = 228 nm, *n*-heptane:EtOH = 99.7:0.3, 0.3 mL/min): τ_R = 19.1 min (minor) and 20.6 min (major), 96% *ee*.

$[\alpha]_D^{20}$ = −4.20 (c = 1.00 g $(100\ mL)^{-1}$, $CHCl_3$).

(*S*)-1-Methoxy-4-(((3-(phenylethynyl)pent-4-en-1-yl)oxy)methyl)benzene (3ab)

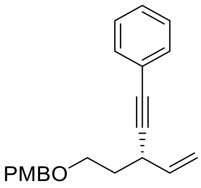

3ab

3ab was synthesized from 1-methoxy-4-((penta-3,4-dien-1-yloxy)methyl)benzene (40.0 µL, 40.9 mg, 200 µmol, 1.00 equiv) and ethynylbenzene (22.0 µL, 20.4 mg, 200 µmol, 1.00 equiv) according to **GP 4**. Purification by flash column chromatography [SiO_2, *n*-pentane/Et_2O 9:1 v/v, R_f = 0.35 (*n*-pentane/Et_2O

9:1 v/v)] gave the title compound **3ab** (49.6 mg, 162 µmol, 81%) as a colorless oil. Absolute configuration assigned by analogy.

^{1}H-NMR (400 MHz, $CDCl_3$) δ = 7.40 – 7.36 (m, 2H), 7.30 – 7.25 (m, 5H), 6.92 – 6.83 (m, 2H), 5.85 (ddd, J = 16.9, 10.0, 6.1 Hz, 1H), 5.37 (dt, J = 17.0, 1.5 Hz, 1H), 5.12 (dt, J = 10.0, 1.4 Hz, 1H), 4.47 (s, 2H), 3.78 (s, 3H), 3.73 – 3.58 (m, 2H), 3.56 – 3.45 (m, 1H), 2.02 – 1.80 (m, 2H) ppm.

^{13}C-NMR (101 MHz, $CDCl_3$) δ = 159.4, 137.8, 131.8, 130.8, 129.4, 128.3, 127.9, 123.9, 115.4, 114.0, 90.0, 83.9, 72.9, 67.6, 55.4, 35.6, 33.1 ppm.

HR-MS ($C_{21}H_{23}O_2^+$; $[M+H]^+$, pos. APCI): calcd: 307.1693, found: 307.1692.

HPLC (AD-3, λ = 240 nm, *n*-heptane:IPA = 99:1, 0.5 mL/min): τ_R = 7.1 min (minor) and 7.4 min (major), 94% *ee*.

$[\alpha]_D^{20}$ = −4.40 (c = 1.00 g (100 mL)$^{-1}$, $CHCl_3$).

(*S*)-1-Fluoro-4-(3-(2-((4-methoxybenzyl)oxy)ethyl)pent-4-en-1-yn-1-yl)benzene (3ac)

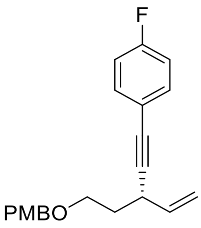

3ac

3ac was synthesized from 1-methoxy-4-((penta-3,4-dien-1-yloxy)methyl)benzene (40.0 µL, 40.9 mg, 200 µmol, 1.00 equiv) and 1-ethynyl-4-fluorbenzene (24.0 mg, 200 µmol, 1.00 equiv) according to **GP 4**. Purification by flash column chromatography [SiO_2, *n*-pentane/Et_2O 9:1 v/v, R_f = 0.30 (*n*-pentane/Et_2O 9:1 v/v)] gave the title compound **3ac** (51.6 mg, 159 µmol, 80%) as a colorless oil. Absolute configuration assigned by analogy.

^{1}H-NMR (500 MHz, $CDCl_3$) δ = 7.38 – 7.31 (m, 2H), 7.30 – 7.26 (m, 2H), 7.04 – 6.94 (m, 2H), 6.91 – 6.83 (m, 2H), 5.84 (ddd, J = 17.0, 10.0, 6.2 Hz, 1H), 5.36 (dq, J = 16.9, 1.3 Hz, 1H), 5.13 (dt, J = 10.0, 1.3 Hz, 1H), 4.51 – 4.43 (m, 2H), 3.79 (s, 3H), 3.73 – 3.58 (m, 2H), 3.55 – 3.43 (m, 1H), 2.03 – 1.77 (m, 2H) ppm.

^{13}C-NMR (126 MHz, $CDCl_3$) δ = 163.3, 159.3, 137.7, 133.6 (d, J = 8 Hz), 130.7, 129.5, 119.88 (d, J = 4 Hz), 115.5 (d, J = 22.1 Hz), 115.5, 113.9, 89.6, 82.8, 72.9, 67.5, 55.4, 35.5, 33.0 ppm.

^{19}F-NMR (471 MHz, $CDCl_3$) δ = –112.0 (m_c) ppm.

HR-MS ($C_{21}H_{22}FO_2^+$; $[M+H]^+$, pos. APCI): calcd: 325.1598, found: 325.1608.

HPLC (AD-3, λ = 240 nm, *n*-heptane:IPA = 95:5, 0.5 mL/min): τ_R = 6.7 min (minor) and 7.0 min (major), 95% *ee*.

$[\alpha]_D^{20}$ = −0.90 (c = 1.00 g (100 mL)$^{-1}$, $CHCl_3$).

(*S*)-1-Methoxy-4-(3-(2-((4-methoxybenzyl)oxy)ethyl)pent-4-en-1-yn-1-yl)benzene (3ad)

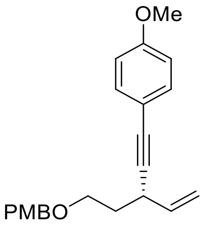

3ad

3ad was synthesized from 1-methoxy-4-((penta-3,4-dien-1-yloxy)methyl)benzene (40.0 µL, 40.9 mg, 200 µmol, 1.00 equiv) and 1-ethynyl-4-methoxybenzene (26.4 mg, 200 µmol, 1.00 equiv) according to **GP 4**. Purification by flash column chromatography [SiO_2, *n*-pentane/Et_2O 9:1 v/v, R_f = 0.30 (*n*-pentane/Et_2O 9:1 v/v)] gave the title compound **3ad** (53.3 mg, 158 µmol, 79%) as a colorless oil. Absolute configuration assigned by analogy.

^{1}H-NMR (400 MHz, $CDCl_3$) δ = 7.38 – 7.26 (m, 4H), 6.93 – 6.77 (m, 4H), 5.85 (ddd, *J* = 17.0, 10.0, 6.2 Hz, 1H), 5.36 (dt, *J* = 17.0, 1.5 Hz, 1H), 5.12 (dt, *J* = 10.0, 1.4 Hz, 1H), 4.47 (s, 2H), 3.80 (s, 3H), 3.79 (s, 3H), 3.75 – 3.58 (m, 2H), 3.55 – 3.41 (m, 1H), 2.01 – 1.78 (m, 2H) ppm.

^{13}C-NMR (101 MHz, $CDCl_3$) δ = 159.4, 159.3, 138.0, 133.1, 130.8, 129.4, 116.1, 115.3, 114.0, 88.4, 83.7, 72.9, 67.7, 55.4, 35.7, 33.1 ppm.

HR-MS ($C_{22}H_{25}O_3^+$; $[M+H]^+$, pos. APCI): calcd: 337.1798, found: 337.1803.

HPLC (AD-3, λ = 240 nm, *n*-heptane:IPA = 95:5, 0.5 mL/min): τ_R = 9.6 min (minor) and 12.4 min (major), 95% *ee*.

$[\alpha]_D^{25}$ = −4.90 (c = 1.00 g (100 mL)$^{-1}$, $CHCl_3$).

(*S*)-2-(3-(2-((4-Methoxybenzyl)oxy)ethyl)pent-4-en-1-yn-1-yl)phenol (3ae)

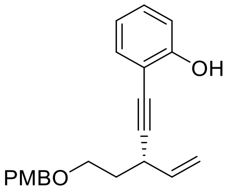

3ae

3ae was synthesized from 1-methoxy-4-((penta-3,4-dien-1-yloxy)methyl)benzene (40.0 µL, 40.9 mg, 200 µmol, 1.00 equiv) and 2-ethynylphenol (23.6 mg, 200 µmol, 1.00 equiv) according to **GP 4**. Purification by flash column chromatography [SiO_2, *n*-pentane/Et_2O 4:1 v/v, R_f = 0.23 (*n*-pentane/Et_2O 4:1 v/v)] gave the title compound **3ae** (35.8 mg, 111 µmol, 56%) as a colorless oil. Absolute configuration assigned by analogy.

^{1}H-NMR (500 MHz, $CDCl_3$) δ = 7.31 – 7.26 (m, 3H), 7.26 – 7.22 (ddd, J = 8.2, 7.4, 1.7 Hz, 1H), 6.93 (dd, J = 8.2, 1.1 Hz, 1H), 6.89 – 6.86 (m, 2H), 6.86 – 6.84 (ddd, J = 7.5, 7.5, 1.1 Hz, 1H), 6.31 (s, 1H), 5.84 (ddd, J = 16.9, 10.0, 6.1 Hz, 1H), 5.38 (ddd, J = 17.0, 1.4, 1.4 Hz, 1H), 5.16 (ddd, J = 10.0, 1.3, 1.3 Hz, 1H), 4.50 (s, 2H), 3.79 (s, 3H), 3.71 – 3.53 (m, 3H), 2.03 – 1.84 (m, 2H) ppm.

^{13}C-NMR (126 MHz, $CDCl_3$) δ = 159.3, 157.3, 137.1, 131.4, 130.3, 129.9, 129.5, 120.0, 115.9, 114.6, 113.9, 110.0, 97.6, 78.1, 72.8, 67.3, 55.3, 35.4, 33.7 ppm.

HR-MS ($C_{21}H_{23}O_3^+$; $[M+H]^+$, pos. APCI): calcd: 323.1642, found: 323.1650.

HPLC (AD-3, λ = 212 nm, *n*-heptane:EtOH = 95:5, 0.5 mL/min): τ_R = 22.4 min (minor) and 26.1 min (major), 88% *ee*.

$[\alpha]_D^{20}$ = +7.81 (c = 700 mg (100 mL)$^{-1}$, $CHCl_3$).

(*S*)-2-(3-(2-((4-Methoxybenzyl)oxy)ethyl)pent-4-en-1-yn-1-yl)aniline (3af)

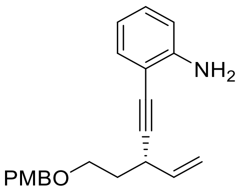

3af

3af was synthesized from 1-methoxy-4-((penta-3,4-dien-1-yloxy)methyl)benzene (40.0 µL, 40.9 mg, 200 µmol, 1.00 equiv) and 2-ethynylaniline (22.5 µL, 23.4 mg, 200 µmol, 1.00 equiv) according to **GP 4**. Purification by flash column chromatography [SiO_2, *n*-pentane/Et_2O 3:2 v/v, R_f = 0.35 (*n*-pentane/Et_2O 3:2 v/v)] gave the title compound **3af** (44.9 mg, 140 µmol, 70%) as a colorless oil. Absolute configuration assigned by analogy.

^{1}H-NMR (400 MHz, $CDCl_3$) δ = 7.31 – 7.26 (m, 2H), 7.26 – 7.23 (ddd, J = 7.4, 1.6, 0.7 Hz, 1H), 7.09 (ddd, J = 8.1, 7.4, 1.6 Hz, 1H), 6.91 – 6.85 (m, 2H), 6.72 – 6.61 (m, 2H), 5.87 (ddd, J = 17.0, 10.0, 6.1 Hz, 1H), 5.38 (ddd, J = 17.0, 1.5, 1.5 Hz, 1H), 5.13 (ddd, J = 10.0, 1.4, 1.4 Hz, 1H), 4.47 (d, J = 1.0 Hz, 2H), 4.12 (s, 2H), 3.79 (s, 3H), 3.75 – 3.61 (m, 2H), 3.61 – 3.54 (m, 1H), 2.04 – 1.80 (m, 2H) ppm.

^{13}C-NMR (101 MHz, $CDCl_3$) δ = 159.3, 147.8, 137.8, 132.2, 130.6, 129.4, 129.2, 117.8, 115.4, 114.2, 113.9, 108.5, 95.3, 80.3, 72.8, 67.5, 55.4, 35.6, 33.4 ppm.

HR-MS ($C_{21}H_{24}NO_2^+$; $[M+H]^+$, pos. ESI): calcd: 322.1802, found: 322.1805.

HPLC (AD-3, λ = 300 nm, *n*-heptane:IPA = 95:5, 0.5 mL/min): τ_R = 24.0 min (minor) and 29.6 min (major), 95% *ee*.

$[\alpha]_D^{21}$ = +5.64 (c = 1.29 g (100 mL)$^{-1}$, $CHCl_3$). (The optical rotation was determined for **ent-3af**.)

(*S*)-*N*-(2-(3-(2-((4-Methoxybenzyl)oxy)ethyl)pent-4-en-1-yn-1-yl)phenyl)-4-methylbenzenesulfonamide (3ag)

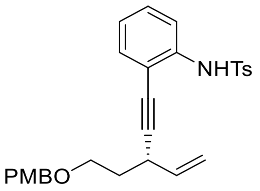

3ag

3ag was synthesized from 1-methoxy-4-((penta-3,4-dien-1-yloxy)methyl)benzene (40.0 µL, 40.9 mg, 200 µmol, 1.00 equiv) and *N*-(2-ethynylphenyl)-4-methylbenzenesulfonamide (54.3 mg, 200 µmol, 1.00 equiv) according to **GP 4**. Purification by flash column chromatography [SiO_2, *n*-pentane/Et_2O 4:1 v/v, R_f = 0.23 (*n*-pentane/Et_2O 4:1 v/v)] gave the title compound **3ag** (54.6 mg, 115 µmol, 57%) as a colorless oil. Absolute configuration assigned by analogy.

^{1}H-NMR (400 MHz, $CDCl_3$) δ = 7.66 – 7.62 (m, 2H), 7.62 – 7.59 (dd, J = 7.8, 1.1 Hz, 1H), 7.31 – 7.26 (m, 2H), 7.26 – 7.22 (dd, J = 7.7, 0.9 Hz, 2H), 7.19 – 7.15 (m, 2H), 7.02 – 6.97 (m, 1H), 6.88 – 6.83 (m, 2H), 5.80 (ddd, J = 17.0, 10.0, 6.3 Hz, 1H), 5.30 – 5.24 (m, 1H), 5.16 (ddd, J = 10.0, 1.3, 1.3 Hz, 1H), 4.49 (s, 2H), 3.78 (s, 3H), 3.67 – 3.56 (m, 2H), 3.51 (dtt, J = 8.8, 6.1, 1.4 Hz, 1H), 2.35 (s, 3H), 2.00 – 1.78 (m, 2H) ppm.

^{13}C-NMR (101 MHz, $CDCl_3$) δ = 159.3, 143.9, 137.8, 137.0, 136.3, 132.0, 130.4, 129.6, 129.5, 129.2, 127.3, 124.3, 119.8, 116.0, 114.7, 113.9, 97.6, 78.5, 72.8, 67.2, 55.3, 35.4, 33.3, 21.6 ppm.

HR-MS ($C_{28}H_{29}NNaSO_4^+$; $[M+Na]^+$, pos. ESI): calcd: 498.1710, found: 498.1713.

HPLC (AD-3, λ = 228 nm, *n*-heptane:EtOH = 85:15, 0.5 mL/min): τ_R = 16.5 min (minor) and 25.4 min (major), 87% *ee*.

$[\alpha]_D^{20}$ = −1.31 (c = 1.13 g (100 mL)$^{-1}$, $CHCl_3$).

(*S*)-3-(3-(2-((4-Methoxybenzyl)oxy)ethyl)pent-4-en-1-yn-1-yl)aniline (3ah)

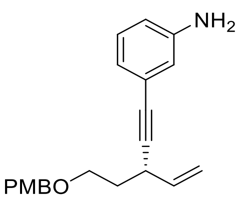

3ah

3ah was synthesized from 1-methoxy-4-((penta-3,4-dien-1-yloxy)methyl)benzene (40.0 µL, 40.9 mg, 200 µmol, 1.00 equiv) and 3-ethynylaniline (23.0 µL, 23.4 mg, 200 µmol, 1.00 equiv) according to **GP 4**. Purification by flash column chromatography [SiO_2, *n*-pentane/Et_2O 1:1 v/v, R_f = 0.35 (*n*-pentane/Et_2O

1:1 v/v)] gave the title compound **3ah** (47.3 mg, 147 µmol, 74%) as a brown oil. Absolute configuration assigned by analogy.

^{1}H-NMR (500 MHz, $CDCl_3$) δ = 7.31 – 7.26 (m, 2H), 7.06 (t, J = 7.8 Hz, 1H), 6.92 – 6.84 (m, 2H), 6.84 – 6.76 (m, 1H), 6.70 – 6.65 (m, 1H), 6.63 – 6.57 (m, 1H), 5.84 (ddd, J = 17.0, 10.0, 6.1 Hz, 1H), 5.37 (dt, J = 17.0, 1.5 Hz, 1H), 5.12 (dt, J = 10.0, 1.4 Hz, 1H), 4.52 – 4.41 (m, 2H), 3.80 – 3.40 (bs, 2H), 3.79 (s, 3H), 3.75 – 3.57 (m, 2H), 3.54 – 3.45 (m, 1H), 2.05 – 1.77 (m, 2H) ppm.

^{13}C-NMR (126 MHz, $CDCl_3$) δ = 159.3, 146.3, 137.8, 130.8, 129.5, 129.2, 124.5, 122.2, 118.1, 115.4, 114.9, 114.0, 89.3, 84.1, 72.9, 67.5, 55.4, 35.6, 33.1 ppm.

HR-MS ($C_{21}H_{24}NO_2^+$; $[M+H]^+$, pos. APCI): calcd: 322.1802, found: 322.1800.

HPLC (AD-3, *n*-heptane:EtOH = 85:15, 0.5 mL/min): τ_R = 19.2 min (minor) and 20.1 min (major), 91% *ee*.

$[\alpha]_D^{20}$ = +42.0 (c = 1.00 g (100 mL)$^{-1}$, $CHCl_3$).

(*S*)-2-(3-(2-((4-Methoxybenzyl)oxy)ethyl)pent-4-en-1-yn-1-yl)thiophene (3ai)

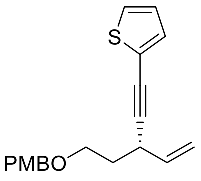

3ai

3ai was synthesized from 1-methoxy-4-((penta-3,4-dien-1-yloxy)methyl)benzene (40.0 µL, 40.9 mg, 200 µmol, 1.00 equiv) and 2-ethynylthiophene (19.0 µL, 21.6 mg, 200 µmol, 1.00 equiv) according to **GP 4**. Purification by flash column chromatography [SiO_2, *n*-pentane/Et_2O 9:1 v/v, R_f = 0.30 (*n*-pentane/Et_2O 9:1 v/v)] gave the title compound **3ai** (39.1 mg, 125 µmol, 63%) as a light-yellow oil. Absolute configuration assigned by analogy.

^{1}H-NMR (500 MHz, $CDCl_3$) δ = 7.30 – 7.26 (m, 2H), 7.19 (dd, J = 5.2, 1.2 Hz, 1H), 7.12 (dd, J = 3.6, 1.2 Hz, 1H), 6.94 (dd, J = 5.2, 3.6 Hz, 1H), 6.91 – 6.84 (m, 2H), 5.83 (ddd, J = 16.9, 10.0, 6.2 Hz, 1H), 5.35 (dt, J = 17.0, 1.4 Hz, 1H), 5.13 (dt, J = 10.0, 1.4 Hz, 1H), 4.47 (s, 2H), 3.79 (s, 3H), 3.71 – 3.57 (m, 2H), 3.58 – 3.46 (m, 1H), 2.02 – 1.80 (m, 2H) ppm.

^{13}C-NMR (126 MHz, $CDCl_3$) δ = 159.3, 137.4, 131.4, 130.7, 129.4, 126.9, 126.3, 123.9, 115.7, 113.9, 94.0, 72.9, 68.9, 67.5, 55.4, 35.4, 33.3 ppm.

HR-MS ($C_{19}H_{21}O_2S^+$; $[M+H]^+$, pos. APCI): calcd: 313.1257, found: 313.1266.

HPLC (LC-3, λ = 277 nm, *n*-heptane:EtOH = 95:5, 0.5 mL/min): τ_R = 20.263 min (minor) and 22.967 min (major), 94% *ee*.

$[\alpha]_D^{20}$ = −4.90 (c = 1.00 g (100 mL)$^{-1}$, $CHCl_3$).

(*S*)-1-(((3-(Cyclopentylethynyl)pent-4-en-1-yl)oxy)methyl)-4-methoxybenzene (3aj)

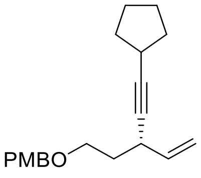

3aj

3aj was synthesized from 1-methoxy-4-((penta-3,4-dien-1-yloxy)methyl)benzene (40.0 µL, 40.9 mg, 200 µmol, 1.00 equiv) and ethynylcyclopentane (23.2 µL, 18.8 mg, 200 µmol, 1.00 equiv) according to **GP 4**. Purification by flash column chromatography [SiO_2, *n*-pentane/Et_2O 15:1 v/v, $R_f = 0.30$ (*n*-pentane/Et_2O 15:1 v/v)] gave the title compound **3aj** (43.9 mg, 147 µmol, 74%) as a colorless oil. Absolute configuration assigned by analogy.

^{1}H-NMR (400 MHz, $CDCl_3$) δ = 7.29 – 7.22 (m, 2H), 6.90 – 6.84 (m, 2H), 5.77 (ddd, *J* = 16.9, 10.0, 6.0 Hz, 1H), 5.28 (ddd, *J* = 16.9, 1.6, 1.6 Hz, 1H), 5.04 (ddd, *J* = 10.0, 1.6, 1.6 Hz, 1H), 4.44 (s, 2H), 3.80 (s, 3H), 3.65 – 3.52 (m, 2H), 3.29 – 3.20 (m, 1H), 2.65 – 2.56 (m, 1H), 1.95 – 1.47 (m, 10H) ppm.

^{13}C-NMR (101 MHz, $CDCl_3$) δ = 159.3, 138.7, 130.8, 129.3, 114.6, 113.9, 88.3, 79.5, 72.8, 67.8, 55.4, 35.8, 34.3, 32.5, 30.4, 25.0 ppm.

HR-MS ($C_{20}H_{27}O_2^+$; $[M+H]^+$, pos. APCI): calcd: 299.2006, found: 299.2008.

HPLC (LC-3, λ = 228 nm, *n*-heptane:EtOH = 99:1, 0.5 mL/min): τ_R = 7.3 min (minor) and 7.6 min (major), 95% *ee*.

$[\alpha]_D^{21}$ = −4.74 (c = 1.78 g (100 mL)$^{-1}$, $CHCl_3$). (The optical rotation was determined for **ent-3aj**.)

(*S*)-1-(((3-(Cyclopropylethynyl)pent-4-en-1-yl)oxy)methyl)-4-methoxybenzene (3ak)

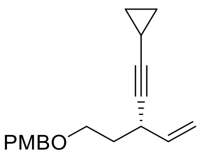

3ak

3ak was synthesized from 1-methoxy-4-((penta-3,4-dien-1-yloxy)methyl)benzene (40.0 µL, 40.9 mg, 200 µmol, 1.00 equiv) and ethynylcyclopropane (17.0 µL, 13.2 mg, 200 µmol, 1.00 equiv) according to **GP 4**. Purification by flash column chromatography [SiO_2, *n*-pentane/Et_2O 9:1 v/v, $R_f = 0.40$ (*n*-pentane/Et_2O 9:1 v/v)] gave the title compound **3ak** (33.0 mg, 122 µmol, 61%) as a colorless oil. Absolute configuration assigned by analogy.

^{1}H-NMR (500 MHz, $CDCl_3$) δ = 7.29 – 7.25 (m, 2H), 6.90 – 6.86 (m, 2H), 5.75 (ddd, *J* = 16.9, 10.0, 6.2 Hz, 1H), 5.25 (dt, *J* = 16.9, 1.6 Hz, 1H), 5.04 (dt, *J* = 10.0, 1.5 Hz, 1H), 4.44 (s, 2H), 3.81 (s, 3H), 3.63 – 3.51 (m, 2H), 3.21 (dtt, *J* = 9.0, 5.9, 1.6 Hz, 1H), 1.87 – 1.65 (m, 2H), 1.23 (ttd, *J* = 8.3, 5.0, 1.8 Hz, 1H), 0.76 – 0.55 (m, 4H) ppm.

^{13}C-NMR (126 MHz, $CDCl_3$) δ = 159.3, 138.6, 130.8, 129.4, 114.9, 113.9, 86.9, 75.4, 72.8, 67.7, 55.4, 35.7, 32.5, 8.4, 8.3, −0.3 ppm.

HR-MS ($C_{18}H_{23}O_2^+$; $[M+H]^+$, pos. APCI): calcd: 271.1693, found: 271.1699.

HPLC (AD-3, *n*-heptane:IPA = 99.6:0.4, 0.5 mL/min): τ_R = 8.8 min (minor) and 9.2 min (major), 93% *ee*.

$[\alpha]_D^{20}$ = −7.10 (c = 1.69 g (100 mL)$^{-1}$, $CHCl_3$).

(*S,E*)-1-Methoxy-4-(((9-phenyl-3-vinylnon-6-en-4-yn-1-yl)oxy)methyl)benzene (3al)

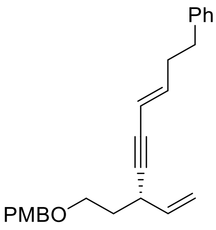

3al

3al was synthesized from 1-methoxy-4-((penta-3,4-dien-1-yloxy)methyl)benzene (40.0 µL, 40.9 mg, 200 µmol, 1.00 equiv) and (*E*)-hex-3-en-5-yn-1-ylbenzene (33.2 µL, 31.2 mg, 200 µmol, 1.00 equiv) according to **GP 4**. Purification by flash column chromatography [SiO_2, *n*-pentane/Et_2O 9:1 v/v, R_f = 0.33 (*n*-pentane/Et_2O 1:1 v/v)] gave the title compound **3al** (36.8 mg, 102 µmol, 51%) as a colorless oil. Absolute configuration assigned by analogy.

^{1}H-NMR (500 MHz, $CDCl_3$) δ = 7.32 – 7.25 (m, 4H), 7.23 – 7.15 (m, 3H), 6.90 – 6.85 (m, 2H), 6.10 (dt, *J* = 15.7, 6.9 Hz, 1H), 5.78 (ddd, *J* = 17.0, 10.0, 6.3 Hz, 1H), 5.53 (ddt, *J* = 15.8, 1.7, 1.7 Hz, 1H), 5.28 (ddd, *J* = 17.0, 1.5, 1.5 Hz, 1H), 5.08 (ddd, *J* = 10.0, 1.4, 1.4 Hz, 1H), 4.45 (s, 2H), 3.81 (s, 3H), 3.67 – 3.53 (m, 2H), 3.43 – 3.34 (m, 1H), 2.76 – 2.65 (m, 2H), 2.65 – 2.42 (m, 2H), 1.94 – 1.72 (m, 2H) ppm.

^{13}C-NMR (126 MHz, $CDCl_3$) δ = 159.2, 142.6, 141.4, 137.9, 130.7, 129.4, 128.5, 128.5, 126.1, 115.2, 113.9, 110.4, 88.7, 82.2, 72.8, 67.6, 55.4, 35.5, 35.3, 34.8, 33.0 ppm.

HR-MS ($C_{25}H_{29}O_2^+$; $[M+H]^+$, pos. APCI): calcd: 361.2162, found: 361.2156.

HPLC (LC-3, λ = 228 nm, *n*-heptane:IPA = 95:5, 0.5 mL/min): τ_R = 18.7 min (minor) and 19.6 min (major), 94% *ee*.

$[\alpha]_D^{20}$ = +3.81 (c = 1.19 g (100 mL)$^{-1}$, $CHCl_3$).

Tert-butyl (S)-(4-(2-((4-methoxybenzyl)oxy)ethyl)hex-5-en-2-yn-1-yl)carbamate (3am)

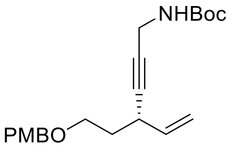

3am

3am was synthesized from 1-methoxy-4-((penta-3,4-dien-1-yloxy)methyl)benzene (40.0 µL, 40.9 mg, 200 µmol, 1.00 equiv) and *tert*-butyl prop-2-yn-1-ylcarbamate (31.0 mg, 200 µmol, 1.00 equiv) according to **GP 4**. Purification by flash column chromatography chromatography [SiO_2, *n*-pentane/Et_2O 3:1 v/v, R_f = 0.30 (*n*-pentane/Et_2O 3:1 v/v)] gave the title compound **3am** (32.4 mg, 90.0 µmol, 45%) as a yellowish oil. Absolute configuration assigned by analogy.

^{1}H-NMR (500 MHz, $CDCl_3$) δ = 7.31 – 7.21 (m, 2H), 6.93 – 6.84 (m, 2H), 5.74 (ddd, J = 17.0, 10.0, 6.2 Hz, 1H), 5.26 (dt, J = 17.0, 1.5 Hz, 1H), 5.07 (dt, J = 10.0, 1.4 Hz, 1H), 4.60 (s, 1H), 4.44 (s, 2H), 3.92 (s, 2H), 3.81 (s, 3H), 3.64 – 3.50 (m, 2H), 3.31 – 3.23 (m, 1H), 1.91 – 1.69 (m, 2H), 1.45 (s, 9H) ppm.

^{13}C-NMR (126 MHz, $CDCl_3$) δ = 159.3, 155.4, 137.7, 130.7, 129.4, 115.4, 113.9, 83.6, 79.5, 72.8, 67.4, 55.4, 35.4, 32.4, 29.9, 28.5 ppm.

HR-MS ($C_{21}H_{29}NNaO_4^+$; $[M+Na]^+$, pos. APCI): calcd: 382.1989, found: 382.1984.

HPLC (LC-4, λ = 228 nm, *n*-heptane:EtOH = 99:1, 0.5 mL/min): τ_R = 15.3 min (major) and 16.2 min (minor), 96% *ee*.

$[\alpha]_D^{20}$ = −7.50 (c = 1.00 g (100 mL)$^{-1}$, $CHCl_3$).

(S)-4,4'-(((5-Vinylhept-3-yne-1,7-diyl)bis(oxy))bis(methylene))bis(methoxybenzene) (3an)

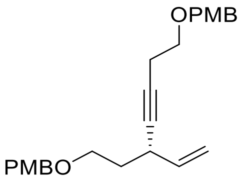

3an

3an was synthesized from 1-methoxy-4-((penta-3,4-dien-1-yloxy)methyl)benzene (40.0 µL, 40.9 mg, 200 µmol, 1.00 equiv) and 1-((but-3-yn-1-yloxy)methyl)-4-methoxybenzene (12.0 µL, 11.2 mg, 200 µmol, 1.00 equiv) according to **GP 4**. Purification by flash column chromatography [SiO_2, *n*-pentane/Et_2O 1:1 v/v, R_f = 0.35 (*n*-pentane/Et_2O 1:1 v/v)] gave the title compound **3an** (61.5 mg, 156 µmol, 78%) as a yellowish oil. Absolute configuration assigned by analogy.

^{1}H-NMR (500 MHz, $CDCl_3$) δ = 7.30 – 7.21 (m, 4H), 6.91 – 6.84 (m, 4H), 5.76 (ddd, J = 16.9, 10.0, 6.1 Hz, 1H), 5.27 (dt, J = 17.0, 1.6 Hz, 1H), 5.05 (dt, J = 10.0, 1.5 Hz, 1H), 4.47 (s, 2H), 4.43 (s, 2H),

3.80 (s, 3H), 3.80 (s, 3H), 3.63 – 3.42 (m, 4H), 3.25 (dddd, *J* = 8.2, 6.2, 4.3, 2.4 Hz, 1H), 2.49 (td, *J* = 7.2, 2.2 Hz, 2H), 1.91 – 1.66 (m, 2H) ppm.

^{13}C-NMR (126 MHz, $CDCl_3$) δ = 159.4, 159.3, 138.3, 130.8, 130.5, 129.4, 129.4, 115.0, 114.0, 113.9, 81.3, 80.3, 72.8, 72.7, 68.8, 67.7, 55.4, 35.6, 32.6, 20.4 ppm.

HR-MS ($C_{25}H_{31}O_4^+$; $[M+H]^+$, pos. APCI): calcd: 395.2217, found: 395.2222.

HPLC (LC-4, λ = 228 nm, *n*-heptane:EtOH = 99.5:0.5, 0.5 mL/min): τ_R = 13.1 min (major) and 14.0 min (minor), 87% *ee*.

$[\alpha]_D^{20}$ = −2.50 (c = 1.00 g (100 mL)$^{-1}$, $CHCl_3$).

(*S*)-5-(2-((4-Methoxybenzyl)oxy)ethyl)-2-methylhept-6-en-3-yn-2-ol (3ao)

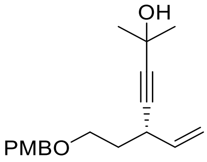

3ao

3ao was synthesized from 1-methoxy-4-((penta-3,4-dien-1-yloxy)methyl)benzene (40.0 µL, 40.9 mg, 200 µmol, 1.00 equiv) and 2-methylbut-3-yn-2-ol (20.0 µL, 16.8 mg, 200 µmol, 1.00 equiv) according to **GP 4**. Purification by flash column chromatography [SiO_2, *n*-pentane/Et_2O 2:1 v/v, R_f = 0.40 (*n*-pentane/Et_2O 2:1 v/v)] gave the title compound **3ao** (48.1 mg, 167 µmol, 83%) as a yellowish oil. Absolute configuration assigned by analogy.

^{1}H-NMR (500 MHz, $CDCl_3$) δ = 7.30 – 7.23 (m, 2H), 6.92 – 6.85 (m, 2H), 5.75 (ddd, *J* = 16.9, 10.0, 6.1 Hz, 1H), 5.27 (dt, *J* = 17.0, 1.5 Hz, 1H), 5.07 (dt, *J* = 10.1, 1.5 Hz, 1H), 4.49 – 4.40 (m, 2H), 3.80 (s, 3H), 3.63 – 3.42 (m, 2H), 3.34 – 3.25 (m, 1H), 1.90 – 1.68 (m, 2H), 1.84 (s, 1H), 1.49 (s, 6H) ppm.

^{13}C-NMR (126 MHz, $CDCl_3$) δ = 159.3, 137.8, 130.7, 130.5, 129.5, 129.4, 115.2, 113.9, 88.6, 82.4, 72.8, 67.4, 65.4, 55.4, 35.4, 32.2, 31.9 ppm.

HR-MS ($C_{18}H_{28}NO_3^+$; $[M+NH_4]^+$, pos. APCI): calcd: 306.2064, found: 306.2069.

HPLC (AD-3, λ = 277 nm, *n*-heptane:IPA = 95:5, 0.5 mL/min): τ_R = 21.9 min (minor) and 36.0 min (major), 92% *ee*.

$[\alpha]_D^{20}$ = −1.40 (c = 1.00 g (100 mL)$^{-1}$, $CHCl_3$).

(*S*)-(3-(2-((4-Methoxybenzyl)oxy)ethyl)pent-4-en-1-yn-1-yl)trimethylsilane (3ap)

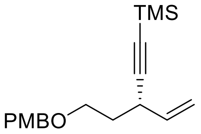

3ap

3ap was synthesized from 1-methoxy-4-((penta-3,4-dien-1-yloxy)methyl)benzene (40.2 µL, 40.9 mg, 200 µmol, 1.00 equiv) and ethynyltrimethylsilane (27.7 µL, 19.6 mg, 200 µmol, 1.00 equiv) according to **GP 4** with 17 h at 80 °C. Purification by flash column chromatography [SiO_2, *n*-pentane/Et_2O 29:1 v/v, R_f = 0.31 (*n*-pentane/Et_2O 19:1 v/v)] gave the title compound **3ap** (20.6 mg, 68.1 µmol, 34%) as a yellowish oil. Absolute configuration assigned by analogy.

^{1}H-NMR (400 MHz, $CDCl_3$) δ = 7.30 – 7.24 (m, 2H), 6.91 – 6.86 (m, 2H), 5.76 (ddd, *J* = 17.0, 10.0, 6.1 Hz, 1H), 5.30 (ddd, *J* = 16.9, 1.5, 1.5 Hz, 1H), 5.08 (ddd, *J* = 10.0, 1.4, 1.4 Hz, 1H), 4.45 (s, 2H), 3.80 (s, 3H), 3.66 – 3.54 (m, 2H), 3.35 – 3.27 (m, 1H), 1.91 – 1.69 (m, 2H), 0.15 (s, 9H) ppm.

^{13}C-NMR (101 MHz, $CDCl_3$) δ = 159.3, 137.5, 130.7, 129.3, 115.4, 113.9, 106.8, 87.8, 72.8, 67.5, 55.4, 35.3, 33.3, 0.2 ppm.

HR-MS ($C_{18}H_{26}O_2Si^+$; $[M+H]^+$, pos. APCI): calcd: 303.1775, found: 303.1773.

HPLC (LC-3, λ = 228 nm, *n*-heptane:EtOH = 99.5:0.5, 0.5 mL/min): τ_R = 7.0 min (minor) and 7.6 min (major), 91% *ee*.

$[\alpha]_D^{21}$ = −2.20 (c = 771 mg $(100\ mL)^{-1}$, $CHCl_3$). (The optical rotation was determined for **ent-3ap**.)

(*S*)-*Tert*-butyldiphenyl((3-(phenylethynyl)pent-4-en-1-yl)oxy)silane (3bb)

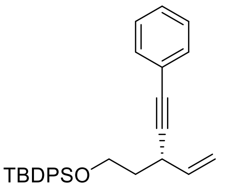

3bb

3bb was synthesized from *tert*-butyl(penta-3,4-dien-1-yloxy)diphenylsilane (63.0 µL, 64.5 mg, 200 µmol, 1.00 equiv) and ethynylbenzene (22.0 µL, 20.4 mg, 200 µmol, 1.00 equiv) according to **GP 4**. Purification by flash column chromatography [SiO_2, *n*-pentane/Et_2O 50:1 v/v, R_f = 0.50 (*n*-pentane/Et_2O 50:1 v/v)] gave the title compound **3bb** (58.4 mg, 138 µmol, 70%) as a yellowish oil. Absolute configuration assigned by analogy.

^{1}H-NMR (400 MHz, $CDCl_3$) δ = 7.74 – 7.65 (m, 4H), 7.47 – 7.27 (m, 11H), 5.86 (ddd, *J* = 17.0, 10.0, 6.1 Hz, 1H), 5.39 (dt, *J* = 17.0, 1.5 Hz, 1H), 5.13 (dt, *J* = 10.0, 1.4 Hz, 1H), 3.98 – 3.79 (m, 2H), 3.67 – 3.58 (m, 1H), 2.00 – 1.77 (m, 2H), 1.07 (s, 9H) ppm.

^{13}C-NMR (101 MHz, $CDCl_3$) δ = 138.0, 135.8, 135.7, 134.1, 134.0, 131.8, 129.7, 129.7, 128.3, 127.8, 123.9, 115.3, 90.2, 83.9, 61.5, 38.3, 32.7, 27.0, 19.4 ppm.

HR-MS ($C_{29}H_{33}OSi^+$; $[M+H]^+$, pos. APCI): calcd: 425.2295, found: 425.2298.

HPLC (OD-3, *n*-heptane:IPA = 99.5:0.5, 0.5 mL/min): τ_R = 4.4 min (major) and 5.0 min (minor), 93% *ee*.

$[\alpha]_D^{20}$ = +8.40 (c = 1.00 g $(100\ mL)^{-1}$, $CHCl_3$).

(*S*)-1-Fluoro-4-((3-(phenylethynyl)pent-4-en-1-yl)oxy)benzene (3cb)

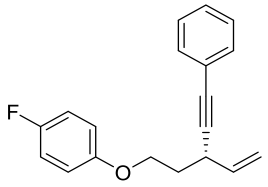

3cb

3cb was synthesized from 1-fluoro-4-(penta-3,4-dien-1-yloxy)benzene (32.0 µL, 36.0 mg, 200 µmol, 1.00 equiv) and ethynylbenzene (22.0 µL, 20.4 mg, 200 µmol, 1.00 equiv) according to **GP 4**. Purification by flash column chromatography [SiO_2, *n*-pentane/Et_2O 20:1 v/v, R_f = 0.55 (*n*-pentane/Et_2O 20:1 v/v)] gave the title compound **3cb** (35.4 mg, 126 µmol, 63%) as a reddish-brown oil. Absolute configuration assigned by analogy.

^{1}H-NMR (500 MHz, $CDCl_3$) δ = 7.47 – 7.37 (m, 2H), 7.34 – 7.27 (m, 3H), 7.00 – 6.93 (m, 2H), 6.88 – 6.85 (m, 2H), 5.90 (ddd, *J* = 16.9, 10.0, 6.1 Hz, 1H), 5.43 (dt, *J* = 17.0, 1.5 Hz, 1H), 5.18 (dt, *J* = 10.0, 1.4 Hz, 1H), 4.25 – 4.06 (m, 2H), 3.60 (dtt, *J* = 8.9, 5.9, 1.5 Hz, 1H), 2.20 – 1.99 (m, 2H) ppm.

^{13}C-NMR (101 MHz, $CDCl_3$) δ = 157.4 (d, *J* = 238 Hz), 155.2, 137.3, 131.8, 128.4, 128.1, 123.6, 116.0, 115.9 (d, *J* = 14 Hz), 115.7 (d, *J* = 8 Hz), 89.4, 84.3, 66.2, 35.0, 33.0 ppm.

^{19}F-NMR (471 MHz, $CDCl_3$) δ = –124.1 (m_c) ppm.

HR-MS ($C_{19}H_{18}FO^+$; $[M+H]^+$, pos. APCI): calcd: 281.1342, found: 281.1336.

HPLC (LC-3, *n*-heptane:EtOH = 99:1, 0.5 mL/min): τ_R = 10.1 min (major) and 12.3 min (minor), 84% *ee*.

$[\alpha]_D^{20}$ = −0.700 (c = 789 mg (100 mL)$^{-1}$, $CHCl_3$).

(*S*)-1-Chloro-4-((3-(phenylethynyl)pent-4-en-1-yl)oxy)benzene (3db)

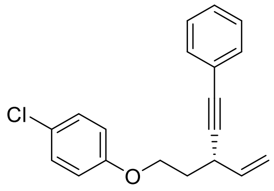

3db

3db was synthesized from 1-chloro-4-(penta-3,4-dien-1-yloxy)benzene (35.0 µL, 38.9 mg, 200 µmol, 1.00 equiv) and ethynylbenzene (22.0 µL, 20.4 mg, 200 µmol, 1.00 equiv) according to **GP 4**. Purification by flash column chromatography [SiO_2, *n*-pentane/Et_2O 20:1 v/v, R_f = 0.60 (*n*-pentane/Et_2O 20:1 v/v)] gave the title compound **3db** (31.4 mg, 106 µmol, 53%) as a reddish-brown oil. Absolute configuration assigned by analogy.

^{1}**H-NMR** (500 MHz, $CDCl_3$) *δ* = 7.42 – 7.38 (m, 2H), 7.31 – 7.27 (m, 3H), 7.25 – 7.21 (m, 2H), 6.89 – 6.82 (m, 2H), 5.89 (ddd, *J* = 16.9, 10.0, 6.1 Hz, 1H), 5.43 (dt, *J* = 17.0, 1.5 Hz, 1H), 5.18 (dt, *J* = 10.0, 1.4 Hz, 1H), 4.28 – 4.07 (m, 2H), 3.65 – 3.55 (m, 1H), 2.22 – 1.99 (m, 2H) ppm.

^{13}**C-NMR** (126 MHz, $CDCl_3$) *δ* = 157.7, 137.3, 131.8, 129.5, 128.4, 128.1, 125.7, 123.6, 116.0, 116.0, 89.3, 84.3, 65.9, 34.9, 33.0 ppm.

HR-MS ($C_{19}H_{18}ClO^+$; $[M+H]^+$, pos. APCI): calcd: 297.1049, found: 297.1041.

HPLC (OD-3, λ = 228 nm, *n*-heptane:IPA = 99.5:0.5, 0.5 mL/min): τ_R = 6.0 min (major) and 6.6 min (minor), 93% *ee*.

$[\alpha]_D^{20}$ = −0.900 (c = 1.00 g (100 mL)$^{-1}$, $CHCl_3$).

(*S*)-1-Bromo-4-((3-(phenylethynyl)pent-4-en-1-yl)oxy)benzene (3eb)

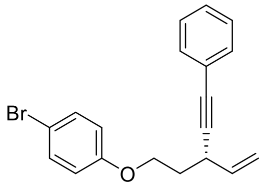

3eb

3eb was synthesized from 1-bromo-4-(penta-3,4-dien-1-yloxy)benzene (36.0 µL, 47.8 mg, 200 µmol, 1.00 equiv) and ethynylbenzene (22.0 µL, 20.4 mg, 200 µmol, 1.00 equiv) according to **GP 4**. Purification by flash column chromatography [SiO_2, *n*-pentane/Et_2O 50:1 v/v, R_f = 0.45 (*n*-pentane/Et_2O 50:1 v/v)] gave the title compound **3eb** (43.8 mg, 128 µmol, 64%) as an orange oil. Absolute configuration assigned by analogy.

^{1}**H-NMR** (400 MHz, $CDCl_3$) *δ* = 7.42 – 7.34 (m, 4H), 7.32 – 7.27 (m, 3H), 6.84 – 6.78 (m, 2H), 5.89 (ddd, *J* = 17.0, 10.0, 6.1 Hz, 1H), 5.42 (dt, *J* = 17.0, 1.5 Hz, 1H), 5.18 (dt, *J* = 10.0, 1.4 Hz, 1H), 4.22 – 4.07 (m, 2H), 3.59 (dtt, *J* = 8.7, 5.9, 1.4 Hz, 1H), 2.19 – 1.99 (m, 2H) ppm.

^{13}**C-NMR** (101 MHz, $CDCl_3$) *δ* = 158.2, 137.3, 132.4, 131.8, 128.4, 128.1, 123.6, 116.6, 116.0, 113.0, 89.3, 84.3, 65.9, 34.9, 33.0 ppm.

HR-MS ($C_{19}H_{18}BrO^+$; $[M+H]^+$, pos. APCI): calcd: 341.0536, found: 341.0542.

HPLC (OD-3, λ = 228 nm, *n*-heptane:IPA = 99.5:0.5, 0.5 mL/min): τ_R = 6.3 min (major) and 7.3 min (minor), 96% *ee*.

$[\alpha]_D^{20}$ = −1.00 (c = 1.00 g (100 mL)$^{-1}$, $CHCl_3$).

(S)-2-((3-(Phenylethynyl)pent-4-en-1-yl)oxy)benzonitrile (3fb)

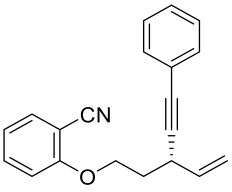

3fb

3fb was synthesized from 2-(penta-3,4-dien-1-yloxy)benzonitrile (34.0 µL, 37.0 mg, 200 µmol, 1.00 equiv) and ethynylbenzene (22.0 µL, 20.4 mg, 200 µmol, 1.00 equiv) according to **GP 4** with 17 h at 80 °C. Purification by flash column chromatography [SiO_2, *n*-pentane/Et_2O 4:1 v/v, R_f = 0.40 (*n*-pentane/Et_2O 4:1 v/v)] gave the title compound **3fb** (26.1 mg, 91.0 µmol, 45%) as an orange oil. Absolute configuration assigned by analogy.

^{1}H-NMR (400 MHz, $CDCl_3$) δ = 7.59 – 7.48 (m, 2H), 7.43 – 7.39 (m, 2H), 7.31 – 7.27 (m, 3H), 7.03 – 6.98 (m, 2H), 5.92 (ddd, J = 17.0, 10.0, 6.1 Hz, 1H), 5.45 (dt, J = 17.0, 1.5 Hz, 1H), 5.20 (dt, J = 10.0, 1.4 Hz, 1H), 4.40 – 4.21 (m, 2H), 3.74 – 3.63 (m, 1H), 2.31 – 2.05 (m, 2H) ppm.

^{13}C-NMR (101 MHz, $CDCl_3$) δ = 160.8, 137.1, 134.4, 134.0, 131.8, 128.4, 128.1, 123.5, 121.0, 116.5, 116.2, 112.5, 102.4, 89.1, 84.5, 66.7, 34.6, 32.9 ppm.

HR-MS ($C_{20}H_{18}NO^+$; $[M+H]^+$, pos. APCI): calcd: 288.1383, found: 288.1384.

HPLC (AD-3, λ = 242 nm, *n*-heptane:IPA = 99:1, 0.5 mL/min): τ_R = 9.7 min (minor) and 10.4 min (major), 91% *ee*.

$[\alpha]_D^{20}$ = +10.6 (c = 310 mg (100 mL)$^{-1}$, $CHCl_3$).

(S)-2-(4-(Phenylethynyl)hex-5-en-1-yl)isoindoline-1,3-dione (3gb)

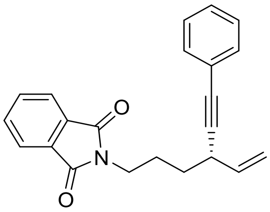

3gb

3gb was synthesized from 2-(hexa-4,5-dien-1-yl)isoindoline-1,3-dione (45.5 mg, 200 µmol, 1.00 equiv) and ethynylbenzene (22.0 µL, 20.4 mg, 200 µmol, 1.00 equiv) according to **GP 4**. Purification by flash column chromatography [SiO_2, *n*-pentane/Et_2O 4:1 v/v, R_f = 0.25 (*n*-pentane/Et_2O 4:1 v/v)] gave the title compound **3gb** (52.5 mg, 159 µmol, 80%) as an orange oil. Absolute configuration assigned by analogy.

^{1}H-NMR (400 MHz, $CDCl_3$) δ = 7.89 – 7.80 (m, 2H), 7.74 – 7.67 (m, 2H), 7.44 – 7.36 (m, 2H), 7.29 – 7.26 (m, 3H), 5.81 (ddd, J = 16.9, 10.0, 6.1 Hz, 1H), 5.36 (dt, J = 17.0, 1.5 Hz, 1H), 5.12 (dt, J = 10.0, 1.4 Hz, 1H), 3.81 – 3.68 (m, 2H), 3.40 – 3.29 (m, 1H), 2.00 – 1.59 (m, 4H) ppm.

^{13}C-NMR (101 MHz, $CDCl_3$) δ = 168.6, 137.6, 134.0, 132.3, 131.8, 128.3, 127.9, 123.7, 123.4, 115.7, 89.8, 84.2, 37.9, 35.8, 32.6, 26.3 ppm.

HR-MS ($C_{22}H_{20}NO_2^+$; $[M+H]^+$, pos. APCI): calcd: 330.1489, found: 330.1495.

HPLC (AD-3, λ = 228 nm, *n*-heptane:IPA = 95:5, 0.5 mL/min): τ_R = 12.8 min (minor) and 13.4 min (major), 93% *ee*.

$[\alpha]_D^{20}$ = +35.6 (c = 1.00 g (100 mL)$^{-1}$, $CHCl_3$).

(*S*)-Tri*iso*propyl((4-methyl-4-(phenylethynyl)hex-5-en-1-yl)oxy)silane (3hb)

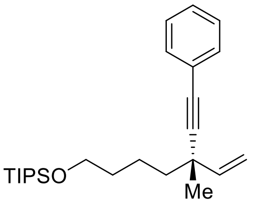

3hb

3hb was synthesized from triisopropyl((4-methylhexa-4,5-dien-1-yl)oxy)silane (61.0 µL, 53.7 mg, 200 µmol, 1.00 equiv) and ethynylbenzene (22.0 µL, 20.4 mg, 200 µmol, 1.00 equiv) according to **GP 4**. Purification by flash column chromatography [SiO_2, *n*-pentane/Et_2O 50:1 v/v, R_f = 0.55 (*n*-pentane/Et_2O 50:1 v/v)] gave the title compound **3hb** (52.6 mg, 142 µmol, 71%) as a light-yellow oil. Absolute configuration assigned by analogy.

^{1}H-NMR (400 MHz, $CDCl_3$) δ = 7.44 – 7.39 (m, 2H), 7.31 – 7.24 (m, 3H), 5.74 (dd, *J* = 17.0, 10.1 Hz, 1H), 5.41 (dd, *J* = 17.0, 1.5 Hz, 1H), 5.07 (dd, *J* = 10.1, 1.5 Hz, 1H), 3.69 (t, *J* = 6.3 Hz, 2H), 1.54 (s, 6H), 1.36 (s, 3H), 1.08 – 1.03 (m, 21H) ppm.

^{13}C-NMR (101 MHz, $CDCl_3$) δ = 143.7, 131.8, 128.3, 127.7, 124.1, 113.1, 93.8, 84.0, 63.5, 42.2, 39.6, 33.5, 28.3, 21.9, 18.2, 12.2 ppm.

HR-MS ($C_{25}H_{41}OSi^+$; $[M+H]^+$, pos. APCI): calcd: 385.2921, found: 385.2915.

HPLC (AD-3, λ = 240 nm, *n*-heptane:EtOH = 99.5:0.5, 0.3 mL/min): τ_R = 28.8 min (major) and 30.0 min (minor), 47% *ee*. (Enantiomeric ratio was determined after removal of the TIPS group with TBAF (1.46 equiv) in THF (0.10 M, rt, 3h).)

(*S*)-3-(Phenylethynyl)pent-4-en-1-yl 4-(*N*,*N*-dipropylsulfamoyl)benzoate (3ib)

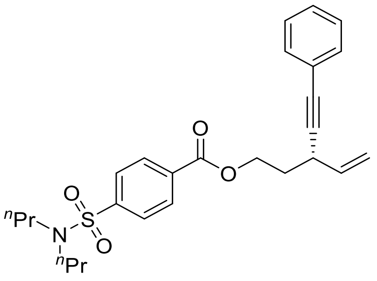

3ib

3ib was synthesized from **1i** (62.0 µL, 70.3 mg, 200 µmol, 1.00 equiv) and ethynylbenzene (22.0 µL, 20.4 mg, 200 µmol, 1.00 equiv) according to **GP 4** with 17 h at 80 °C. Purification by flash column chromatography [SiO_2, *n*-pentane/Et_2O 4:1 v/v, R_f = 0.30 (*n*-pentane/Et_2O 4:1 v/v)] gave the title compound **3ib** (58.1 mg, 128 µmol, 64%) as a yellow oil. Absolute configuration assigned by analogy.

^{1}H-NMR (400 MHz, $CDCl_3$) δ = 8.21 – 8.12 (m, 2H), 7.88 – 7.80 (m, 2H), 7.46 – 7.37 (m, 2H), 7.34 – 7.25 (m, 3H), 5.90 (ddd, *J* = 17.0, 10.0, 6.0 Hz, 1H), 5.45 (dt, *J* = 16.9, 1.4 Hz, 1H), 5.21 (dt, *J* = 10.0, 1.3 Hz, 1H), 4.63 – 4.51 (m, 2H), 3.14 – 3.05 (m, 5H), 2.21 – 2.03 (m, 2H), 1.55 (m, 4H), 0.87 (t, *J* = 7.4 Hz, 6H) ppm.

^{13}C-NMR (101 MHz, $CDCl_3$) δ = 165.4, 144.5, 137.0, 133.7, 131.8, 130.4, 128.4, 128.2, 127.2, 123.5, 116.3, 88.9, 84.6, 63.5, 50.1, 33.3, 29.9, 22.1, 11.3 ppm.

HR-MS ($C_{26}H_{31}NNaO_4S^+$; [M+Na]$^+$, pos. ESI): calcd: 476.1866, found: 476.1868.

HPLC (AD-3, *n*-heptane:EtOH = 90:10, 0.5 mL/min): τ_R = 12.3 min (major) and 14.6 min (minor), 80% *ee*.

$[\alpha]_D^{20}$ = −6.00 (c = 1.00 g (100 mL)$^{-1}$, $CHCl_3$).

(*R*)-2,5,7,8-Tetramethyl-6-(((*S*)-3-(phenylethynyl)pent-4-en-1-yl)oxy)-2-((4*R*,8*R*)-4,8,12-trimethyltridecyl)chromane (3jb)

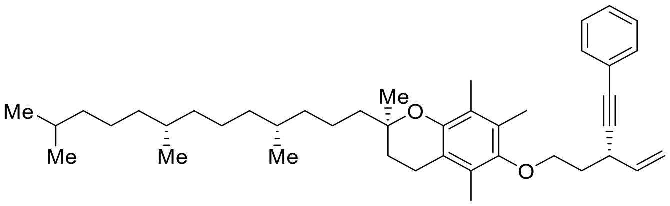

3jb

3jb was synthesized from **1j** (119.8 mg, 200 µmol, 1.00 equiv) and ethynylbenzene (22.0 µL, 20.4 mg, 200 µmol, 1.00 equiv) according to **GP 4**. Purification by flash column chromatography [SiO_2, *n*-pentane/Et_2O 50:1 v/v, R_f = 0.45 (*n*-pentane/Et_2O 50:1 v/v)] gave the title compound **3jb** (105 mg, 175 µmol, 88%) as an orange oil. Absolute configuration assigned by analogy.

^{1}**H-NMR** (500 MHz, $CDCl_3$) δ = 7.45 – 7.39 (m, 2H), 7.32 – 7.26 (m, 3H), 5.94 (ddd, *J* = 16.9, 10.0, 6.0 Hz, 1H), 5.45 (dt, *J* = 17.0, 1.5 Hz, 1H), 5.18 (dt, *J* = 10.0, 1.4 Hz, 1H), 3.99 – 3.76 (m, 2H), 3.72 (dq, *J* = 9.9, 6.4 Hz, 1H), 2.57 (t, *J* = 6.8 Hz, 2H), 2.25 – 1.96 (m, 2H), 1.86 – 1.70 (m, 2H), 1.23 (s, 3H), 2.21 (s, 3H), 2.17 (s, 3H), 2.08 (s, 3H), 1.52 – 0.99 (m, 21H), 0.90 – 0.80 (m, 12H) ppm.

^{13}**C-NMR** (126 MHz, $CDCl_3$) δ = 148.3, 147.9, 137.8, 131.7, 128.4, 127.9, 126.0, 123.8, 123.0, 117.7, 115.6, 89.9, 84.2, 74.9, 70.3, 40.2, 39.5, 37.6, 37.6, 37.4, 36.1, 33.1, 33.0, 32.9, 31.5, 29.9, 28.1, 25.0, 24.6, 24.0, 22.9, 22.8, 21.2, 20.8, 19.9, 19.8, 12.9, 12.0, 11.9 ppm.

HR-MS ($C_{42}H_{63}O_2^+$; $[M+H]^+$, pos. APCI): calcd: 599.4823, found: 599.4822.

$[\alpha]_D^{20}$ = +16.2 (c = 1.00 g (100 mL)$^{-1}$, $CHCl_3$).

(*S*)-2-(3-(2-(4-Bromophenoxy)ethyl)pent-4-en-1-yn-1-yl)aniline (3ef)

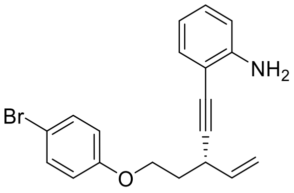

3ef

3ef was synthesized from 1-bromo-4-(penta-3,4-dien-1-yloxy)benzene (33.6 µL, 47.8 mg, 200 µmol, 1.00 equiv) and 2-ethynylaniline (22.5 µL, 23.4 mg, 200 µmol, 1.00 equiv) according to **GP 4**. Purification by flash column chromatography [SiO_2, *n*-pentane/Et_2O 9:1 to 4:1 v/v, R_f = 0.28 (*n*-pentane/Et_2O 4:1 v/v)] gave the title compound **3ef** (35.7 mg, 100 µmol, 50%) as a colorless oil. Absolute configuration assigned by analogy.

^{1}**H-NMR** (400 MHz, $CDCl_3$) δ = 7.40 – 7.33 (m, 2H), 7.27 – 7.22 (m, 1H), 7.10 (ddd, *J* = 8.2, 7.4, 1.6 Hz, 1H), 6.83 – 6.77 (m, 2H), 6.71 – 6.64 (m, 2H), 5.91 (ddd, *J* = 17.0, 10.0, 6.2 Hz, 1H), 5.43 (ddd, *J* = 16.9, 1.4, 1.4 Hz, 1H), 5.18 (ddd, *J* = 10.0, 1.4, 1.4 Hz, 1H), 4.23 – 4.07 (m, 4H), 3.66 (dtt, *J* = 8.9, 6.0, 1.5 Hz, 1H), 2.21 – 2.00 (m, 2H) ppm.

^{13}**C-NMR** (101 MHz, $CDCl_3$) δ = 158.1, 147.8, 137.3, 132.4, 132.2, 129.4, 118.0, 116.5, 116.0, 114.3, 113.0, 108.3, 94.6, 80.8, 65.8, 34.9, 33.2 ppm.

HR-MS ($C_{19}H_{17}NOBr^-$; $[M-H]^-$, neg. ESI): calcd: 354.0499, found: 354.0493.

HPLC (AD-3, λ = 218 nm, *n*-heptane:IPA = 90:10, 0.5 mL/min): τ_R = 12.9 min (major) and 13.5 min (minor), 95% *ee*.

$[\alpha]_D^{21}$ = −5.25 (c = 629 mg (100 mL)$^{-1}$, $CHCl_3$).

(*S*)-*N*-benzyl-2-(3-phenethylpent-4-en-1-yn-1-yl)aniline (3kq)

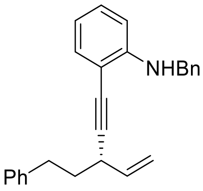

3kq

3kq was synthesized from penta-3,4-dien-1-ylbenzene (31.9 µL, 28.8 mg, 200 µmol, 1.00 equiv) and *N*-benzyl-2-ethynylaniline (37.7 µL, 41.5 mg, 200 µmol, 1.00 equiv) according to **GP 4**. Purification by flash column chromatography [SiO_2, *n*-pentane/Et_2O 49:1 to 4:1 v/v, R_f = 0.29 (*n*-pentane/Et_2O 49:1 v/v)] gave the title compound **3kq** (44.5 mg, 127 µmol, 63%) as a colorless oil. Absolute configuration assigned by analogy.

^{1}H-NMR (400 MHz, $CDCl_3$) δ = 7.43 – 7.27 (m, 8H), 7.24 – 7.15 (m, 4H), 6.67 (ddd, *J* = 7.5, 7.5, 1.1 Hz, 1H), 6.61 (dd, *J* = 8.2, 1.0 Hz, 1H), 5.87 (ddd, *J* = 16.9, 10.0, 6.1 Hz, 1H), 5.38 (dt, *J* = 17.0, 1.5 Hz, 1H), 5.13 (dt, *J* = 10.0, 1.4 Hz, 1H), 5.04 (s, 1H), 4.44 (d, *J* = 3.0 Hz, 2H), 3.36 (dtt, *J* = 7.6, 6.0, 1.5 Hz, 1H), 2.91 – 2.71 (m, 2H), 2.02 – 1.88 (m, 2H) ppm.

^{13}C-NMR (101 MHz, $CDCl_3$) δ = 148.8, 141.7, 139.2, 137.8, 132.1, 129.5, 128.7, 128.6, 128.5, 127.3, 126.0, 116.6, 115.5, 109.8, 108.3, 96.1, 80.7, 47.9, 37.2, 35.9, 33.4 ppm.

HR-MS ($C_{26}H_{26}N^+$; $[M+H]^+$, pos. ESI): calcd: 352.2026, found: 352.2068.

HPLC (LC-3, λ = 218 nm, *n*-heptane:EtOH = 99:1, 0.5 mL/min): τ_R = 12.3 min (major) and 13.0 min (minor), 91% *ee*.

$[\alpha]_D^{21}$ = +0.575 (c = 652 mg $(100\ mL)^{-1}$, $CHCl_3$).

Unsuccessful Substrates:

Products that could not be obtained using **GP 4**, or only in small quantities.

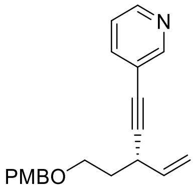

S51

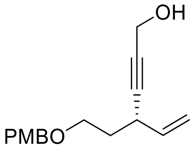

S52

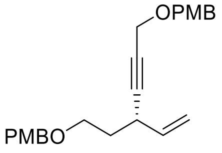

S53

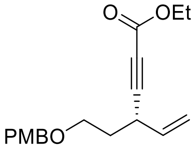

S1

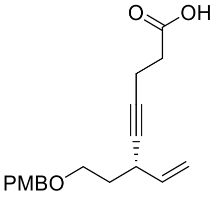

S54

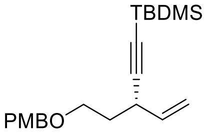

S56

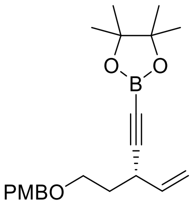

S57

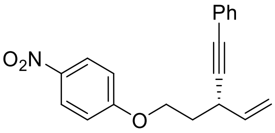

S58

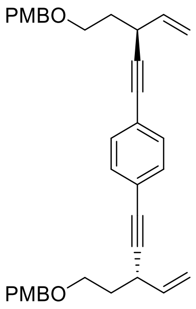

S59
(with *p*-diethynylbenzene)

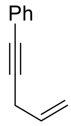

S60
(with 5.00 equiv propadiene in DCE)

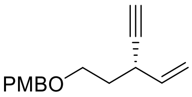

or

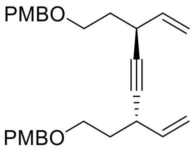

6 **S61**
(with 1 atm acetylene)

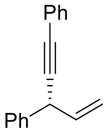

S62

5 Mechanistic and Control Experiments

5.1 Scale-up Experiment

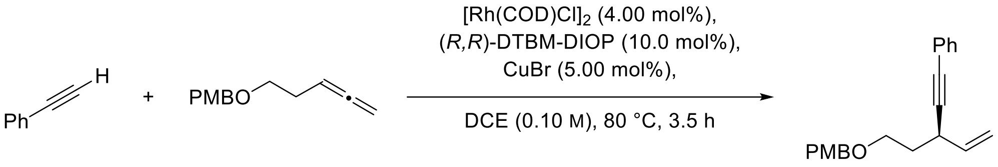

1a **2b** **ent-3ab**

In a SCHLENK-tube [Rh(COD)Cl]$_2$ (19.7 mg, 40.0 µmol, 4.00 mol%), (*R,R*)-DTBM-DIOP (107 mg, 100 µmol. 10.0 mol%) and CuBr (7.17 mg, 50.0 µmol, 5.00 mol%) were dissolved in DCE (10 mL, 0.1 M). The mixture was stirred for 20 min. The allene (1.00 mmol, 1.00 equiv) was added to the solution, followed by the addition of the alkyne (1.00 mmol, 1.00 equiv). The reaction mixture was heated to 80 °C for 3.5 h. After cooling to rt, the solvent was removed under reduced pressure. Purification by flash column chromatography [SiO$_2$, *n*-pentane/Et$_2$O 29:1 to 19:1 v/v, R_f = 0.45 (*n*-pentane/EtOAc 19:1 v/v)] gave the title compound **ent-3ab** (185 mg, 604 µmol, 60%) as a colorless oil. Absolute configuration assigned by analogy.

The analytical data were consistent with those reported for **3ab**.

HPLC (AD-3, λ = 250 nm, *n*-heptane:IPA = 99:1, 0.5 mL/min): τ_R = 6.7 min (major) and 7.0 min (minor), 93% *ee*.

5.2 Deuterium Labeling Experiments

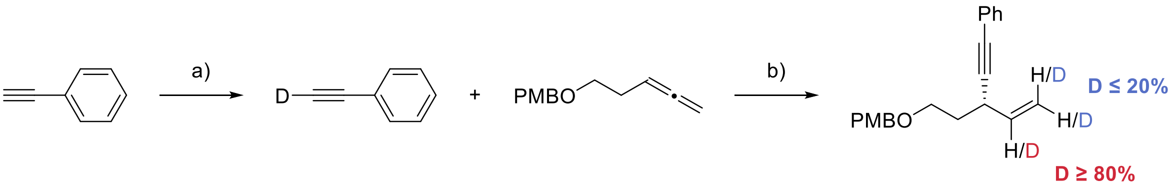

2b **D-2b** **1a** **D-3ab**

a) *n*-BuLi (2.50 M in hexane, 4.00 mL, 10.0 mmol, 2.00 equiv) was added dropwise to a solution of phenylacetylene (549 µL, 511 mg, 5.00 mmol, 1.00 equiv) in *n*-hexane (10 mL) at −78 °C. The solution was stirred for 30 min. Then, it was allowed to warm up to rt, D_2O (4.00 mL, 4.42 g, 22.1 mmol, 44.0 equiv) was added and the mixture was stirred overnight. The layers were separated and the organic phase was extracted with CH_2Cl_2 (3 × 10 mL). The combined organic extracts were dried over Na_2SO_4, the solvent was removed under reduced pressure and the target product **D-2b** (419 mg, 4.06 mmol, 81%, 98% deuteriation) was obtained as a colorless oil and used in the next step without further purification.

This procedure is based on a previously reported one.[27] Analytical data are in accordance with literature.[28]

b) **D-3ab** was synthesized according to **GP 4** from (ethynyl-d)benzene (20.5 µL, 20.6 mg, 200 µmol, 1.00 equiv) and 1-methoxy-4-((penta-3,4-dien-1-yloxy)methyl)benzene (40.0 µL, 40.9 mg, 200 µmol, 1.00 equiv). Purification by flash column chromatography [SiO_2, *n*-pentane/Et_2O 9:1 v/v, R_f = 0.35 (*n*-pentane/Et_2O 9:1 v/v)] gave the title compound **D-3ab** (43.9 mg, 143 µmol, 71%) as a colorless oil.

Absolute configuration assigned by analogy. The ^{1}H-NMR and ^{13}C-NMR shifts are analogous to those of substrate **3ab**.

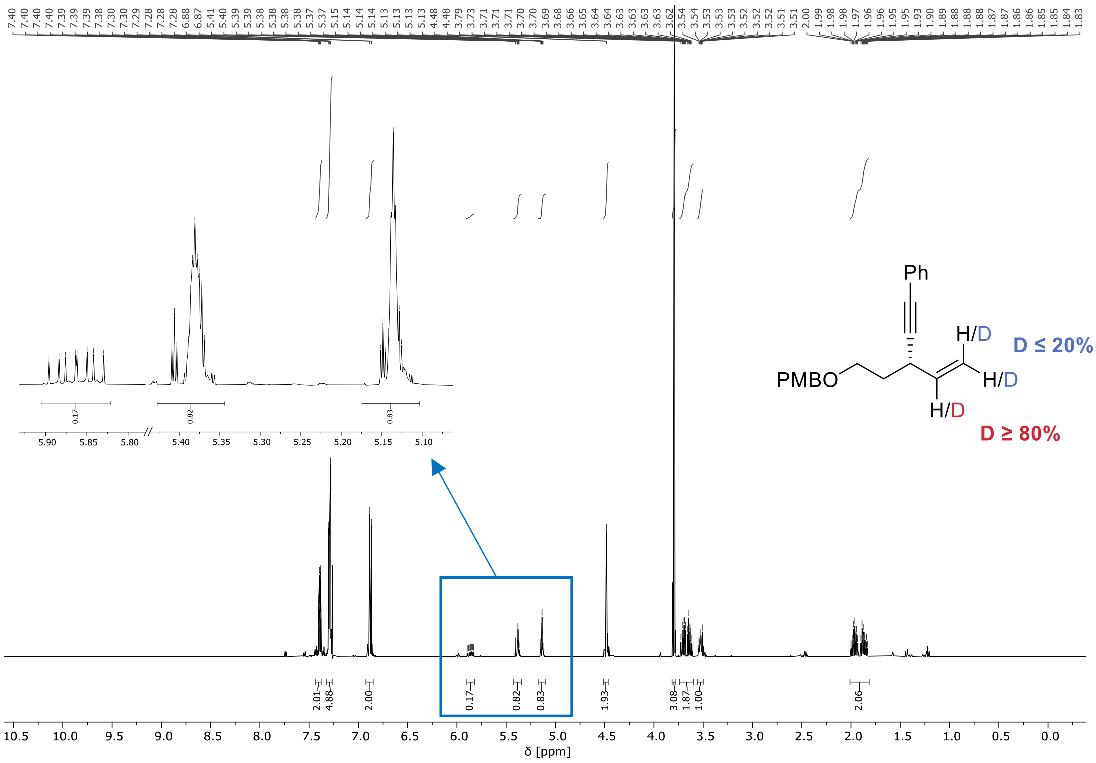

^{2}H-NMR (77 MHz, $CDCl_3$) δ = 5.88, 5.40, 5.15 ppm.

HPLC (AD-3, λ = 250 nm, *n*-heptane:IPA = 99:1, 0.5 mL/min): τ_R = 6.8 min (minor) and 7.1 min (major), 82% *ee*.

HR-MS ($C_{21}H_{22}DO_2^+$; $[M+H]^+$, pos. APCI): calcd: 308.1755, found: 308.1752.

5.3 FT-IR Spectroscopy

The reaction course of the enantioselective addition of alkyne **2a** to allene **1a** in DCE (0.1 M) at 80 °C with CuBr (5.00 mol%) and (*S,S*)-DTBM-DIOP (10.0 mol%) was investigated using 3D IR spectroscopy. For this purpose, time was plotted against the wave number and the intensity of the vibrations. The decrease in the alkyne could be observed based on the C-H stretching vibration at 3310 cm^{-1}. The decrease in the alkenes could be determined from the C-C stretching vibration at a wavenumber of 1971 cm^{-1}. Product formation could not be tracked due to signal overlap.

The spectra showed an exponential decline of both substrates over time. In addition, both substrates were completely converted in the reaction after approximately 3 h. This prompted a reduction in the

reaction time from 17 h to 3.5 h, which, after experimental verification, resulted in unchanged yields and enantioselectivities.

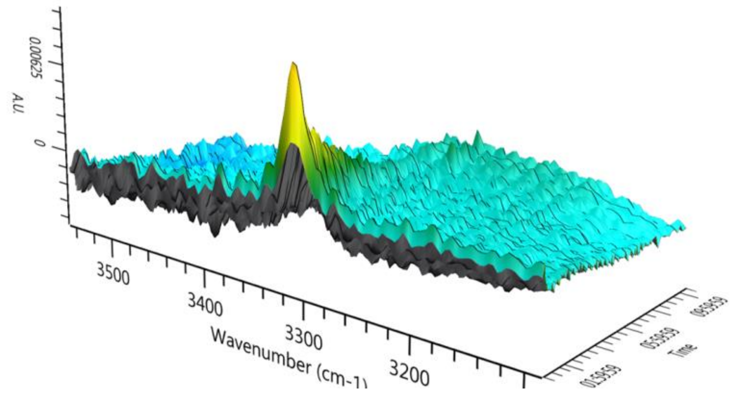

Figure 1: 3D IR spectrum of the C-H stretching vibration of the alkyne.

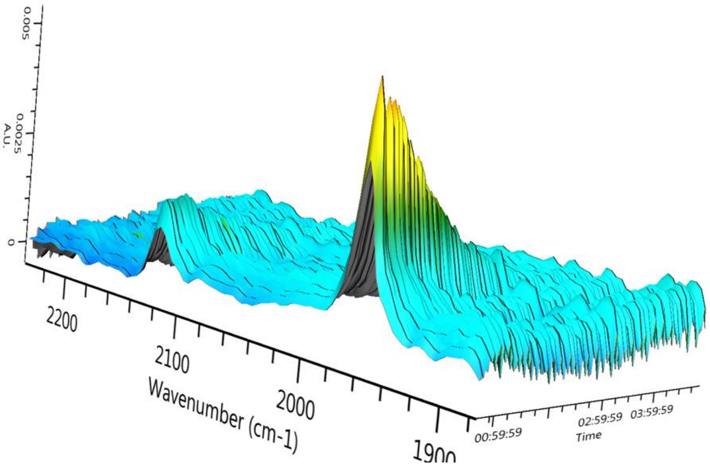

Figure 2: 3D IR spectrum of the C-C stretching vibration of the allene.

5.4 Control Experiments

Catalysis without Catalysts or Ligand

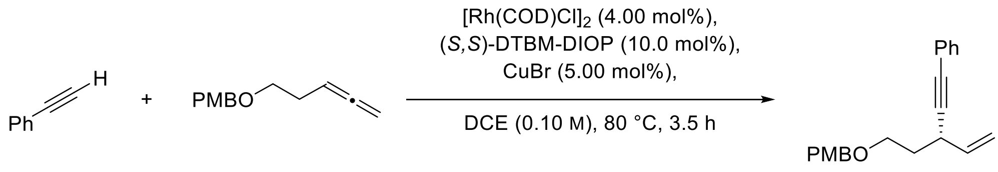

2b **1a** **3ab**

entry[a]	variation	yield	*ee*
1	w/o (*S,S*)-DTBM-DIOP (10.0 mol%)	0%	-
2	w/o CuBr (5.00 mol%)	0%	-
3	w/o $[Rh(COD)Cl]_2$ (4.00 mol%)	0%	-

[a] Reaction conditions (unless otherwise specified): **1a** (100 µmol), **2b** (100 µmol), Rh source (4.00 mol%), ligand (10.0 mol%), Cu source (5.00 mol%) in DCE (1.0 mL), 80 °C, 3.5 h. NMR yield of the product was determined by ^{1}H NMR spectroscopy using DBM as internal standard. Enantiomeric ratio determined by chiral HPLC.

Catalysis without Allene

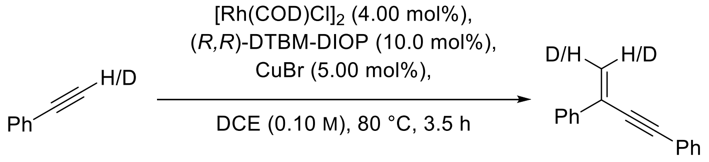

2b or D-2b **10 or D-10**

entry[a]	alkyne	variation	yield
1	**2b**	with CuBr (5.00 mol%)	87%
2	**2b**	w/o CuBr	97%
3	**D-2b**	with CuBr (5.00 mol%)	47%
4	**D-2b**	w/o CuBr	48%

[a] Reaction conditions (unless otherwise specified): **2b** (100 µmol), Rh source (4.00 mol%), ligand (10.0 mol%), Cu source (5.00 mol%) in DCE (1.0 mL), 80 °C, 3.5 h. NMR yield of the product was determined by ^{1}H NMR spectroscopy using DBM as internal standard.

Analytical data from **10**:

^{1}H-NMR (300 MHz, $CDCl_3$) δ = 7.76 – 7.69 (m, 2H), 7.57 – 7.50 (m, 2H), 7.43 – 7.30 (m, 6H), 5.99 (d, *J* = 1.1 Hz, 1H), 5.76 (d, *J* = 1.1 Hz, 1H) ppm.

HR-MS ($C_{16}H_{17}^+$; $[M+H]^+$, pos. APCI): calcd: 205.1012, found: 205.1009.

Catalysis Using Product 3ab as the Starting Material

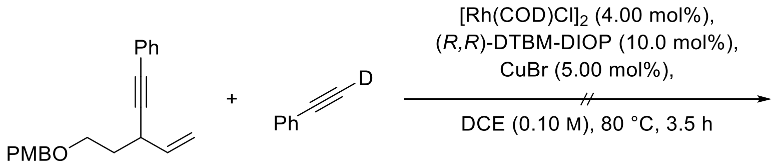

***rac*-3ab** **D-2b**

6 Follow-Up Chemistry

6.1 Absolute Configuration Assignment

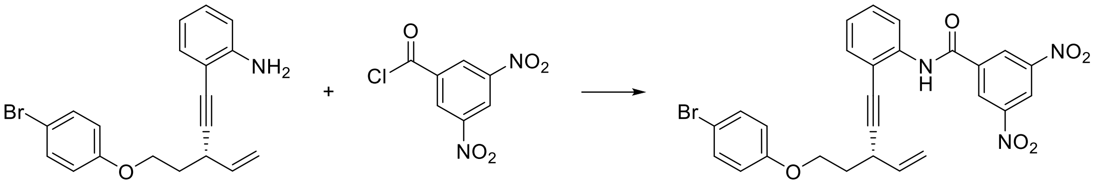

3ef **S63** **4**

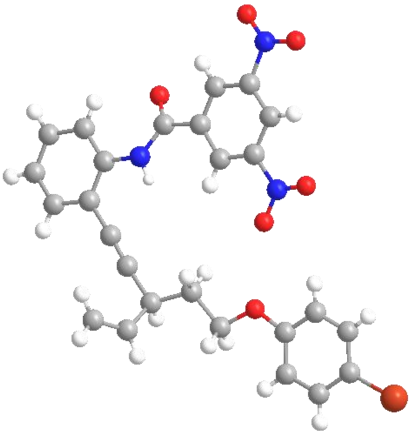

A solution of 3,5-dinitrobenzoyl chloride (28.8 mg, 125 µmol, 1.25 equiv) in THF (5.0 mL) was added to a solution of **3ef** (35.7 mg, 100 µmol, 1.00 equiv) in THF (10 mL) at 0 °C. The mixture was stirred at 0 °C for 2 h and at rt for 22 h. The solution was poured into ice and a saturated aqueous K_2CO_3 solution (15 mL) was added. The mixture was extracted with CH_2Cl_2 (3 × 15 mL), the combined organic extracts were dried over Na_2SO_4 and the solvent was removed under reduced pressure. The residue was purified by column chromatography chromatography [SiO_2, *n*-pentane/Et_2O 4:1 to 3:2 v/v, R_f = 0.25 (*n*-pentane/Et_2O 7:3 v/v)] gave **4** (47.7 mg, 86.7 µmol, 87%) as a yellow oil, which crystallized into yellow crystals from *n*-pentane/Et_2O.

This procedure is based on a previously reported one.[29]

^{1}H-NMR (500 MHz, $CDCl_3$) δ = 9.10 (t, *J* = 2.1 Hz, 1H), 8.96 (d, *J* = 2.1 Hz, 2H), 8.88 (s, 1H), 8.51 (d, *J* = 8.3 Hz, 1H), 7.48 (dd, *J* = 7.7, 1.4 Hz, 1H), 7.41 (td, *J* = 7.8, 1.2 Hz, 1H), 7.25 – 7.21 (m, 2H), 7.16 (td, *J* = 7.6, 1.2 Hz, 1H), 6.66 – 6.61 (m, 2H), 5.95 (ddd, *J* = 16.9, 10.0, 6.4 Hz, 1H), 5.42 (ddd, *J* = 16.9, 1.3, 1.3 Hz, 1H), 5.18 (ddd, *J* = 10.0, 1.2, 1.2 Hz, 1H), 4.17 – 4.00 (m, 2H), 3.83 – 3.76 (m, 1H), 2.27 – 2.09 (m, 2H) ppm.

^{13}C-NMR (126 MHz, $CDCl_3$) δ = 160.1, 157.7, 148.7, 138.0, 137.8, 136.5, 132.3, 132.0, 129.7, 127.0, 124.9, 121.4, 119.4, 116.7, 116.2, 113.2, 113.1, 98.8, 79.0, 65.6, 34.7, 33.4 ppm.

HR-MS ($C_{26}H_{19}BrN_3O_6^-$; $[M-H]^-$, neg. ESI): calcd: 548.0463, found: 550.0442.

HPLC (OD-3, λ = 250 nm, *n*-heptane:EtOH = 70:30, 0.5 mL/min): τ_R = 17.1 min (minor) and 18.7 min (major), 94% *ee*.

$[\alpha]_D^{21}$ = +24.7 (c = 1.36 g (100 mL)$^{-1}$, $CHCl_3$).

X-ray and derived ORTEP structures provided by Dr. Burkhard Butschke, Krossing Group, Inorganic Chemistry Department, Albert-Ludwigs-University Freiburg.

6.2 Deprotection of 3ap and Azide-Alkyne Cycloaddition

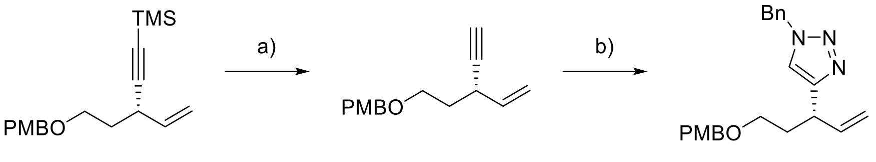

3ap **5** **6**

a) Synthesis of **5** according to **GP 1** by the addition of K_2CO_3 (504 g, 3.65 mmol, 2.00 equiv) to a solution of **3ap** (552 g, 1.82 mmol, 1.00 equiv) in MeOH (3.0 mL). Purification by flash column chromatography [SiO_2, *n*-pentane/Et_2O 19:1 to 9:1 v/v, R_f = 0.35 (*n*-pentane/Et_2O 19:1 v/v)] gave the title compound **5** (369 mg, 1.60 mmol, 88%) as a yellow oil.

^{1}H-NMR (700 MHz, $CDCl_3$) *δ* = 7.28 – 7.23 (m, 3H), 6.90 – 6.85 (m, 2H), 5.78 (ddd, *J* = 16.9, 10.1, 6.2 Hz, 1H), 5.33 (ddd, *J* = 17.0, 1.5, 1.5 Hz, 1H), 5.11 (ddd, *J* = 10.1, 1.4, 1.4 Hz, 1H), 4.53 – 4.37 (m, 2H), 3.81 (s, 3H), 3.68 – 3.56 (m, 2H), 3.34 – 3.29 (m, 1H), 2.20 (d, *J* = 2.5 Hz, 1H), 1.96 – 1.75 (m, 3H) ppm.

^{13}C-NMR (176 MHz, $CDCl_3$) *δ* = 159.3, 137.3, 130.7, 129.4, 115.6, 113.9, 84.5, 72.8, 71.4, 67.3, 55.4, 35.3, 32.1 ppm.

HR-MS ($C_{15}H_{22}NO_2^+$; $[M+NH_4]^+$, pos. APCI): calcd: 248.1645, found: 248.1647.

HPLC (LC-3, λ = 218 nm, *n*-heptane:EtOH = 99:1, 0.5 mL/min): τ_R = 13.6 min (major) and 14.6 min (minor), 90% *ee*.

$[\alpha]_D^{21}$ = −3.64 (c = 557 mg (100 mL)$^{-1}$, $CHCl_3$).

b) To a mixture of terminal alkyne **5** (23.0 mg, 100 µmmol, 1.00 equiv) and benzyl azide (15.0 µL, 16.0 mg, 120 µmol, 1.20 equiv) in *t*-BuOH/H_2O (3:1, 2.0 mL) were added $CuSO_4 \times 5\,H_2O$ (10.0 mg, 40.0 µmol, 40.0 mol%) and sodium ascorbate (15.8 mg, 80.0 µmol, 80.0 mol%) at rt. The mixture was stirred for 2 h and diluted with EtOAc (3.0 mL) and an aqueous saturated NH_4Cl solution (3.0 mL). The layers were separated and the aqueous phase was extracted with EtOAc (3 × 15 mL). The combined organic extracts were washed with brine, dried over $MgSO_4$ and concentrated under reduced pressure. The residue was purified by column chromatography chromatography [SiO_2, *n*-pentane/Et_2O 1:2 v/v, R_f = 0.27 (*n*-pentane/Et_2O 1:2 v/v)] gave triazole **6** (28.0 mg, 77.0 µmol, 77%) as a colorless oil.

^{1}**H-NMR** (500 MHz, $CDCl_3$) δ = 7.40 – 7.31 (m, 3H), 7.25 – 7.19 (m, 4H), 7.13 (s, 1H), 6.89 – 6.82 (m, 2H), 5.96 – 5.87 (m, 1H), 5.87 – 5.96 (m, 1H), 5.47 (s, 2H), 5.10 – 5.04 (m, 2H), 5.04 – 5.10 (m, 2H), 4.38 – 4.36 (m, 2H), 3.80 (s, 3H), 3.69 (dt, *J* = 7.7, 7.7 Hz, 1H), 3.56 – 3.39 (m, 2H), 2.18 – 1.93 (m, 2H) ppm.

^{13}**C-NMR** (126 MHz, $CDCl_3$) δ = 159.2, 150.6, 139.7, 134.9, 130.7, 129.4, 129.2, 128.7, 128.1, 120.7, 115.7, 113.8, 72.6, 67.5, 55.4, 54.1, 38.0, 34.4 ppm.

HR-MS ($C_{22}H_{26}N_2O_2^+$; $[M+H]^+$, pos. ESI): calcd: 364.2020, found: 364.2023.

HPLC (LC-3, λ = 218 nm, *n*-heptane:EtOH = 80:20, 0.5 mL/min): τ_R = 24.9 (major) and 28.4 min (minor), 92% *ee*.

$[\alpha]_D^{21}$ = +13.7 (c = 671 mg (100 mL)$^{-1}$, $CHCl_3$).

6.3 Indole Cyclization of 3kq

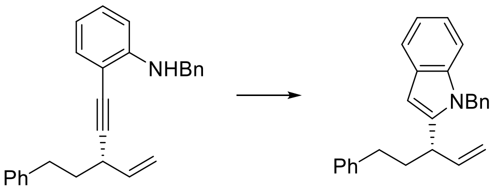

3kq **7**

A solution of enyne **3kq** (22.3 mg, 63.3 µmol, 1.00 equiv) in MeCN (1.0 mL) was added to PPh_3AuCl (1.57 mg, 3.17 µmol, 5.00 mol%) and AgOTf (814 µg, 3.17 mg, 5.00 mol%). The mixture was stirred in the dark at 90 °C for 22 h. Then, it was allowed to cool down to rt, diluted with Et_2O and filtered over celite. The filtrate was concentrated under reduced pressure and the residue was purified by column chromatography [SiO_2, *n*-pentane/Et_2O 99:1 v/v, R_f = 0.21 (*n*-pentane/Et_2O 99:1 v/v)] to afford indole **7** (15.7 mg, 44.7 µmol, 71%) as a yellow oil.

^{1}**H-NMR** (700 MHz, $CDCl_3$) δ = 7.64 – 7.61 (m, 1H), 7.26 – 7.20 (m, 8H), 7.19 – 7.16 (m, 1H), 7.14 – 7.09 (m, 4H), 7.09 – 7.06 (m, 2H), 6.92 – 6.88 (m, 2H), 6.46 (d, *J* = 0.9 Hz, 1H), 5.90 (ddd, *J* = 17.0, 10.1, 7.6 Hz, 1H), 5.27 (s, 2H), 5.06 (ddd, *J* = 10.3, 1.1, 1.1 Hz, 1H), 4.94 (ddd, *J* = 17.2, 1.3, 1.3 Hz, 1H), 3.41 (ddd, *J* = 7.3, 7.3, 6.9 Hz, 1H), 2.69 – 2.58 (m, 2H), 2.20 – 2.02 (m, 2H) ppm.

^{13}**C-NMR** (176 MHz, $CDCl_3$) δ = 142.4, 141.9, 140.4, 138.1, 137.4, 128.8, 128.5, 128.5, 128.1, 127.3, 126.0, 125.9, 121.2, 120.2, 119.7, 115.6, 109.5, 99.2, 46.5, 40.9, 36.2, 33.5 ppm.

HR-MS ($C_{26}H_{25}N^+$; $[M]^+$, pos. ESI): calcd: 351.1987, found: 351.1982.

HPLC (AD-3, λ = 218 nm, *n*-heptane:EtOH = 99:1, 0.5 mL/min): τ_R = 4.8 min (minor) and 5.5 min (major), 91% *ee*.

$[\alpha]_D^{21}$ = +112 (c = 552 mg (100 mL)$^{-1}$, $CHCl_3$).

6.4 Benzofuran Cyclization of 3ae and Hydroformylation

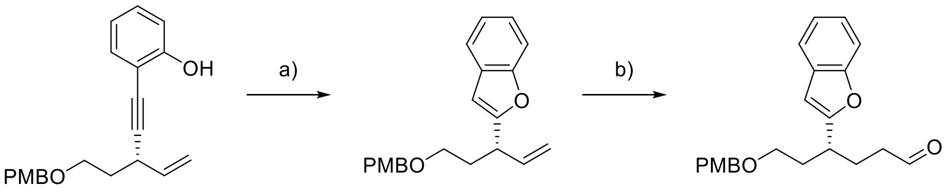

3ae **8** **9**

a) A solution of enyne **3ae** (17.9 mg, 55.5 µmmol, 1.00 equiv) in MeCN (1.0 mL) was added to CuCl (275 µg, 2.78 µmol, 5.00 mol%) and Cs_2CO_3 (906 µg, 2.78 µmol, 5.00 mol%). The mixture was stirred at 90 °C for 17 h. Then, it was allowed to cool down to rt, diluted with Et_2O and filtered over celite. The filtrate was concentrated and purified by column chromatography [SiO_2, *n*-pentane/Et_2O 9:1 v/v, R_f = 0.35 (*n*-pentane/Et_2O 9:1 v/v)] to afford benzofuran **8** (16.7 mg, 51.8 µmol, 93%) as a yellow oil.

^{1}H-NMR (400 MHz, $CDCl_3$) δ = 7.51 – 7.46 (m, 1H), 7.45 – 7.40 (m, 1H), 7.27 – 7.15 (m, 4H), 6.91 – 6.83 (m, 2H), 6.40 (dd, *J* = 0.9, 0.9 Hz, 1H), 5.95 (ddd, *J* = 17.0, 10.2, 7.9 Hz, 1H), 5.22 – 5.12 (m, 2H), 4.42 (s, 2H), 3.80 (s, 3H), 3.79 – 3.72 (m, 1H), 3.56 – 3.43 (m, 2H), 2.27 – 1.96 (m, 2H) ppm.

^{13}C-NMR (101 MHz, $CDCl_3$) δ = 160.1, 159.3, 154.8, 138.1, 130.6, 129.4, 128.8, 123.4, 122.5, 120.5, 116.5, 113.9, 111.0, 102.3, 72.7, 67.3, 55.4, 40.5, 33.2 ppm.

HR-MS ($C_{21}H_{23}NO_3^+$; $[M+H]^+$, pos. ESI): calcd: 323.1642, found: 323.1642.

HPLC (LC-3, λ = 212 nm, *n*-heptane:EtOH = 95:5, 0.5 mL/min): τ_R = 29.0 min (minor) and 41.4 min (major), 92% *ee*.

$[\alpha]_D^{21}$ = −12.1 (c = 710 mg (100 mL)$^{-1}$, $CHCl_3$). (The optical rotation was determined for **ent-8**)

b) $[Rh(CO)_2acac]_2$ (120 µg, 466 nmol, 0.500 mol%), 6-DPPon (2.60 mg, 9.31 µmol, 10.0 mol%) and benzofuran **8** (30.0 mg, 93.1 µmol, 1.00 equiv) were solved in toluene (2.0 mL) and stirred for 19 h at 70 °C and synthesis gas (CO/H_2 1:1, 10 bar). The mixture was allowed to cool down to rt, diluted with EtOAc and filtered over celite. The filtrate was concentrated under reduced pressure and purified by column chromatography [SiO_2, *n*-pentane/Et_2O 7:3 v/v, R_f = 0.28 (*n*-pentane/Et_2O 7:3 v/v)] to afford aldehyde **9** (24.4 mg, 69.2 µmol, 74%) as a colorless oil.

^{1}H-NMR (500 MHz, $CDCl_3$) δ = 9.68 (t, *J* = 1.4 Hz, 1H), 4.41 – 4.33 (m, 2H), 3.80 (s, 3H), 3.51 – 3.30 (m, 2H), 3.07 (tt, *J* = 9.1, 5.7 Hz, 1H), 2.40 (ddd, *J* = 8.1, 7.1, 1.3 Hz, 2H), 2.09 – 1.97 (m, 4H) ppm.

^{13}C-NMR (101 MHz, $CDCl_3$) δ = 201.9, 159.8, 159.3, 154.8, 130.5, 129.4, 128.6, 123.5, 122.7, 120.5, 113.8, 111.0, 103.6, 72.8, 67.5, 55.4, 41.8, 35.8, 34.0, 26.2 ppm.

HR-MS ($C_{22}H_{25}O_4^+$; $[M+H]^+$, pos. ESI): calcd: 353.1747, found: 353.1750.

$[\alpha]_D^{21}$ = +48.5 (c = 819 mg (100 mL)$^{-1}$, $CHCl_3$).

7 Literature

[1] Y. Ueda, H. Tsurugi, K. Mashima, *Angew. Chem. Int. Ed.* **2020**, *59*, 1552.

[2] Z. Lu, X.-D. Hu, H. Zhang, X.-W. Zhang, J. Cai, M. Usman, H. Cong, W.-B. Liu, *J. Am. Chem. Soc.* **2020**, *142*, 7328.

[3] B. L. Elbert, D. S. W. Lim, H. G. Gudmundsson, J. A. O'Hanlon, E. A. Anderson, *Chem. Eur. J.* **2014**, *20*, 8594.

[4] R. H. Pawle, T. E. Haas, P. Müller, S. W. Thomas III, *Chem. Sci.* **2014**, *5*, 4184.

[5] J. Bucher, T. Wurm, K. S. Nalivela, M. Rudolph, F. Rominger, A. S. K. Hashmi, *Angew. Chem. Int. Ed.* **2014**, *53*, 3854.

[6] S. Zhang, J. Li, T. Xiao, B. Yang, Y. Jiang, *Molecules* **2022**, *27*.

[7] J. E. Ross, P. C. Knipe, S. Thompson, A. D. Hamilton, *Chem. Eur. J.* **2015**, *21*, 13518.

[8] A. B. Dapkekar, S. K. Nag, G. Satyanarayana, *Adv. Synth. Catal.* **2025**, *367*.

[9] P. H. S. Paioti, K. A. Abboud, A. Aponick, *J. Am. Chem. Soc.* **2016**, *138*, 2150.

[10] X.-K. Qi, H. Zhang, Z.-T. Pan, R.-B. Liang, C.-M. Zhu, J.-H. Li, Q.-X. Tong, X.-W. Gao, L.-Z. Wu, J.-J. Zhong, *Chem. Commun.* **2019**, *55*, 10848.

[11] G. C. Senadi, J.-Q. Wang, B. S. Gore, J.-J. Wang, *Adv. Synth. Catal.* **2017**, *359*, 2747.

[12] J. Zhang, X. Huo, J. Xiao, L. Zhao, S. Ma, W. Zhang, *J. Am. Chem. Soc.* **2021**, *143*, 12622.

[13] P. A. Spreider, A. M. Haydl, M. Heinrich, B. Breit, *Angew. Chem. Int. Ed.* **2016**, *55*, 15569.

[14] A. Ďuriš, D. M. Barber, H. J. Sanganee, D. J. Dixon, *Chem. Commun.* **2013**, *49*, 2777.

[15] Y. Zhang, H.-Q. Geng, X.-F. Wu, *Chem. Eur. J.* **2021**, *27*, 17682.

[16] Y. Zhao, S. Ge, *Angew. Chem. Int. Ed.* **2021**, *60*, 2149.

[17] K. Takai, R. Kokumai, S. Toshikawa, *Synlett* **2002**, *2002*, 1164.

[18] A. Köpfer, B. Breit, *Angew. Chem. Int. Ed.* **2015**, *54*, 6913.

[19] T. Bury, S. Kullmann, B. Breit, *Adv. Synth. Catal.* **2023**, *365*, 335.

[20] M. S. Shepard, E. M. Carreira, *Tetrahedron* **1997**, *53*, 16253.

[21] D. J. Clausen, S. Wan, P. E. Floreancig, *Angew. Chem. Int. Ed.* **2011**, *50*, 5178.

[22] S. Ganss, B. Breit, *Angew. Chem.* **2016**, *128*, 9890.

[23] P. Koschker, M. Kähny, B. Breit, *J. Am. Chem. Soc.* **2015**, *137*, 3131.

[24] L. Zou, X. Bao, Y. Ma, Y. Song, J. Qu, B. Wang, *Chem. Commun.* **2014**, *50*, 5760.

[25] T. Kippo, T. Fukuyama, I. Ryu, *Org. Lett.* **2011**, *13*, 3864.

[26] Z. Cao, A. Scalabre, S. Nlate, S. Buffière, R. Oda, E. Pouget, B. Bibal, *Chem. Eur. J.* **2021**, *27*, 427.

[27] H. Chen, M. Yang, G. Wang, L. Gao, Z. Ni, J. Zou, S. Li, *Org. Lett.* **2021**, *23*, 5533.

[28] D. G. Kohler, S. N. Gockel, J. L. Kennemur, P. J. Waller, K. L. Hull, *Nat. Chem.* **2018**, *10*, 333.

[29] K. Skobridis, M. Kinigopoulou, V. Theodorou, E. Giannousi, A. Russell, R. Chauhan, R. Sala, N. Brownlow, S. Kiriakidis, J. Domin et al., *ChemMedChem* **2010**, *5*, 130.

[30] L. Krause, R. Herbst-Irmer, G. M. Sheldrick, D. Stalke, *J. Appl. Cryst.* **2015**, *48*, 3.

[31] a) G. M. Sheldrick, *Struct. Chem.* **2015**, *71*, 3; b) G. M. Sheldrick, *Acta Crystallogr. A* **2015**, *71*, 3.

[32] C. R. Groom, I. J. Bruno, M. P. Lightfoot, S. C. Ward, *Acta Crystallogr. B* **2016**, *72*, 171.

8 Appendix

8.1 Abbreviations

6-DPPon	6-diphenylphosphinopyridin-2-(1*H*)-one
BINAP	2,2′-bis(diphenylphosphino)-1,1′-binaphthyl
COD	cycloocta-1,5-diene
CPTS	collidinium *p*-toluenesulfonate
CPTS	Camphor-10-sulfonic acid
d	day(s)
DBM	dibromomethane
DIAD	diisopropyl azodicarboxylate
DMAP	*N*,*N*-Dimethylpyridin-4-amine
DMF	*N*,*N*-dimethylformamide
DMSO	dimethyl sulfoxide
dppf	1,1′-bis(diphenylphosphino)ferrocene
DTBM	diterbutylmethoxy
equiv	equivalents
h	hour
HPLC	high-performance liquid chromatography
IPA	isopropanol
min	minutes
NMR	nuclear magnetic resonance
PC	protective group
PES	Pyridinium ethylsulfate
PMB	*para*-methoxy benzyl
PPTS	Pyridinium *p*-toluolsulfonat
PTFA	Pyridinium triflate
quant	quantitaive
rt	room temperature
TBS	*tert*-butyldimethylsilyl
TBDPS	*tert*-butyldiphenylsilyl
TIPS	triisopropylsilyl
TFA	Trifluoroacetic acid
THF	tetrahydrofuran

8.2 NMR Spectra

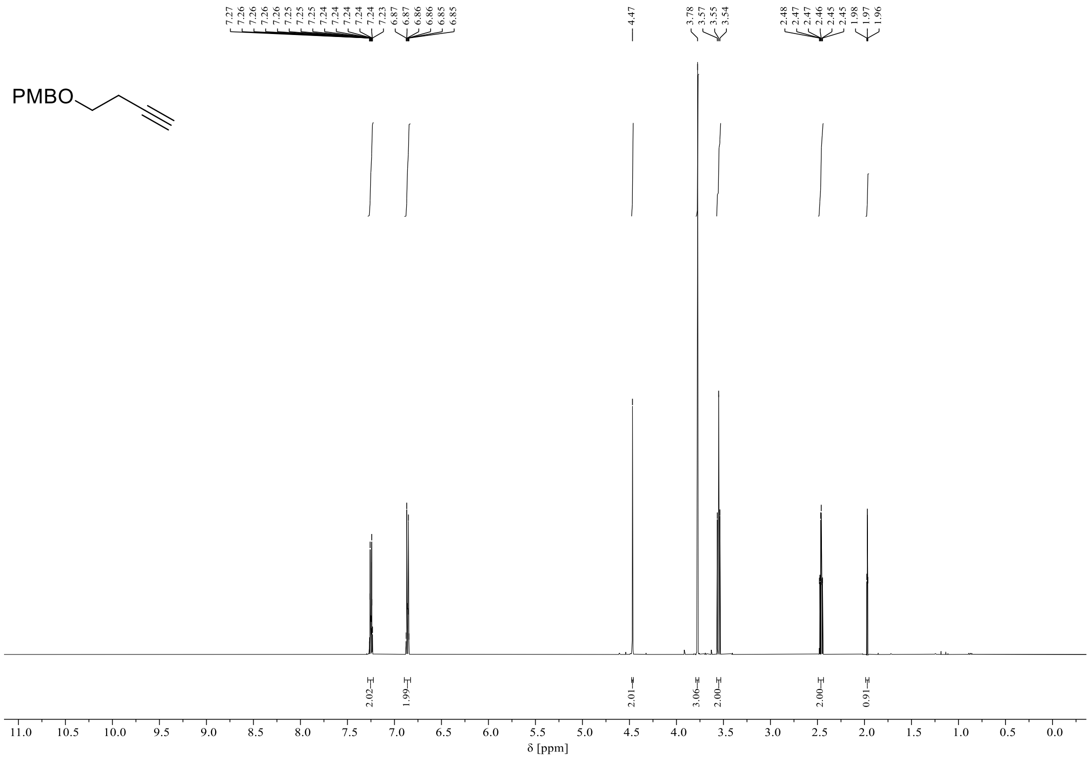

Figure 3: ^{1}H-NMR spectrum of 1-((but-3-yn-1-yloxy)methyl)-4-methoxybenzene ($CDCl_3$, 500 MHz).

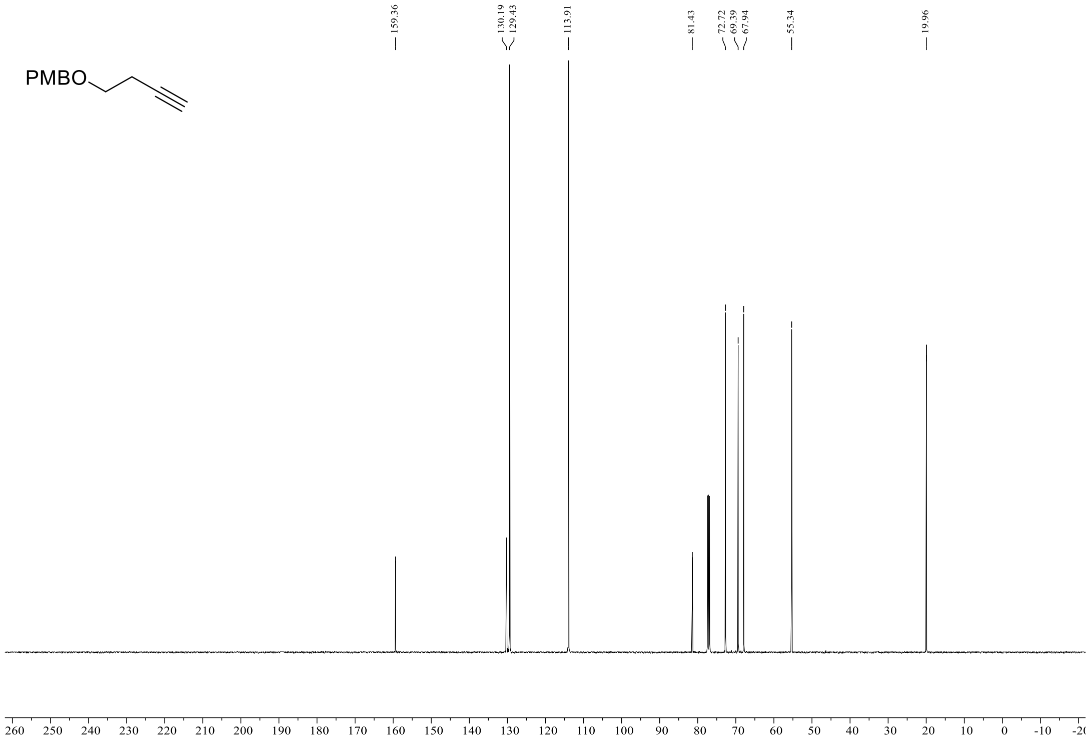

Figure 4: ^{13}C-NMR spectrum of 1-((but-3-yn-1-yloxy)methyl)-4-methoxybenzene ($CDCl_3$, 126 MHz).

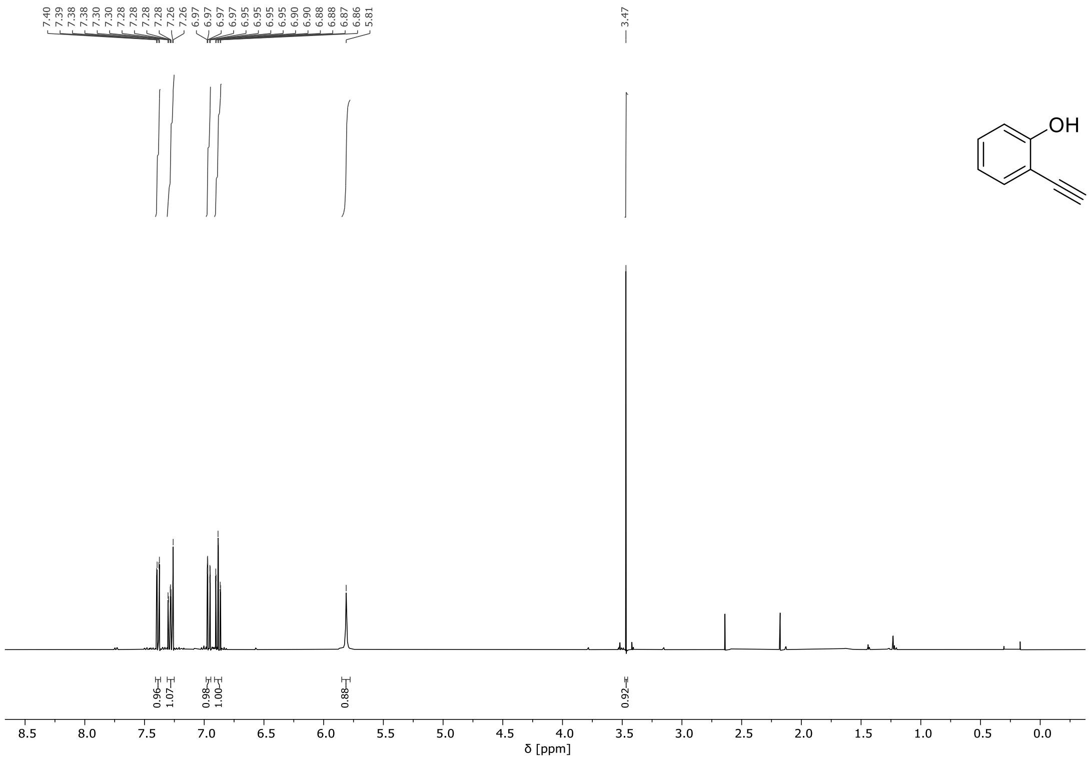

Figure 5: ^{1}H-NMR spectrum of 2-ethynylphenol ($CDCl_3$, 400 MHz, contains acetone).

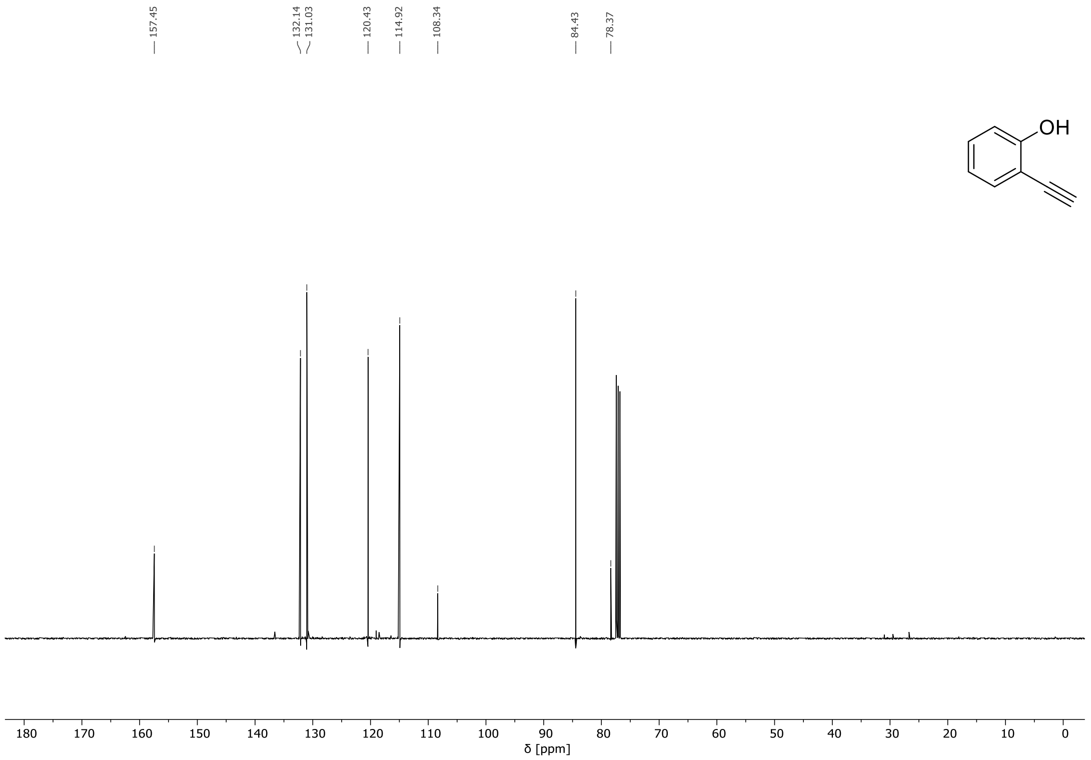

Figure 6: ^{13}C-NMR spectrum of 2-ethynylphenol ($CDCl_3$, 101 MHz).

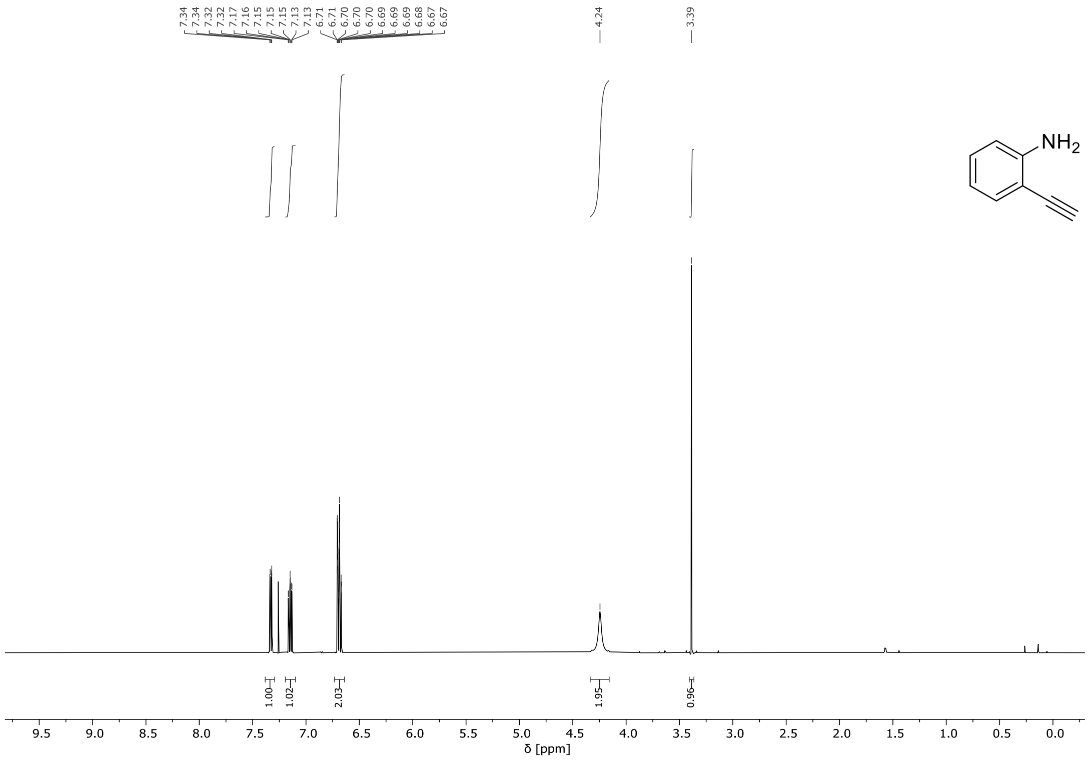

Figure 7: ^{1}H-NMR spectrum of 2-ethynylaniline ($CDCl_3$, 500 MHz).

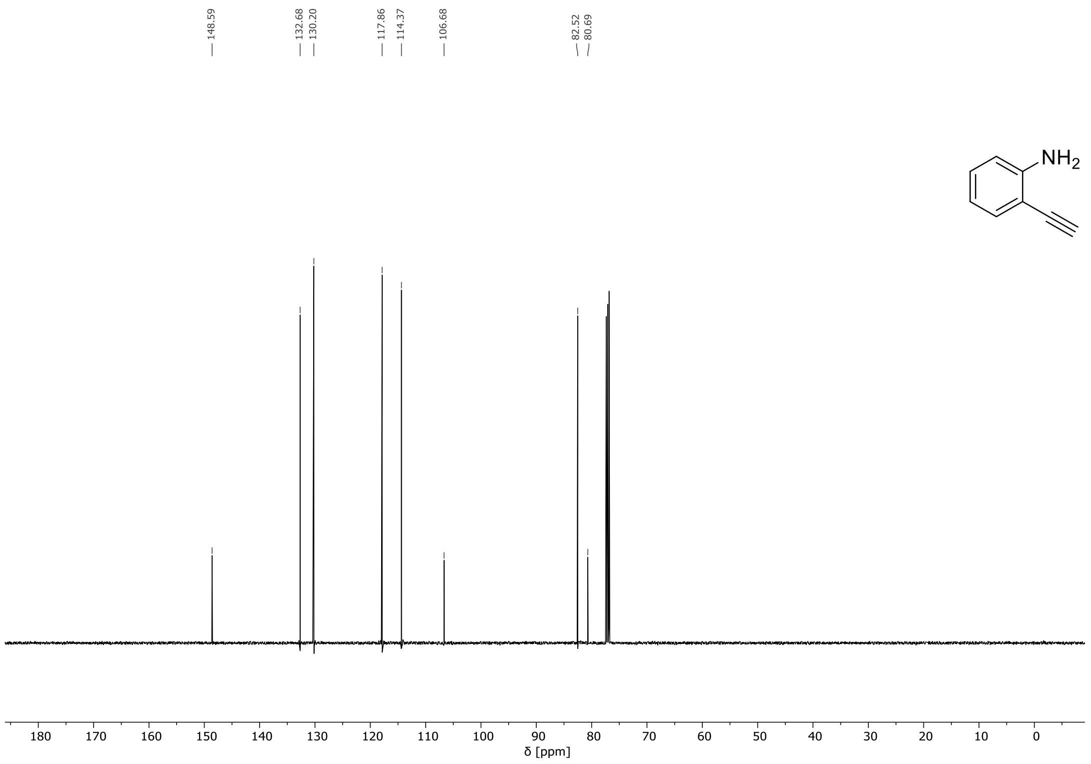

Figure 8: ^{13}C-NMR spectrum of 2-ethynylaniline ($CDCl_3$, 126 MHz).

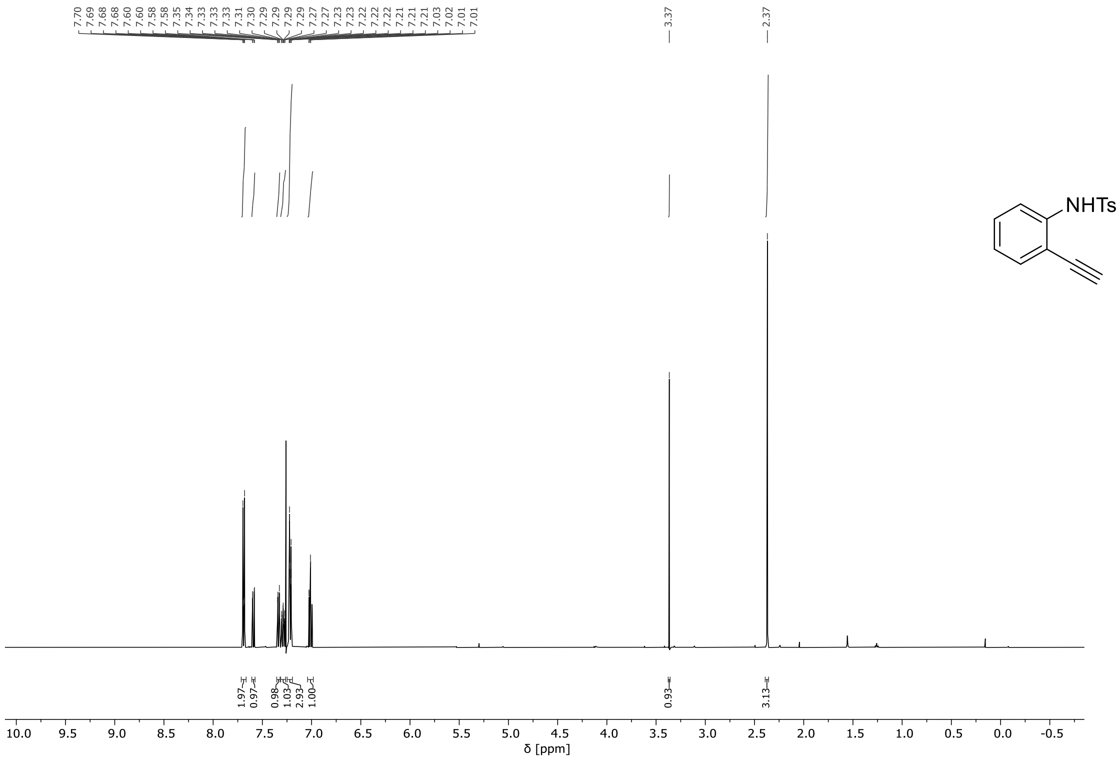

Figure 9: ^{1}H-NMR spectrum of *N*-(2-ethynylphenyl)-4-methylbenzenesulfonamide ($CDCl_3$, 500 MHz).

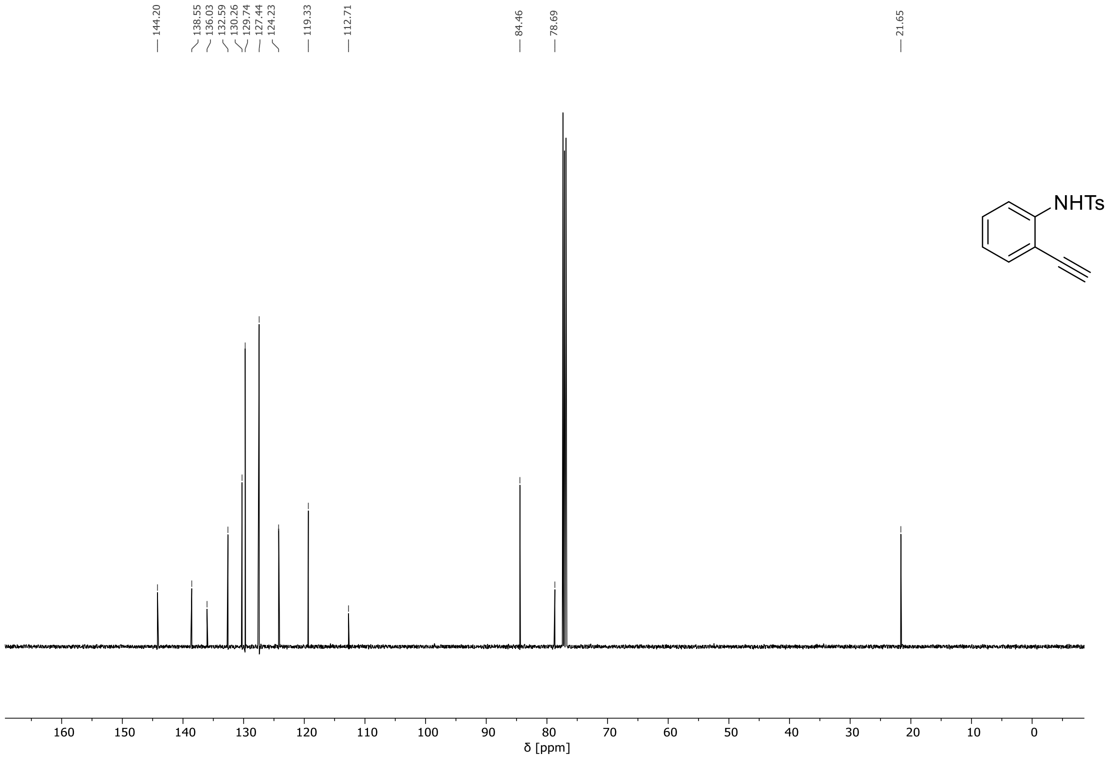

Figure 10: ^{13}C-NMR spectrum of *N*-(2-ethynylphenyl)-4-methylbenzenesulfonamide ($CDCl_3$, 126 MHz).

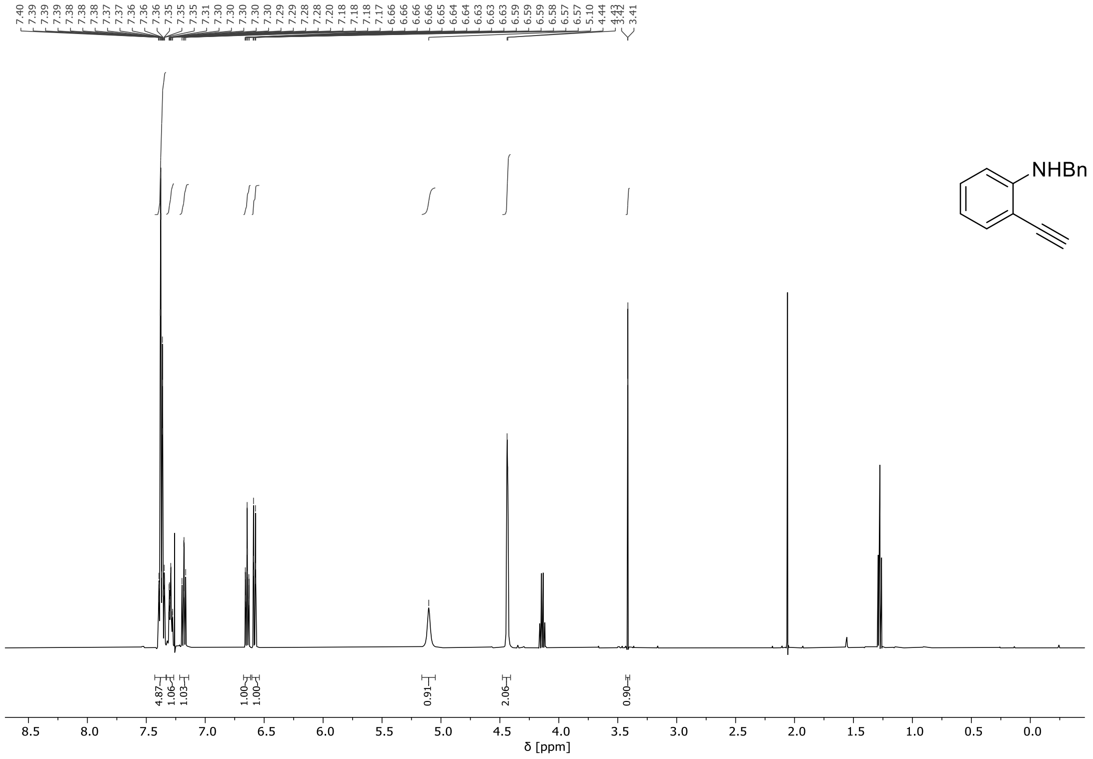

Figure 11: ^{1}H-NMR spectrum of *N*-benzyl-2-ethynylaniline ($CDCl_3$, 500 MHz, contains EtOAc).

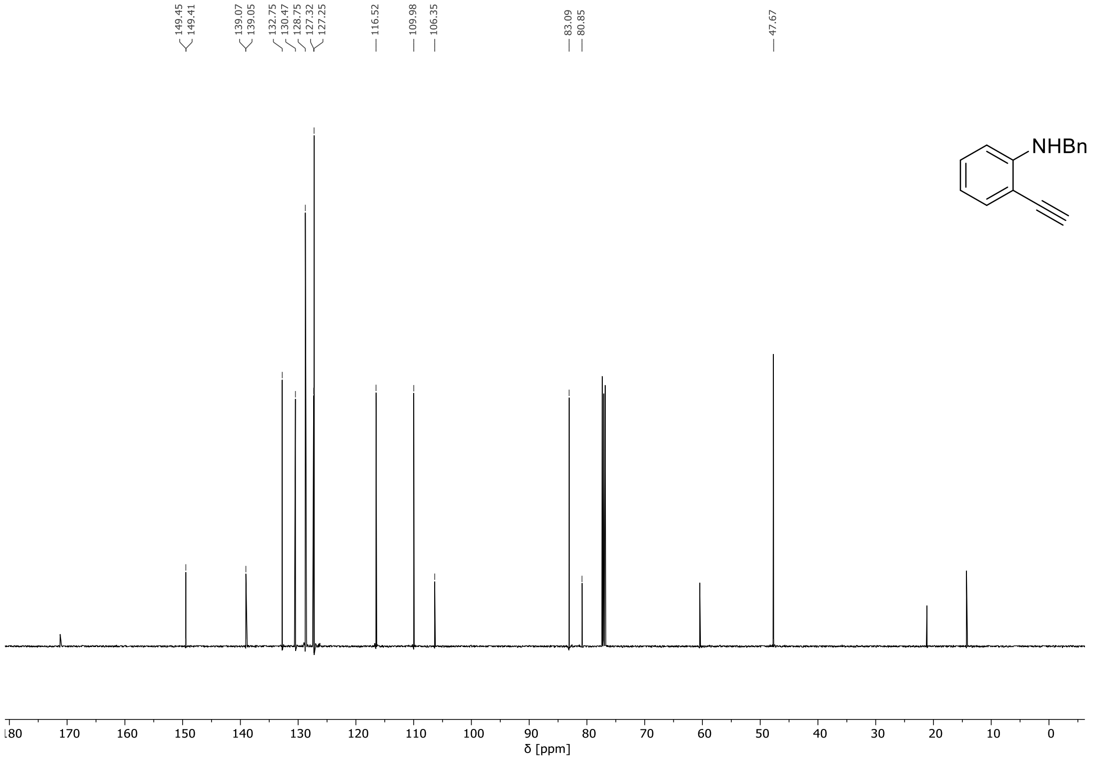

Figure 12: ^{1}H-NMR spectrum of *N*-benzyl-2-ethynylaniline ($CDCl_3$, 126 MHz, contains EtOAc).

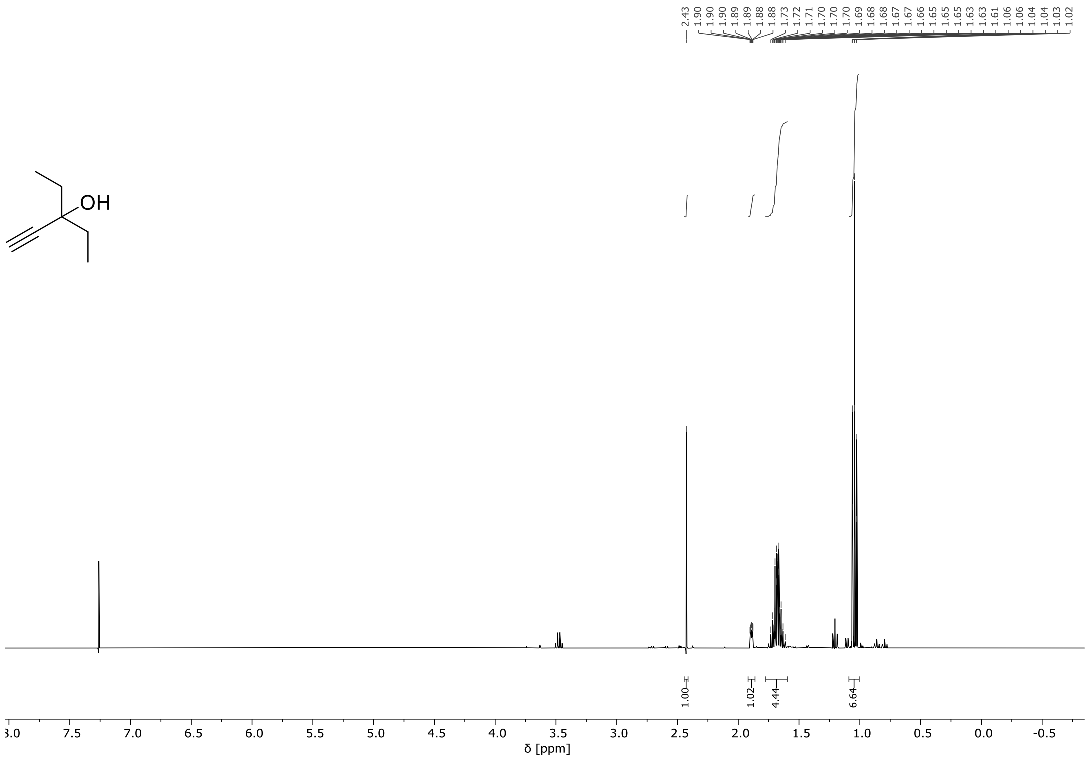

Figure 13: ^{1}H-NMR spectrum of 3-ethylpent-1-yn-3-ol ($CDCl_3$, 400 MHz, contains Et_2O).

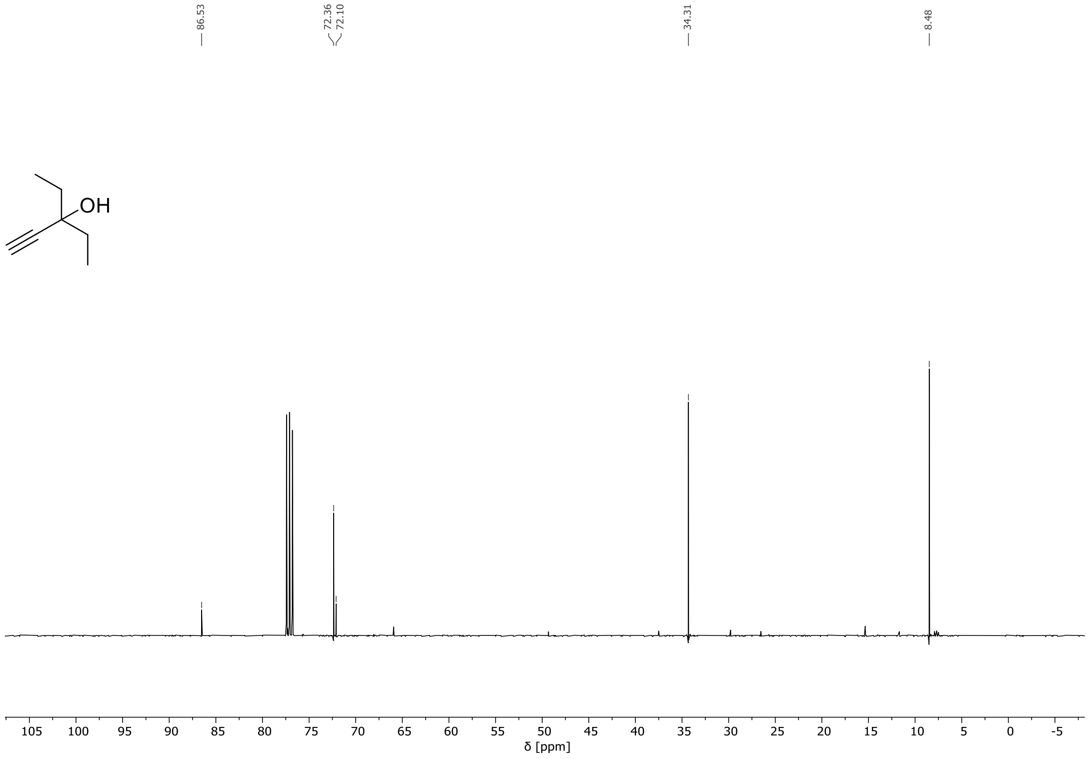

Figure 14: ^{13}C-NMR spectrum of 3-ethylpent-1-yn-3-ol ($CDCl_3$, 101 MHz).

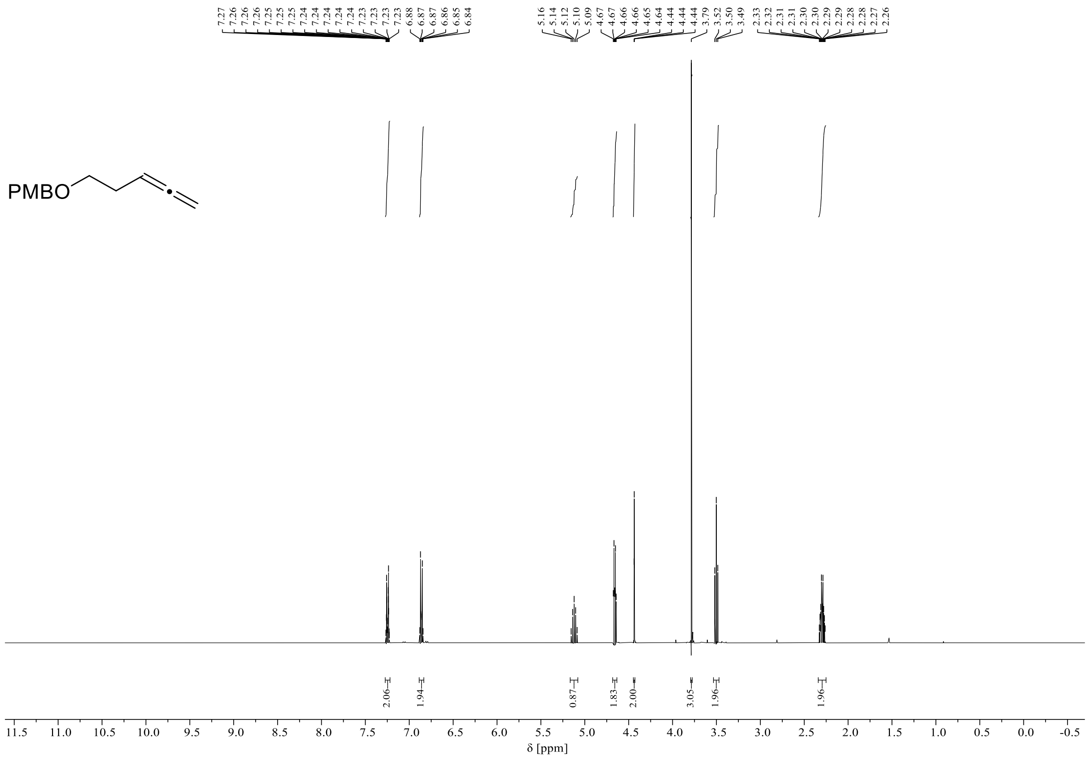

Figure 15: ^{1}H-NMR spectrum of 1-methoxy-4-((penta-3,4-dien-1-yloxy)methyl)benzene ($CDCl_3$, 400 MHz).

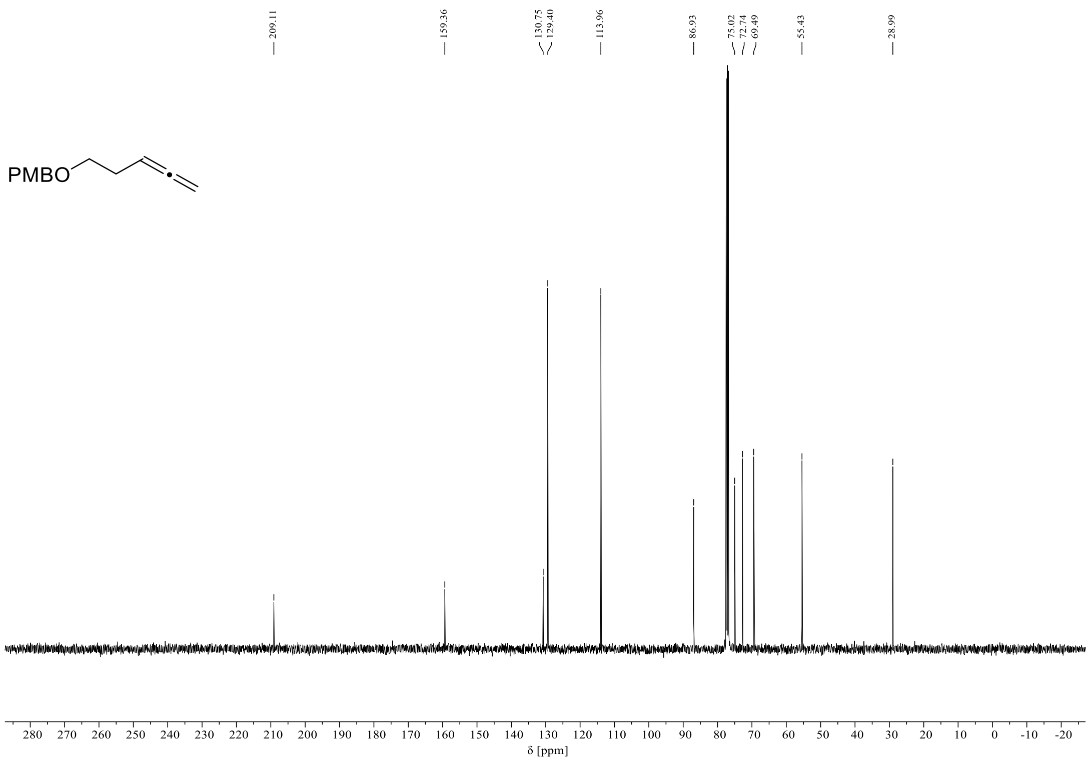

Figure 16: ^{13}C-NMR spectrum of 1-methoxy-4-((penta-3,4-dien-1-yloxy)methyl)benzene ($CDCl_3$, 101 MHz).

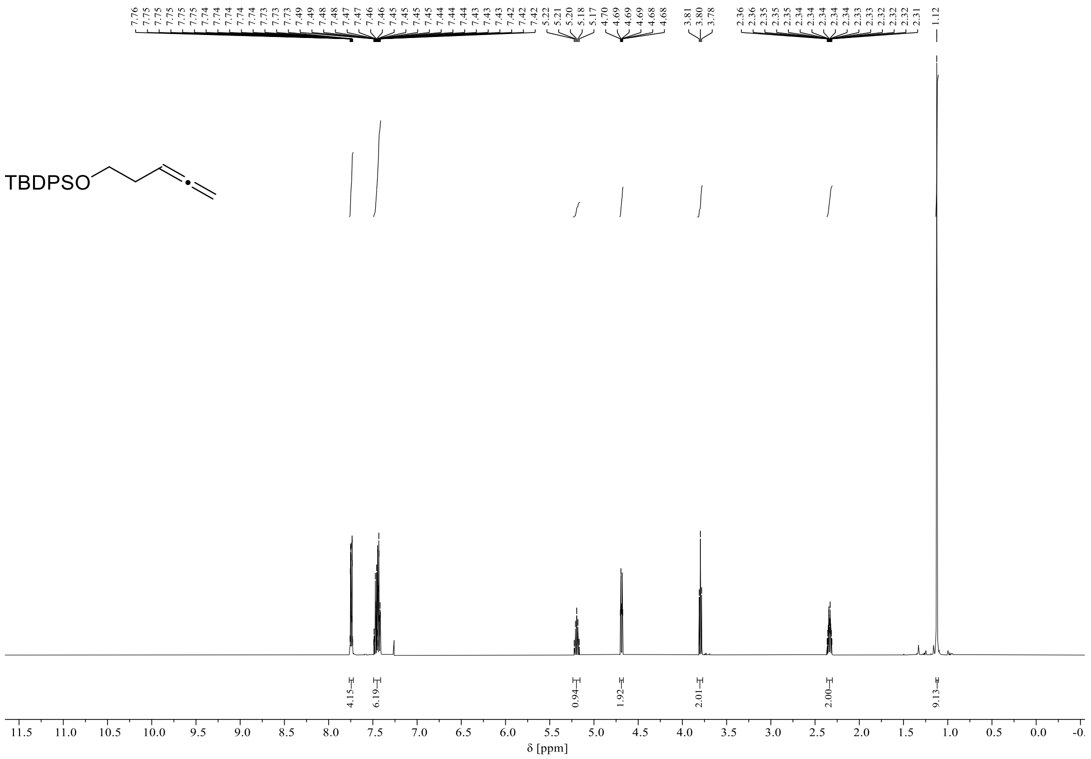

Figure 17: ^{1}H-NMR spectrum of *tert*-butyl(penta-3,4-dien-1-yloxy)diphenylsilane ($CDCl_3$, 500 MHz).

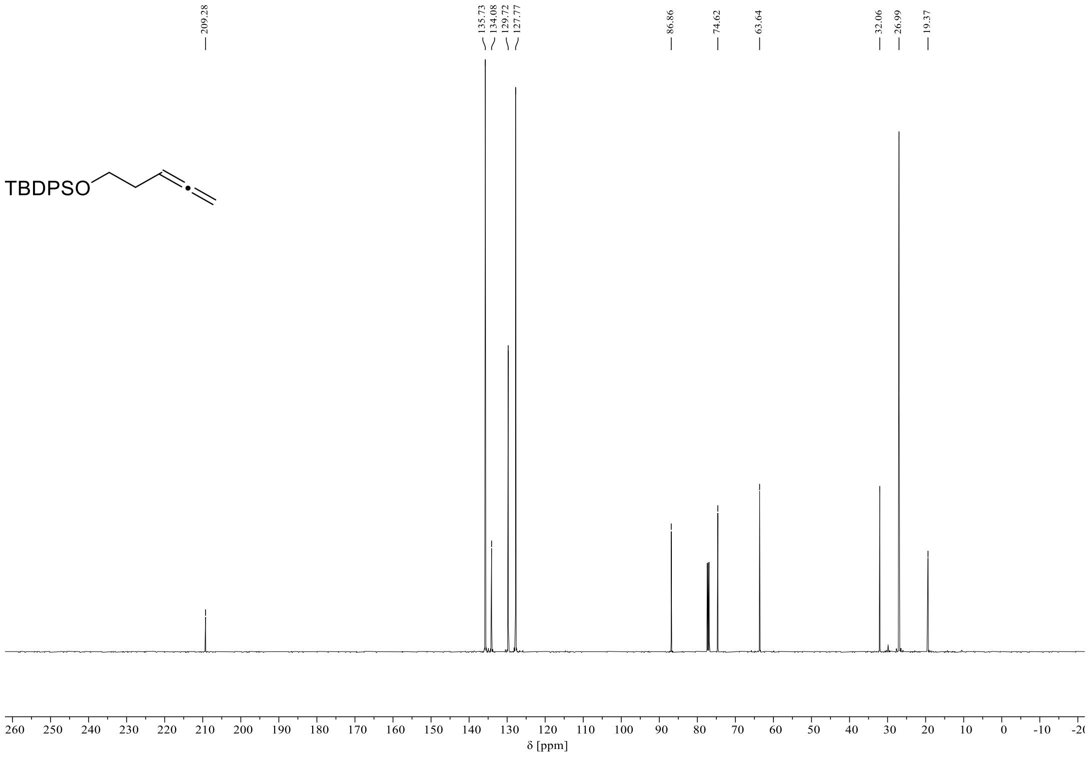

Figure 18: ^{13}C-NMR spectrum of *tert*-butyl(penta-3,4-dien-1-yloxy)diphenylsilane ($CDCl_3$, 126 MHz).

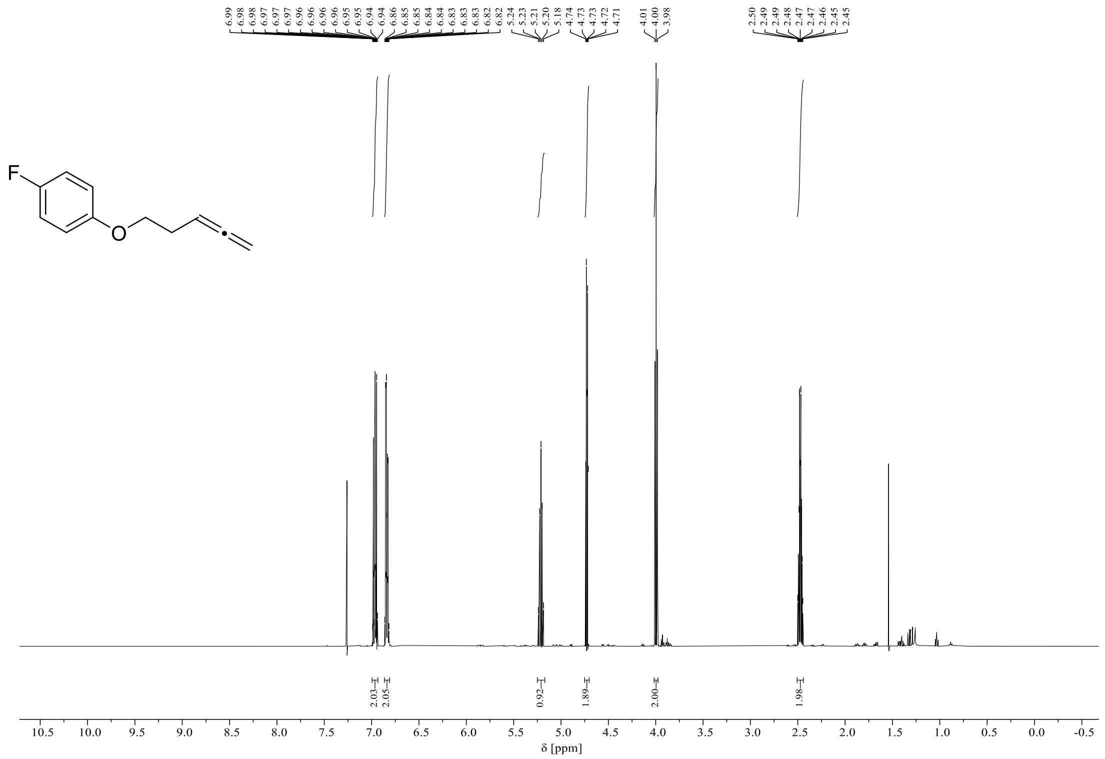

Figure 19: ^{1}H-NMR spectrum of 1-fluoro-4-(penta-3,4-dien-1-yloxy)benzene ($CDCl_3$, 500 MHz).

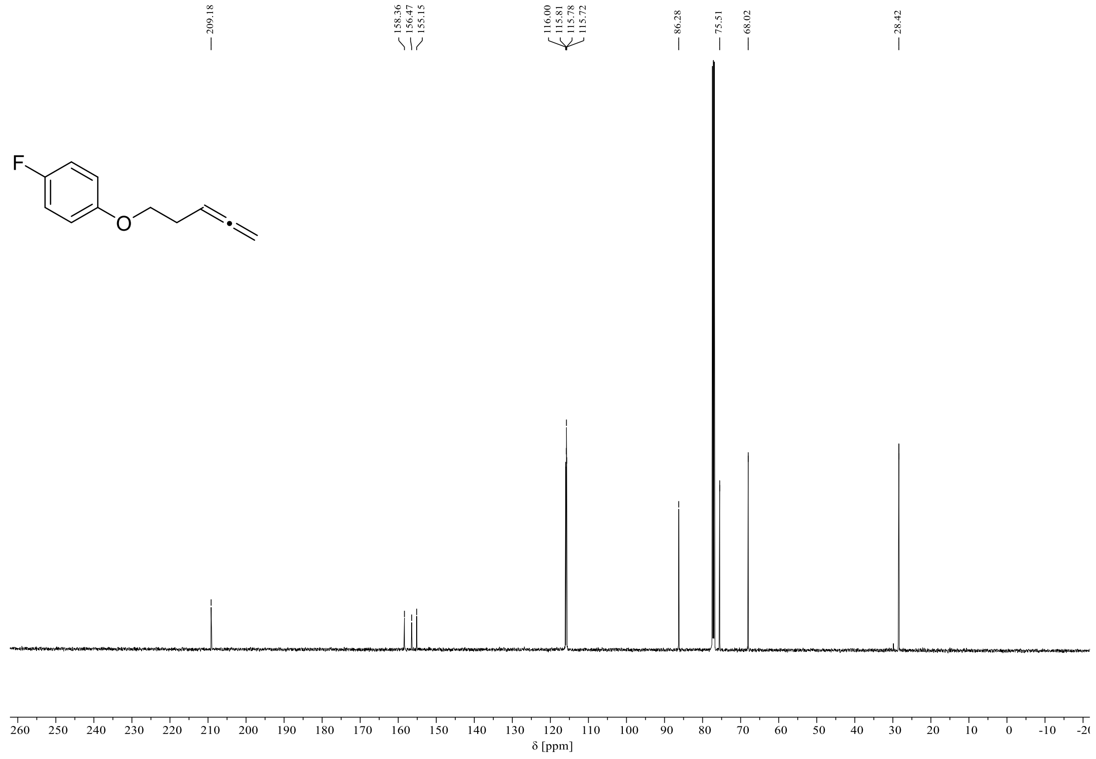

Figure 20: ^{13}C-NMR spectrum of 1-fluoro-4-(penta-3,4-dien-1-yloxy)benzene ($CDCl_3$, 126 MHz).

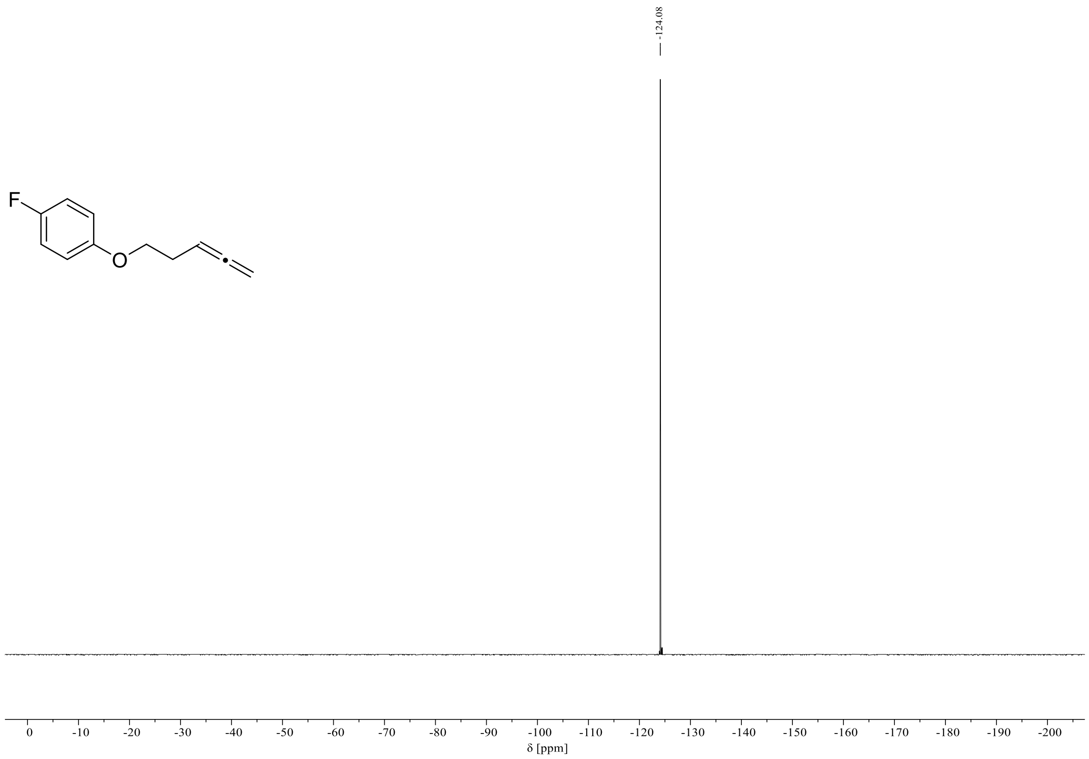

Figure 21: ^{19}F-NMR spectrum of 1-fluoro-4-(penta-3,4-dien-1-yloxy)benzene ($CDCl_3$, 471 MHz).

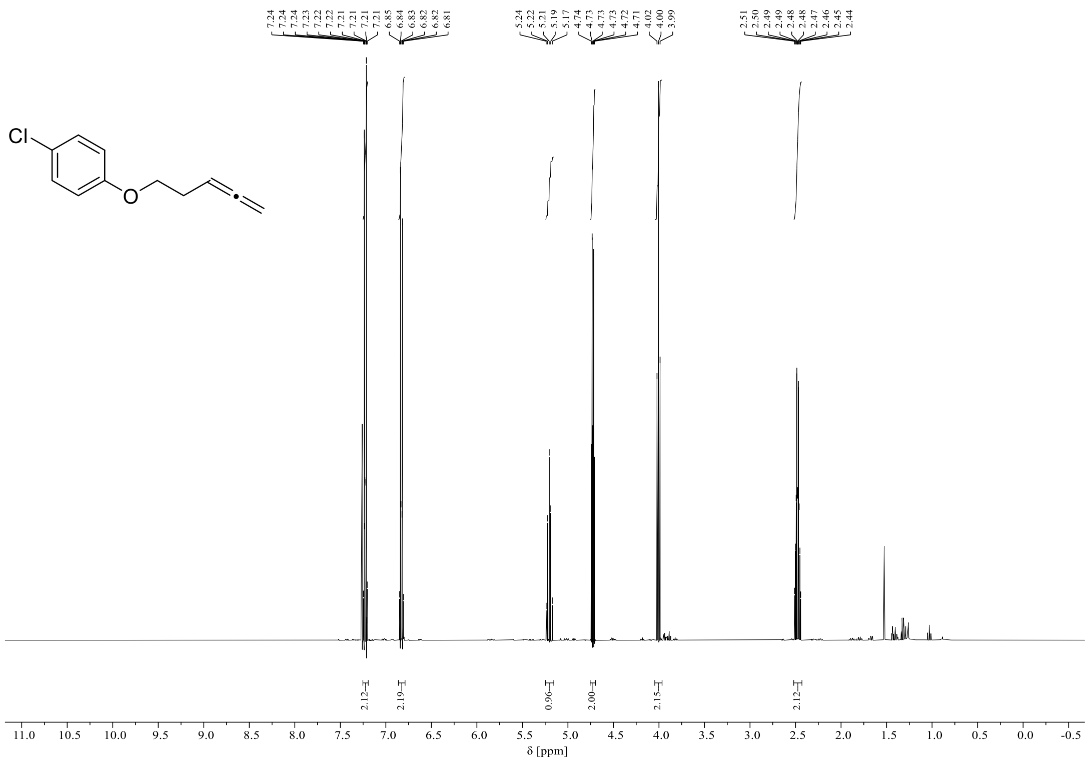

Figure 22: ^{1}H-NMR spectrum of 1-chloro-4-(penta-3,4-dien-1-yloxy)benzene ($CDCl_3$, 400 MHz).

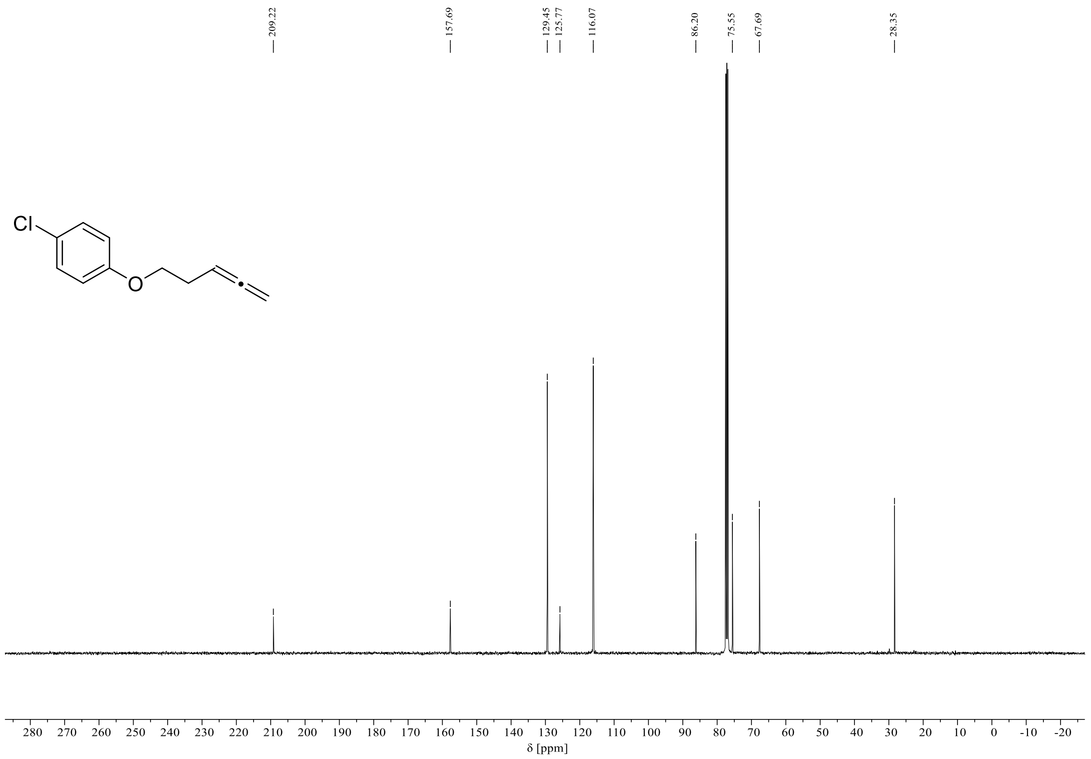

Figure 23: ^{13}C-NMR spectrum of 1-chloro-4-(penta-3,4-dien-1-yloxy)benzene ($CDCl_3$, 101 MHz).

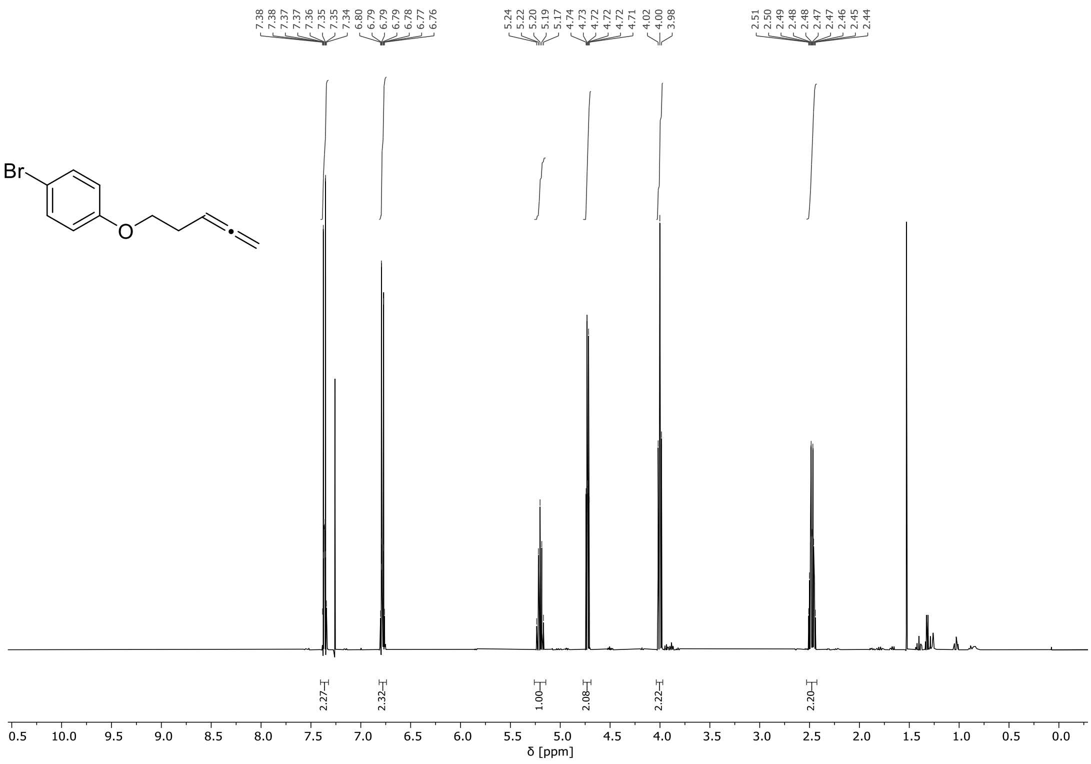

Figure 24: ^{1}H-NMR spectrum of 1-bromo-4-(penta-3,4-dien-1-yloxy)benzene ($CDCl_3$, 400 MHz).

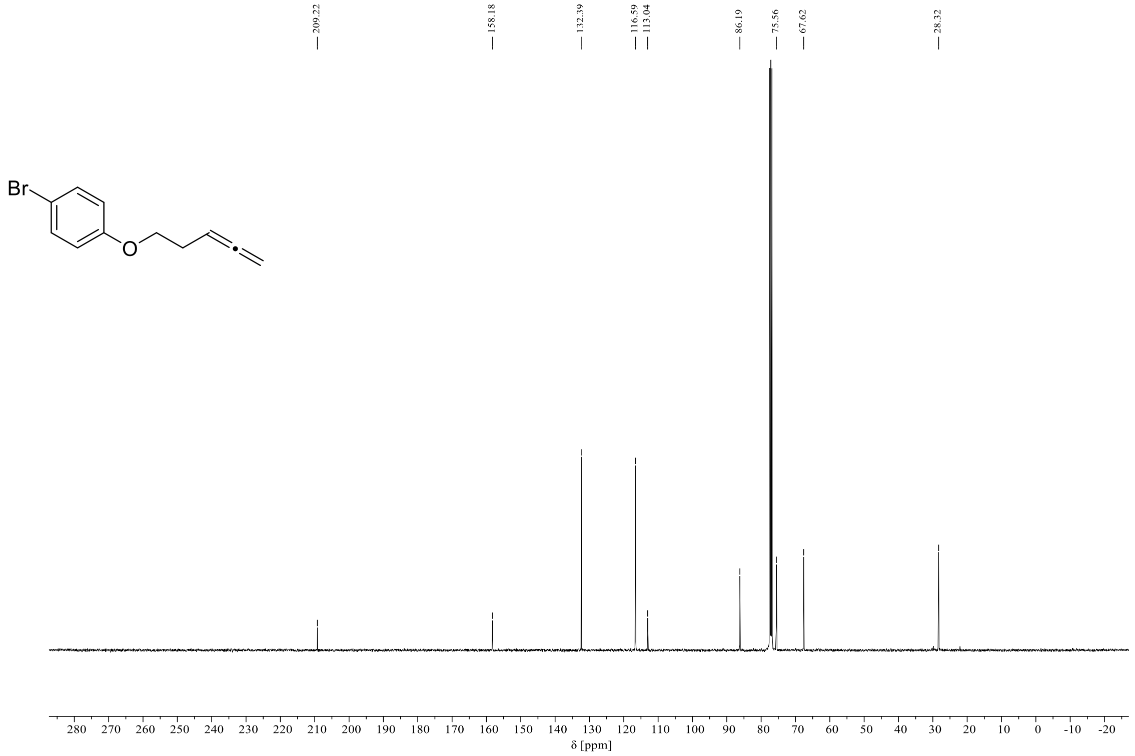

Figure 25: ^{13}C-NMR spectrum of 1-bromo-4-(penta-3,4-dien-1-yloxy)benzene ($CDCl_3$, 101 MHz).

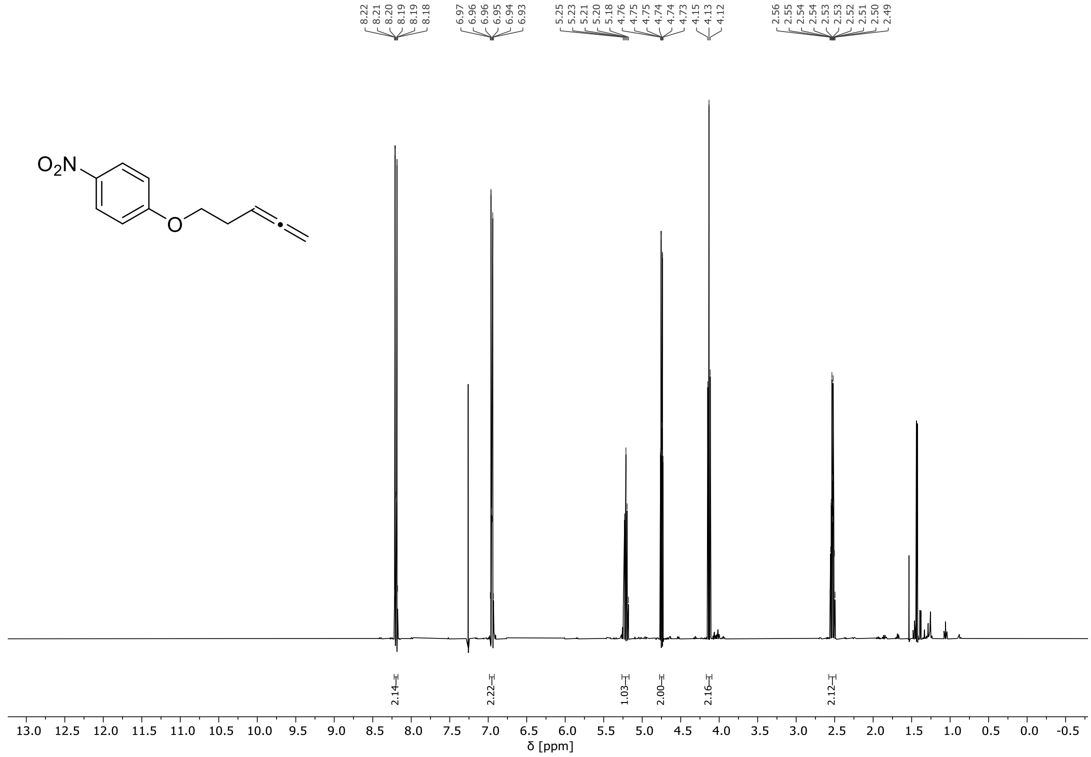

Figure 26: ^{1}H-NMR spectrum of 1-nitro-4-(penta-3,4-dien-1-yloxy)benzene ($CDCl_3$, 400 MHz).

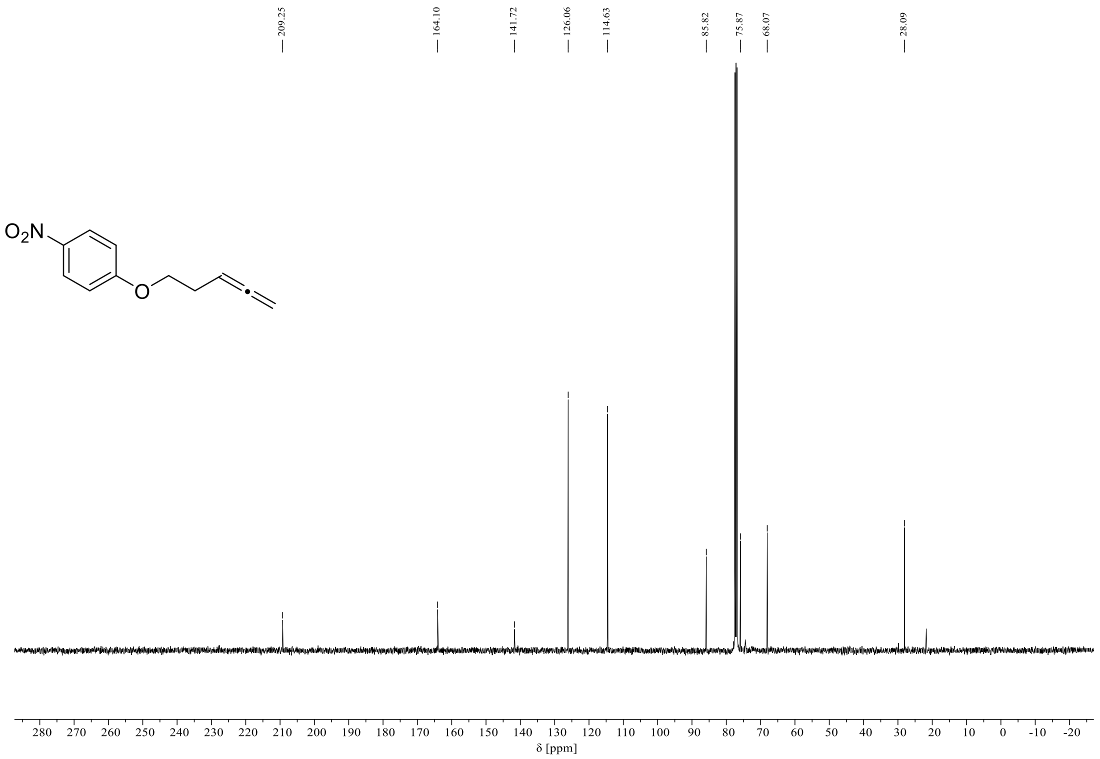

Figure 27: ^{13}C-NMR spectrum of 1-nitro-4-(penta-3,4-dien-1-yloxy)benzene ($CDCl_3$, 101 MHz).

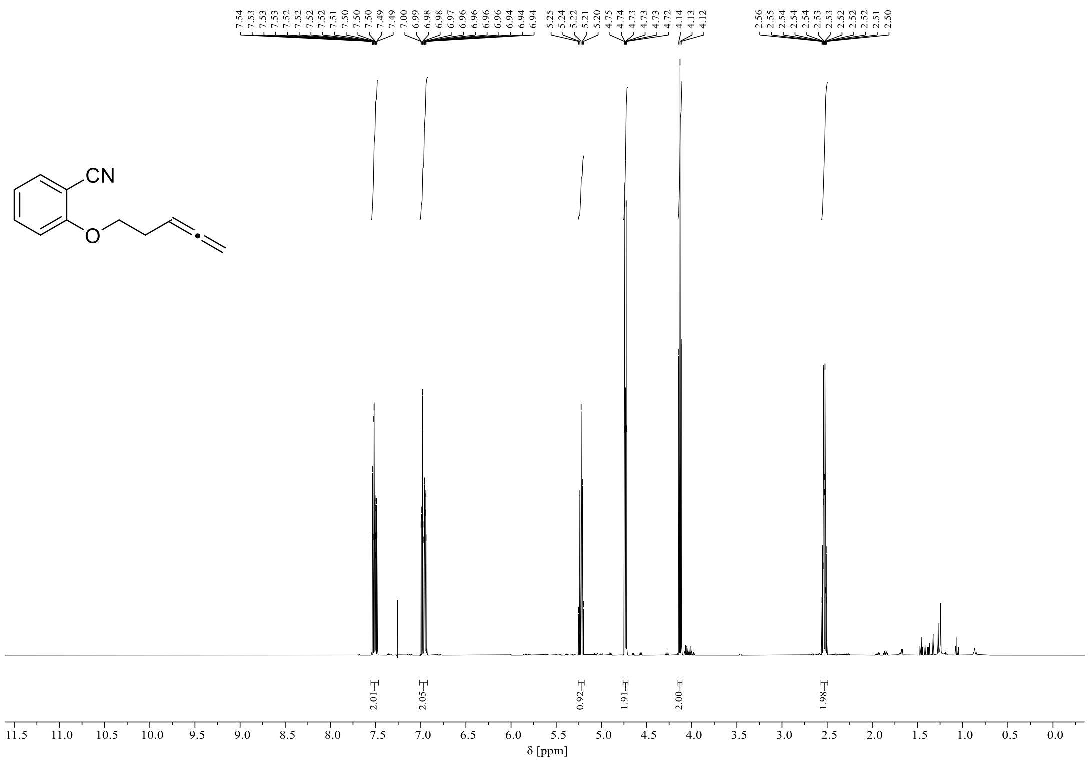

Figure 28: ^{1}H-NMR spectrum of 2-(penta-3,4-dien-1-yloxy)benzonitrile ($CDCl_3$, 500 MHz).

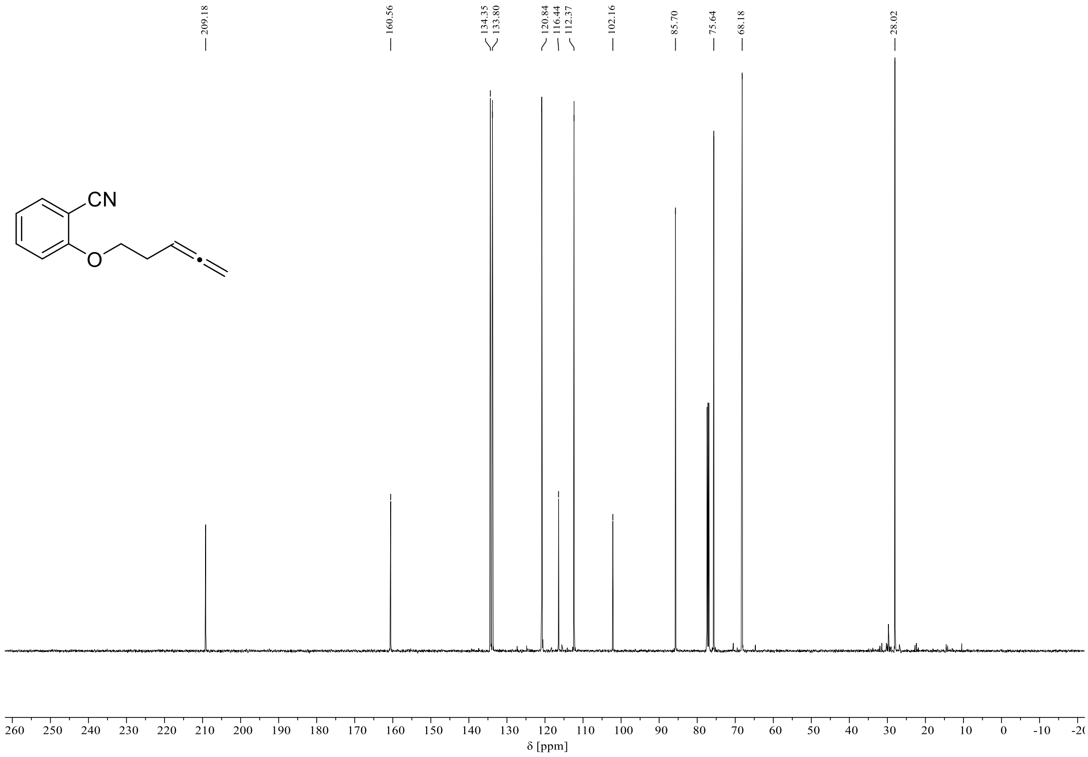

Figure 29: ^{13}C-NMR spectrum of 2-(penta-3,4-dien-1-yloxy)benzonitrile ($CDCl_3$, 126 MHz).

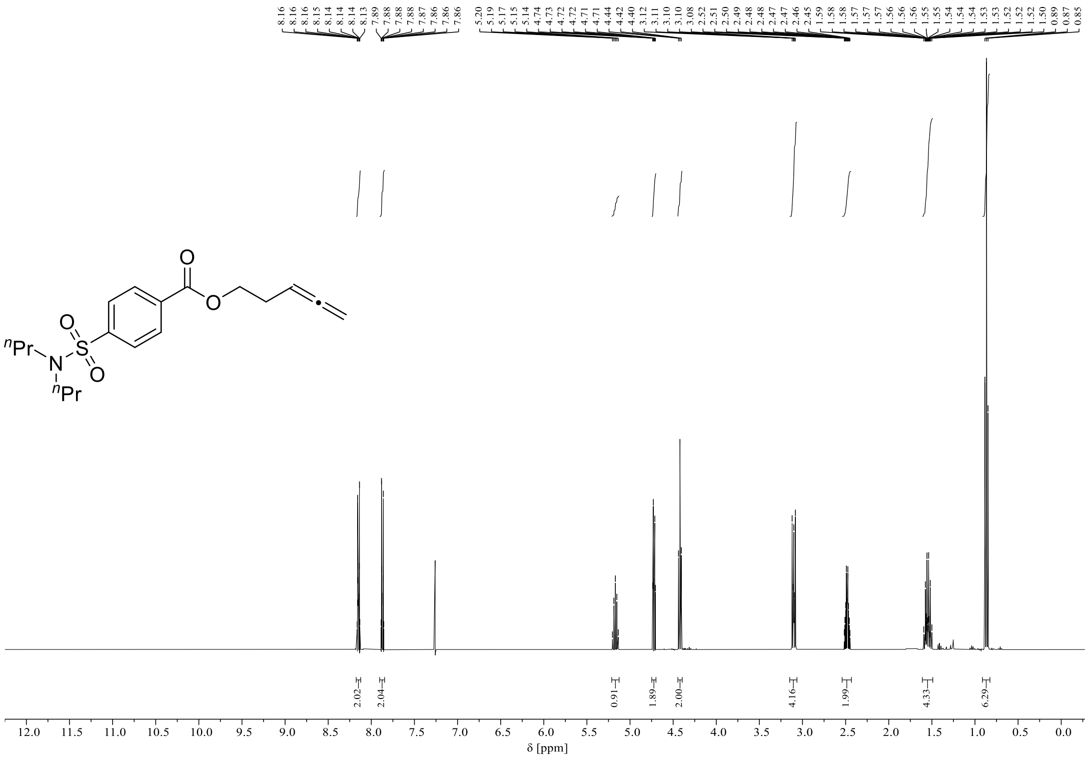

Figure 30: ^{1}H-NMR spectrum of penta-3,4-dien-1-yl 4-(*N*,*N*-dipropylsulfamoyl)benzoate ($CDCl_3$, 400 MHz).

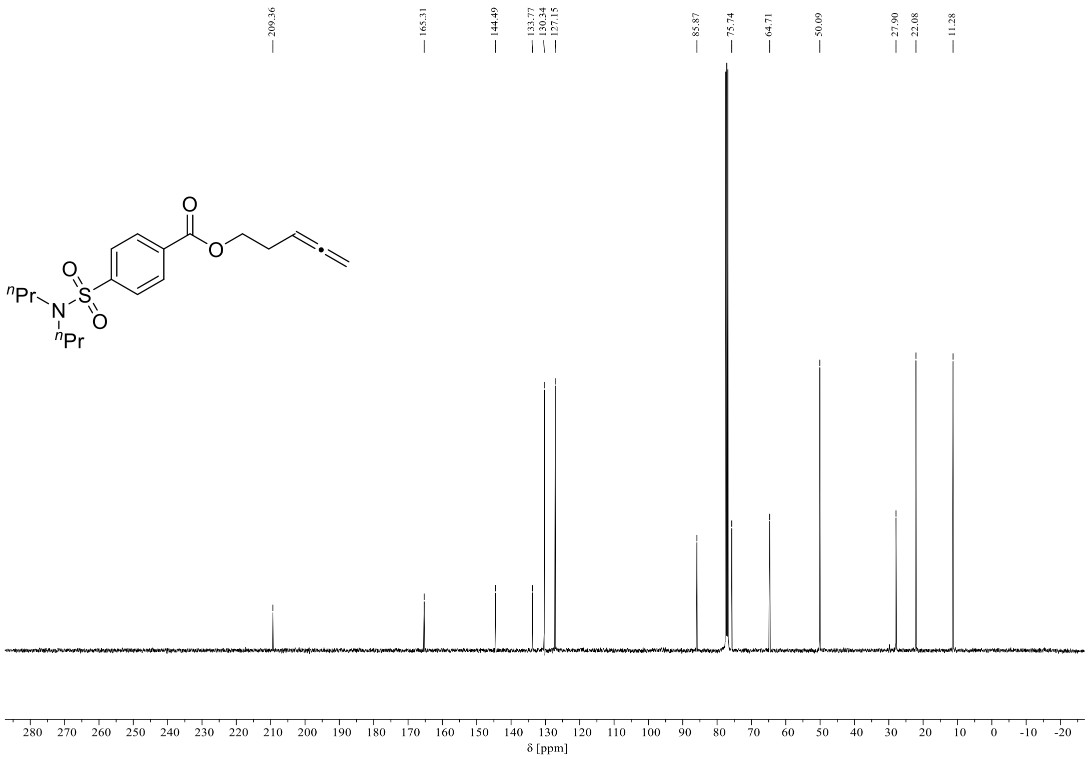

Figure 31: ^{13}C-NMR spectrum of penta-3,4-dien-1-yl 4-(*N*,*N*-dipropylsulfamoyl)benzoate ($CDCl_3$, 101 MHz).

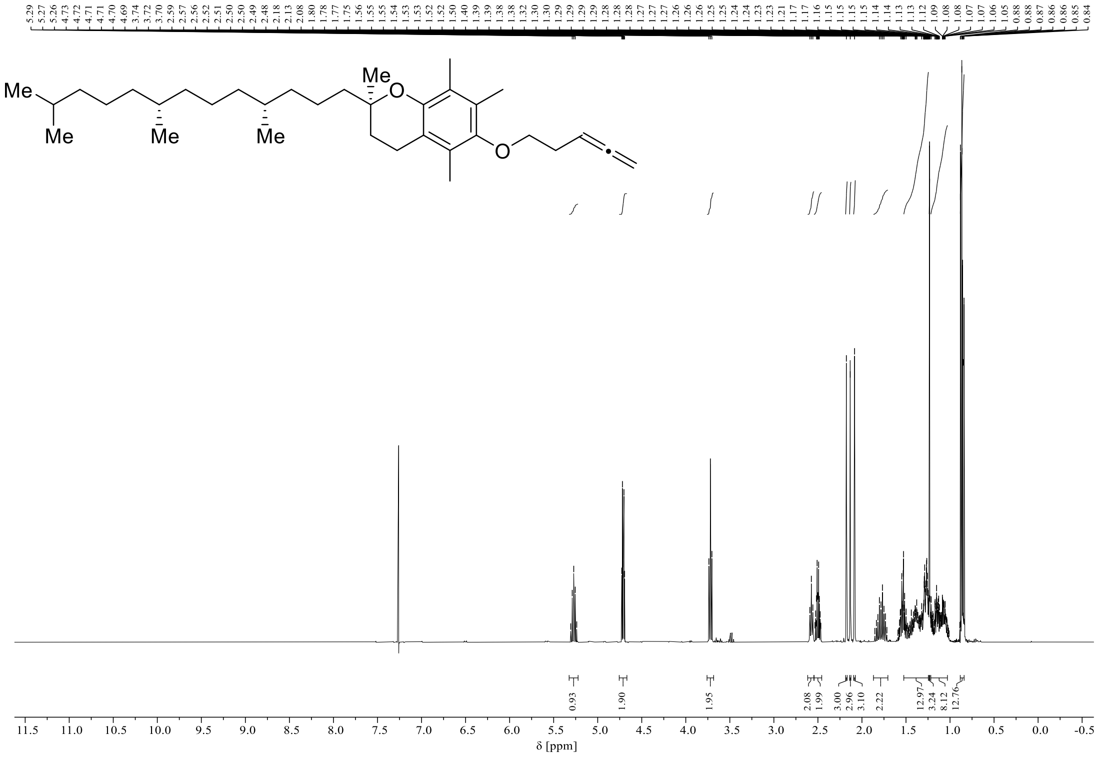

Figure 32: ^{1}H-NMR spectrum of (*R*)-2,5,7,8-tetramethyl-6-(penta-3,4-dien-1-yloxy)-2-((4*R*,8*R*)-4,8,12-trimethyltridecyl)chromane ($CDCl_3$, 400 MHz).

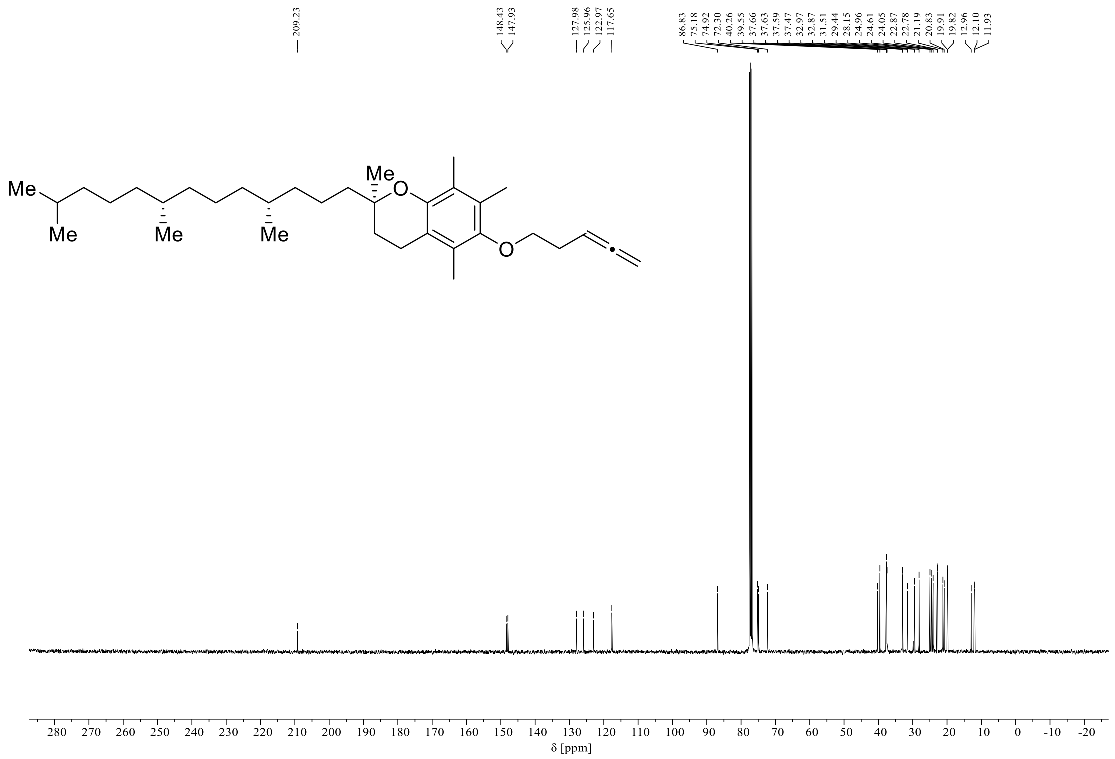

Figure 33: ^{13}C-NMR spectrum of (*R*)-2,5,7,8-tetramethyl-6-(penta-3,4-dien-1-yloxy)-2-((4*R*,8*R*)-4,8,12-trimethyltridecyl)chromane ($CDCl_3$, 101 MHz).

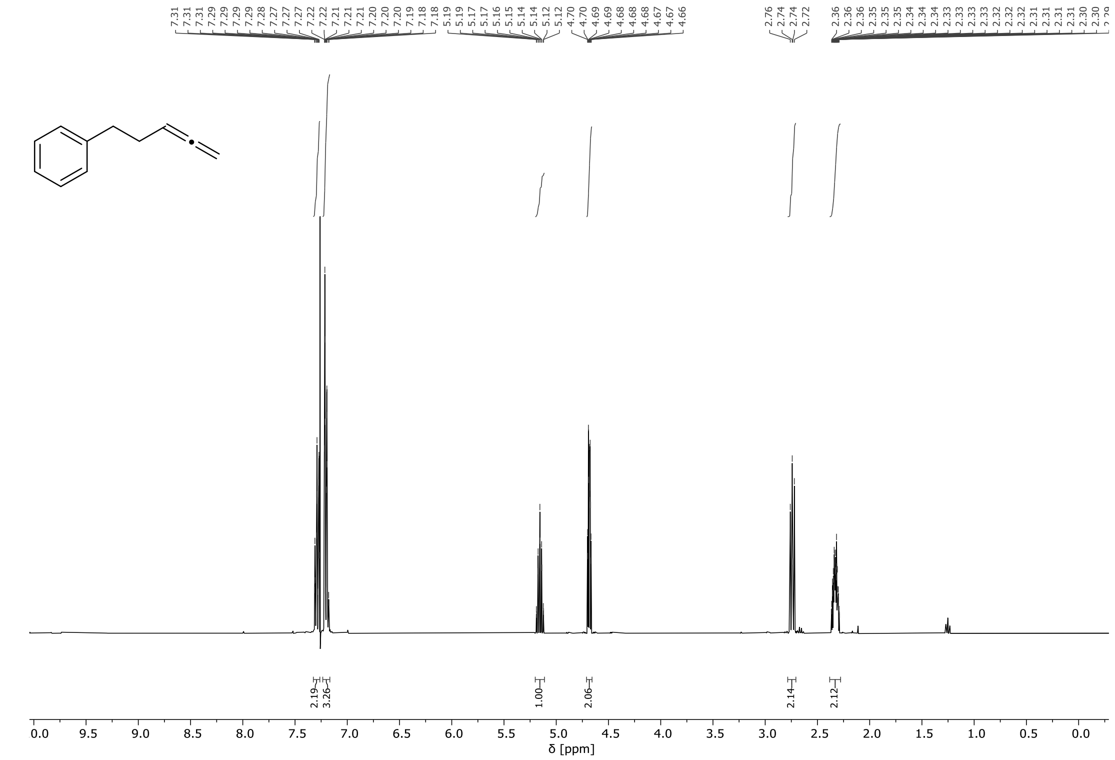

Figure 34: ^{1}H-NMR spectrum of penta-3,4-dien-1-ylbenzene ($CDCl_3$, 400 MHz).

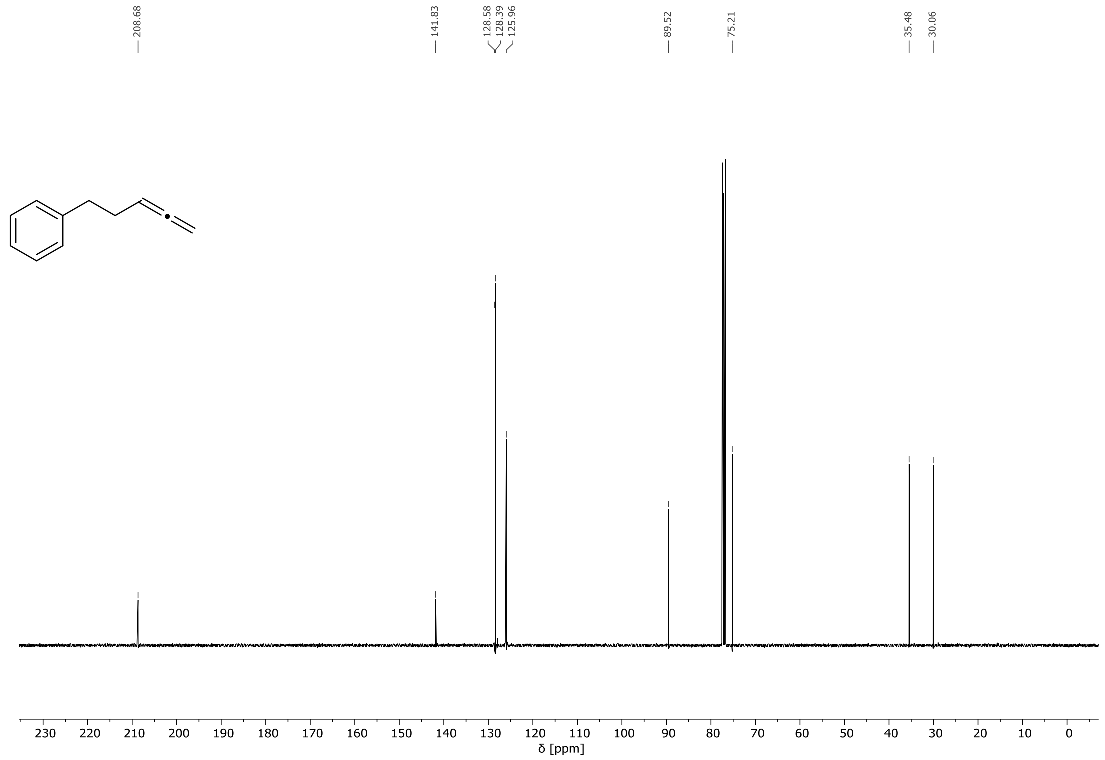

Figure 35: ^{13}C-NMR spectrum of penta-3,4-dien-1-ylbenzene ($CDCl_3$, 101 MHz).

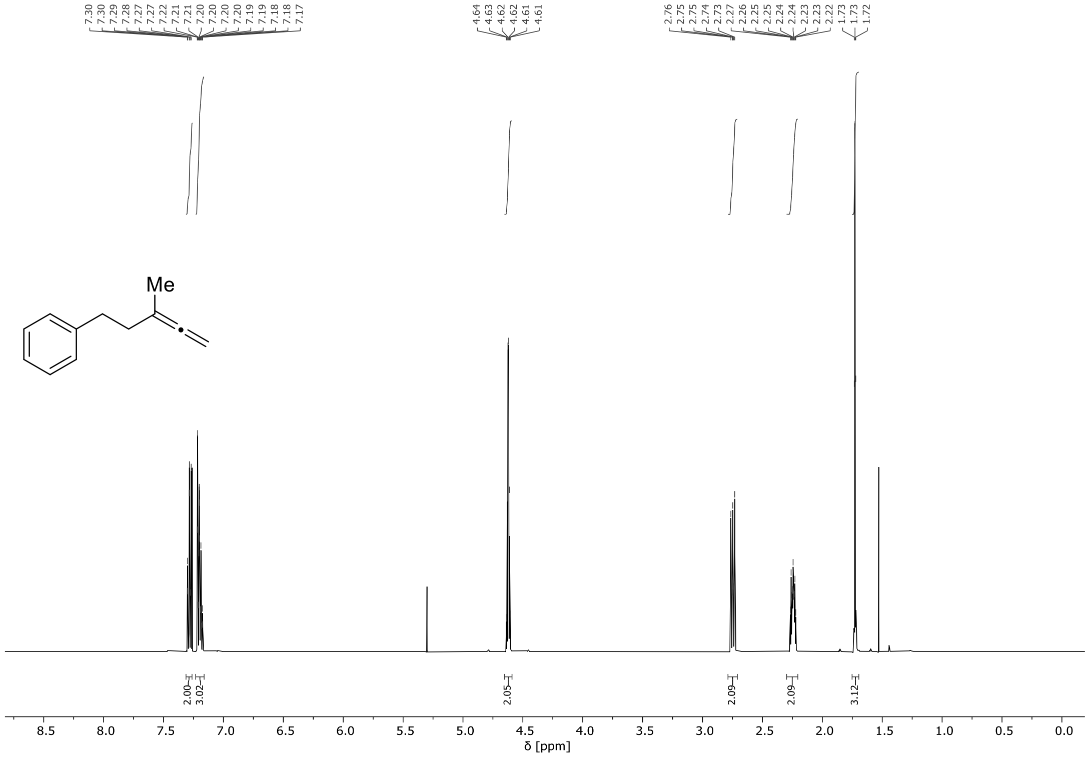

Figure 36: ^{1}H-NMR spectrum of (3-methylpenta-3,4-dien-1-yl)benzene ($CDCl_3$, 500 MHz, contains CH_2Cl_2).

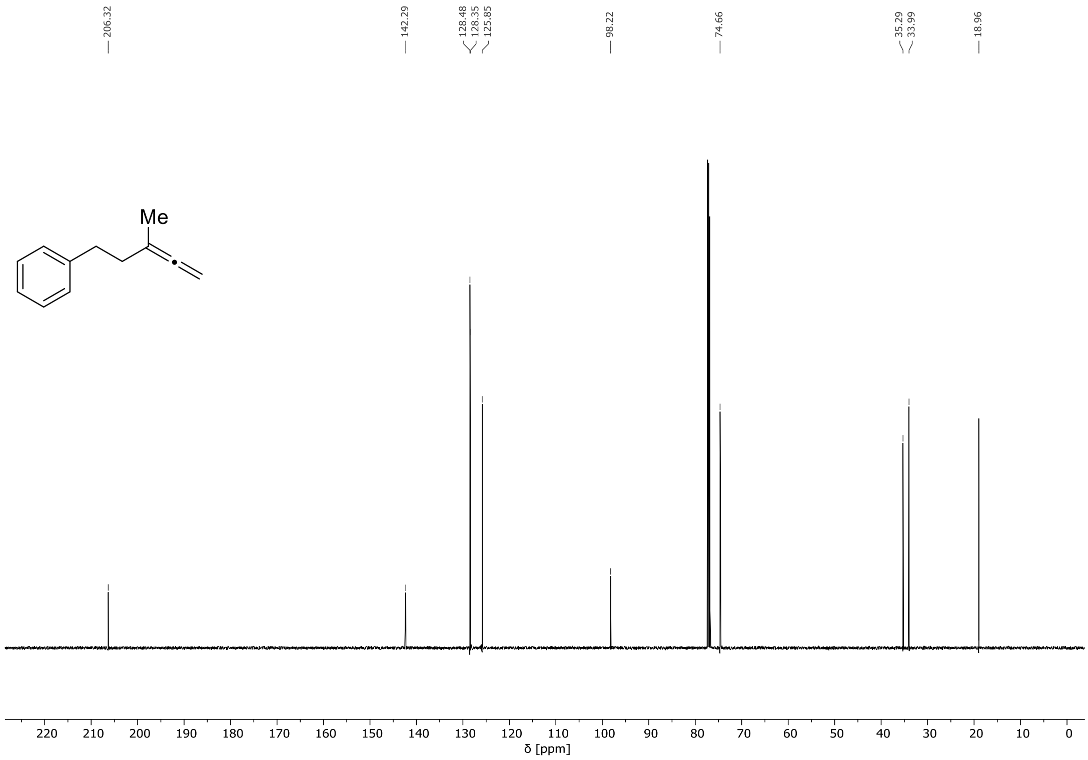

Figure 37: ^{13}C-NMR spectrum of (3-methylpenta-3,4-dien-1-yl)benzene ($CDCl_3$, 126 MHz).

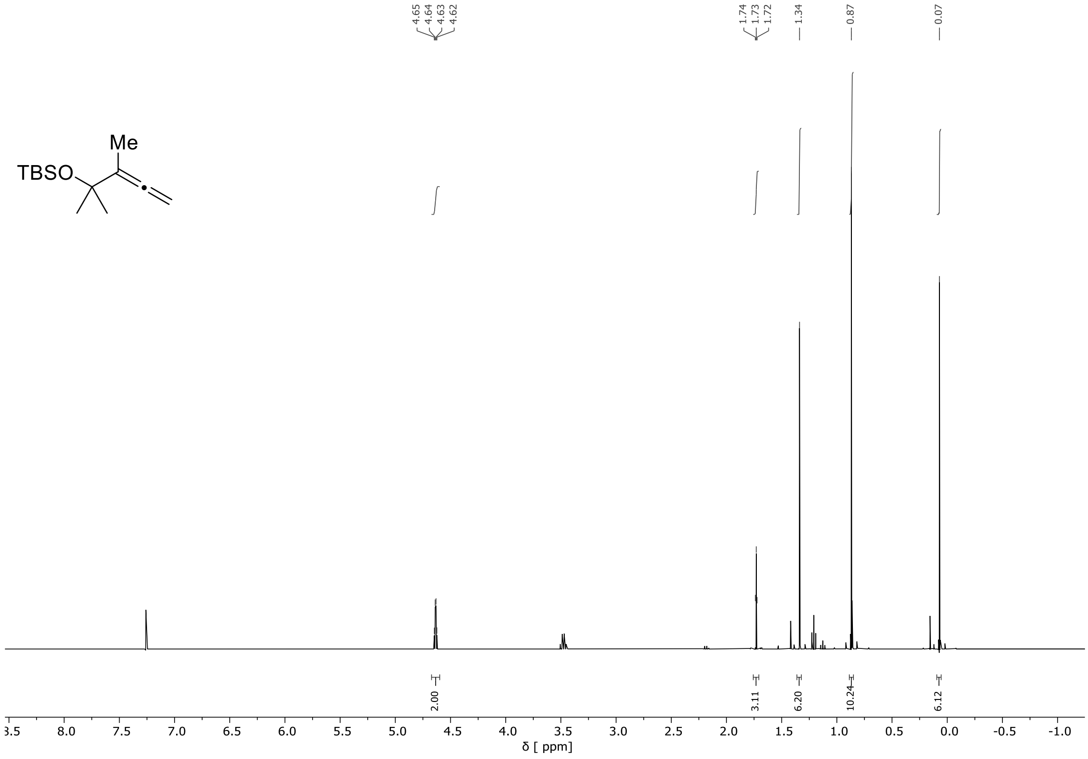

Figure 38: ^{1}H-NMR spectrum of *tert*-butyl((2,3-dimethylpenta-3,4-dien-2-yl)oxy)dimethylsilane ($CDCl_3$, 400 MHz, contains Et_2O).

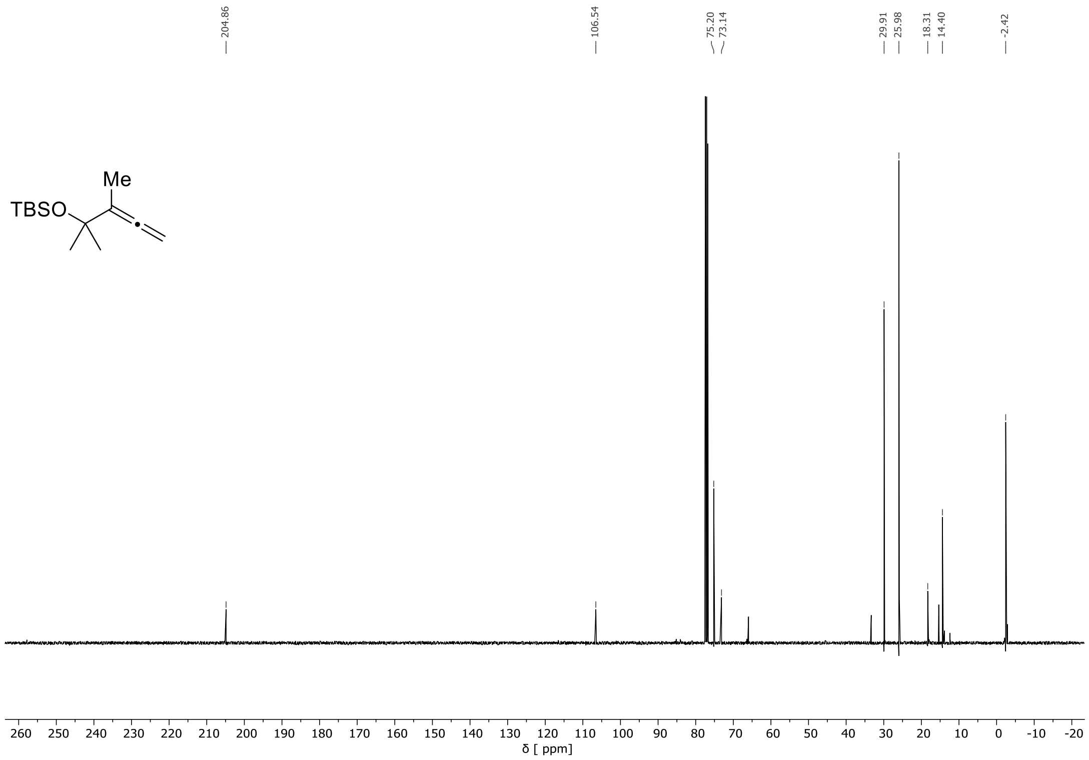

Figure 39: ^{13}C-NMR spectrum of *tert*-butyl((2,3-dimethylpenta-3,4-dien-2-yl)oxy)dimethylsilane ($CDCl_3$, 101 MHz, contains Et_2O).

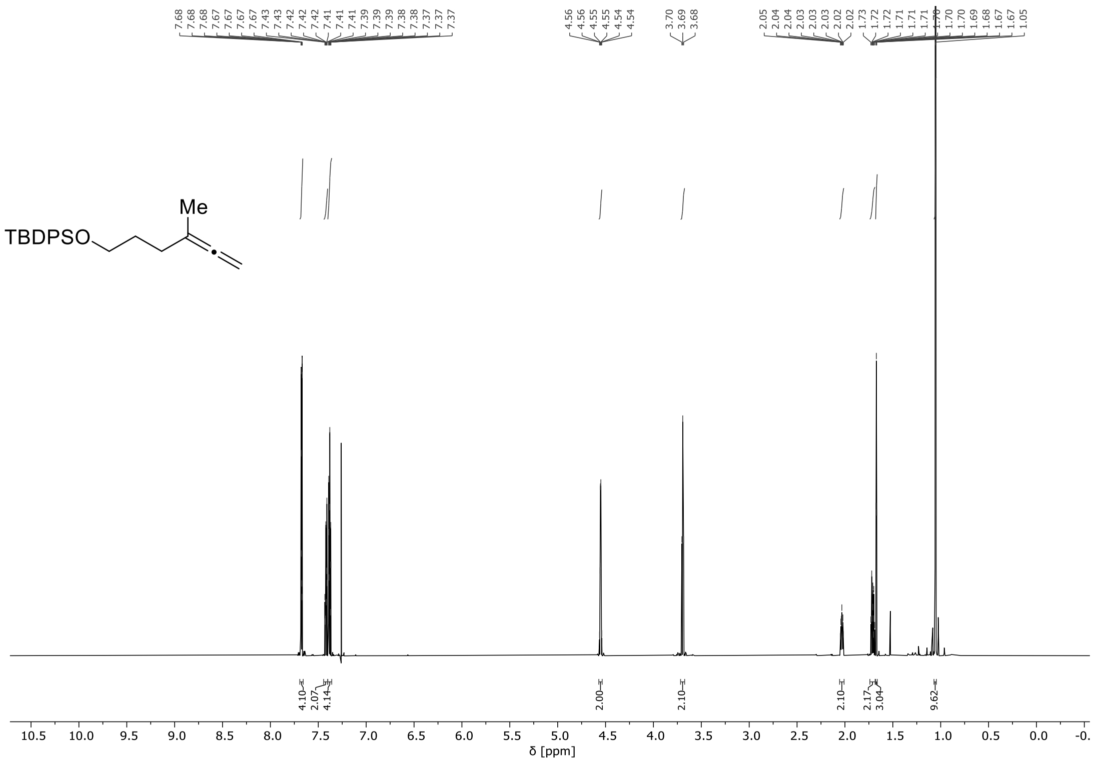

Figure 40: ^{1}H-NMR spectrum of *tert*-butyl((4-methylhexa-4,5-dien-1-yl)oxy)diphenylsilane ($CDCl_3$, 700 MHz).

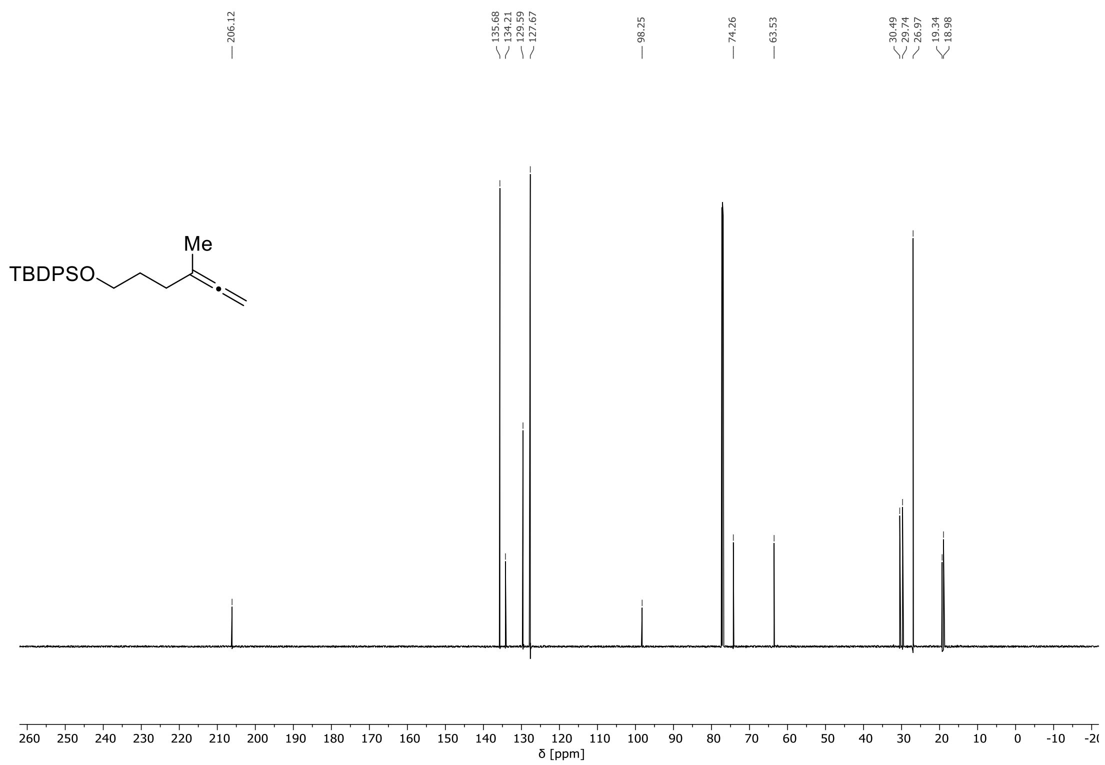

Figure 41: ^{13}C-NMR spectrum of *tert*-butyl((4-methylhexa-4,5-dien-1-yl)oxy)diphenylsilane ($CDCl_3$, 176 MHz).

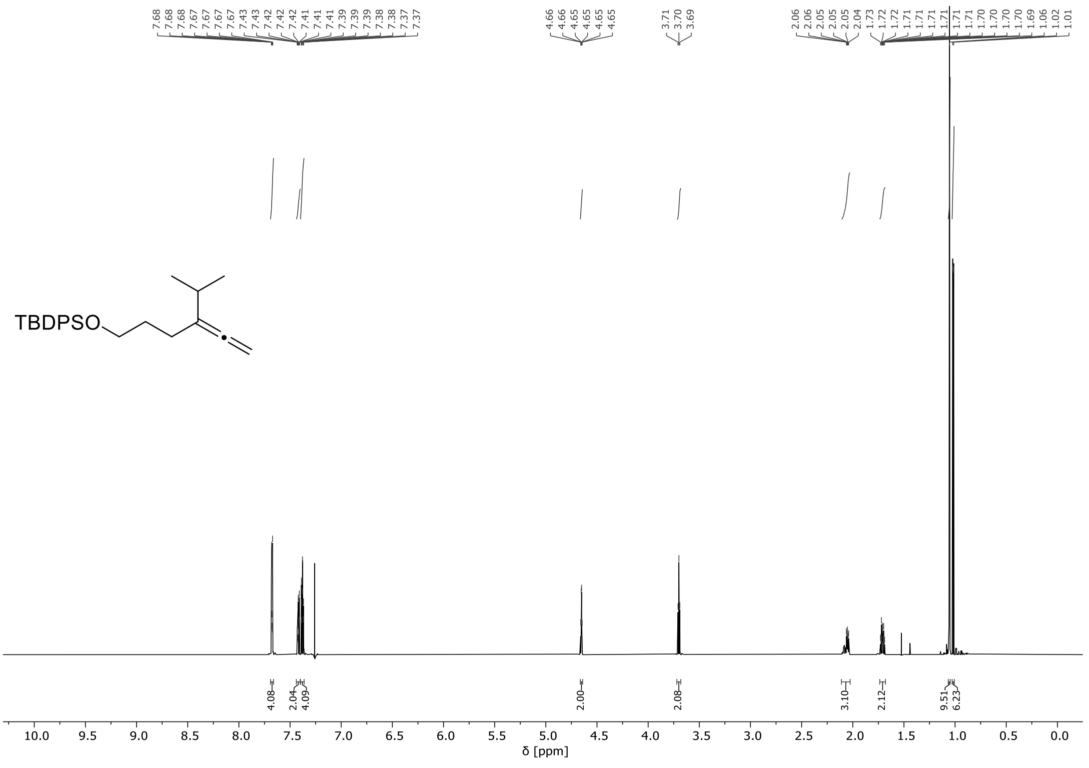

Figure 42: ^{1}H-NMR spectrum of *tert*-butyl((4-isopropylhexa-4,5-dien-1-yl)oxy)diphenylsilane ($CDCl_3$, 700 MHz).

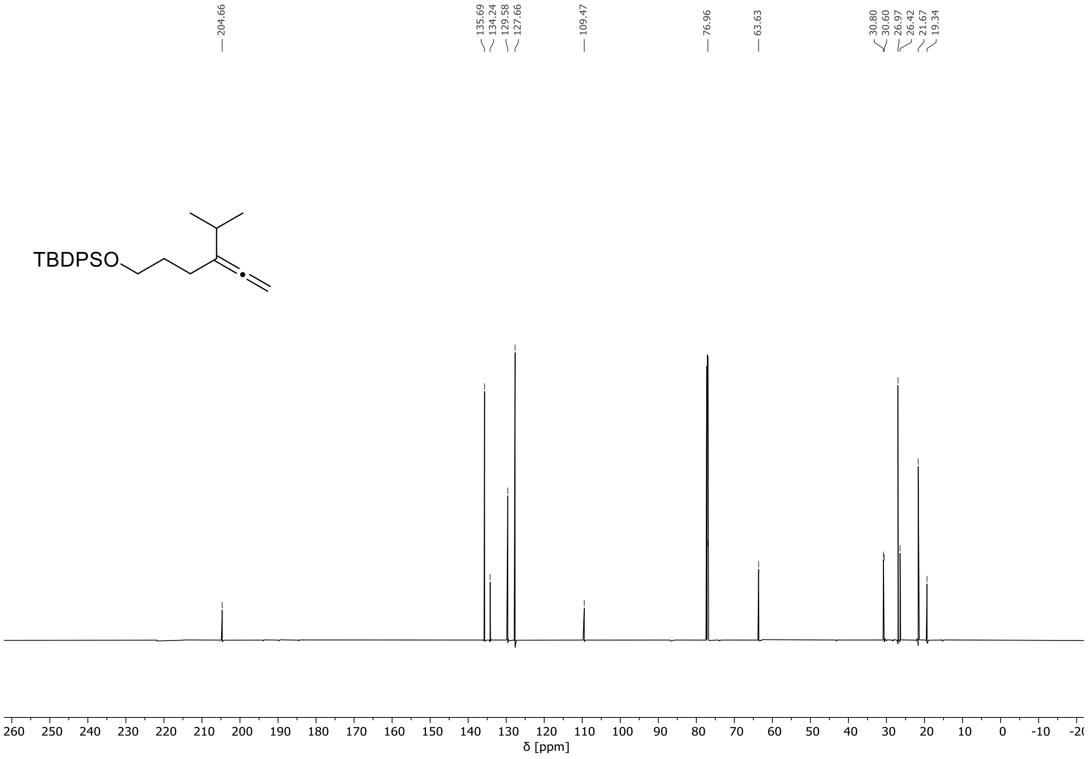

Figure 43: ^{13}C-NMR spectrum of *tert*-butyl((4-isopropylhexa-4,5-dien-1-yl)oxy)diphenylsilane ($CDCl_3$, 176 MHz).

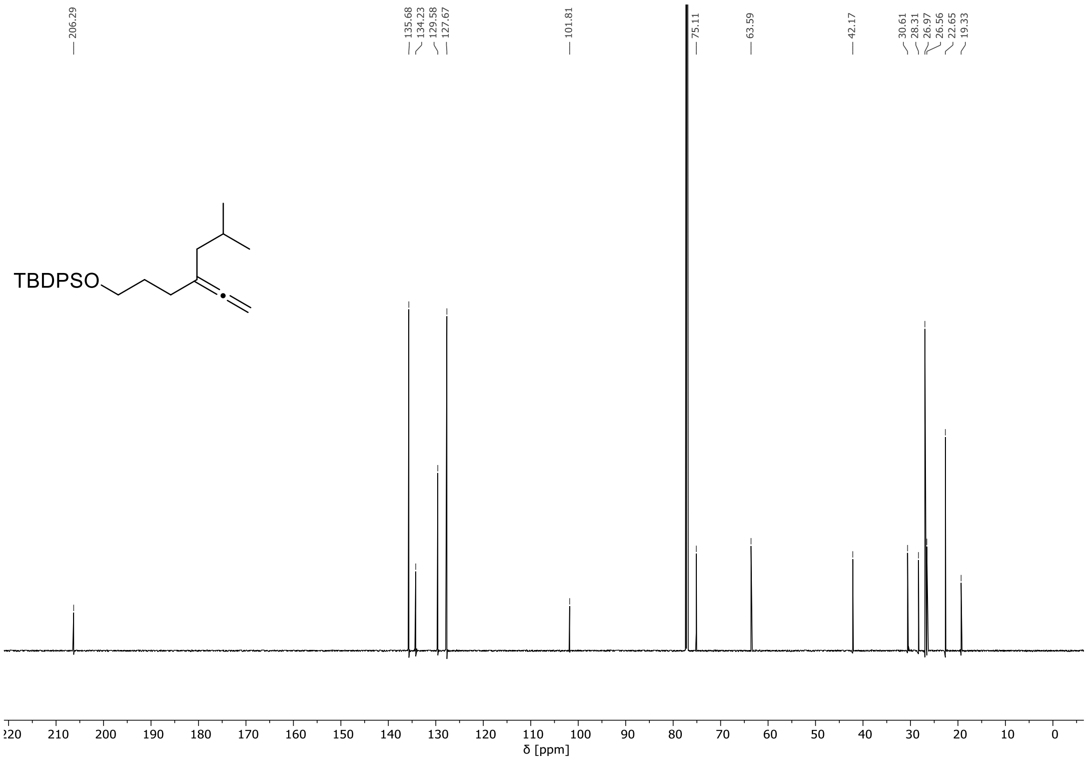

Figure 44: ^{1}H-NMR spectrum of *tert*-butyl((6-methyl-4-vinylideneheptyl)oxy)diphenylsilane ($CDCl_3$, 700 MHz).

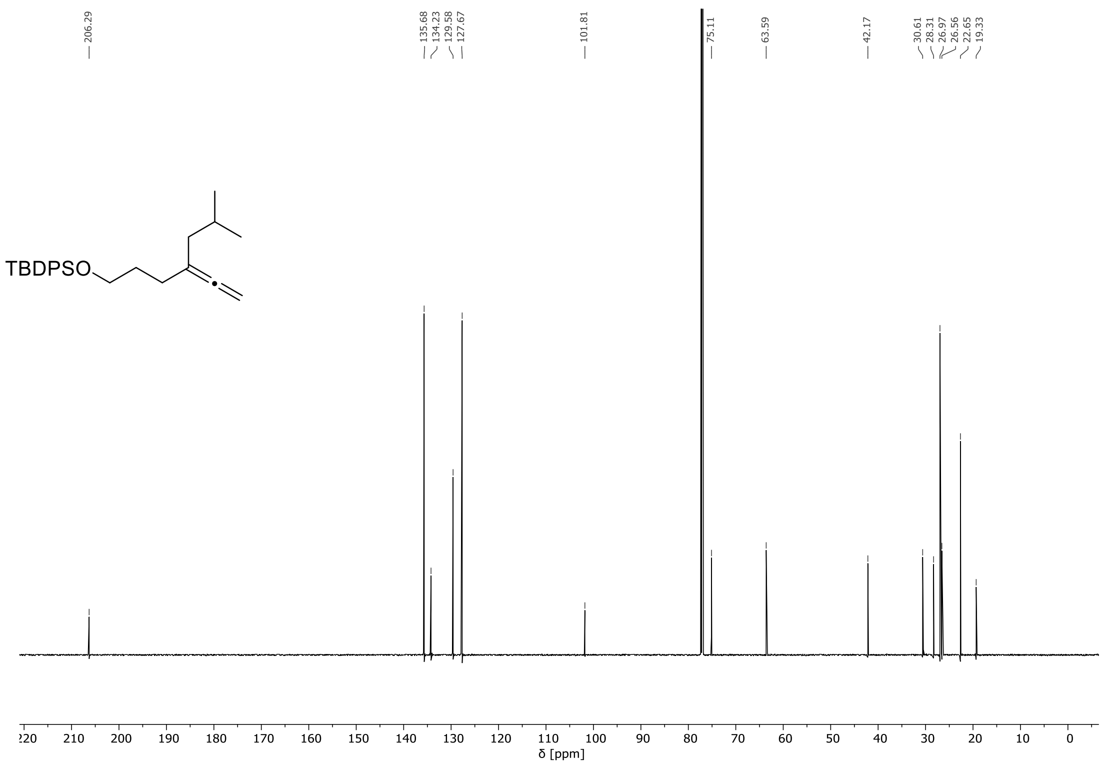

Figure 45: ^{13}C-NMR spectrum of *tert*-butyl((6-methyl-4-vinylideneheptyl)oxy)diphenylsilane ($CDCl_3$, 176 MHz).

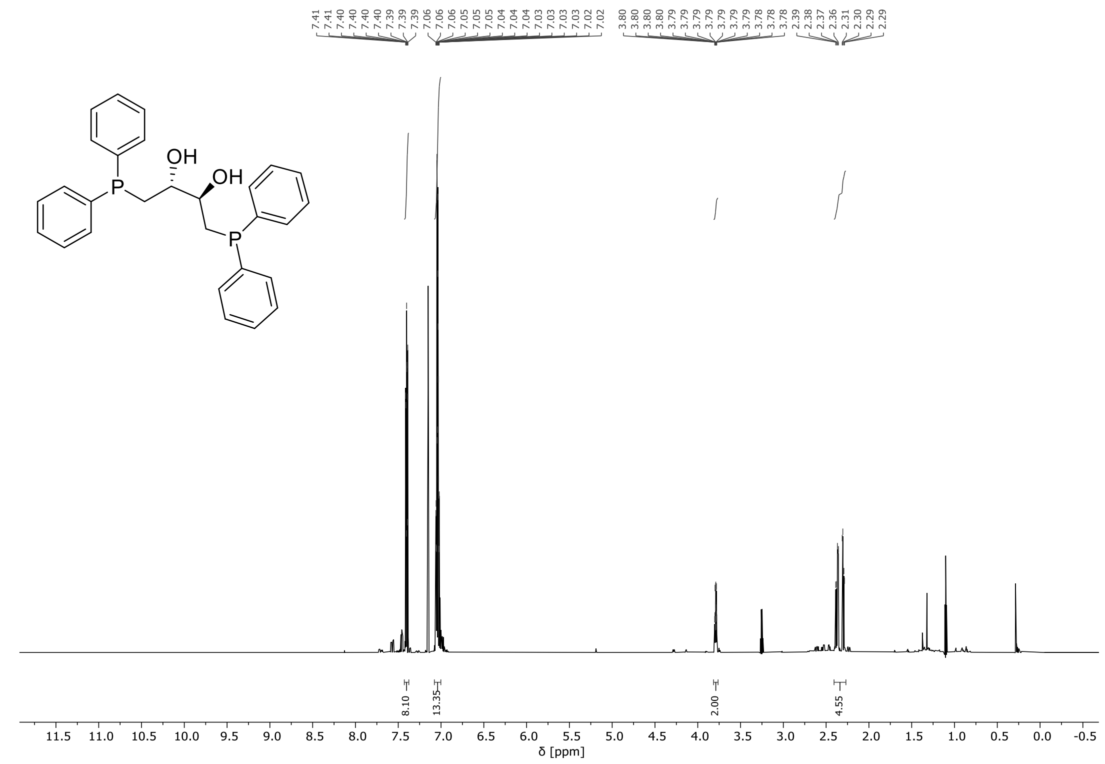

Figure 46: ^{1}H-NMR spectrum of (2*R*,3*R*)-1,4-bis(diphenylphosphaneyl)butane-2,3-diol (C_6D_6, 700 MHz, contains Et_2O).

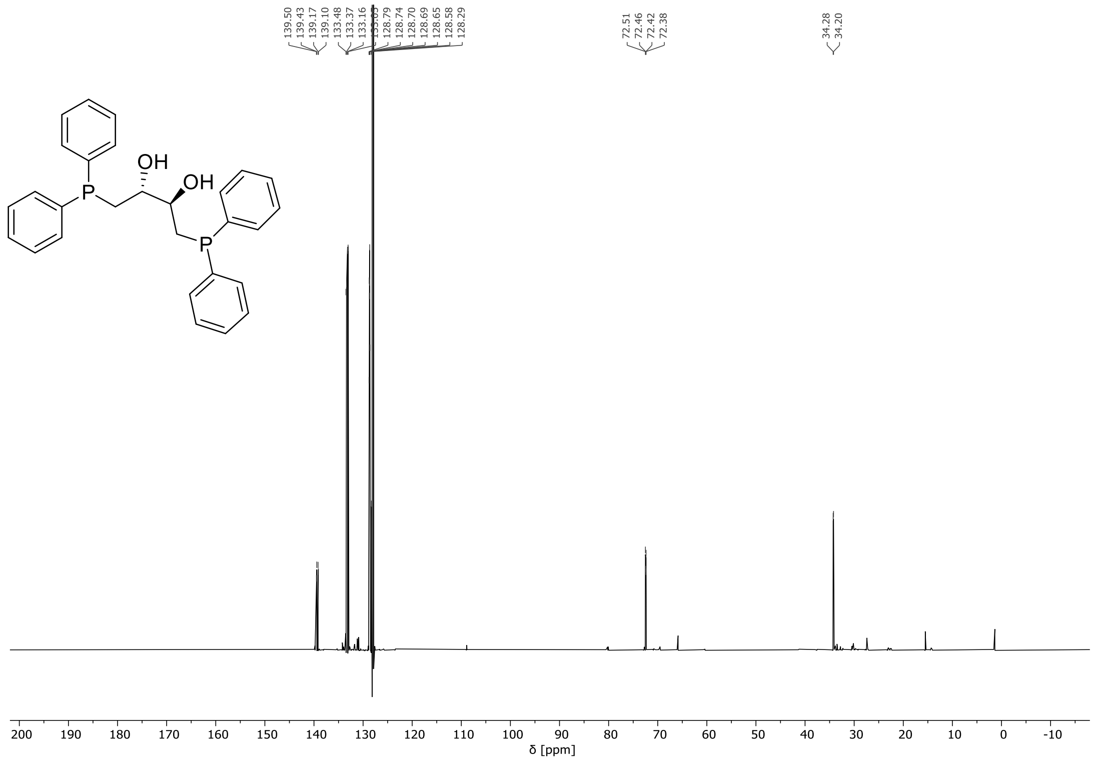

Figure 47: ^{13}C-NMR spectrum of (2*R*,3*R*)-1,4-bis(diphenylphosphaneyl)butane-2,3-diol (C_6D_6, 176 MHz, contains Et_2O).

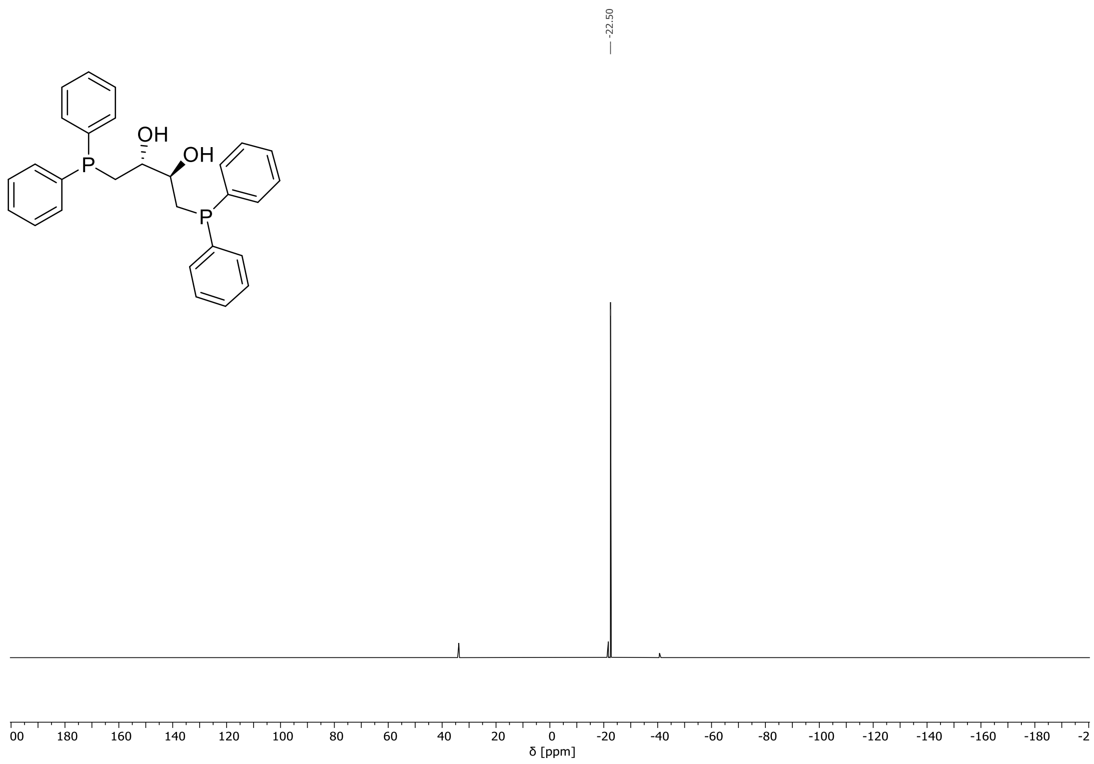

Figure 48: ^{31}P-NMR spectrum of (2*R*,3*R*)-1,4-bis(diphenylphosphaneyl)butane-2,3-diol (C_6D_6, 283 MHz).

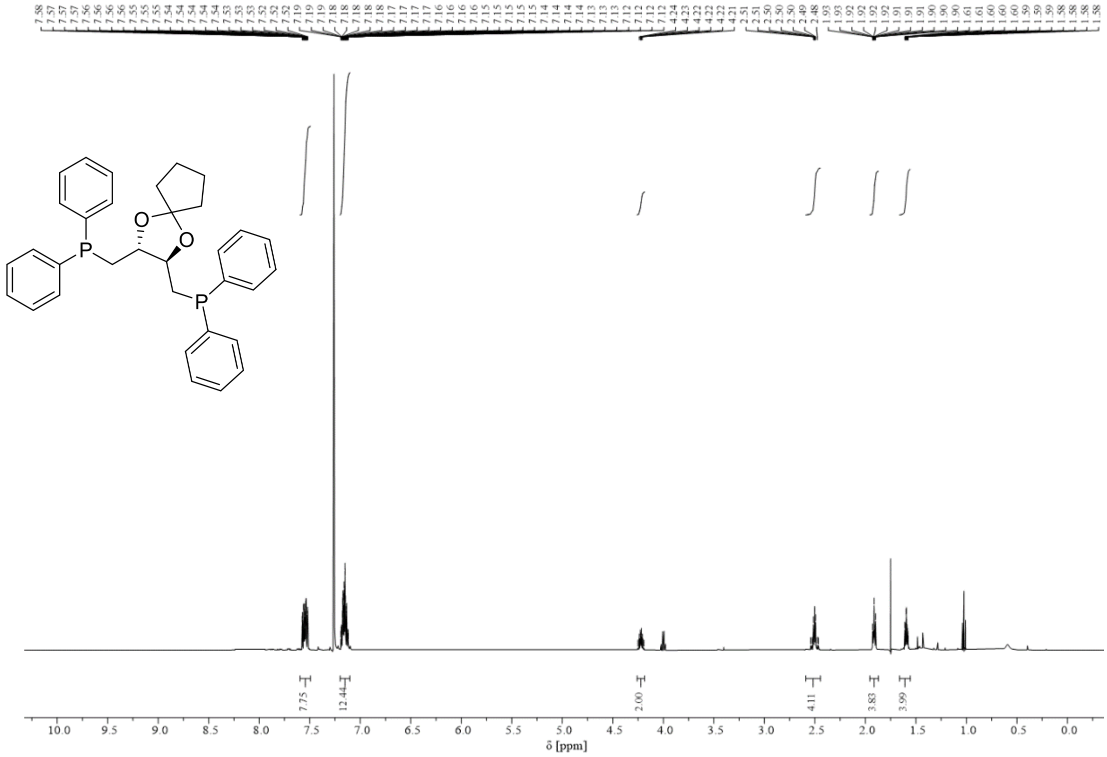

Figure 49: ^{1}H-NMR spectrum of (2*R*,3*R*)-2,3-bis((diphenylphosphaneyl)methyl)-1,4-dioxaspiro[4.4]nonane (C_6D_6, 500 MHz).

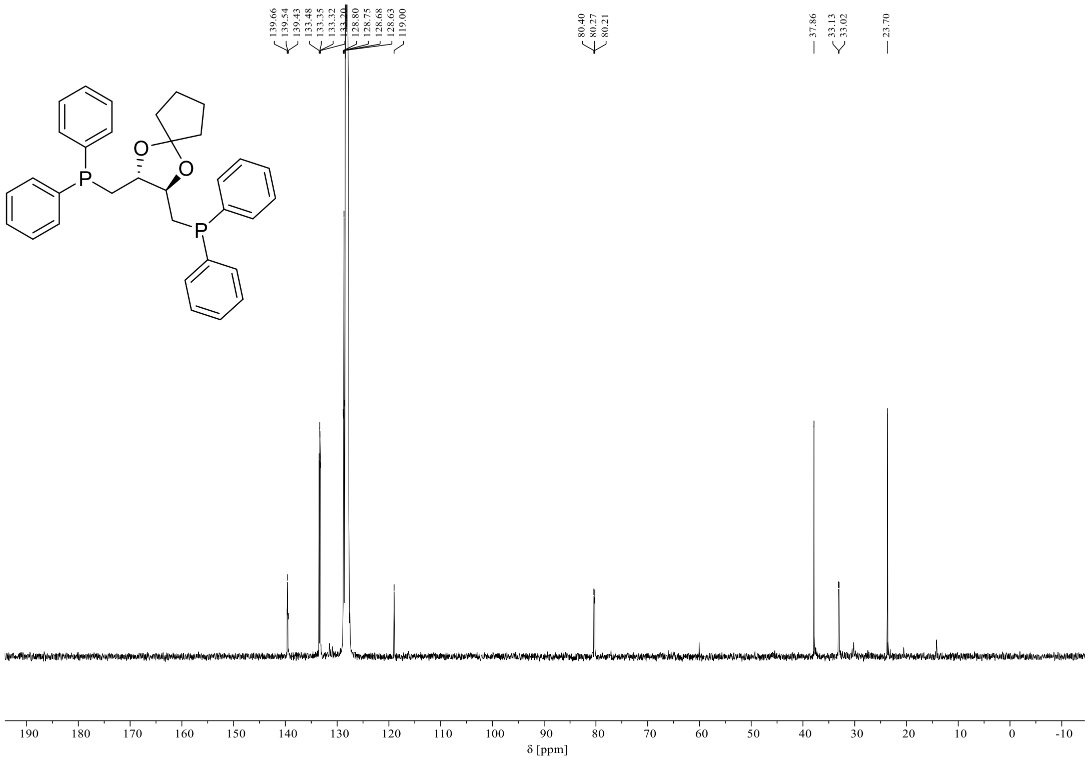

Figure 50: ^{13}C-NMR spectrum of (2*R*,3*R*)-2,3-bis((diphenylphosphaneyl)methyl)-1,4-dioxaspiro[4.4]nonane (C_6D_6, 126 MHz).

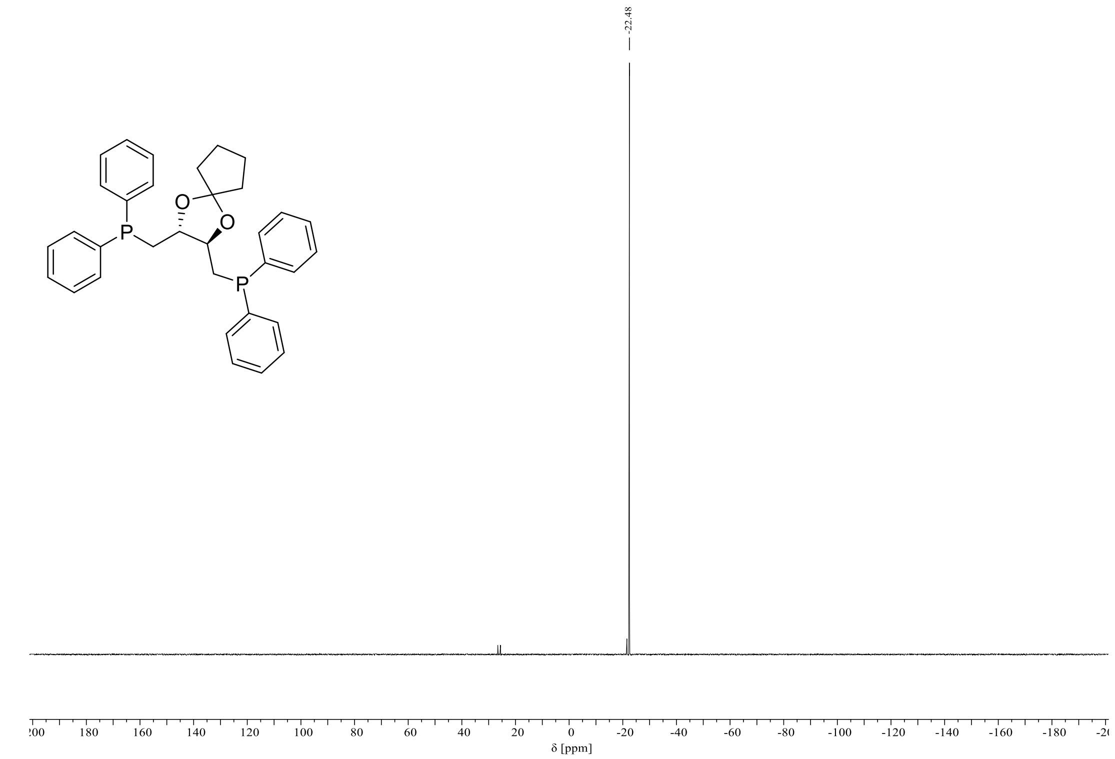

Figure 51: ^{31}P-NMR spectrum of (2*R*,3*R*)-2,3-bis((diphenylphosphaneyl)methyl)-1,4-dioxaspiro[4.4]nonane (C_6D_6, 203 MHz).

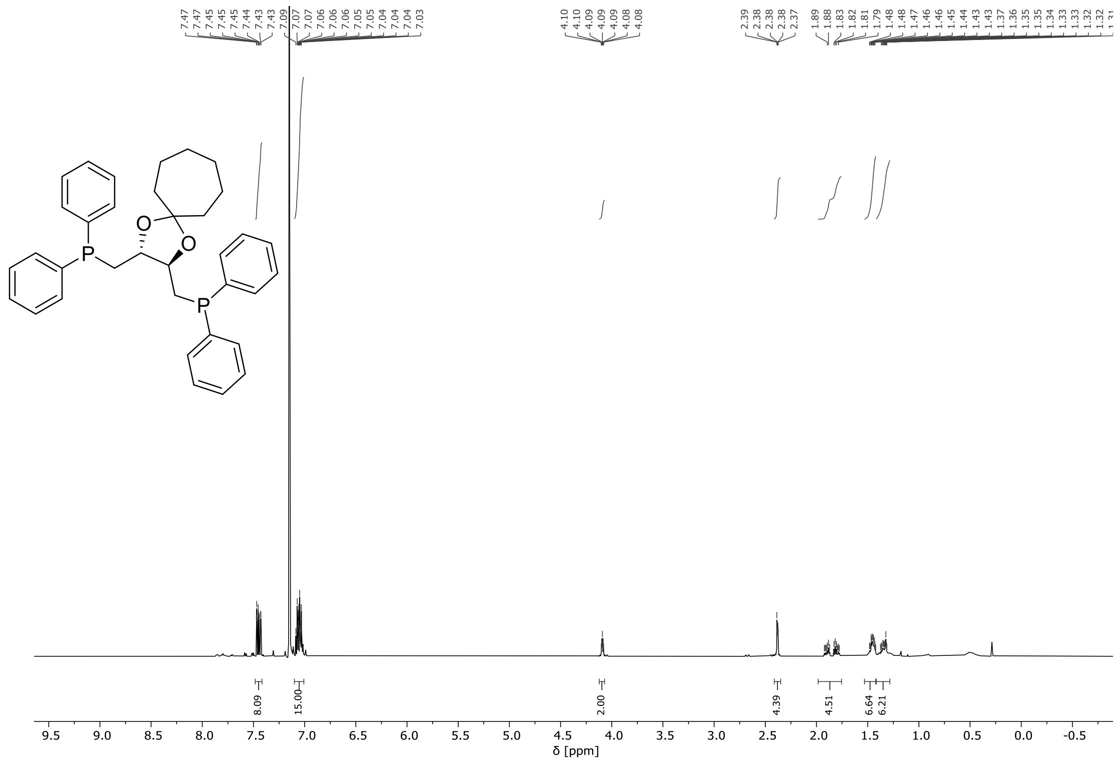

Figure 52: ^{1}H-NMR spectrum of (2*R*,3*R*)-2,3-bis((diphenylphosphaneyl)methyl)-1,4-dioxaspiro[4.6]undecane (C_6D_6, 500 MHz).

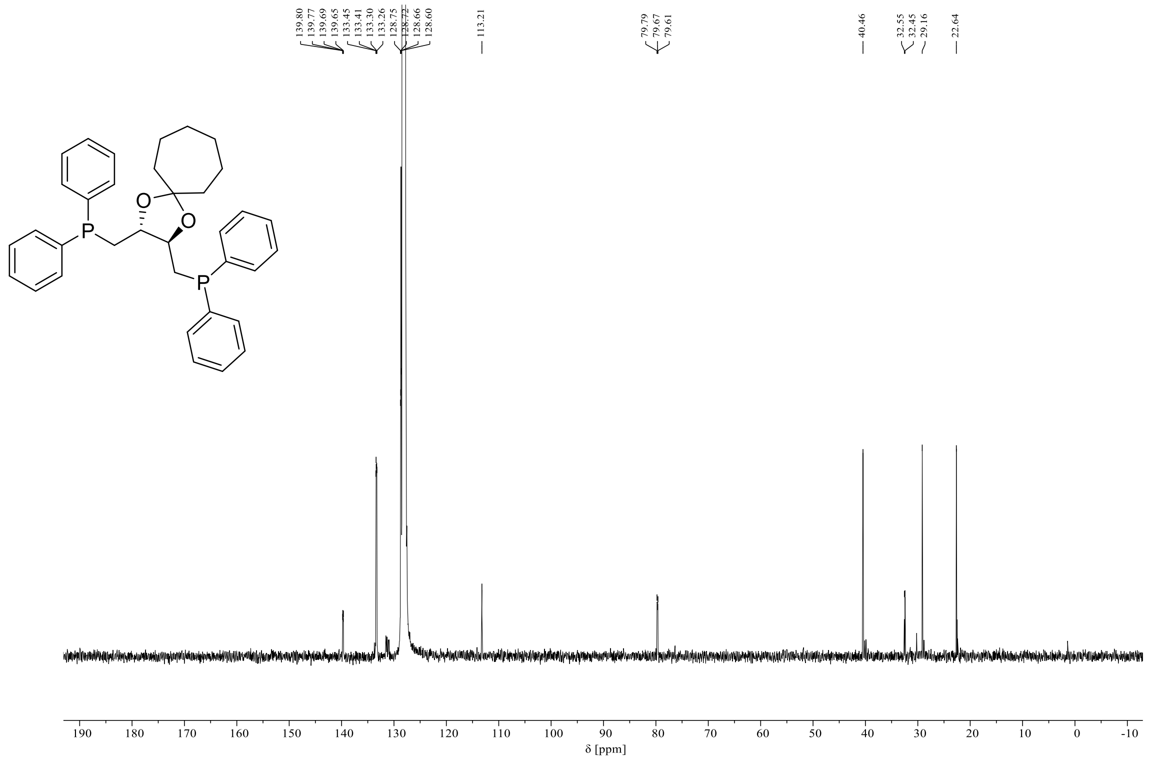

Figure 53: ^{13}C-NMR spectrum of (2*R*,3*R*)-2,3-bis((diphenylphosphaneyl)methyl)-1,4-dioxaspiro[4.6]undecane (C_6D_6, 126 MHz).

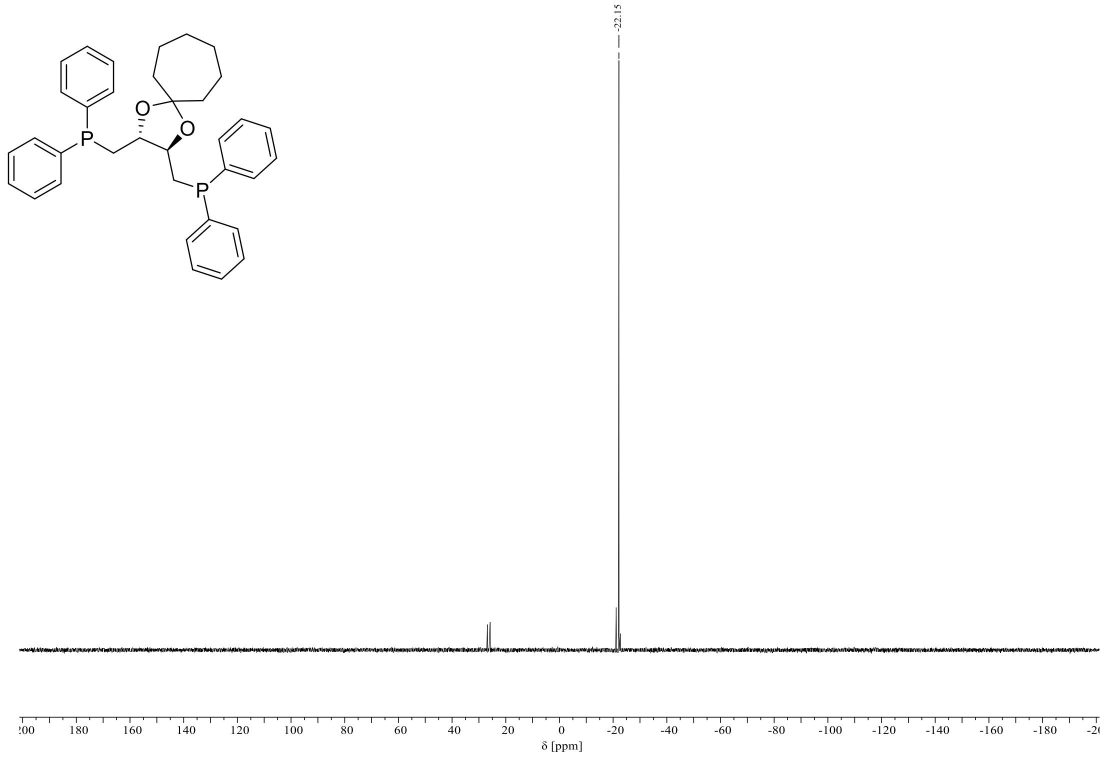

Figure 54: ^{31}P-NMR spectrum of (2*R*,3*R*)-2,3-bis((diphenylphosphaneyl)methyl)-1,4-dioxaspiro[4.6]undecane (C_6D_6, 203 MHz).

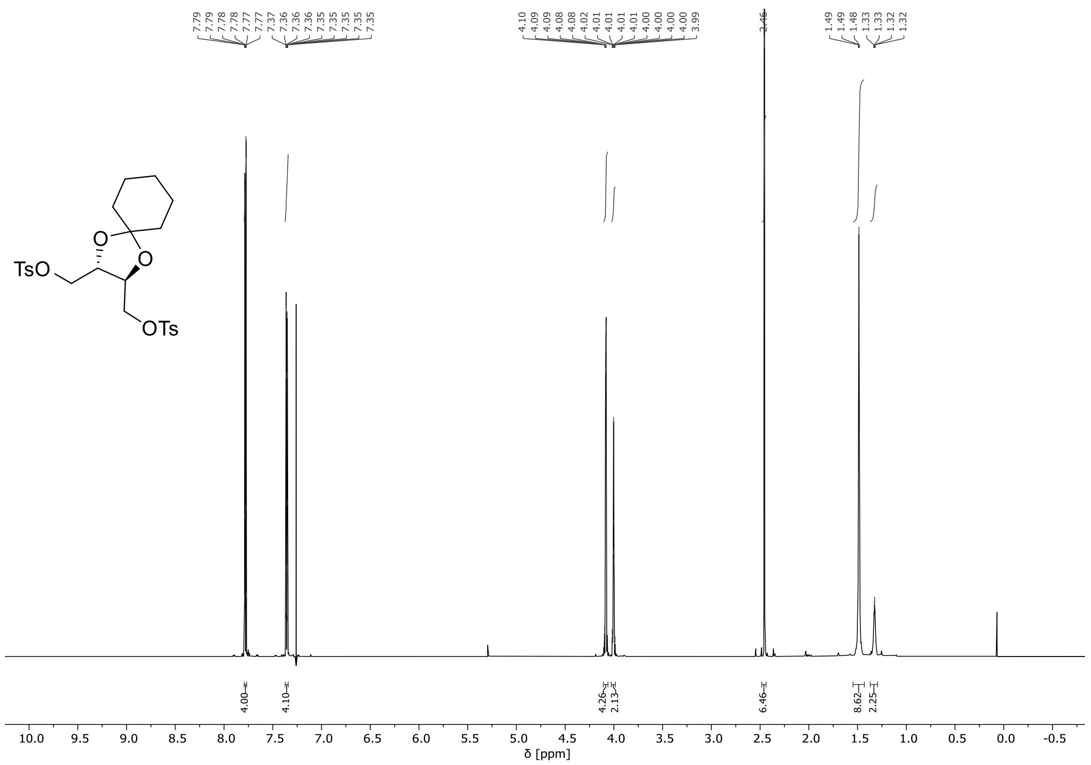

Figure 55: ^{1}H-NMR spectrum of ((2*S*,3*S*)-1,4-dioxaspiro[4.5]decane-2,3-diyl)bis(methylene) bis(4-methylbenzenesulfonate) ($CDCl_3$, 700 MHz).

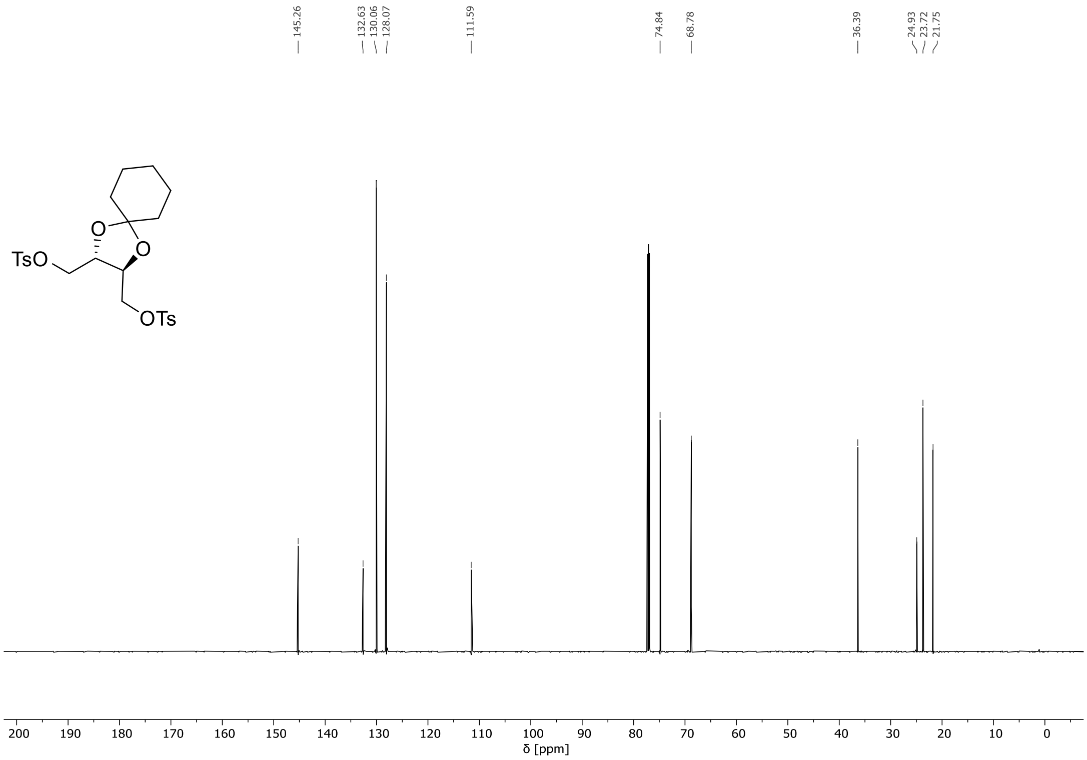

Figure 56: ^{13}C-NMR spectrum of ((2*S*,3*S*)-1,4-dioxaspiro[4.5]decane-2,3-diyl)bis(methylene) bis(4-methylbenzenesulfonate) ($CDCl_3$, 176 MHz).

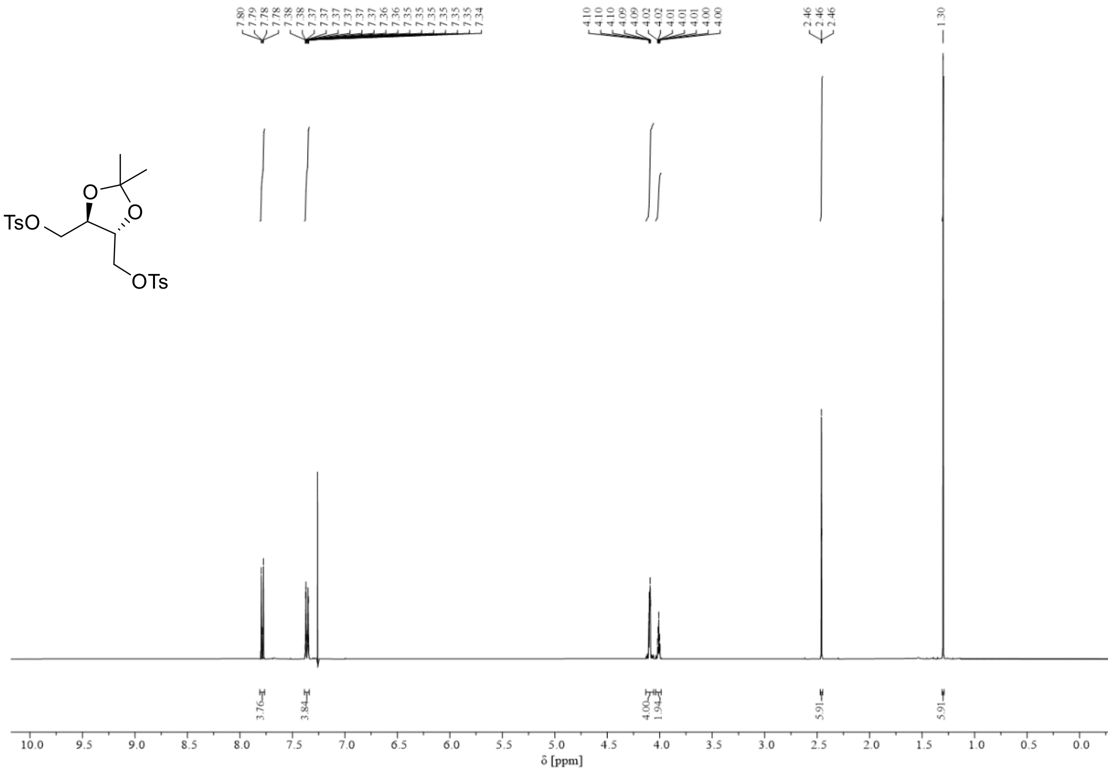

Figure 57: ^{1}H-NMR spectrum of ((4*R*,5*R*)-2,2-dimethyl-1,3-dioxolane-4,5-diyl)bis(methylene) bis(4-methylbenzenesulfonate) ($CDCl_3$, 400 MHz).

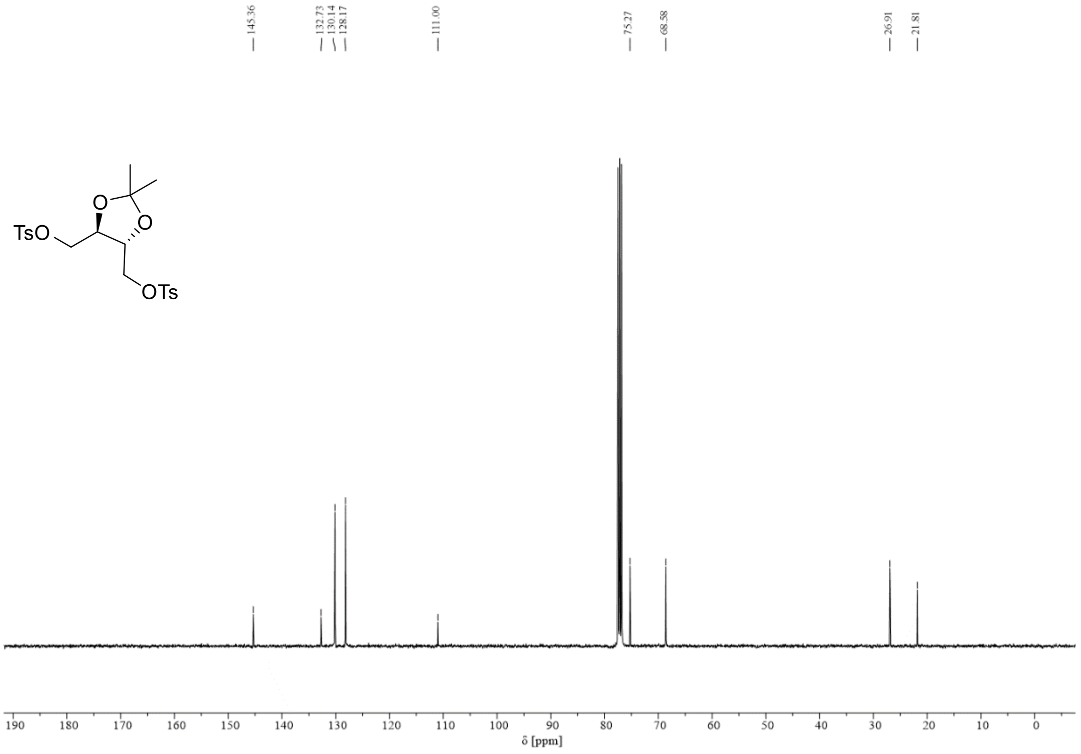

Figure 58: ^{13}C-NMR spectrum of ((4*R*,5*R*)-2,2-dimethyl-1,3-dioxolane-4,5-diyl)bis(methylene) bis(4-methylbenzenesulfonate) ($CDCl_3$, 101 MHz).

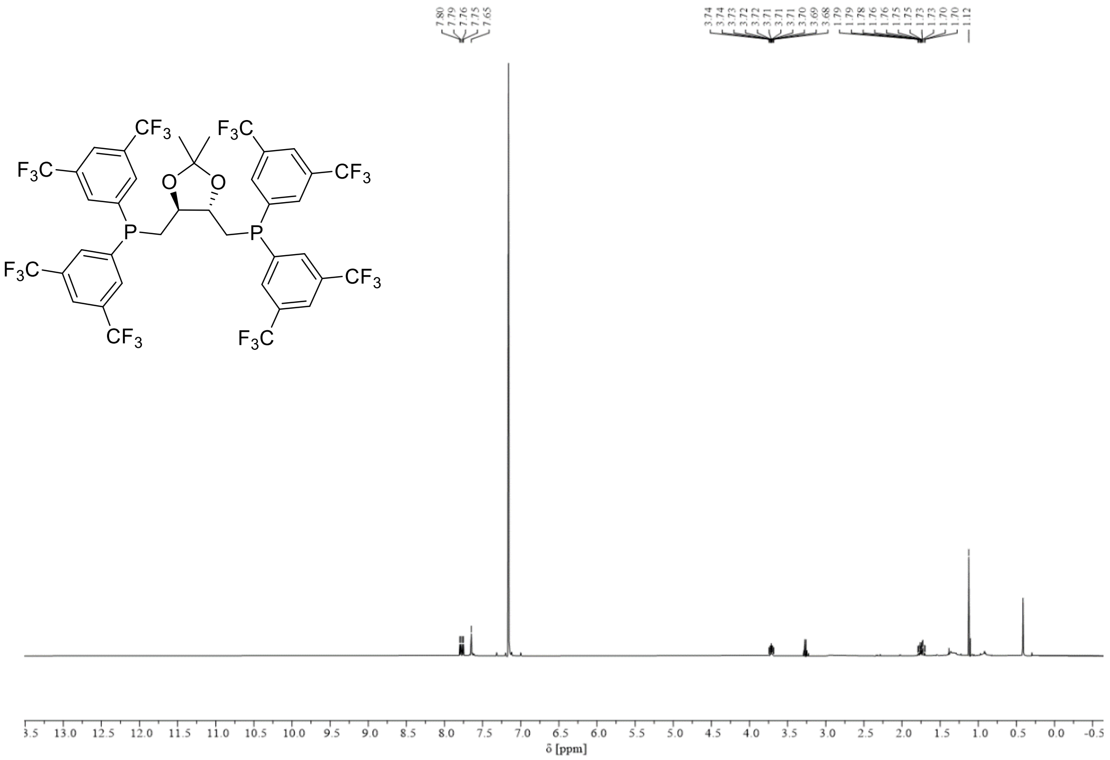

Figure 59: ^{1}H-NMR spectrum of (((4*S*,5*S*)-2,2-dimethyl-1,3-dioxolane-4,5-diyl)bis(methylene))bis(bis(3,5-bis(trifluoromethyl)-phenyl)phosphane) (C_6D_6, 500 MHz).

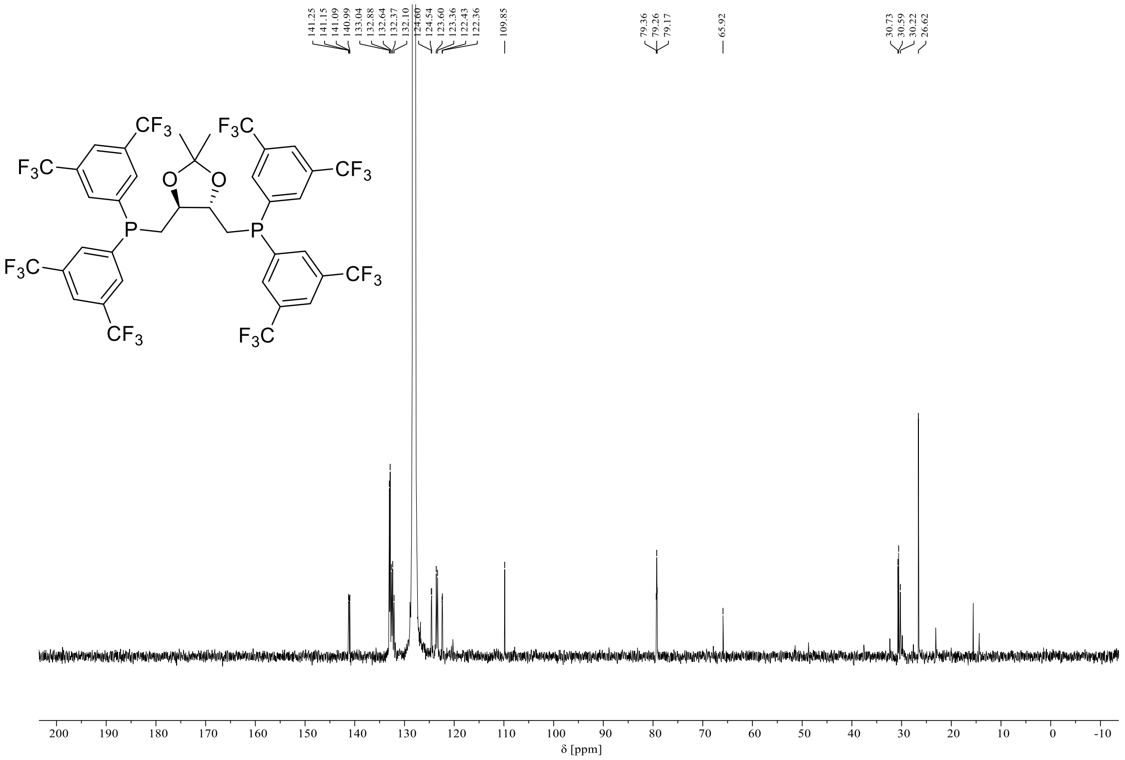

Figure 60: ^{13}C-NMR spectrum of (((4*S*,5*S*)-2,2-dimethyl-1,3-dioxolane-4,5-diyl)bis(methylene))bis(bis(3,5-bis(trifluoromethyl)-phenyl)phosphane) (C_6D_6, 126 MHz).

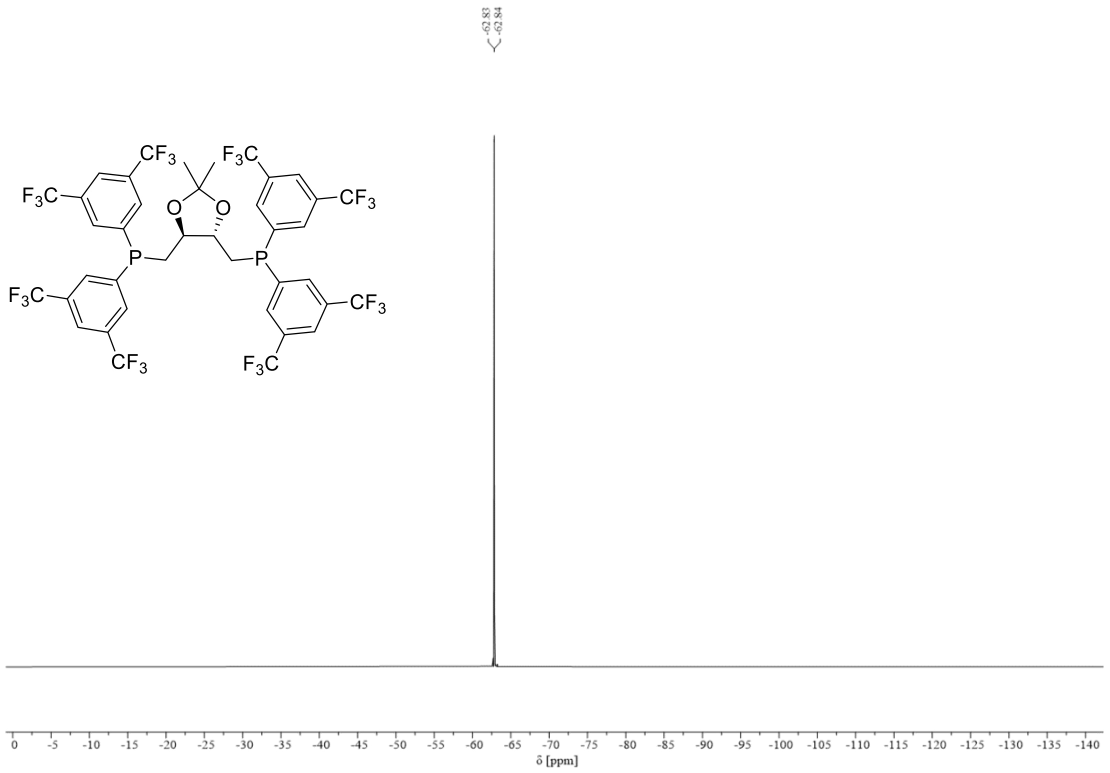

Figure 61: ^{19}F-NMR spectrum of (((4*S*,5*S*)-2,2-dimethyl-1,3-dioxolane-4,5-diyl)bis(methylene))bis(bis(3,5-bis(trifluoromethyl)-phenyl)phosphane) (C_6D_6, 471 MHz).

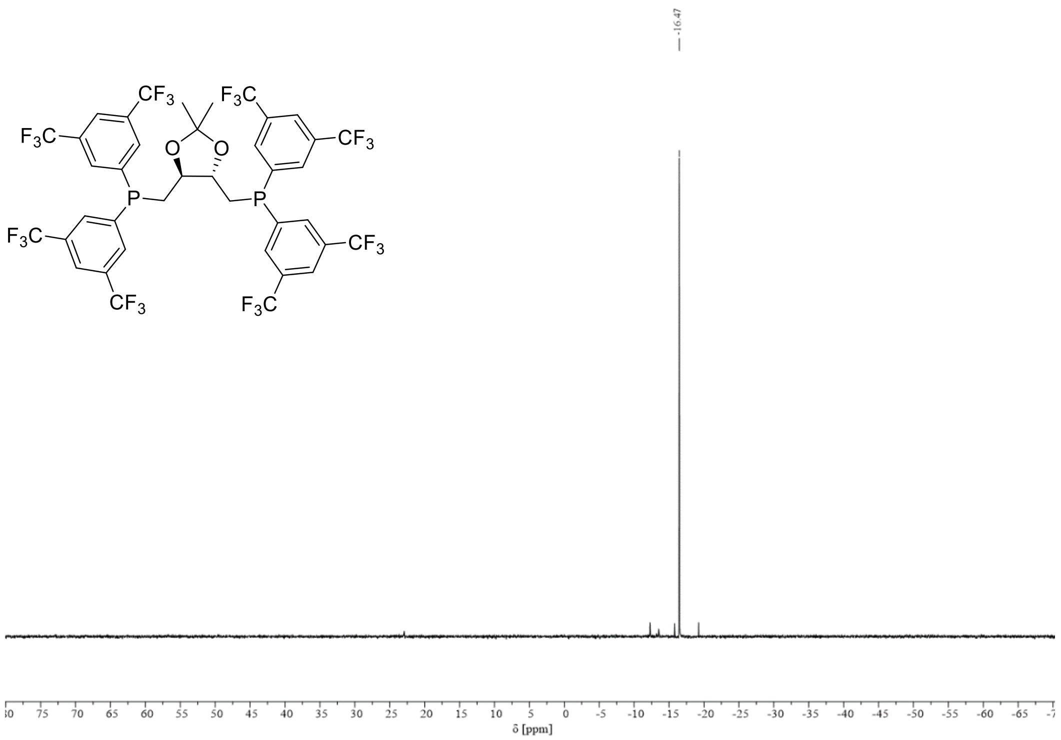

Figure 62: ^{31}P-NMR spectrum of (((4*S*,5*S*)-2,2-dimethyl-1,3-dioxolane-4,5-diyl)bis(methylene))bis(bis(3,5-bis(trifluoromethyl)-phenyl)phosphane) (C_6D_6, 203 MHz).

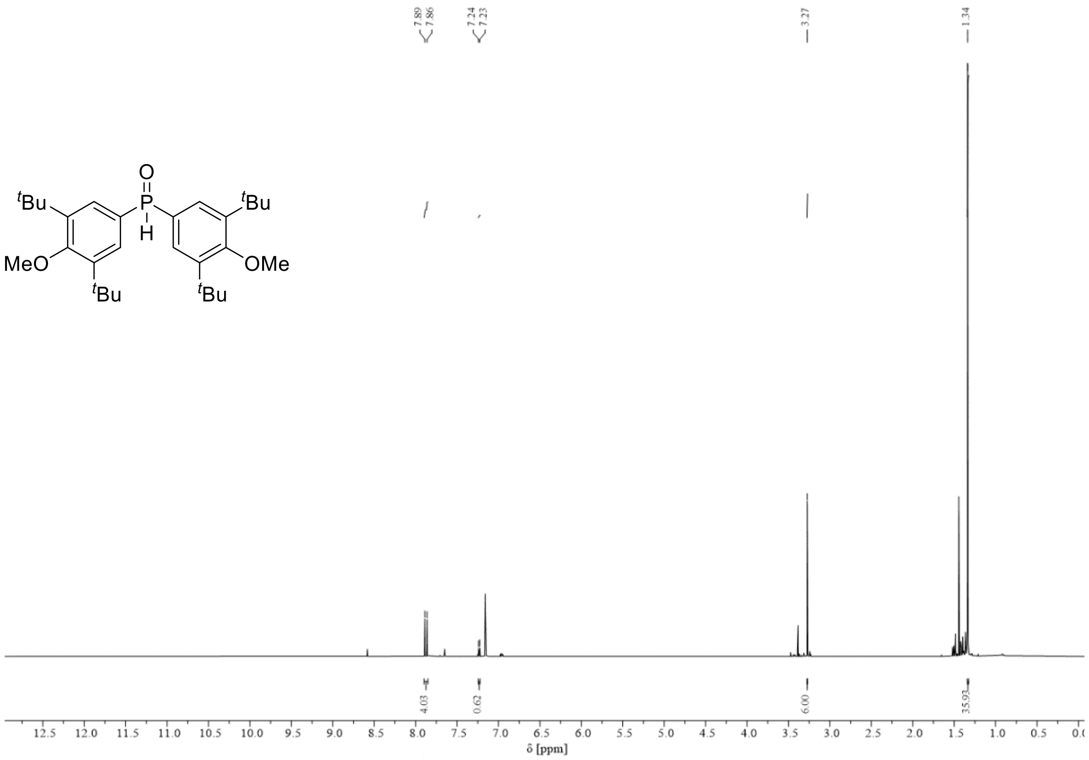

Figure 63: ^{1}H-NMR spectrum of bis(4-methoxy-3,5-dimethylphenyl)phosphine oxide (C_6D_6, 500 MHz).

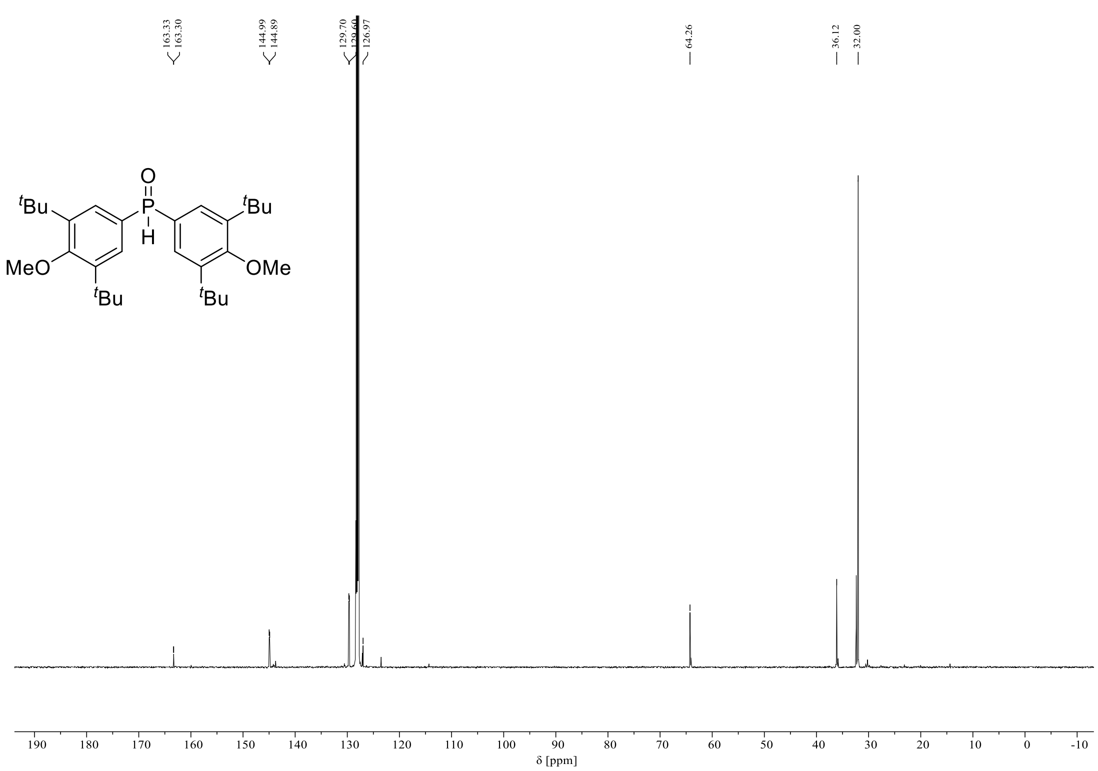

Figure 64: ^{13}C-NMR spectrum of bis(4-methoxy-3,5-dimethylphenyl)phosphine oxide (C_6D_6, 126 MHz).

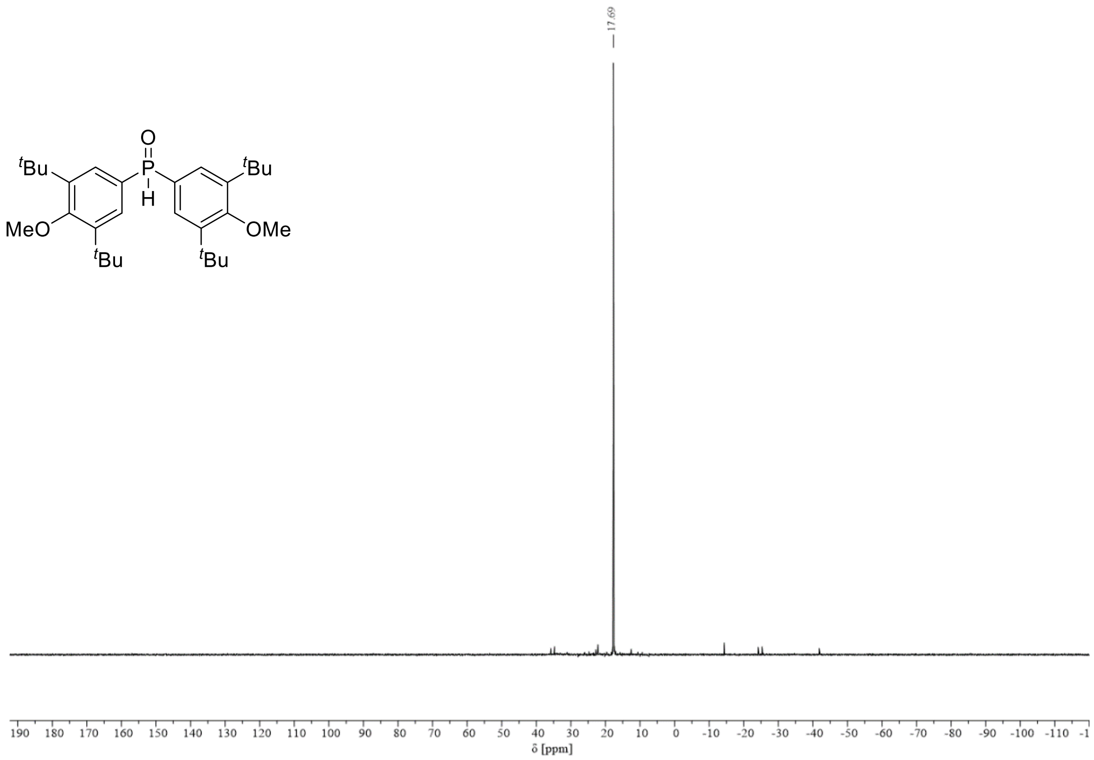

Figure 65: ^{31}P-NMR spectrum of bis(4-methoxy-3,5-dimethylphenyl)phosphine oxide (C_6D_6, 203 MHz).

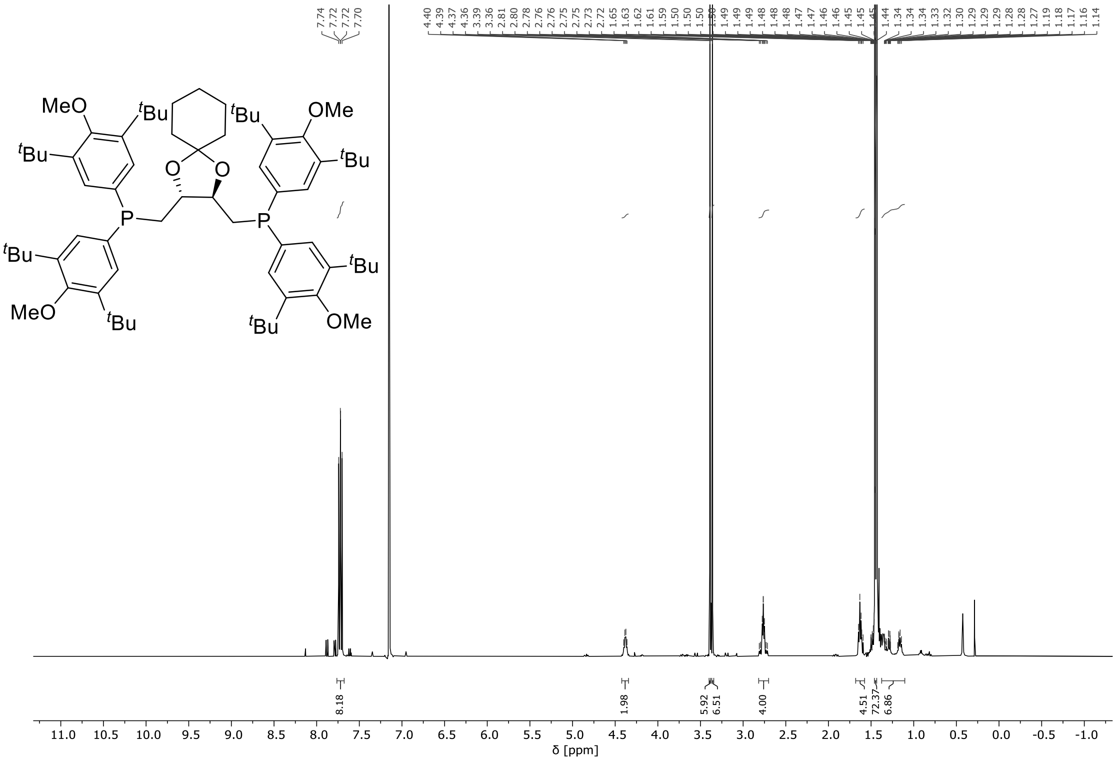

Figure 66: ^{1}H-NMR spectrum of (2*R*,3*R*)-2,3-bis((bis(3,5-di-tert-butyl-4-methoxyphenyl)phosphaneyl)methyl)-1,4-dioxaspiro-[4.5]decane (C_6D_6, 400 MHz).

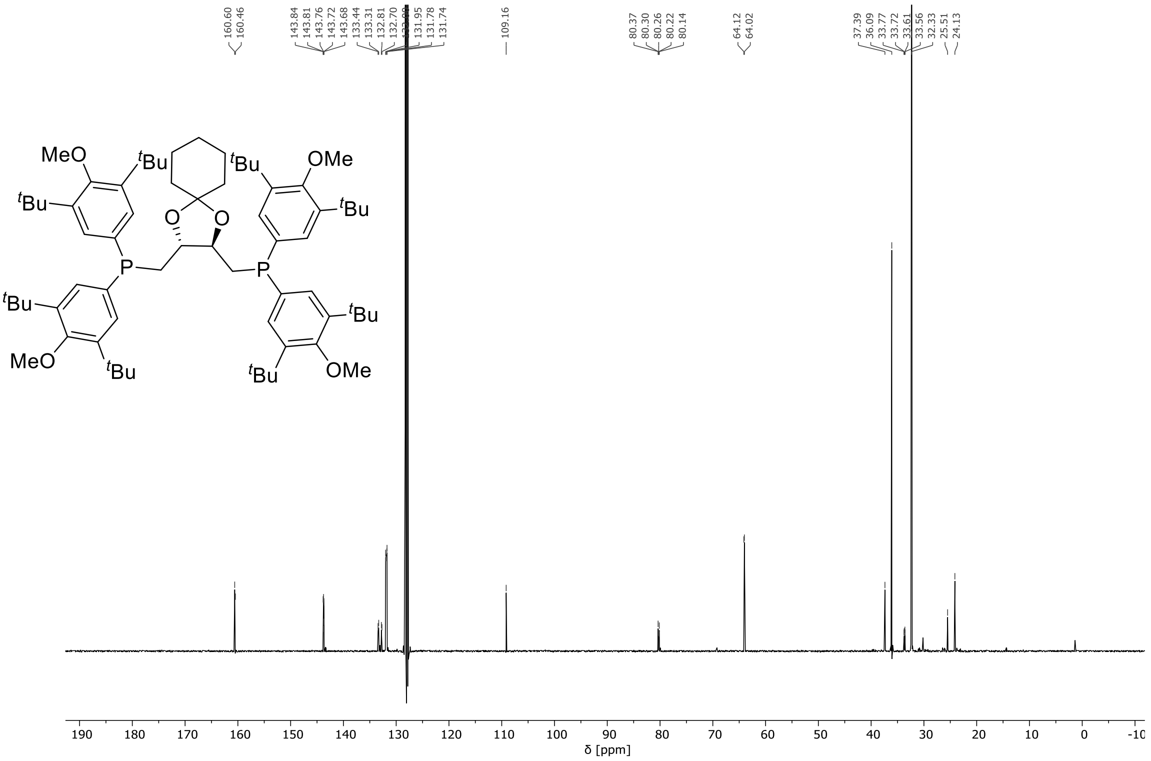

Figure 67: ^{13}C-NMR spectrum of (2*R*,3*R*)-2,3-bis((bis(3,5-di-tert-butyl-4-methoxyphenyl)phosphaneyl)methyl)-1,4-dioxaspiro-[4.5]decane (C_6D_6, 101 MHz).

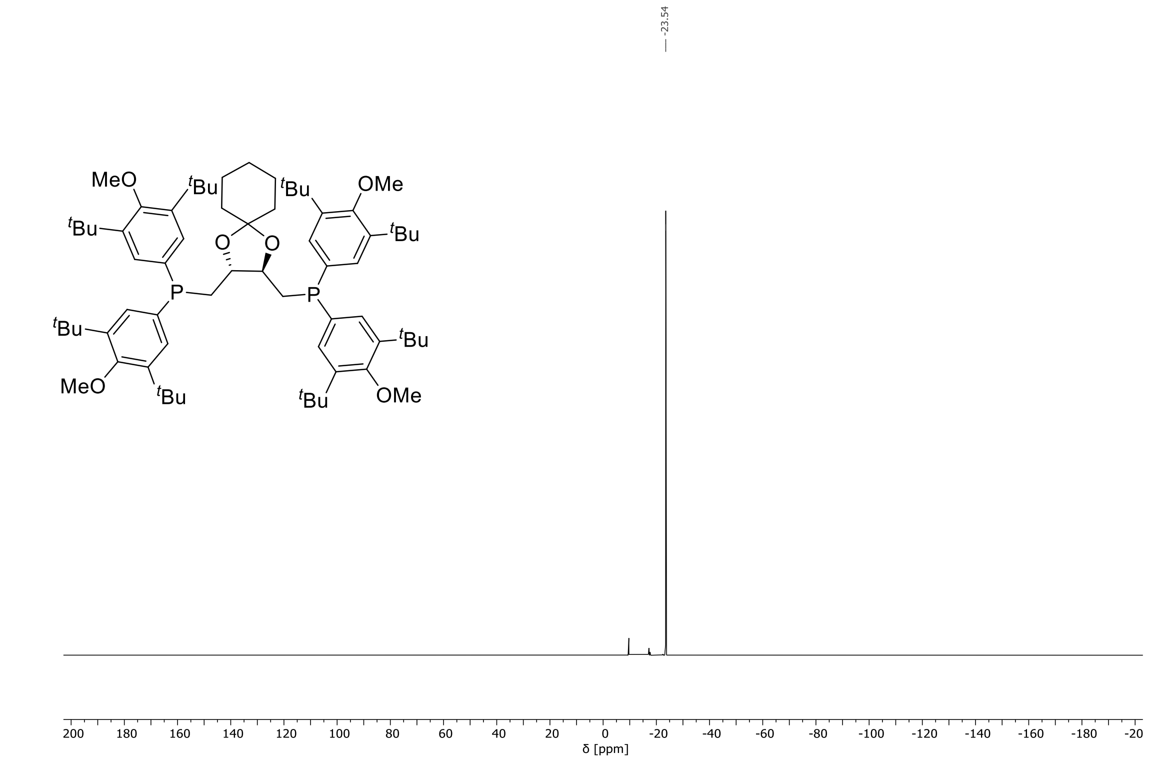

Figure 68: ^{31}P-NMR spectrum of (2*R*,3*R*)-2,3-bis((bis(3,5-di-tert-butyl-4-methoxyphenyl)phosphaneyl)methyl)-1,4-dioxaspiro-[4.5]decane (C_6D_6, 162 MHz).

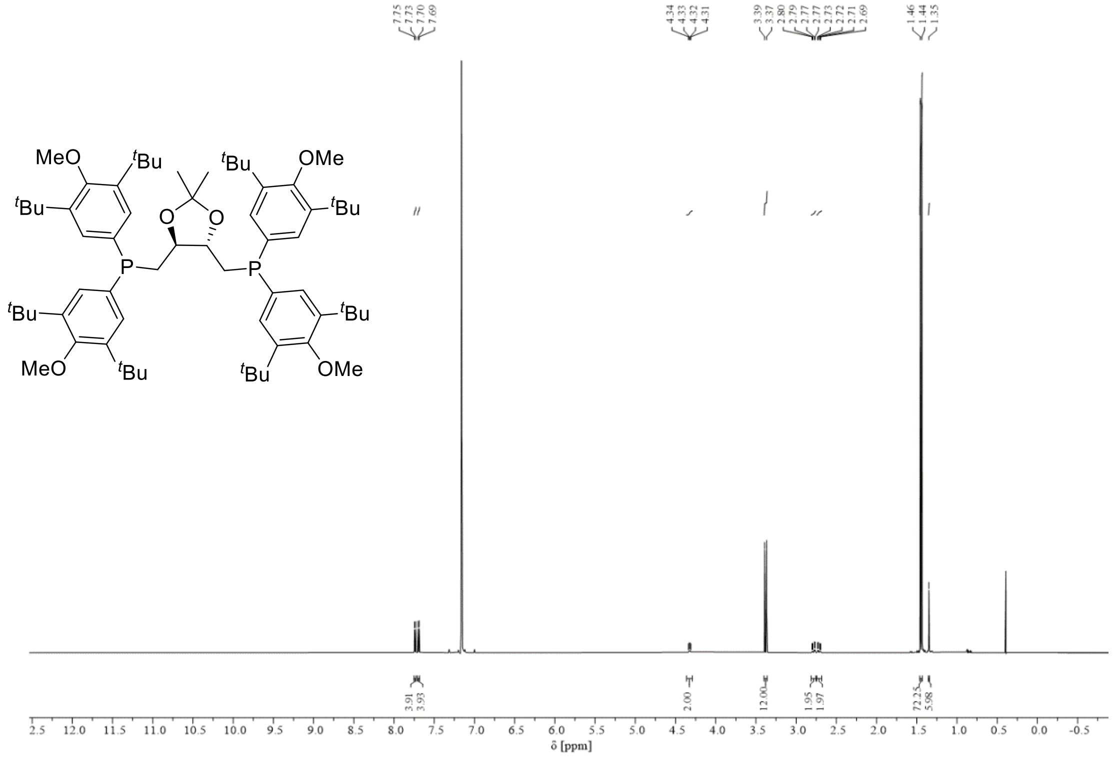

Figure 69: ^{1}H-NMR spectrum of (((4*S*,5*S*)-2,2-dimethyl-1,3-dioxolane-4,5-diyl)bis(methylene))bis(bis(3,5-di-*tert*-butyl-4-methoxyphenyl)phosphane) (C_6D_6, 500 MHz).

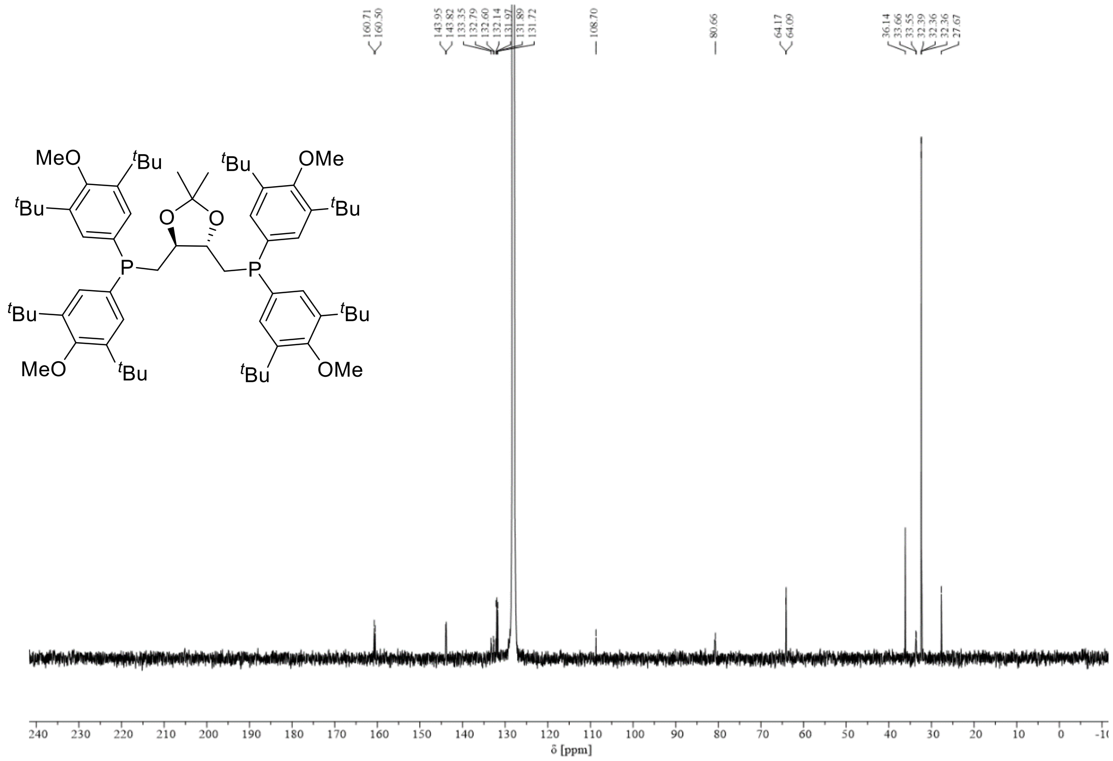

Figure 70: ^{13}C-NMR spectrum of (((4*S*,5*S*)-2,2-dimethyl-1,3-dioxolane-4,5-diyl)bis(methylene))bis(bis(3,5-di-*tert*-butyl-4-methoxyphenyl)phosphane) (C_6D_6, 126 MHz).

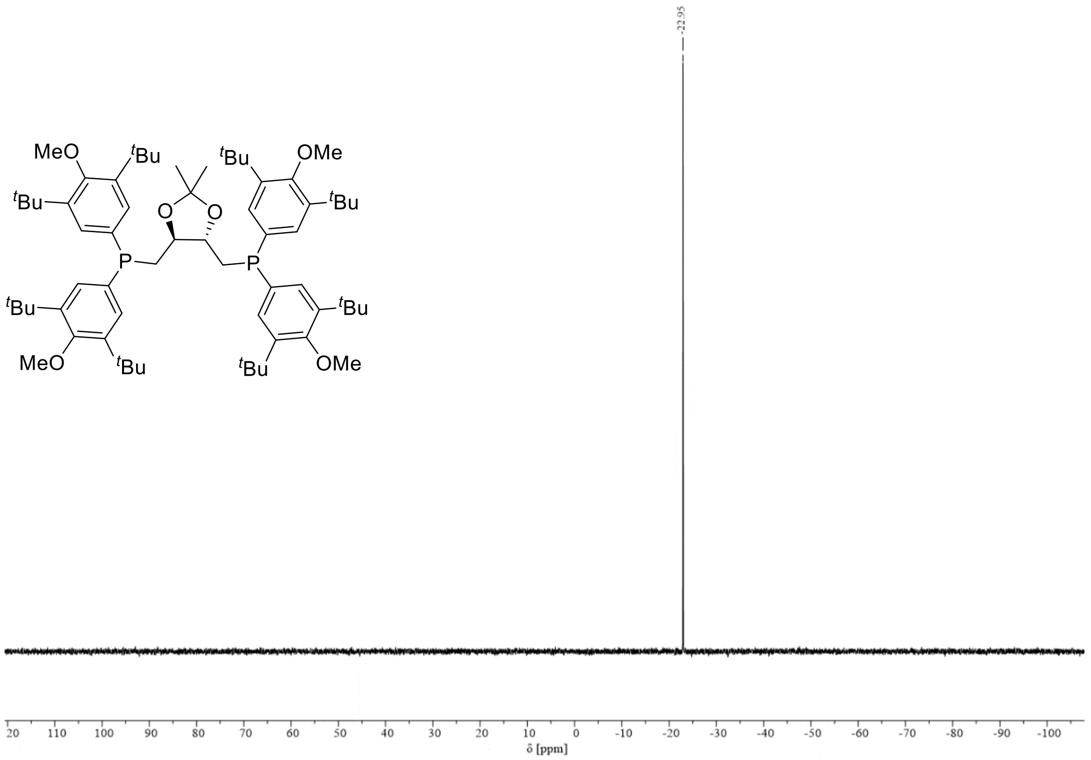

Figure 71: ^{31}P-NMR spectrum of (((4*S*,5*S*)-2,2-dimethyl-1,3-dioxolane-4,5-diyl)bis(methylene))bis(bis(3,5-di-*tert*-butyl-4-methoxyphenyl)phosphane) (C_6D_6, 203 MHz).

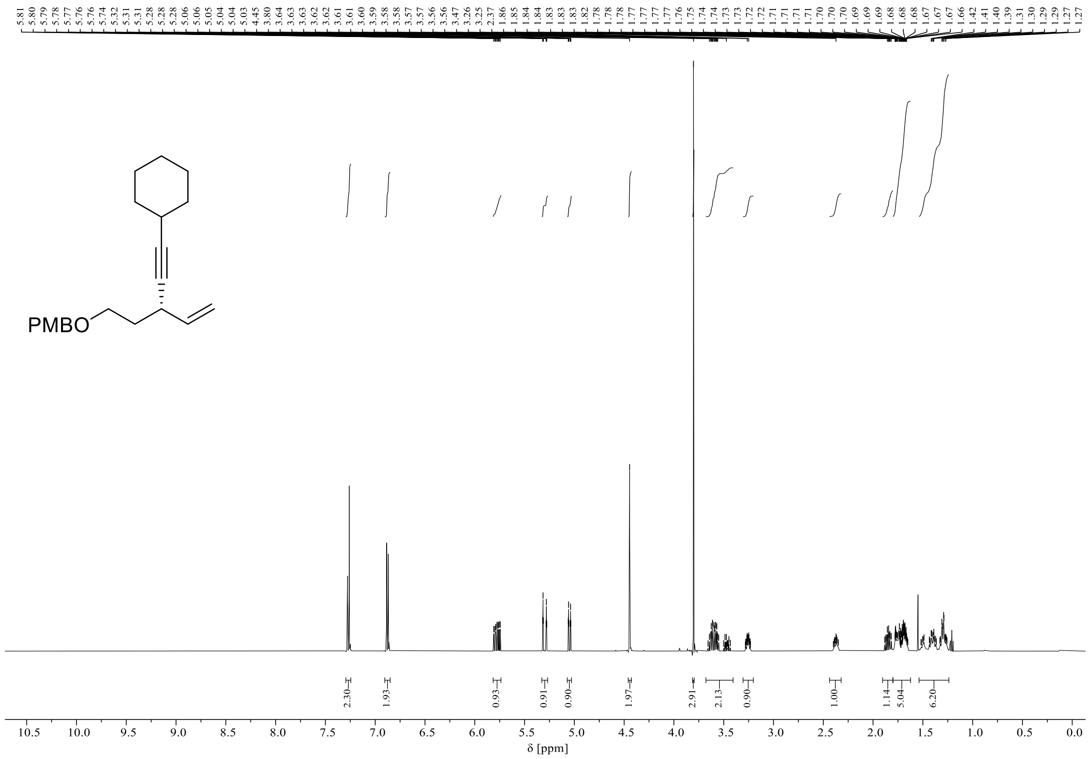

Figure 72: ^{1}H-NMR spectrum of (*S*)-1-(((3-(cyclohexylethynyl)pent-4-en-1-yl)oxy)methyl)-4-methoxybenzene ($CDCl_3$, 500 MHz).

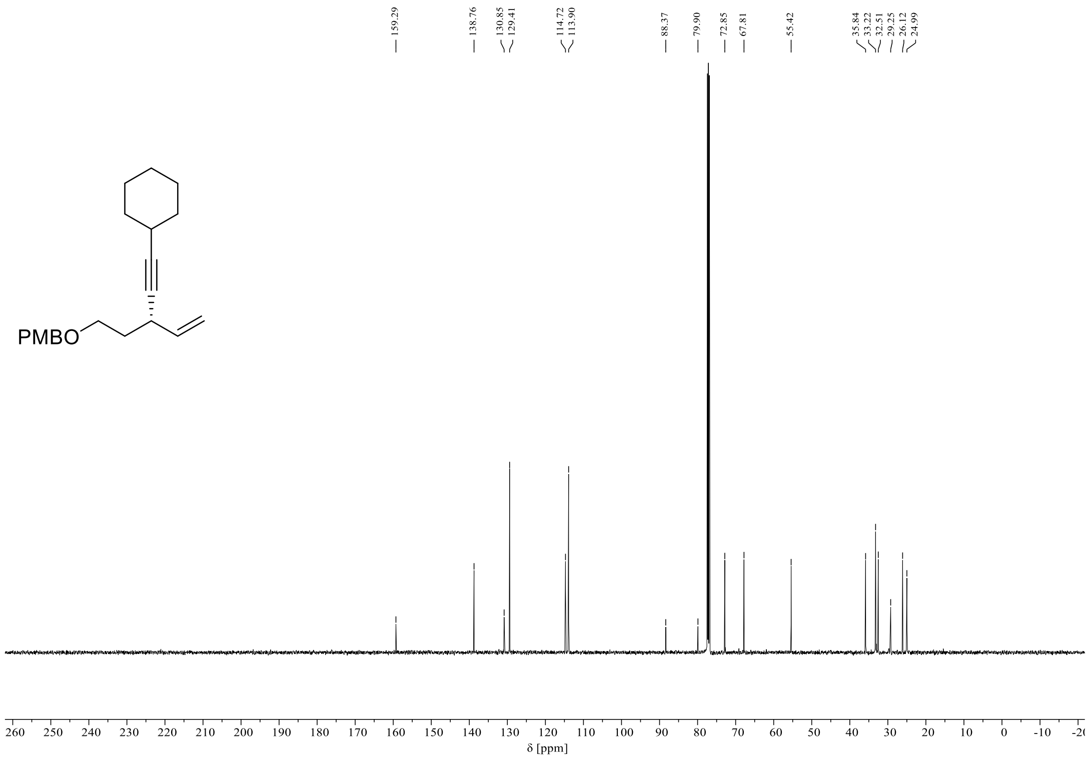

Figure 73: ^{13}C-NMR spectrum of (*S*)-1-(((3-(cyclohexylethynyl)pent-4-en-1-yl)oxy)methyl)-4-methoxybenzene ($CDCl_3$, 126 MHz).

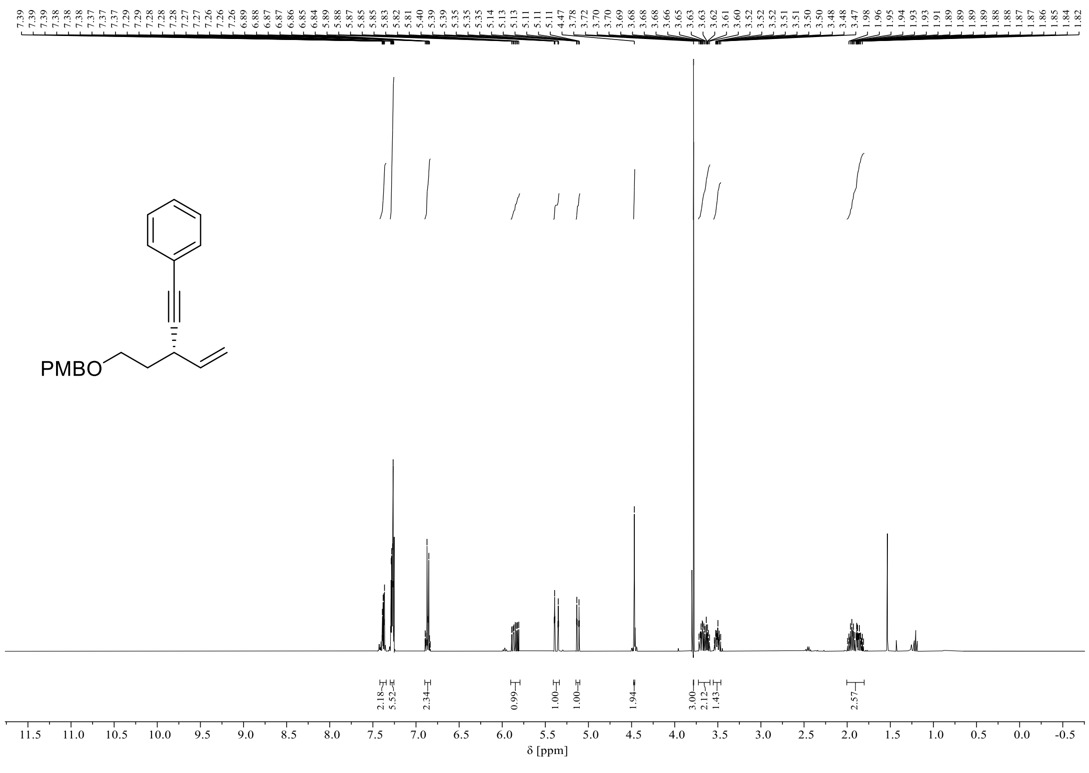

Figure 74: ^{1}H-NMR spectrum of (*S*)-1-methoxy-4-(((3-(phenylethynyl)pent-4-en-1-yl)oxy)methyl)benzene ($CDCl_3$, 400 MHz).

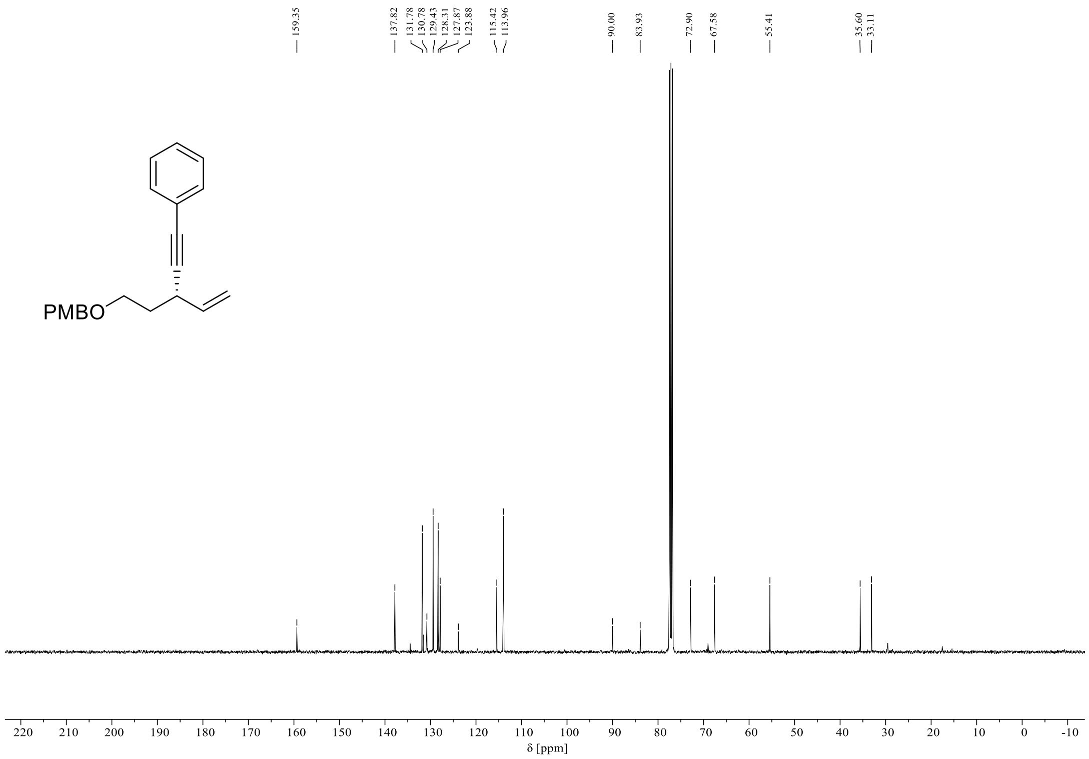

Figure 75: ^{13}C-NMR spectrum of (*S*)-1-methoxy-4-(((3-(phenylethynyl)pent-4-en-1-yl)oxy)methyl)benzene ($CDCl_3$, 101 MHz).

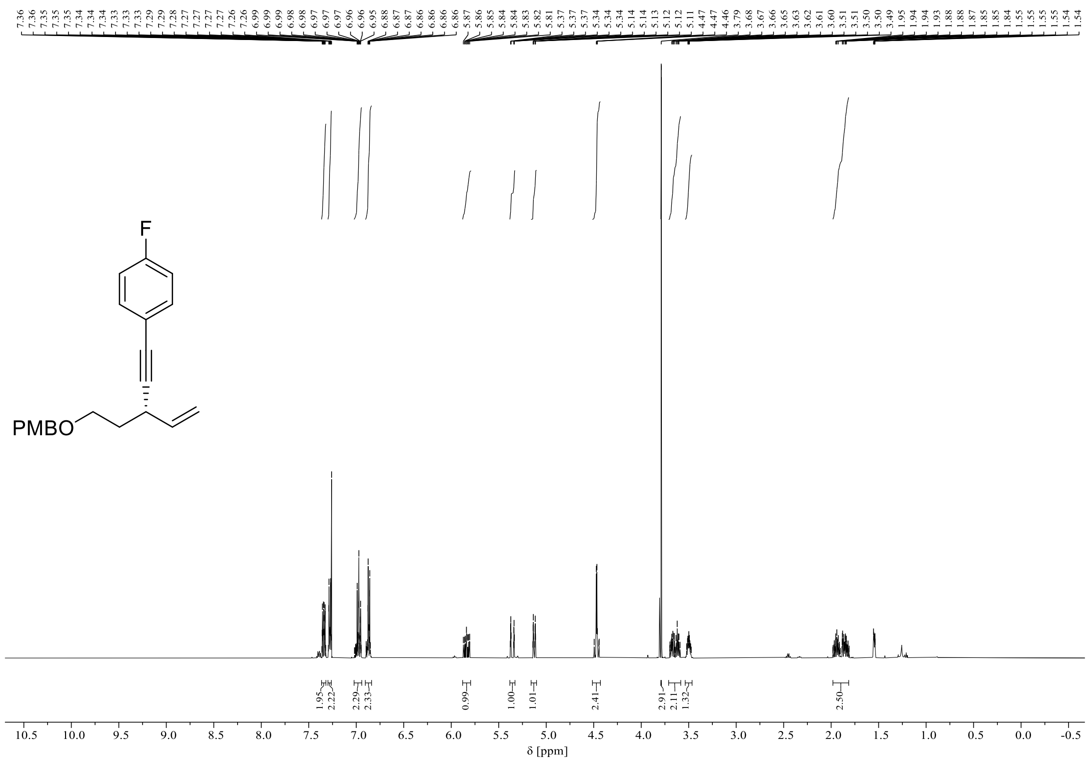

Figure 76: ^{1}H-NMR spectrum of (*S*)-1-fluoro-4-(3-(2-((4-methoxybenzyl)oxy)ethyl)pent-4-en-1-yn-1-yl)benzene ($CDCl_3$, 500 MHz).

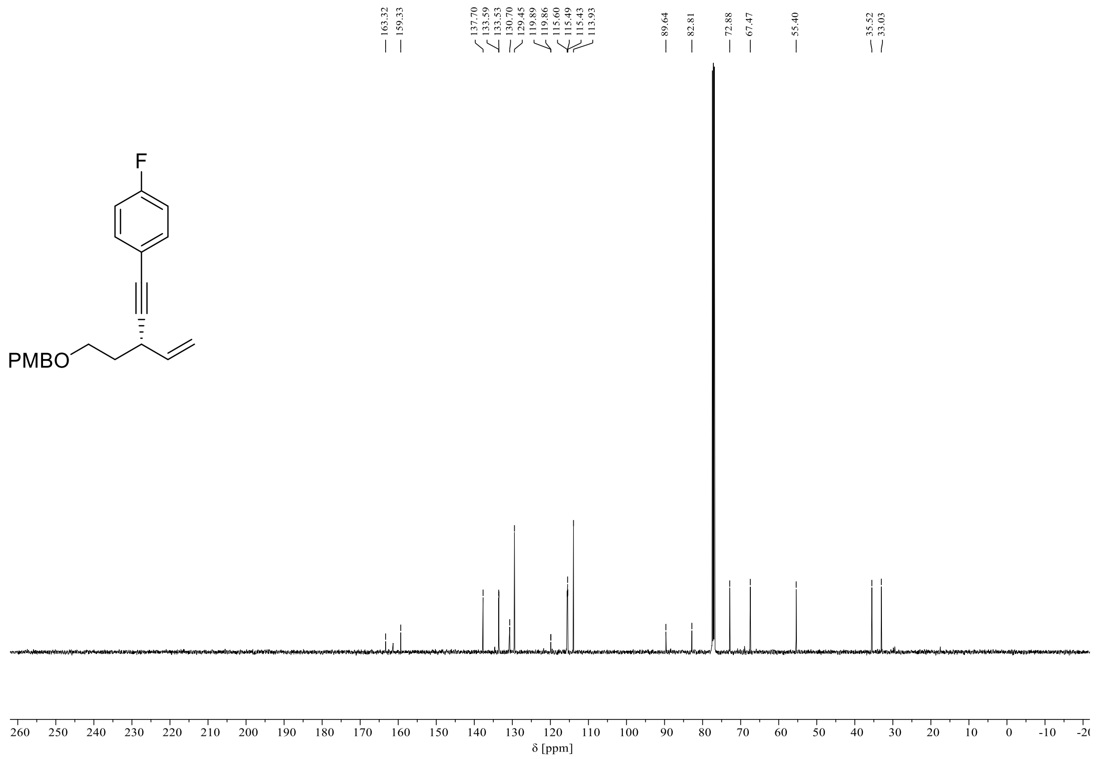

Figure 77: ^{13}C-NMR spectrum of (*S*)-1-fluoro-4-(3-(2-((4-methoxybenzyl)oxy)ethyl)pent-4-en-1-yn-1-yl)benzene ($CDCl_3$, 126 MHz).

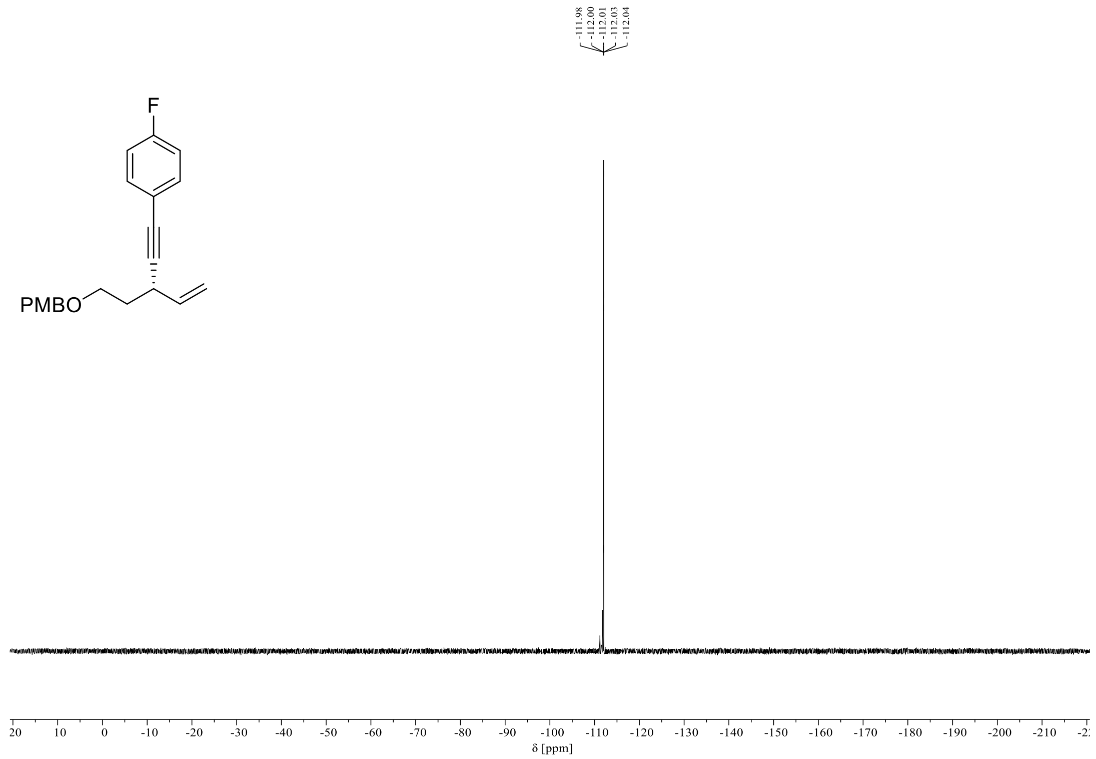

Figure 78: ^{19}F-NMR spectrum of (*S*)-1-fluoro-4-(3-(2-((4-methoxybenzyl)oxy)ethyl)pent-4-en-1-yn-1-yl)benzene ($CDCl_3$, 471 MHz).

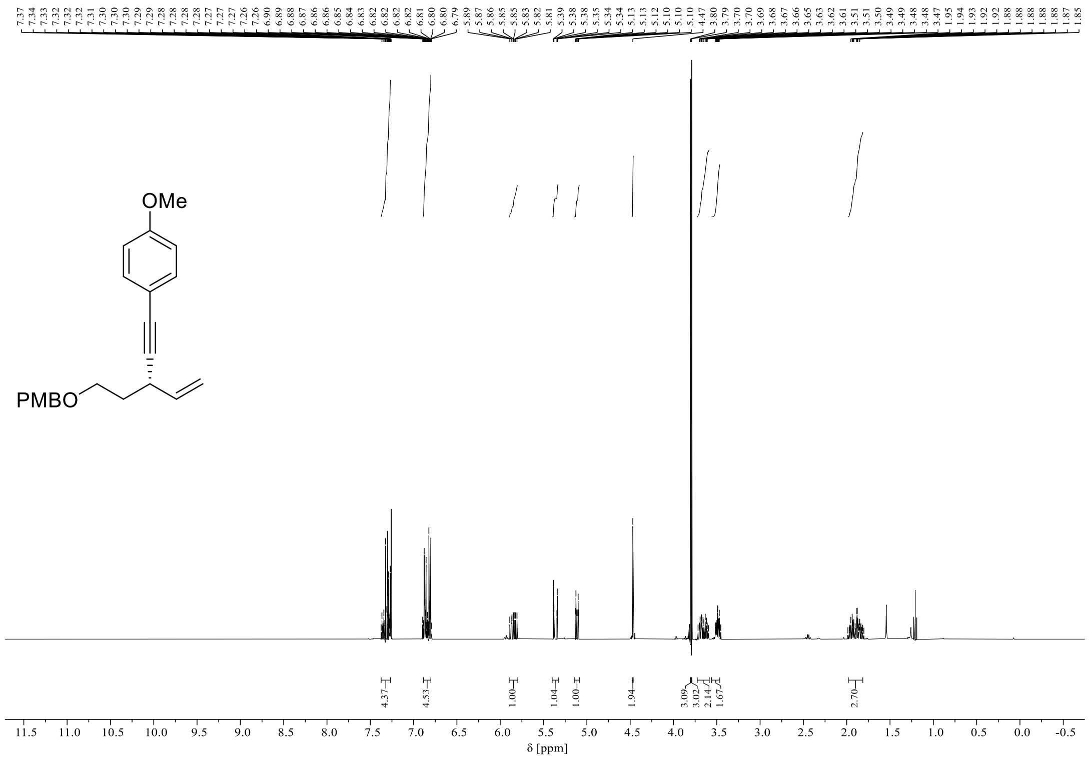

Figure 79: ^{1}H-NMR spectrum of (*S*)-1-Methoxy-4-(3-(2-((4-methoxybenzyl)oxy)ethyl)pent-4-en-1-yn-1-yl)benzene ($CDCl_3$, 400 MHz).

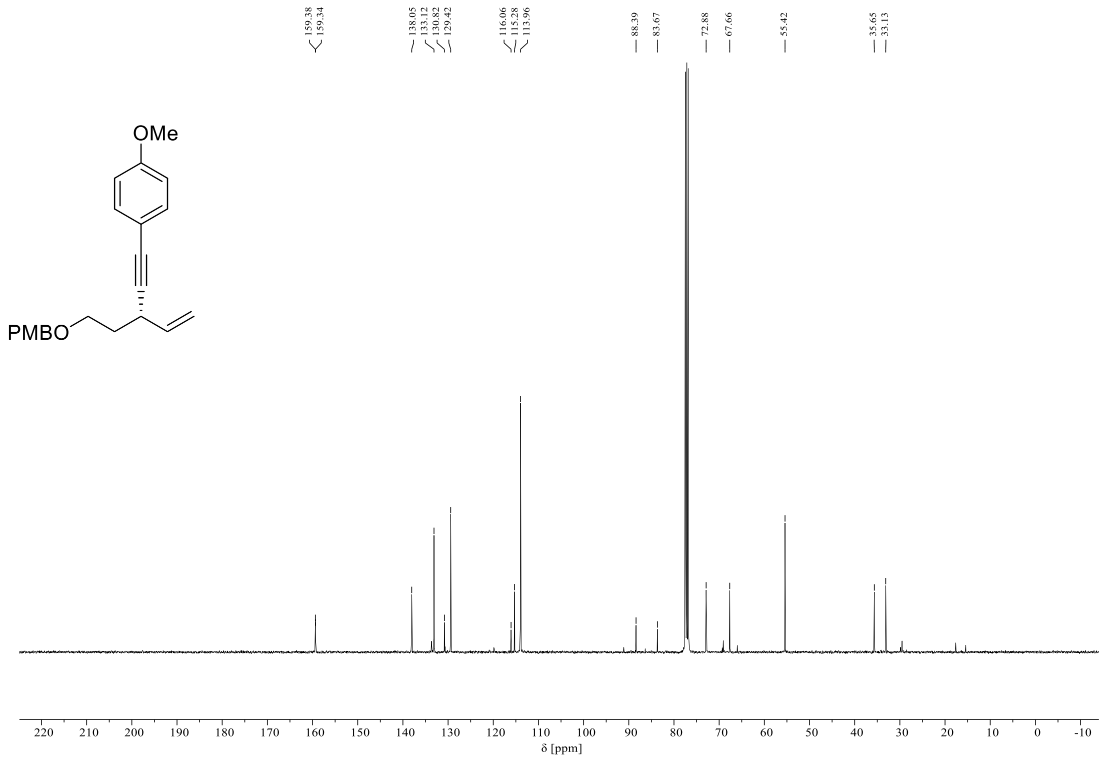

Figure 80: ^{13}C-NMR spectrum of (*S*)-1-Methoxy-4-(3-(2-((4-methoxybenzyl)oxy)ethyl)pent-4-en-1-yn-1-yl)benzene ($CDCl_3$, 101 MHz).

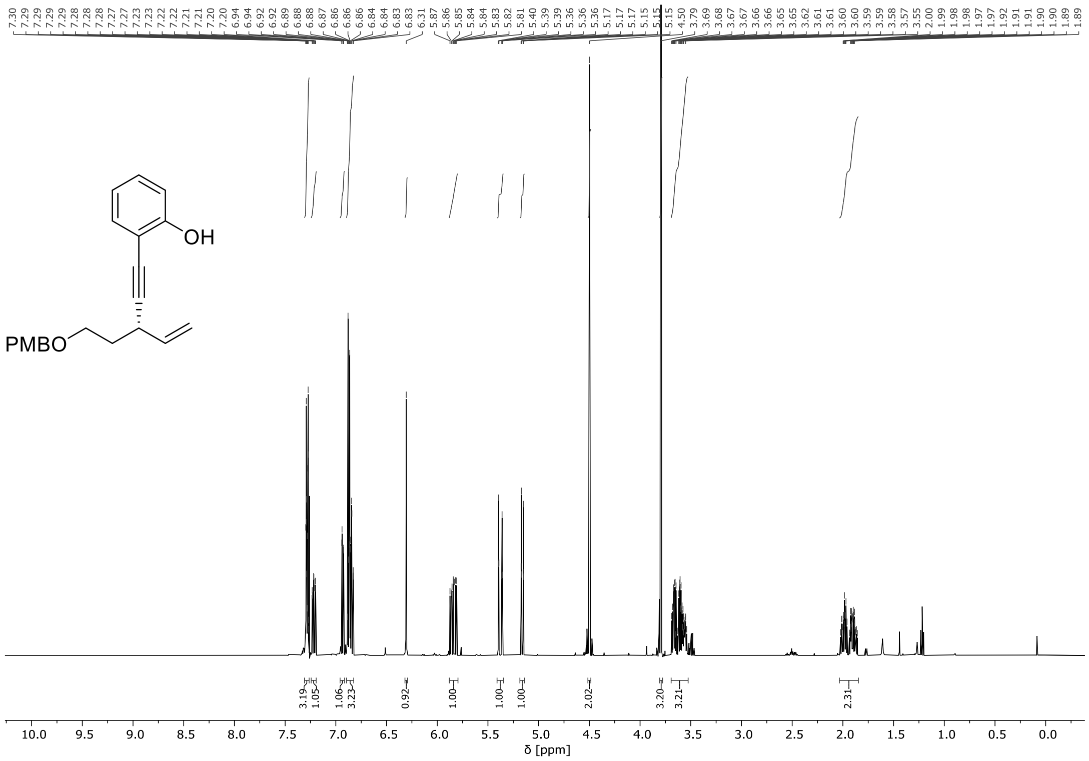

Figure 81: ^{1}H-NMR spectrum of (*S*)-2-(3-(2-((4-methoxybenzyl)oxy)ethyl)pent-4-en-1-yn-1-yl)phenol ($CDCl_3$, 500 MHz).

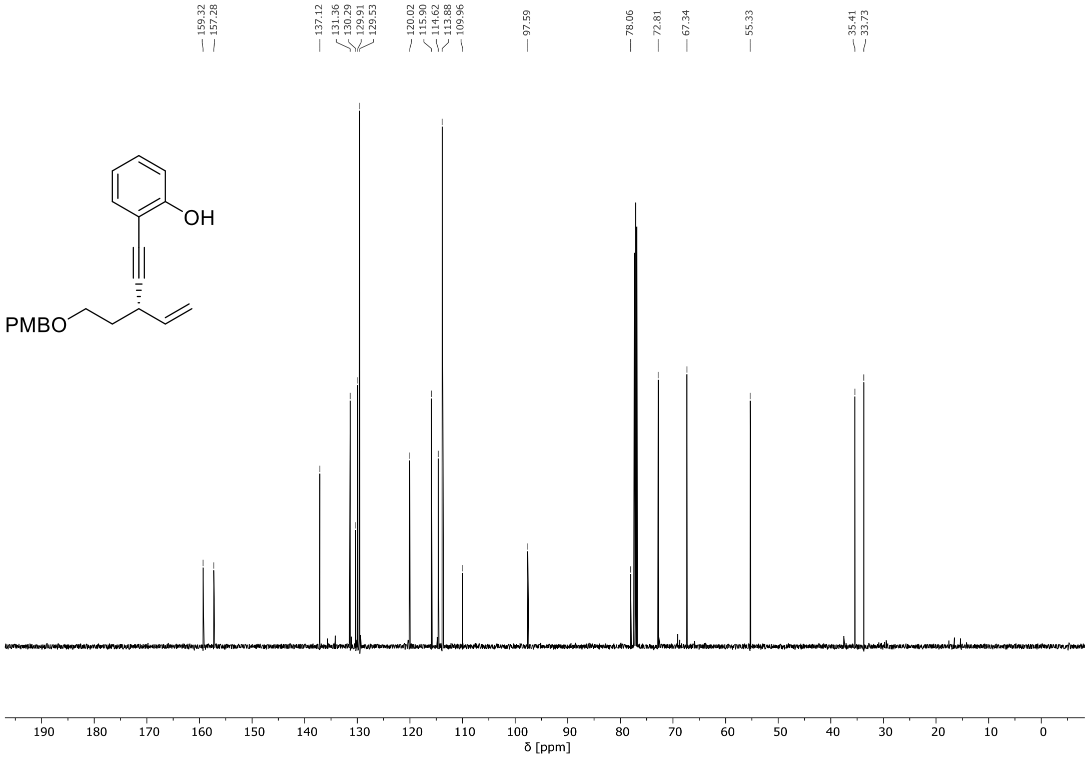

Figure 82: ^{13}C-NMR spectrum of (*S*)-2-(3-(2-((4-methoxybenzyl)oxy)ethyl)pent-4-en-1-yn-1-yl)phenol ($CDCl_3$, 126 MHz).

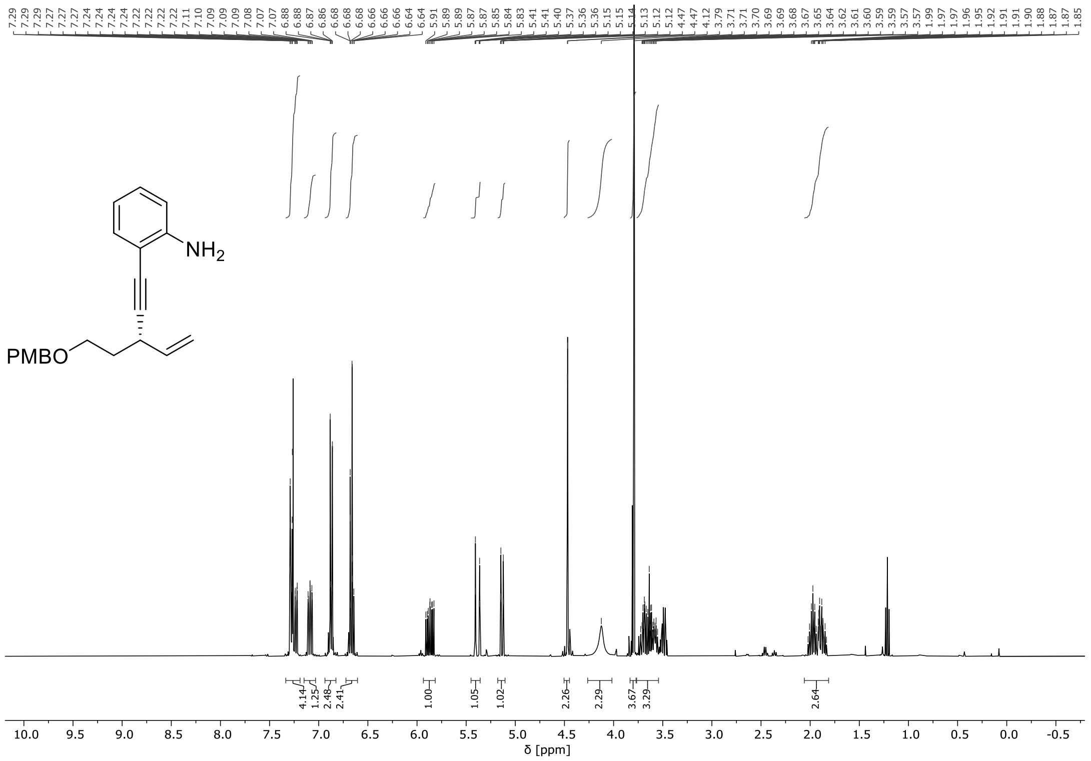

Figure 83: ^{1}H-NMR spectrum of (*S*)-2-(3-(2-((4-methoxybenzyl)oxy)ethyl)pent-4-en-1-yn-1-yl)aniline ($CDCl_3$, 400 MHz, contains Et_2O).

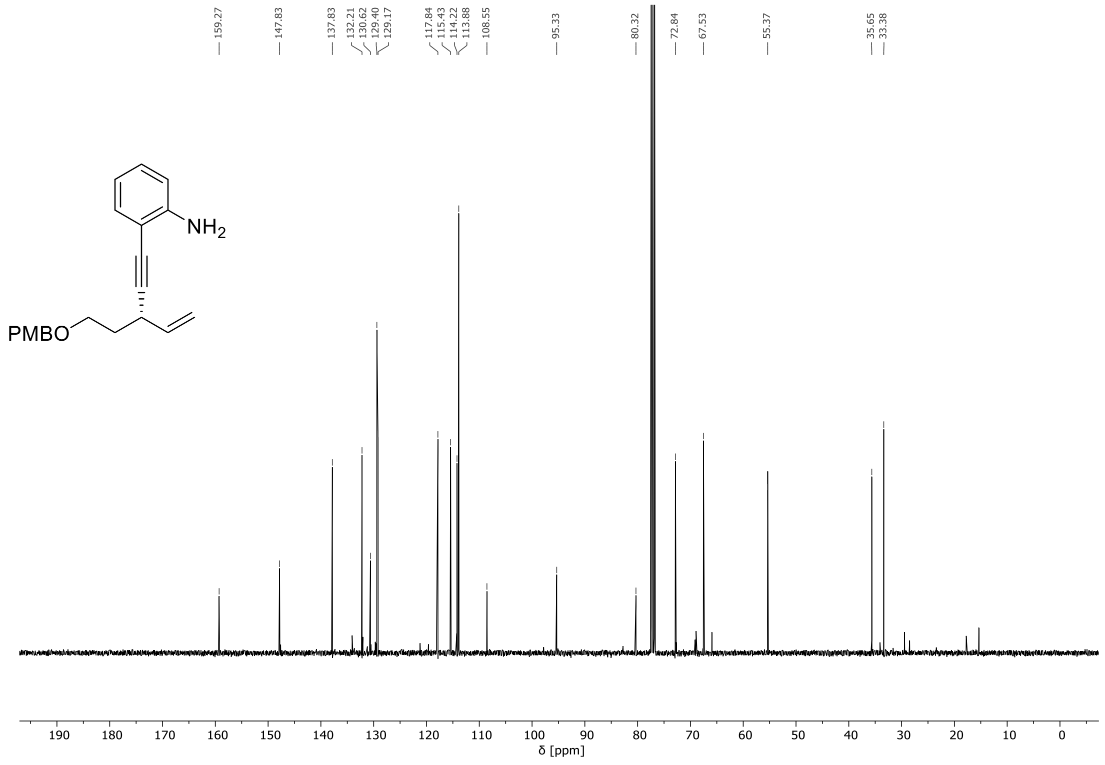

Figure 84: ^{13}C-NMR spectrum of (*S*)-2-(3-(2-((4-methoxybenzyl)oxy)ethyl)pent-4-en-1-yn-1-yl)aniline ($CDCl_3$, 101 MHz).

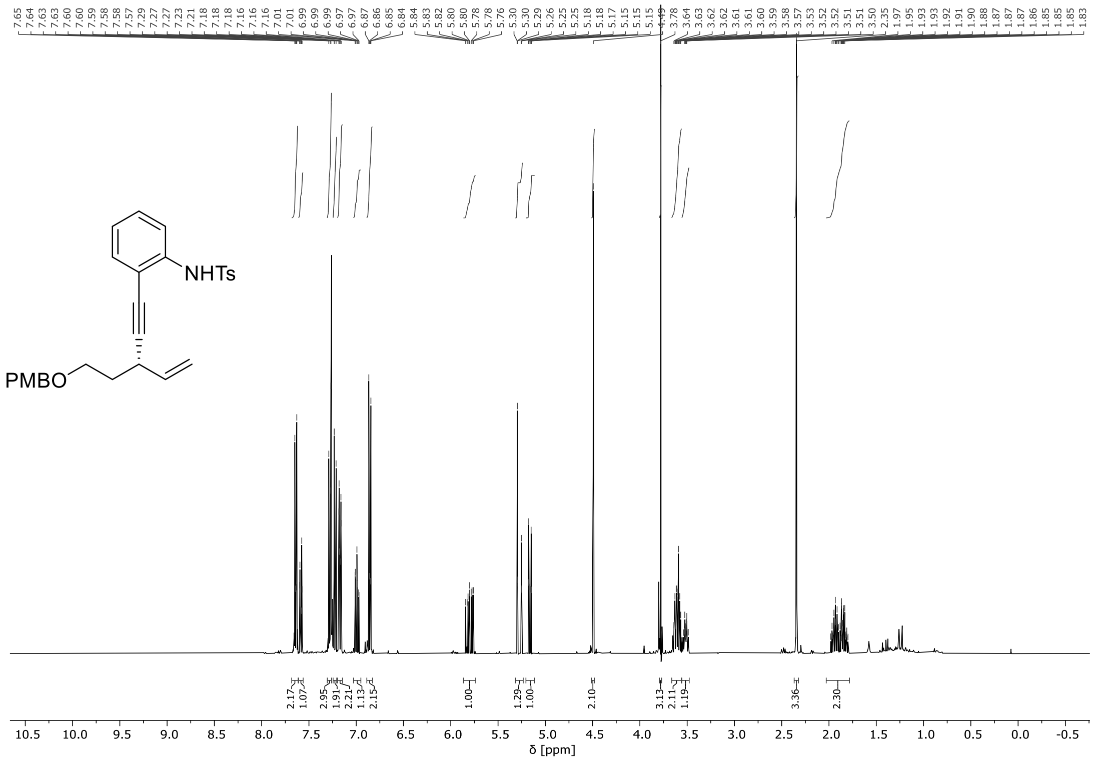

Figure 85: ^{1}H-NMR spectrum of (*S*)-N-(2-(3-(2-((4-methoxybenzyl)oxy)ethyl)pent-4-en-1-yn-1-yl)phenyl)-4-methylbenzene-sulfonamide ($CDCl_3$, 400 MHz, contains CH_2Cl_2).

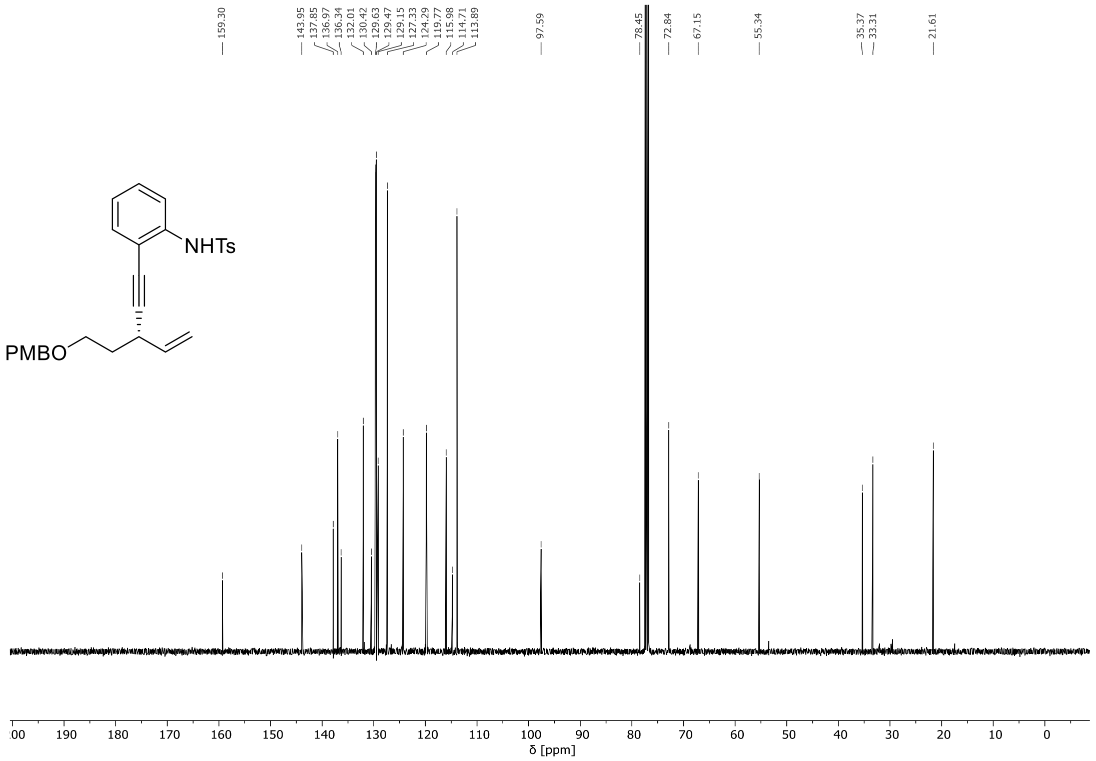

Figure 86: ^{13}C-NMR spectrum of (*S*)-N-(2-(3-(2-((4-methoxybenzyl)oxy)ethyl)pent-4-en-1-yn-1-yl)phenyl)-4-methylbenzene-sulfonamide ($CDCl_3$, 101 MHz).

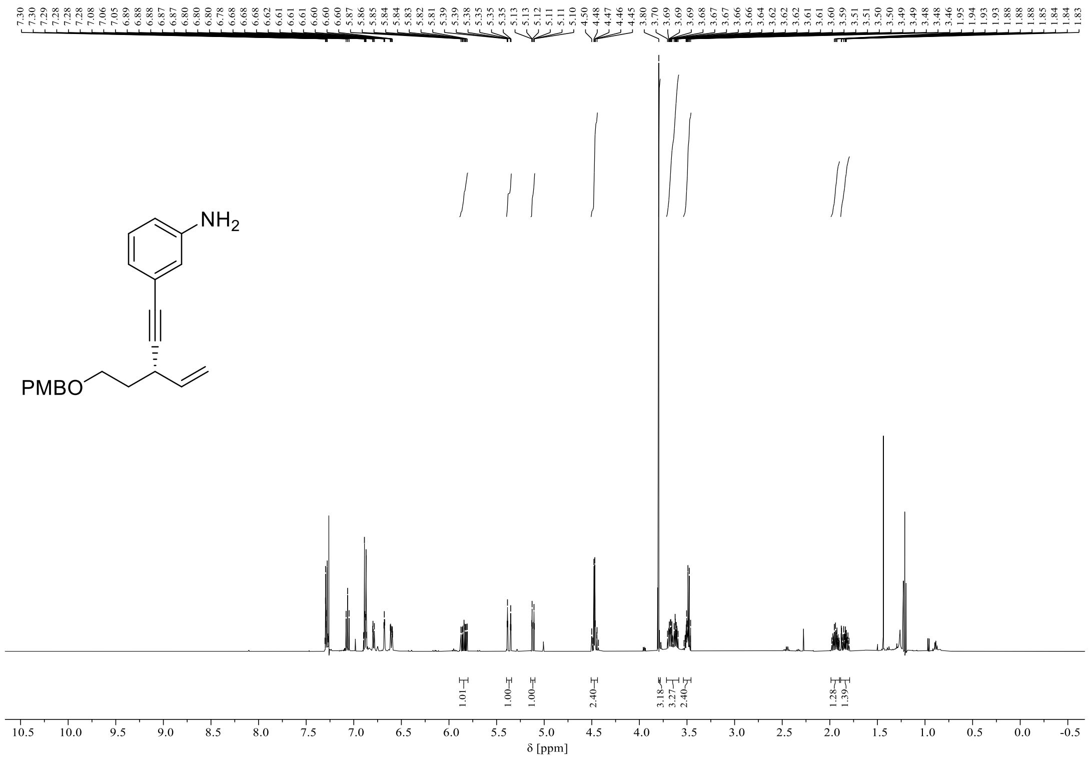

Figure 87: ^{1}H-NMR spectrum of (*S*)-3-(3-(2-((4-methoxybenzyl)oxy)ethyl)pent-4-en-1-yn-1-yl)aniline ($CDCl_3$, 500 MHz).

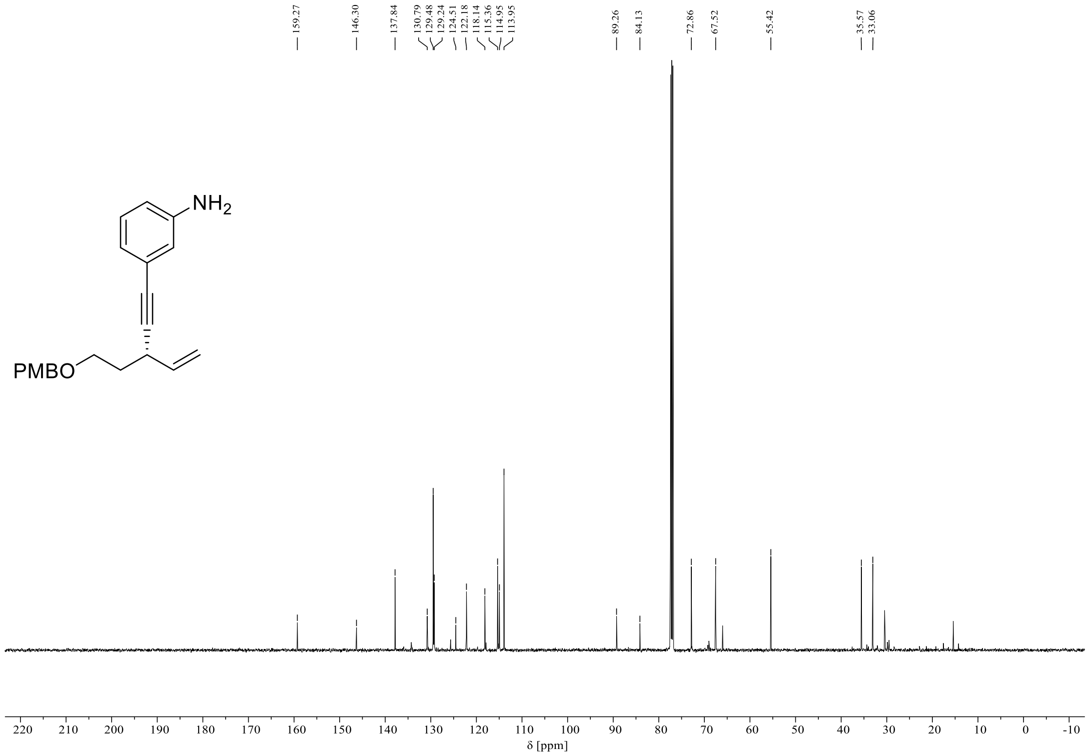

Figure 88: ^{13}C-NMR spectrum of (*S*)-3-(3-(2-((4-methoxybenzyl)oxy)ethyl)pent-4-en-1-yn-1-yl)aniline ($CDCl_3$, 126 MHz).

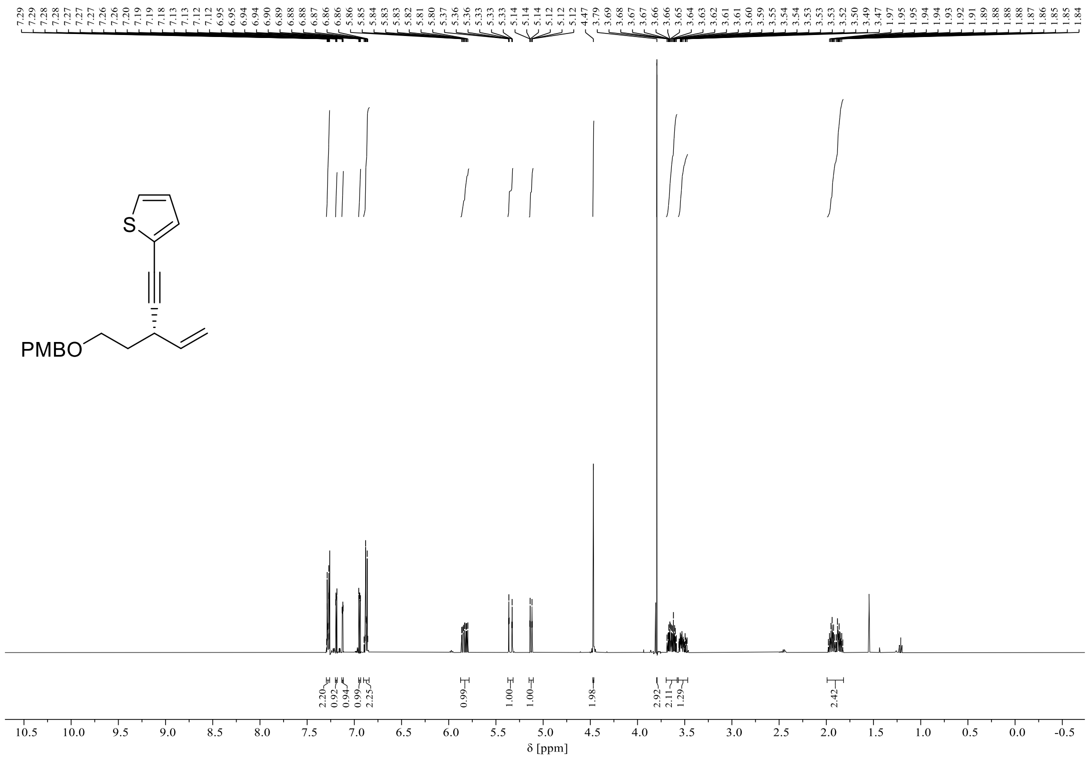

Figure 89: ^{1}H-NMR spectrum of (*S*)-2-(3-(2-((4-methoxybenzyl)oxy)ethyl)pent-4-en-1-yn-1-yl)thiophene ($CDCl_3$, 500 MHz).

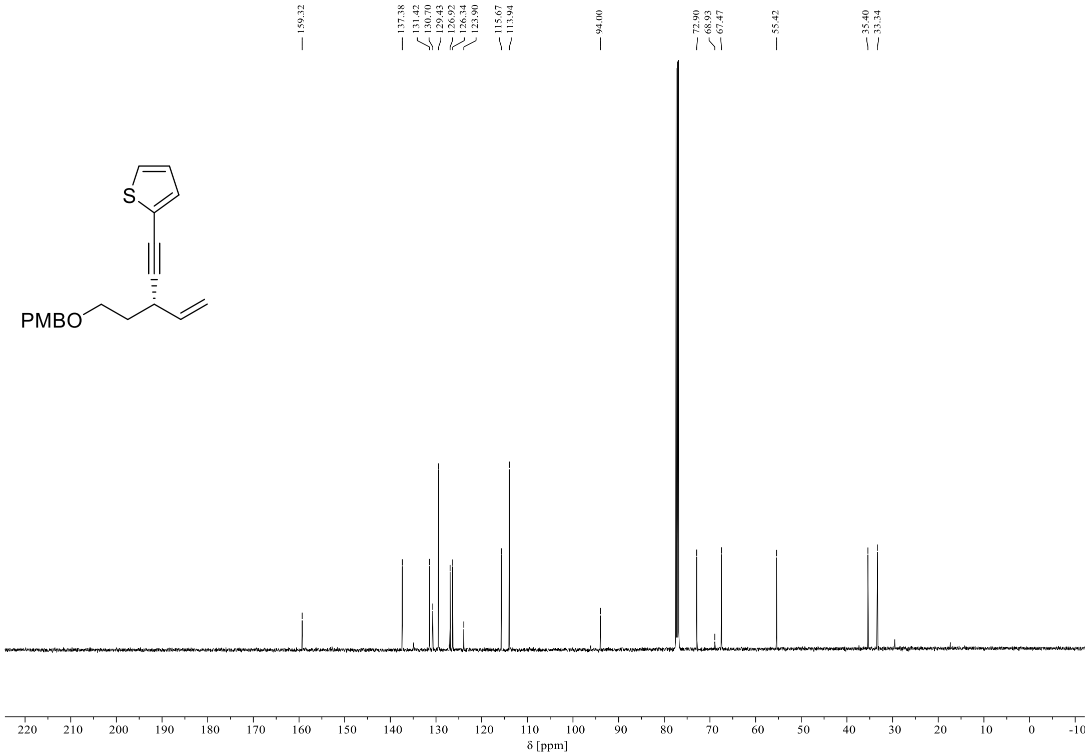

Figure 90: ^{13}C-NMR spectrum of (*S*)-2-(3-(2-((4-Methoxybenzyl)oxy)ethyl)pent-4-en-1-yn-1-yl)thiophene ($CDCl_3$, 126 MHz).

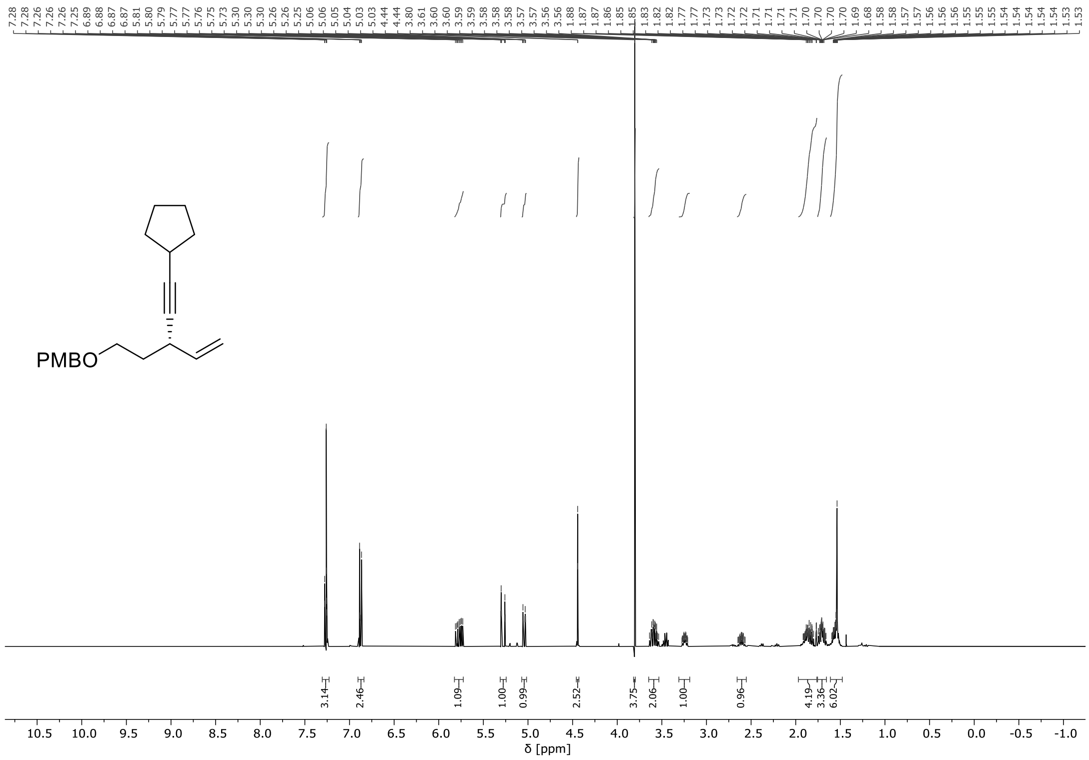

Figure 91: ^{1}H-NMR spectrum of (*S*)-1-(((3-(cyclopentylethynyl)pent-4-en-1-yl)oxy)methyl)-4-methoxybenzene ($CDCl_3$, 400 MHz).

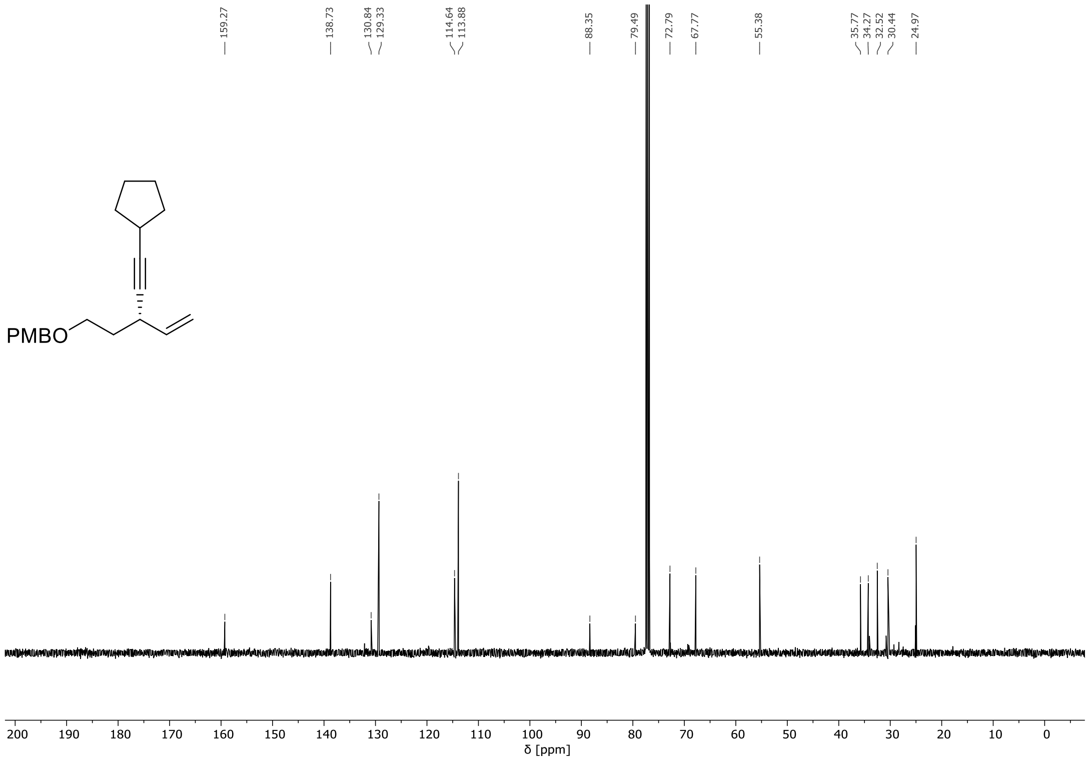

Figure 92: ^{13}C-NMR spectrum of (*S*)-1-(((3-(cyclopentylethynyl)pent-4-en-1-yl)oxy)methyl)-4-methoxybenzene ($CDCl_3$, 101 MHz).

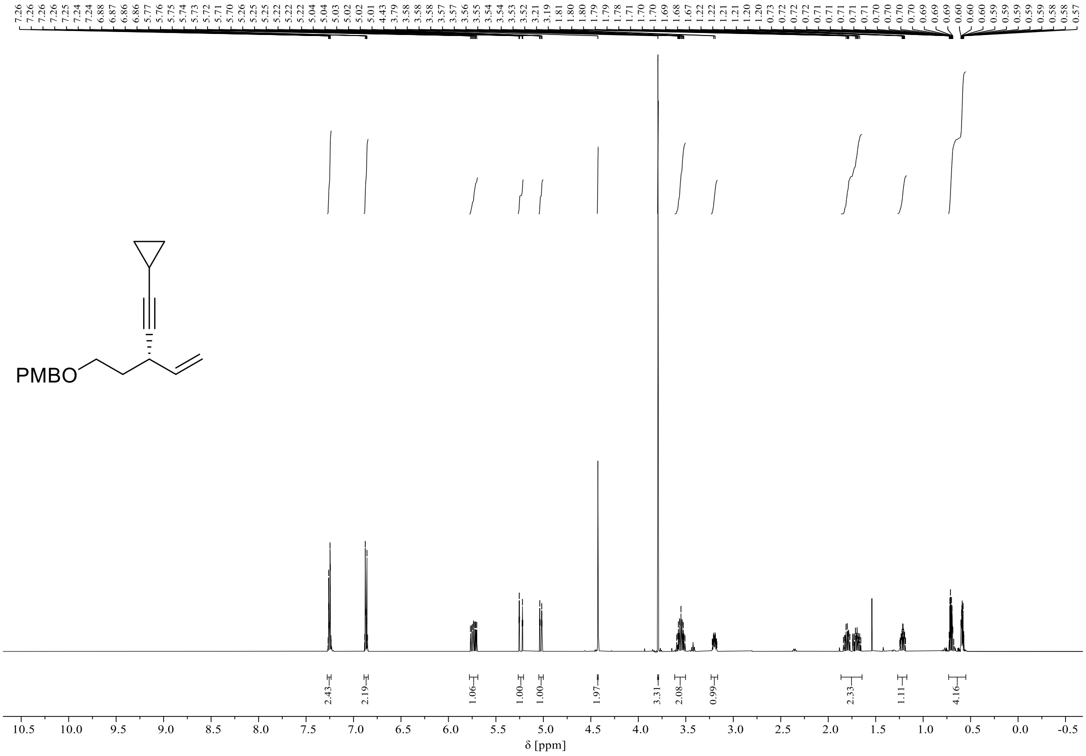

Figure 93: ^{1}H-NMR spectrum of (*S*)-1-(((3-(cyclopropylethynyl)pent-4-en-1-yl)oxy)methyl)-4-methoxybenzene ($CDCl_3$, 500 MHz).

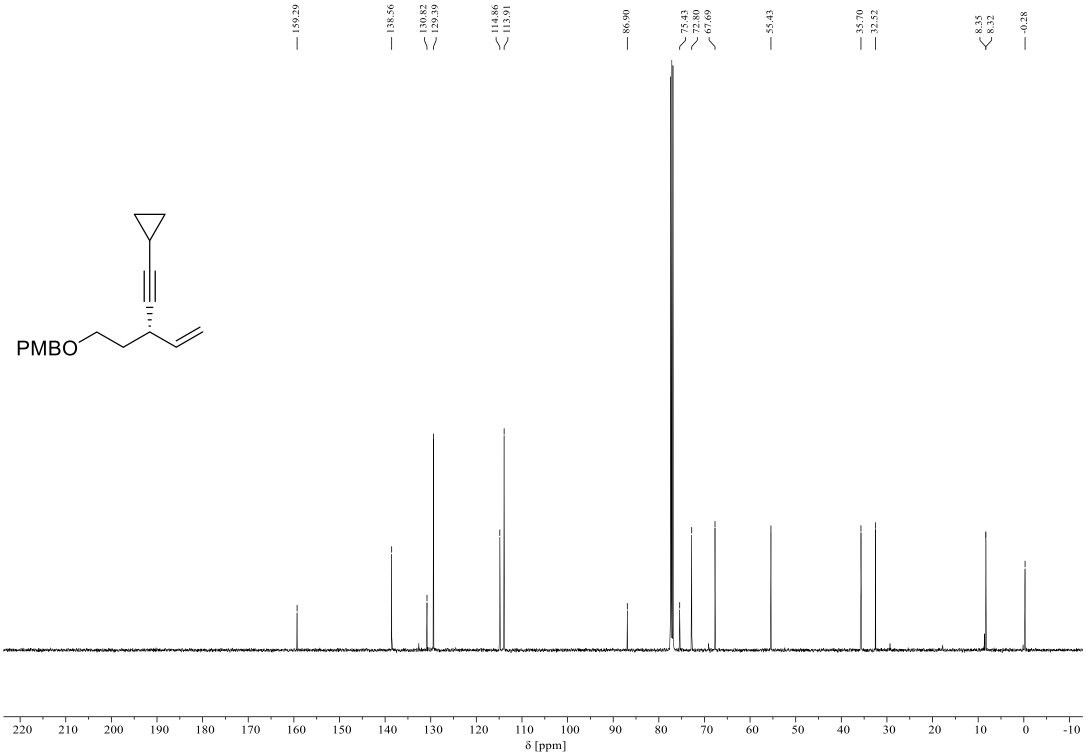

Figure 94: ^{13}C-NMR spectrum of (*S*)-1-(((3-(cyclopropylethynyl)pent-4-en-1-yl)oxy)methyl)-4-methoxybenzene ($CDCl_3$, 126 MHz).

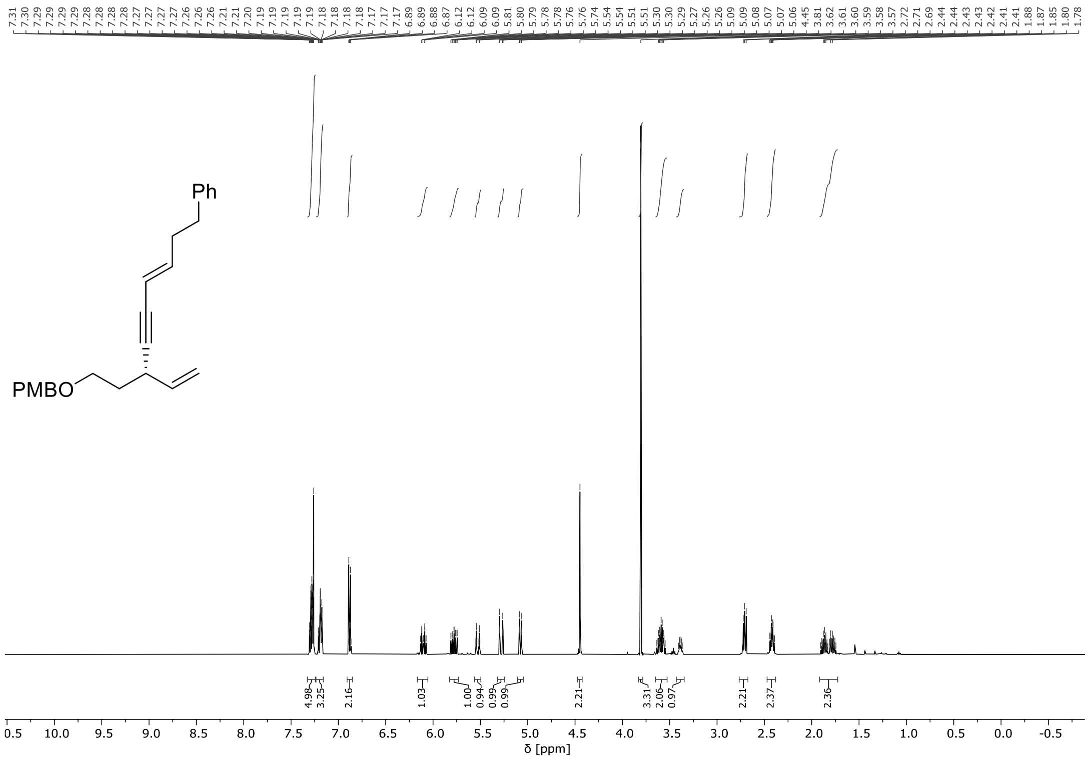

Figure 95: ^{1}H-NMR spectrum of (*S,E*)-1-methoxy-4-(((9-phenyl-3-vinylnon-6-en-4-yn-1-yl)oxy)methyl)benzene ($CDCl_3$, 500 MHz).

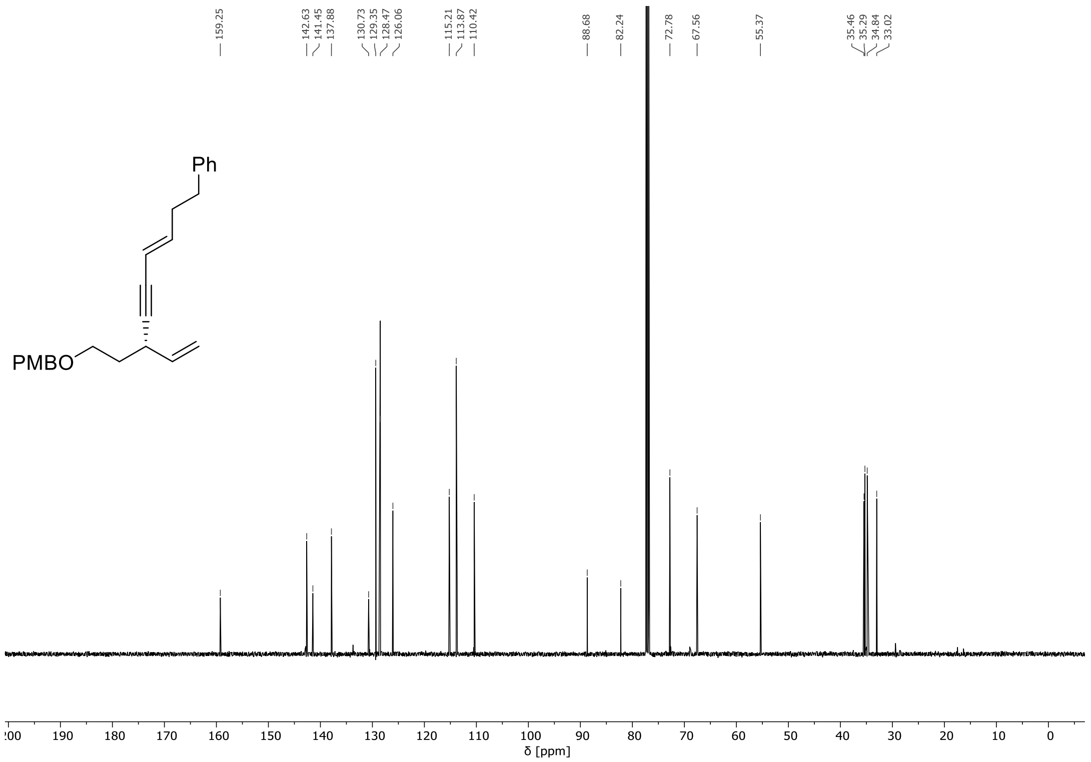

Figure 96: ^{13}C-NMR spectrum of (*S,E*)-1-methoxy-4-(((9-phenyl-3-vinylnon-6-en-4-yn-1-yl)oxy)methyl)benzene ($CDCl_3$, 126 MHz).

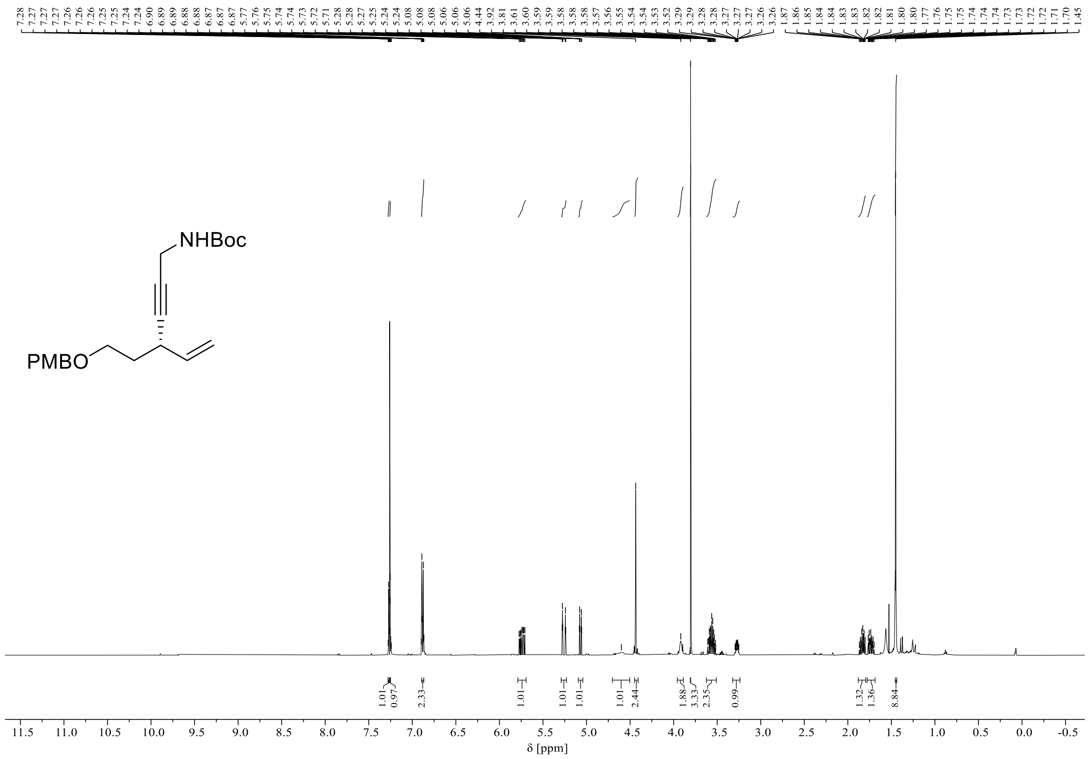

Figure 97: ^{1}H-NMR spectrum of *tert*-butyl (*S*)-(4-(2-((4-methoxybenzyl)oxy)ethyl)hex-5-en-2-yn-1-yl)carbamate ($CDCl_3$, 500 MHz).

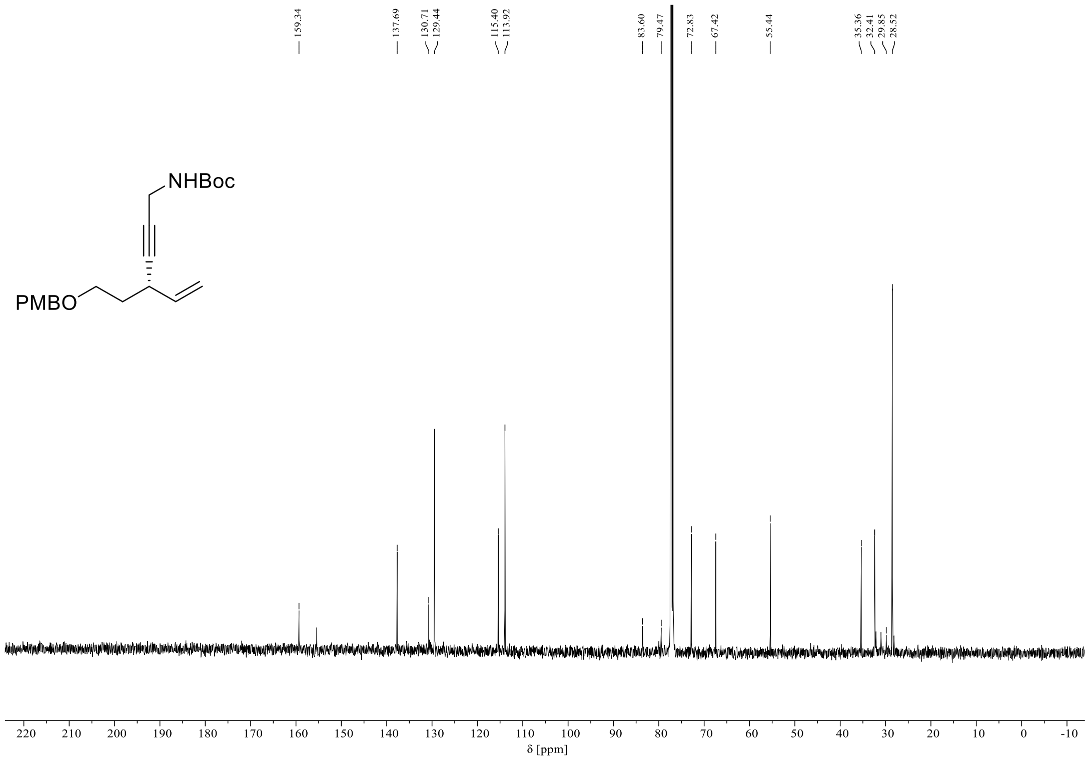

Figure 98: ^{13}C-NMR spectrum of *tert*-butyl (*S*)-(4-(2-((4-methoxybenzyl)oxy)ethyl)hex-5-en-2-yn-1-yl)carbamate ($CDCl_3$, 126 MHz).

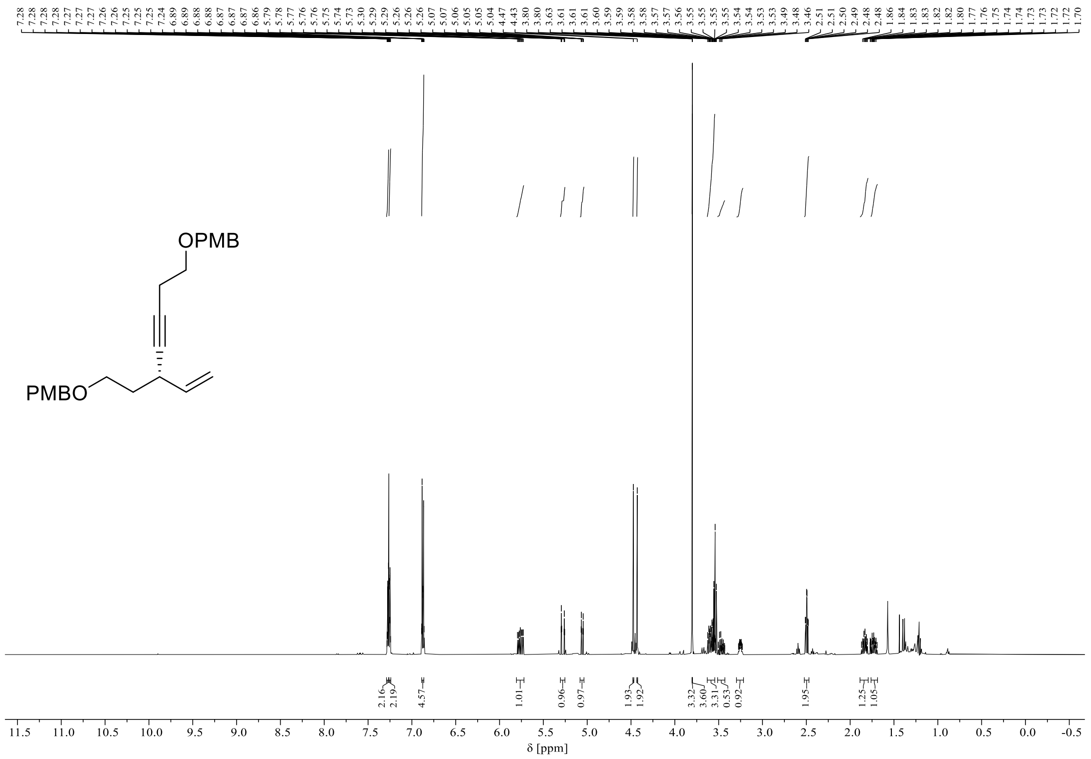

Figure 99: ^{1}H-NMR spectrum of *tert*-butyl (*S*)-4,4'-(((5-vinylhept-3-yne-1,7-diyl)bis(oxy))bis(methylene))bis(methoxybenzene) ($CDCl_3$, 500 MHz).

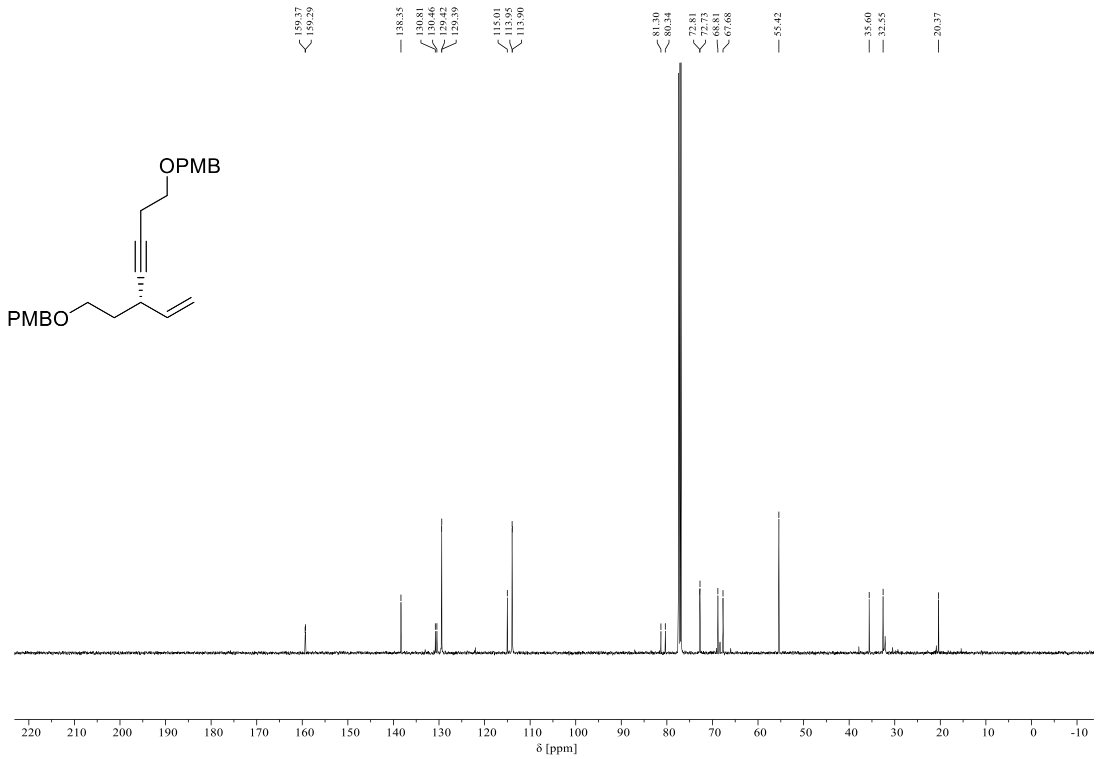

Figure 100: ^{13}C-NMR spectrum of *tert*-butyl (*S*)-4,4'-(((5-vinylhept-3-yne-1,7-diyl)bis(oxy))bis(methylene))bis(methoxybenzene) ($CDCl_3$, 126 MHz).

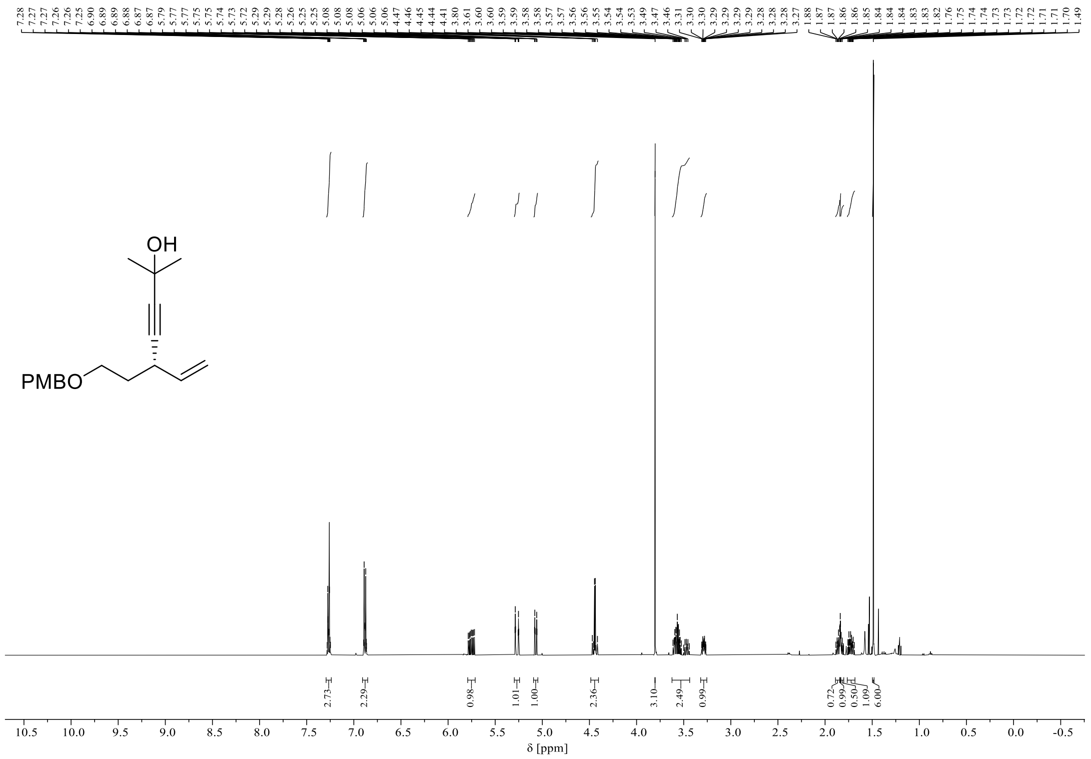

Figure 101: ^{1}H-NMR spectrum of *tert*-butyl (*S*)-5-(2-((4-methoxybenzyl)oxy)ethyl)-2-methylhept-6-en-3-yn-2-ol ($CDCl_3$, 500 MHz).

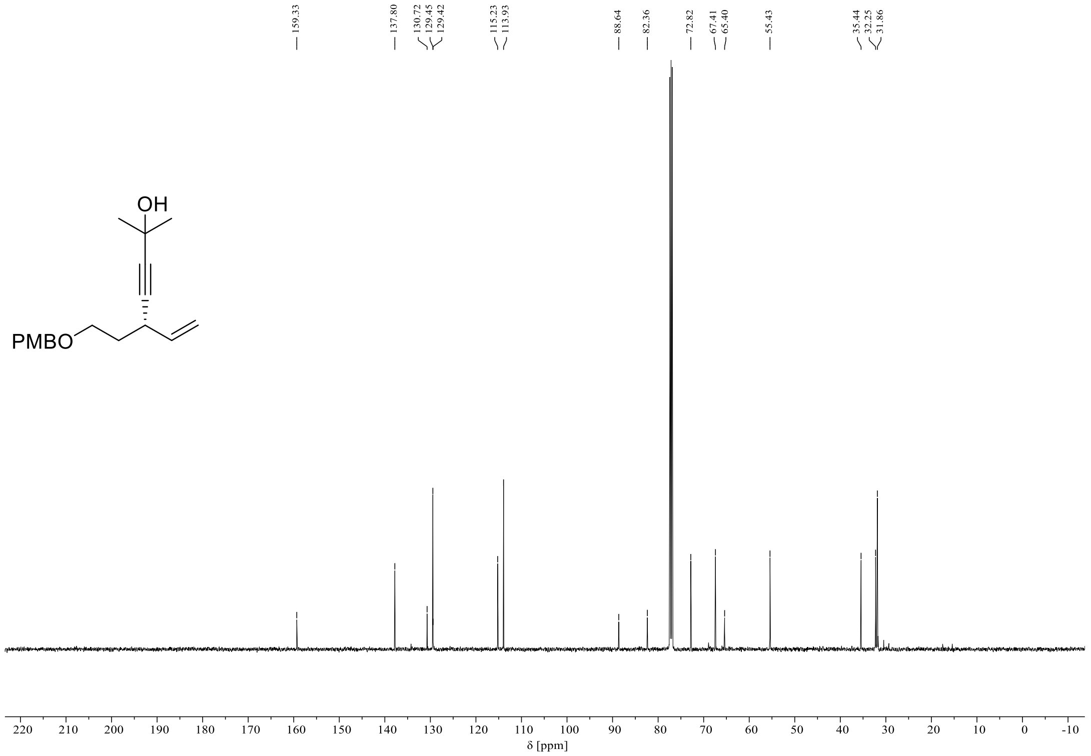

Figure 102: ^{13}C-NMR spectrum of *tert*-butyl (*S*)-5-(2-((4-methoxybenzyl)oxy)ethyl)-2-methylhept-6-en-3-yn-2-ol ($CDCl_3$, 126 MHz).

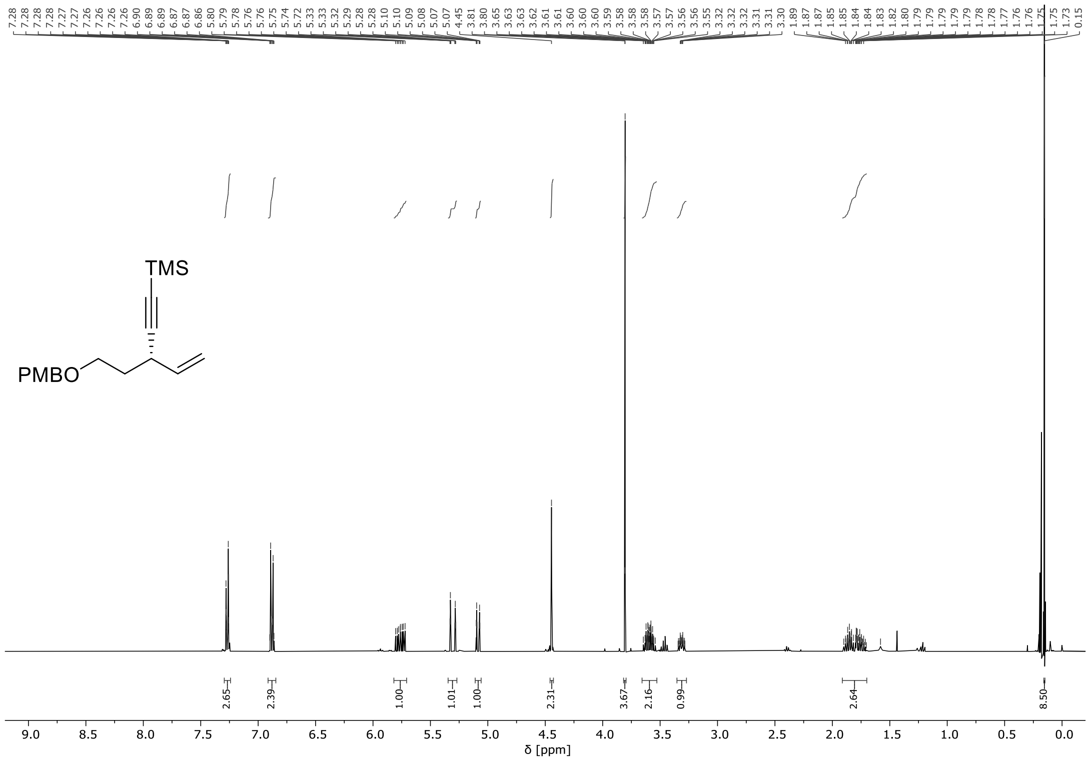

Figure 103: ^{1}H-NMR spectrum of *tert*-butyl (*S*)-(3-(2-((4-methoxybenzyl)oxy)ethyl)pent-4-en-1-yn-1-yl)trimethylsilane ($CDCl_3$, 400 MHz).

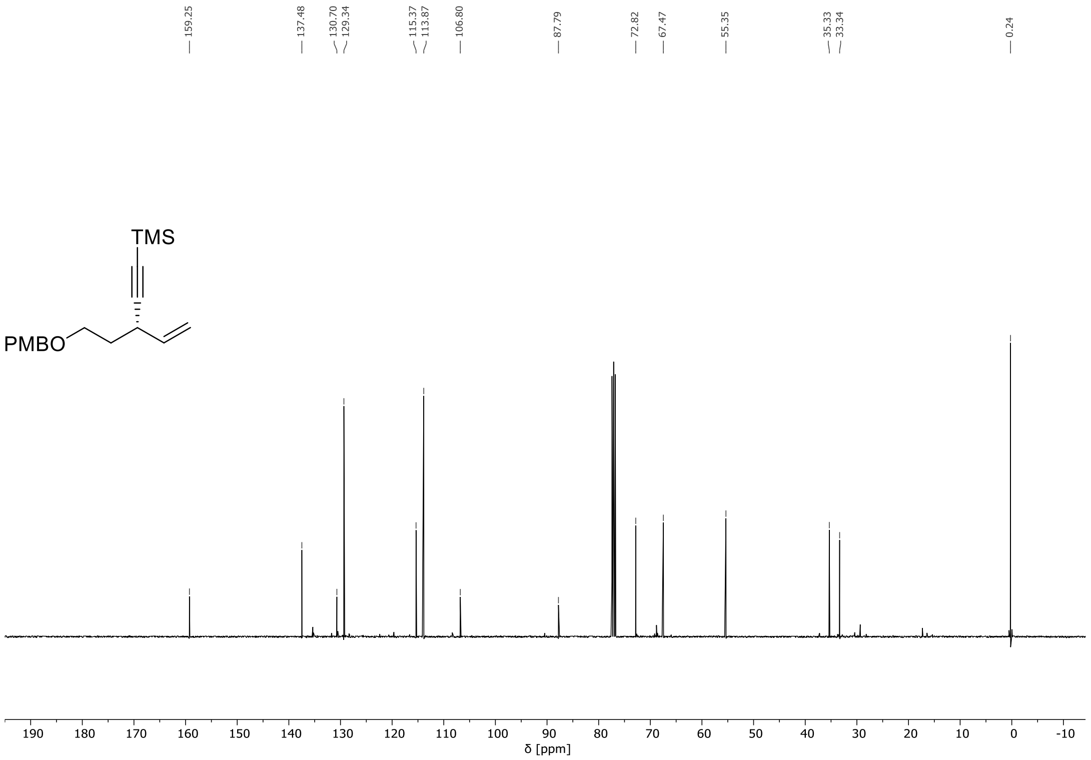

Figure 104: ^{13}C-NMR spectrum of *tert*-butyl (*S*)-(3-(2-((4-methoxybenzyl)oxy)ethyl)pent-4-en-1-yn-1-yl)trimethylsilane ($CDCl_3$, 101 MHz).

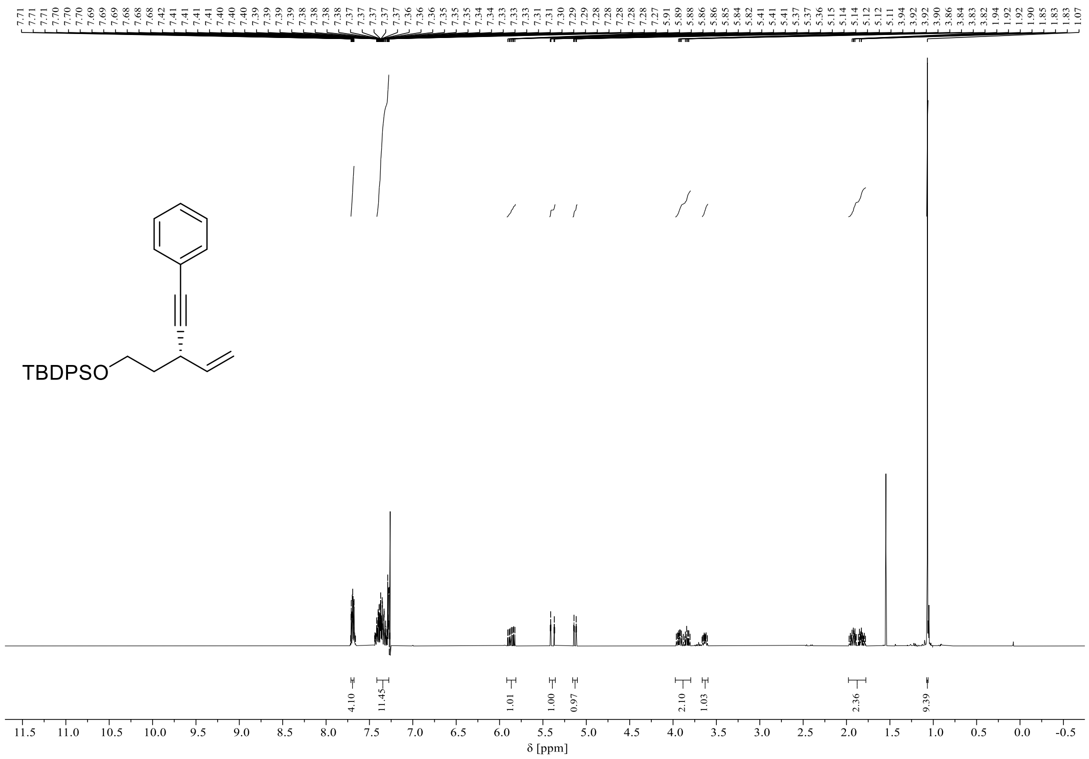

Figure 105: ^{1}H-NMR spectrum of (*S*)-*tert*-butyldiphenyl((3-(phenylethynyl)pent-4-en-1-yl)oxy)silane ($CDCl_3$, 400 MHz).

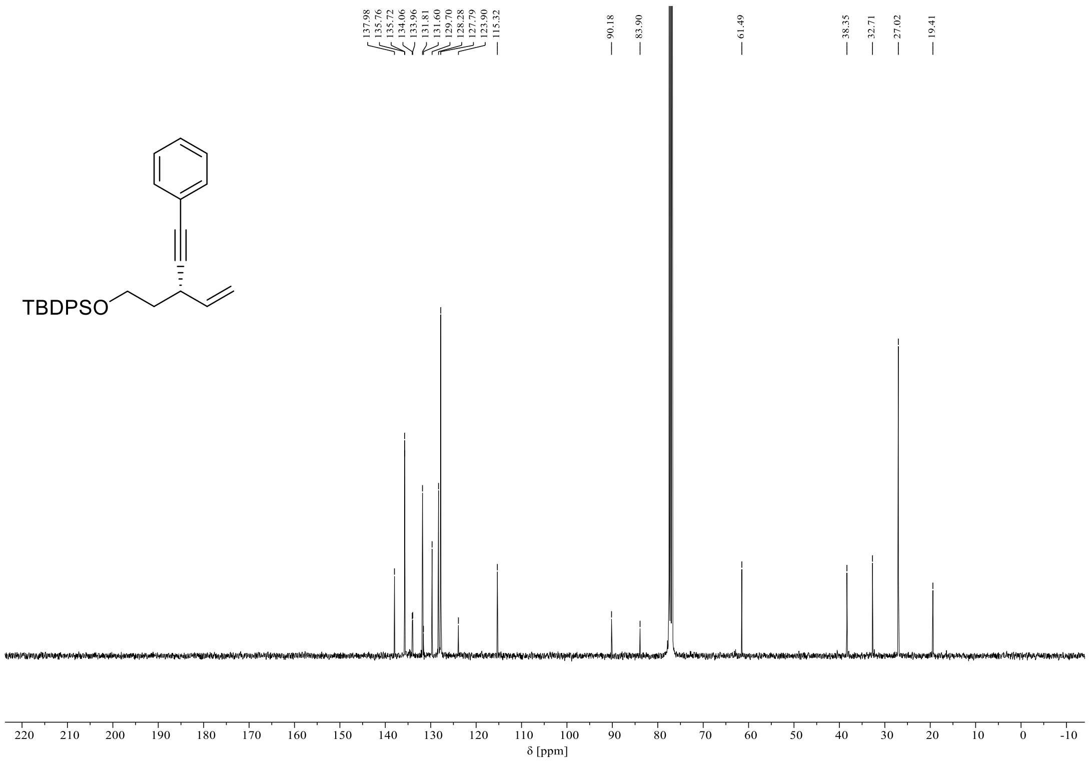

Figure 106: ^{13}C-NMR spectrum of (*S*)-*tert*-butyldiphenyl((3-(phenylethynyl)pent-4-en-1-yl)oxy)silane ($CDCl_3$, 101 MHz).

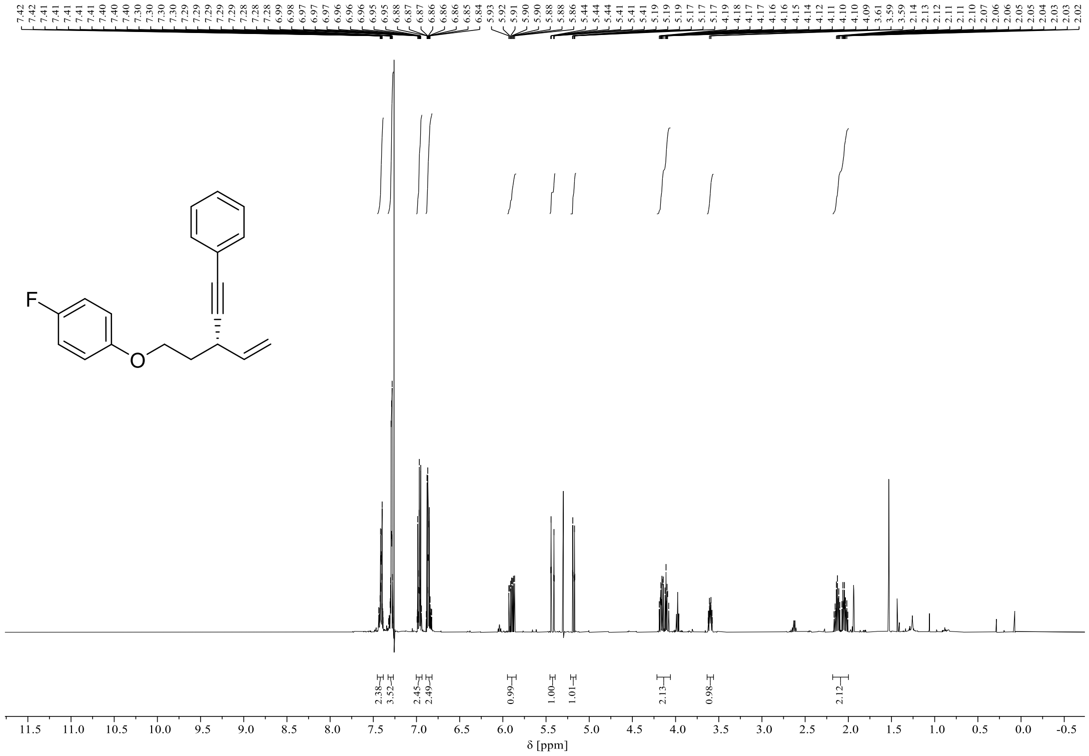

Figure 107: ^{1}H-NMR spectrum of (*S*)-1-fluoro-4-((3-(phenylethynyl)pent-4-en-1-yl)oxy)benzene ($CDCl_3$, 500 MHz).

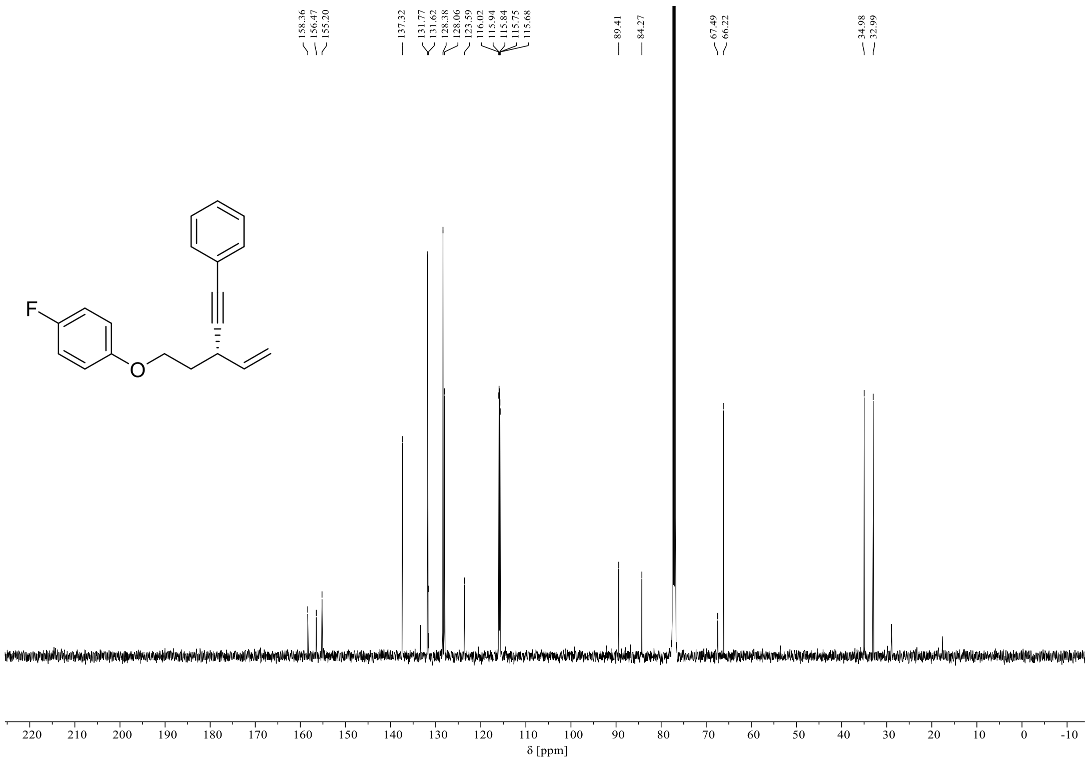

Figure 108: ^{1}H-NMR spectrum of (*S*)-1-fluoro-4-((3-(phenylethynyl)pent-4-en-1-yl)oxy)benzene ($CDCl_3$, 126 MHz).

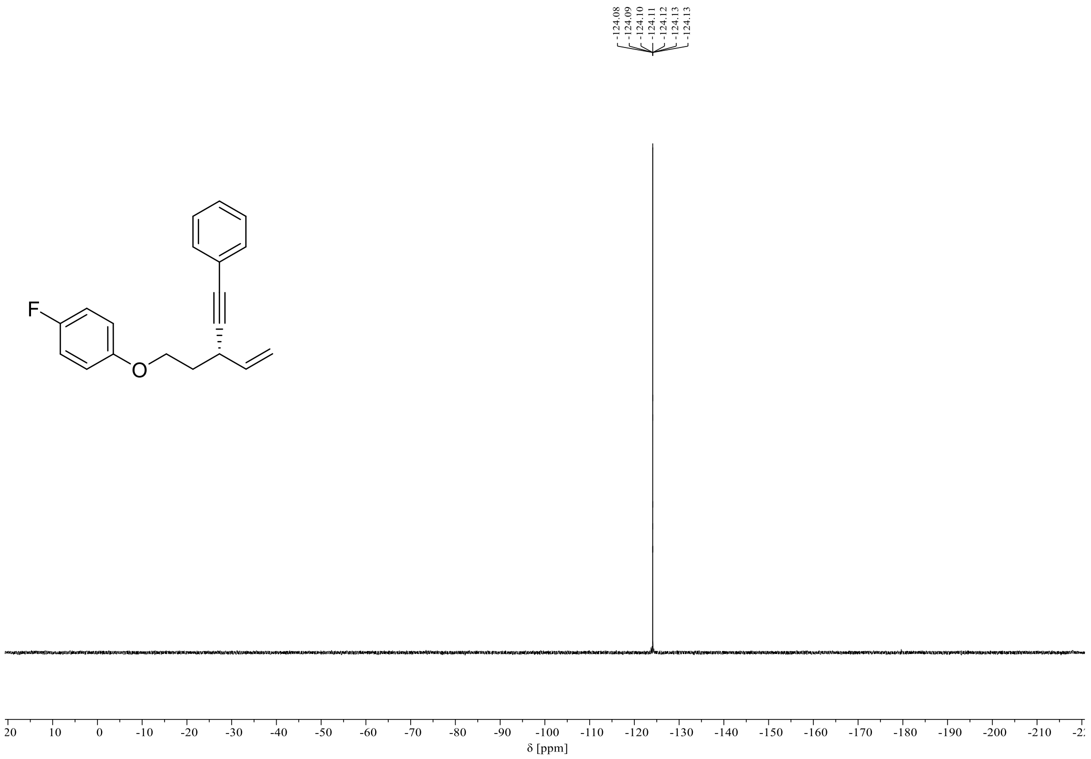

Figure 109: ^{19}F-NMR spectrum of (*S*)-1-fluoro-4-((3-(phenylethynyl)pent-4-en-1-yl)oxy)benzene ($CDCl_3$, 471 MHz).

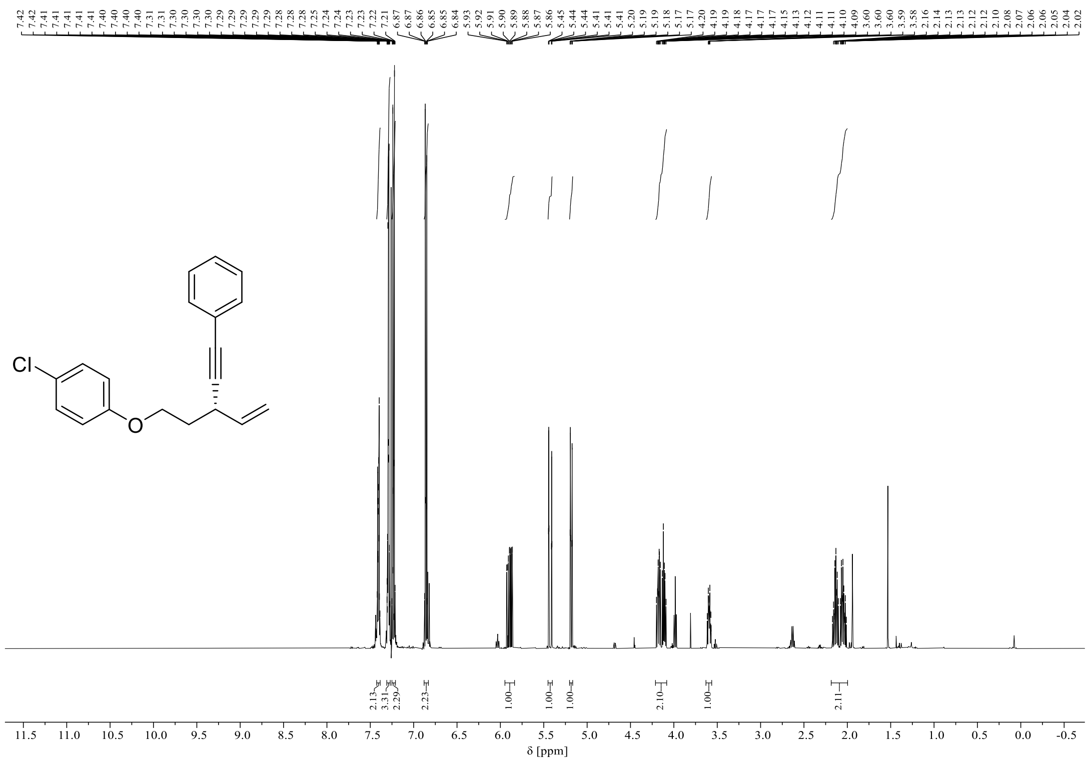

Figure 110: ^{1}H-NMR spectrum of (*S*)-1-chloro-4-((3-(phenylethynyl)pent-4-en-1-yl)oxy)benzene ($CDCl_3$, 500 MHz).

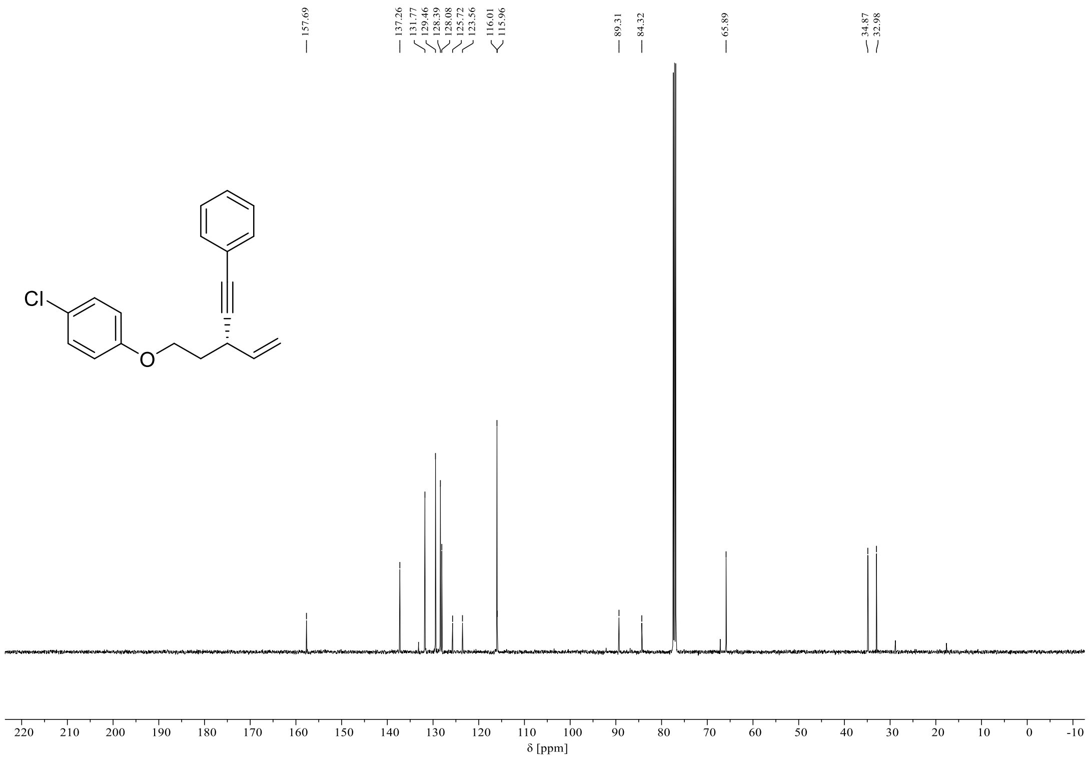

Figure 111: ^{13}C-NMR spectrum of (*S*)-1-chloro-4-((3-(phenylethynyl)pent-4-en-1-yl)oxy)benzene ($CDCl_3$, 126 MHz).

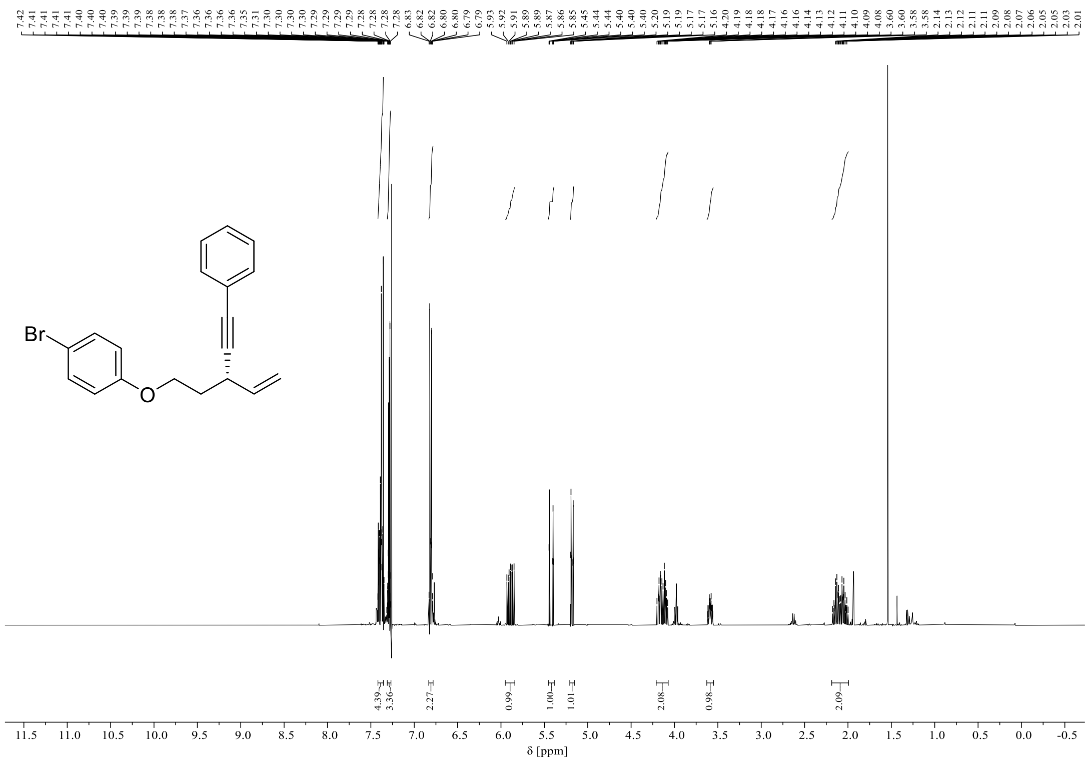

Figure 112: ^{1}H-NMR spectrum of (*S*)-1-bromo-4-((3-(phenylethynyl)pent-4-en-1-yl)oxy)benzene ($CDCl_3$, 400 MHz).

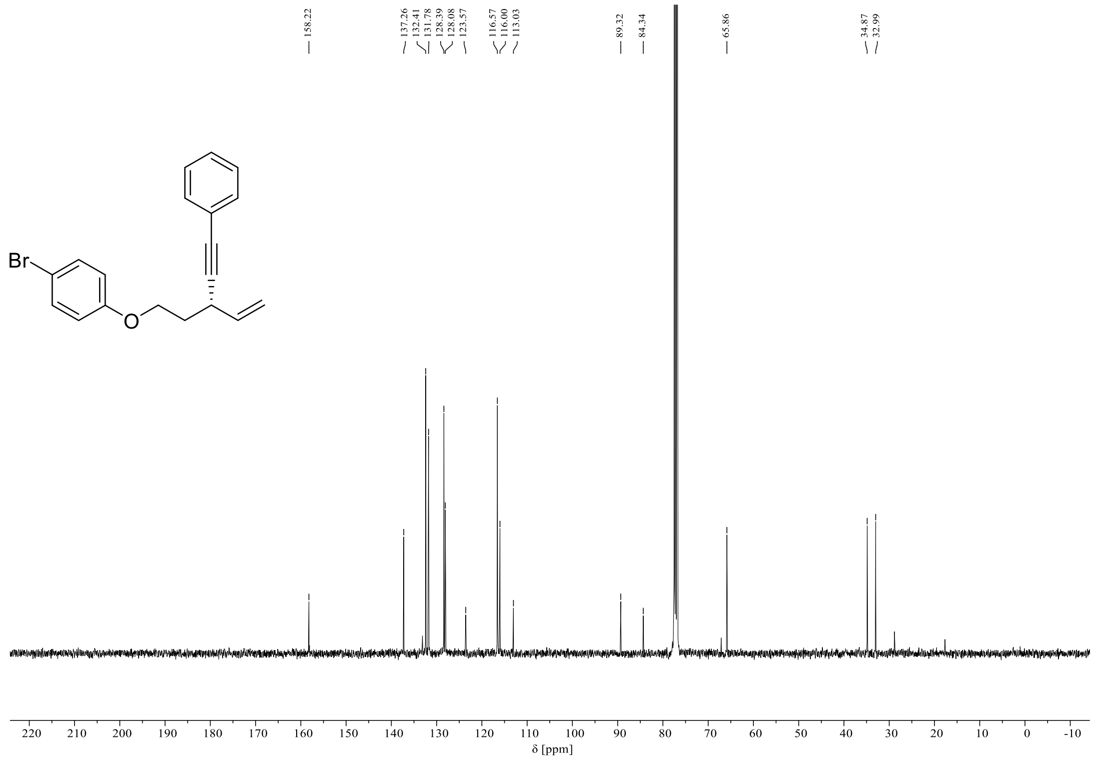

Figure 113: ^{13}C-NMR spectrum of (*S*)-1-bromo-4-((3-(phenylethynyl)pent-4-en-1-yl)oxy)benzene ($CDCl_3$, 101 MHz).

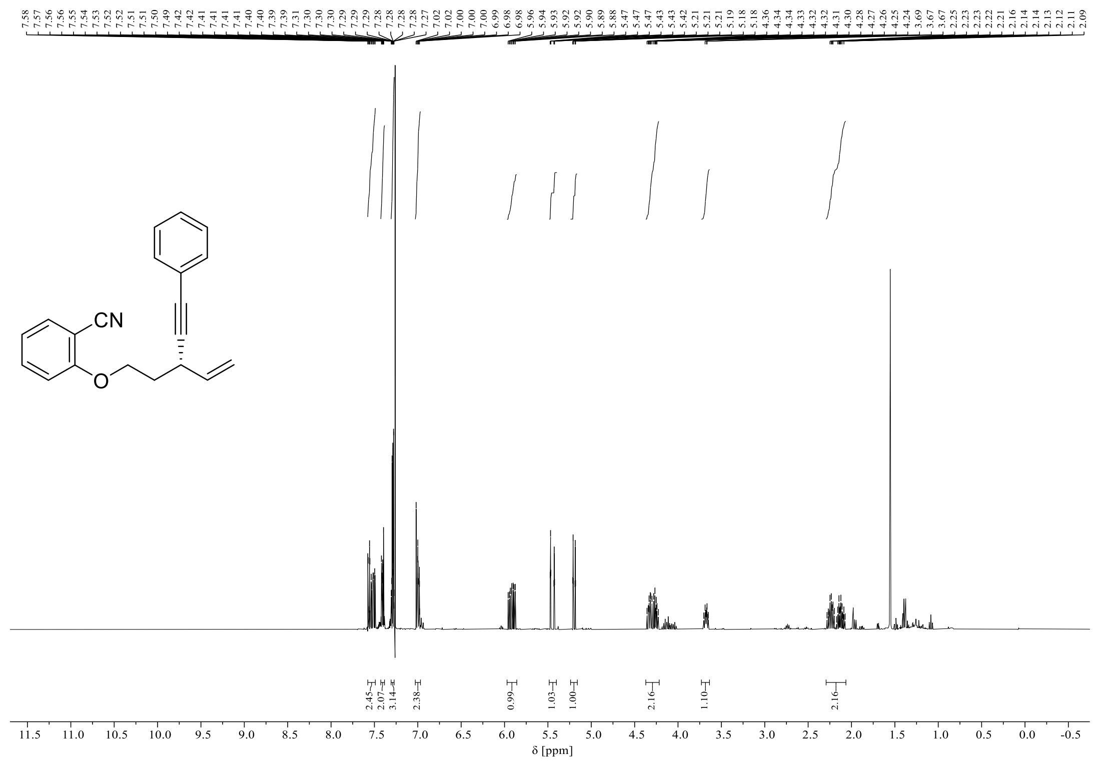

Figure 114: ^{1}H-NMR spectrum of (*S*)-2-((3-(phenylethynyl)pent-4-en-1-yl)oxy)benzonitrile ($CDCl_3$, 400 MHz).

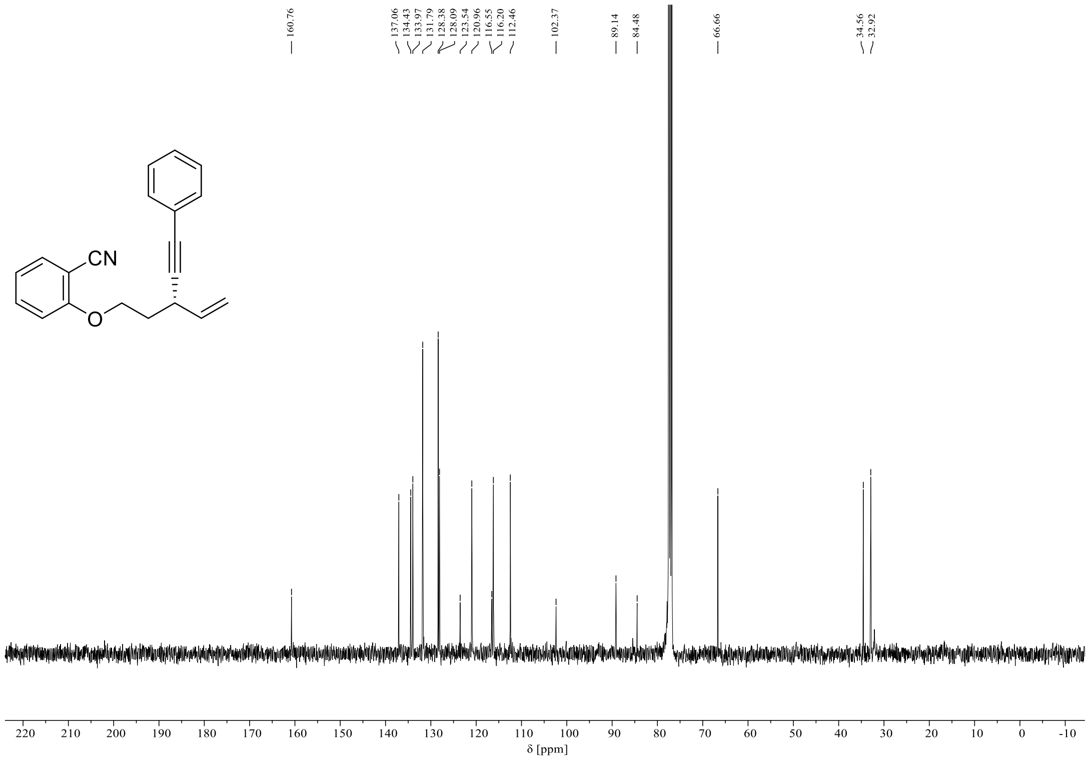

Figure 115: ^{13}C-NMR spectrum of (*S*)-2-((3-(phenylethynyl)pent-4-en-1-yl)oxy)benzonitrile ($CDCl_3$, 101 MHz).

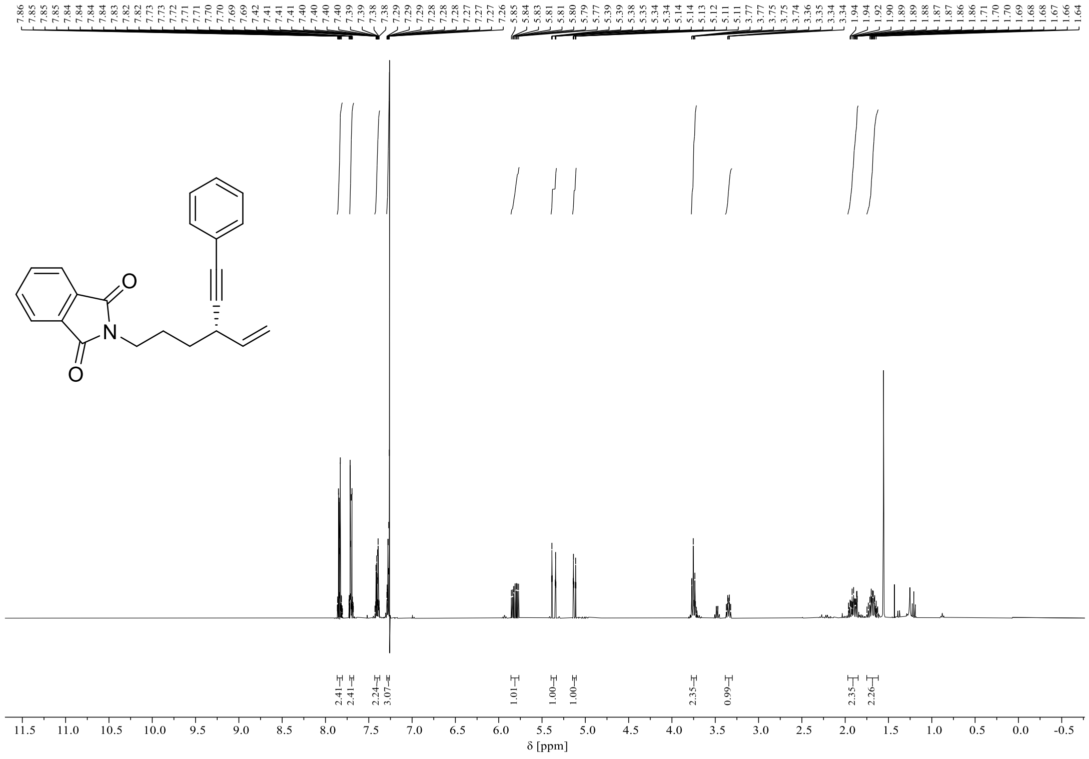

Figure 116: ^{1}H-NMR spectrum of (*S*)-2-(4-(phenylethynyl)hex-5-en-1-yl)isoindoline-1,3-dione ($CDCl_3$, 400 MHz).

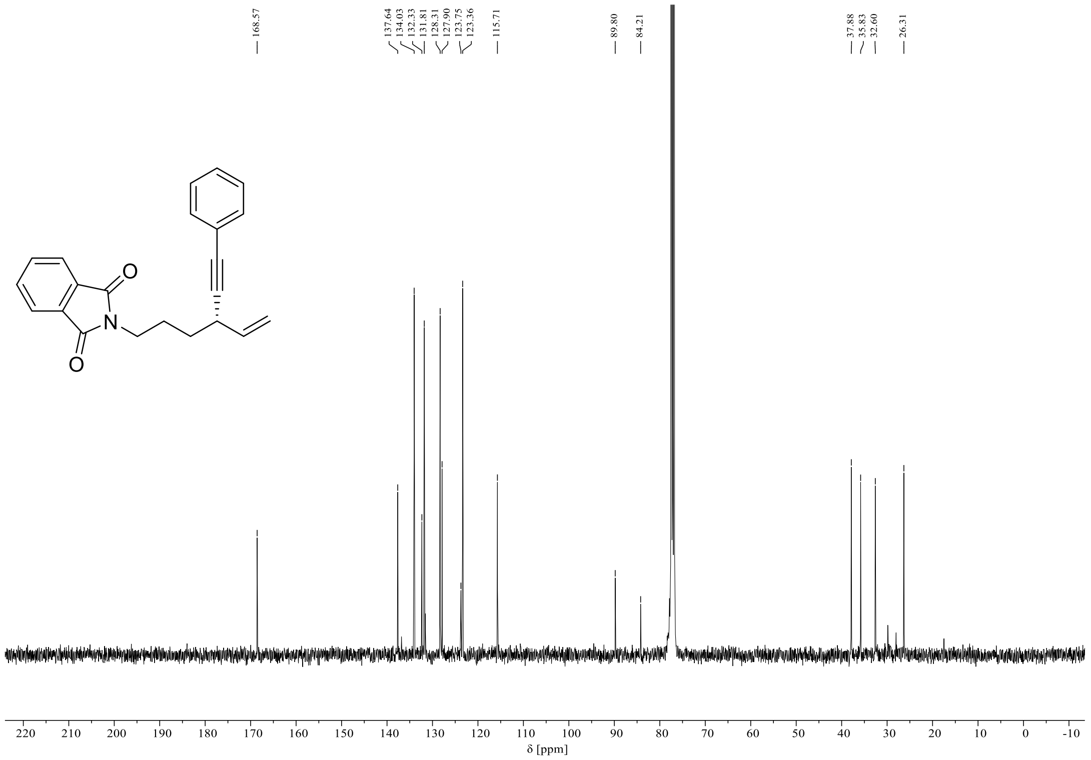

Figure 117: ^{13}C-NMR spectrum of (*S*)-2-(4-(phenylethynyl)hex-5-en-1-yl)isoindoline-1,3-dione ($CDCl_3$, 101 MHz).

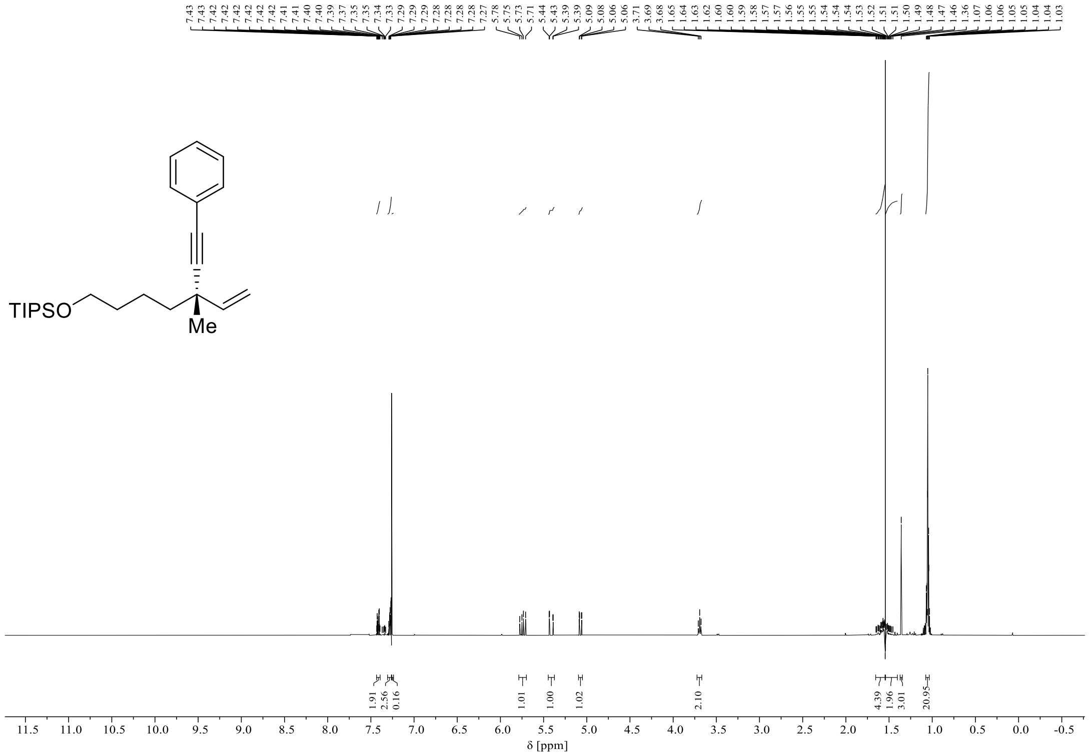

Figure 118: ^{1}H-NMR spectrum of (*S*)-triisopropyl((4-methyl-4-(phenylethynyl)hex-5-en-1-yl)oxy)silane ($CDCl_3$, 400 MHz).

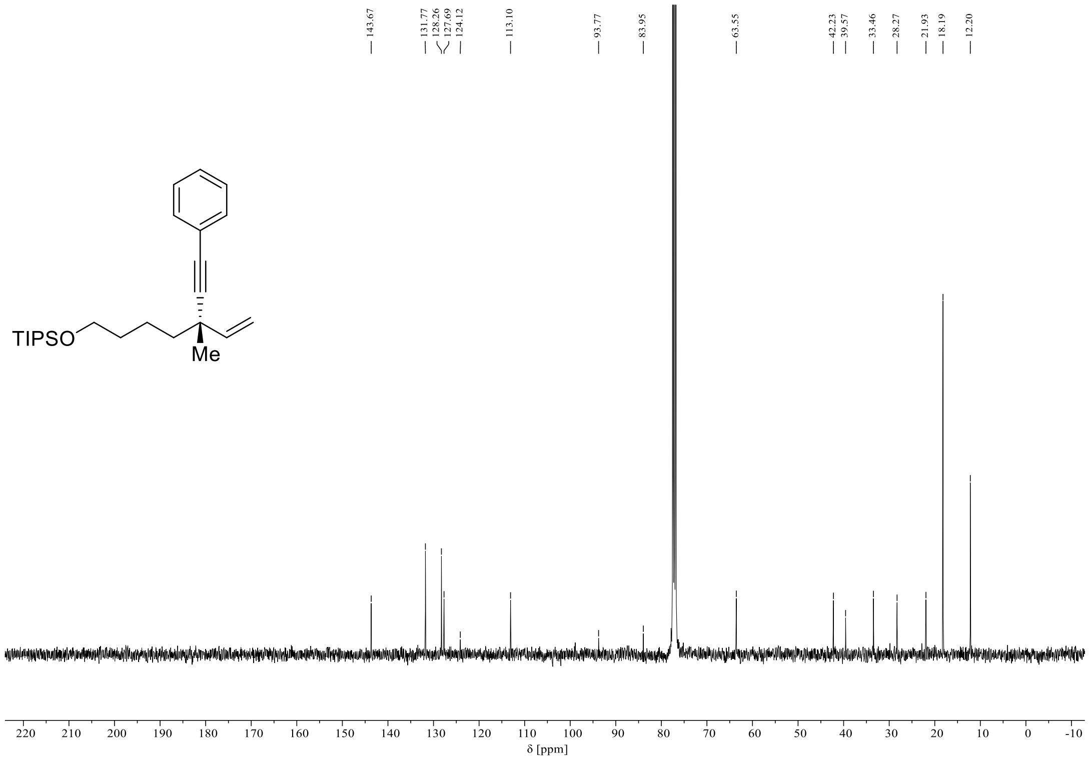

Figure 119: ^{13}C-NMR spectrum of (*S*)-triisopropyl((4-methyl-4-(phenylethynyl)hex-5-en-1-yl)oxy)silane ($CDCl_3$, 101 MHz).

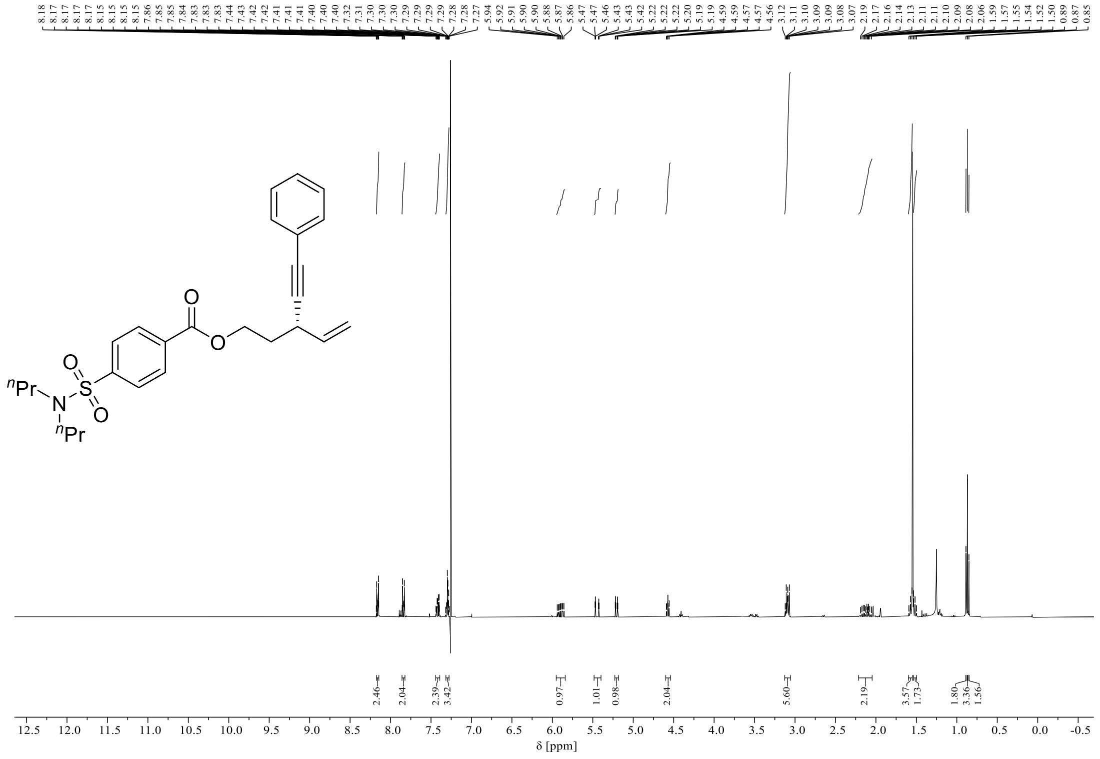

Figure 120: ^{1}H-NMR spectrum of (*S*)-3-(phenylethynyl)pent-4-en-1-yl 4-(*N*,*N*-dipropylsulfamoyl)benzoate ($CDCl_3$, 400 MHz).

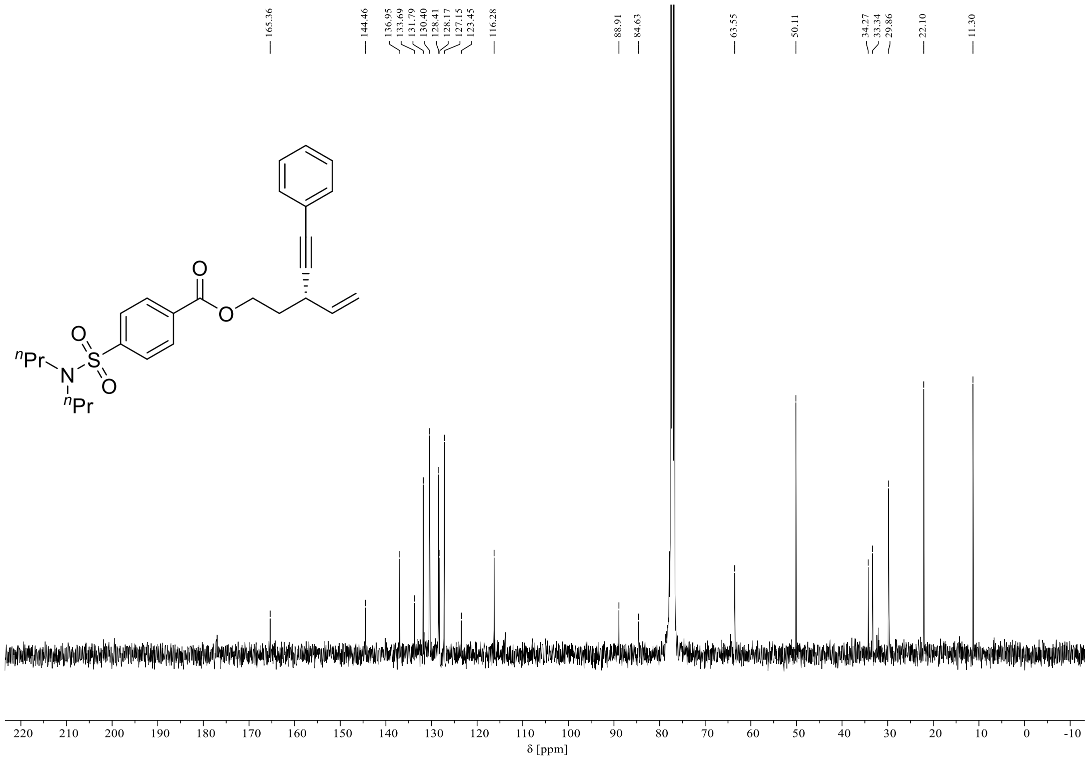

Figure 121: ^{13}C-NMR spectrum of (*S*)-3-(phenylethynyl)pent-4-en-1-yl 4-(*N*,*N*-dipropylsulfamoyl)benzoate ($CDCl_3$, 101 MHz).

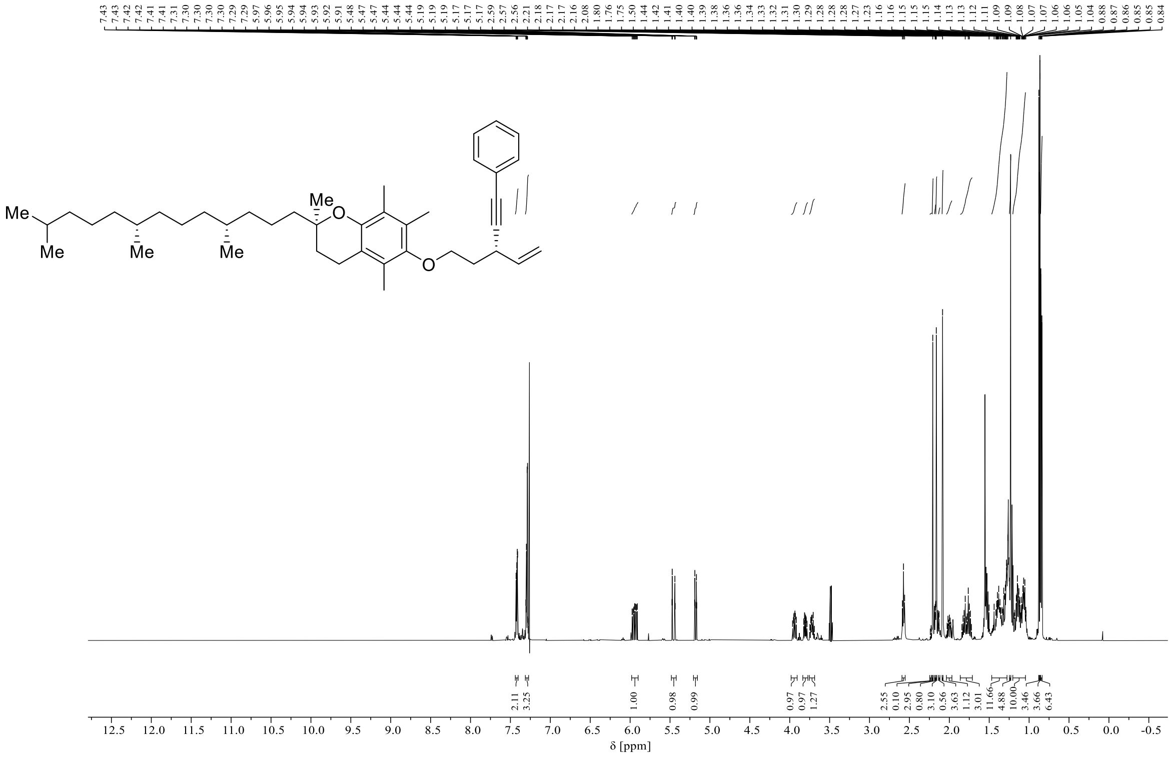

Figure 122: ^{1}H-NMR spectrum of (*R*)-2,5,7,8-tetramethyl-6-(((*S*)-3-(phenylethynyl)pent-4-en-1-yl)oxy)-2-((4*R*,8*R*)-4,8,12-trimethyltridecyl)chromane ($CDCl_3$, 500 MHz).

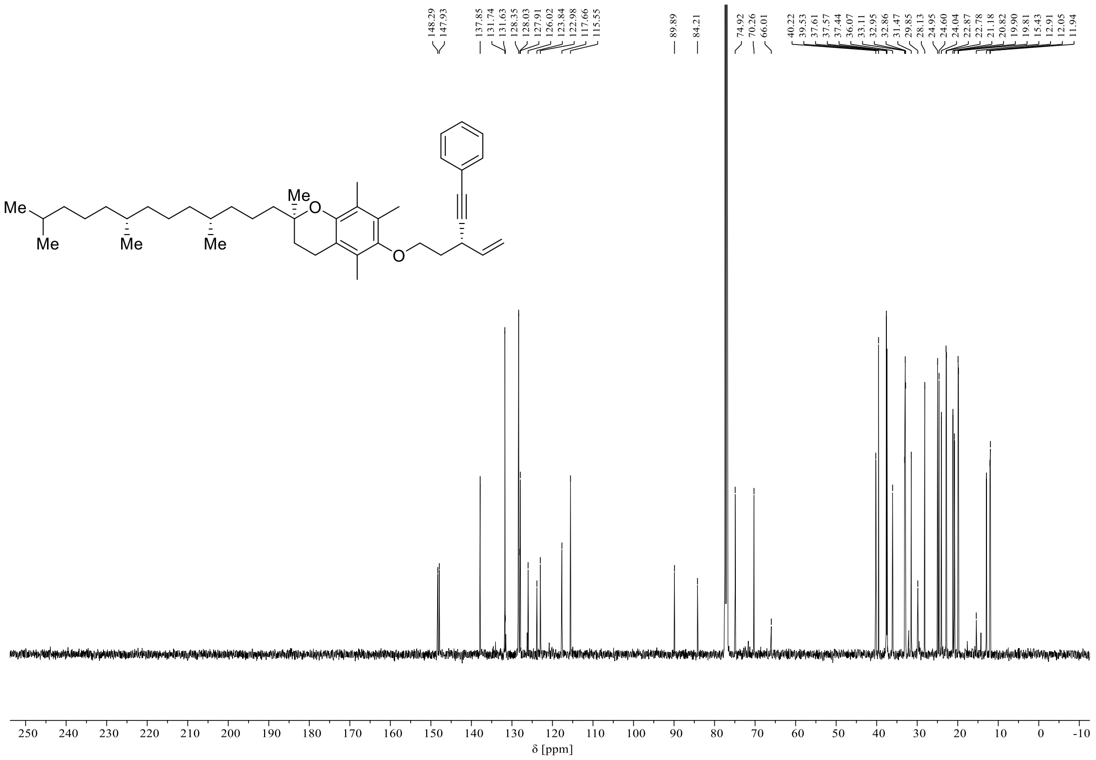

Figure 123: ^{13}C-NMR spectrum of (*R*)-2,5,7,8-tetramethyl-6-(((*S*)-3-(phenylethynyl)pent-4-en-1-yl)oxy)-2-((4*R*,8*R*)-4,8,12-trimethyltridecyl)chromane ($CDCl_3$, 126 MHz).

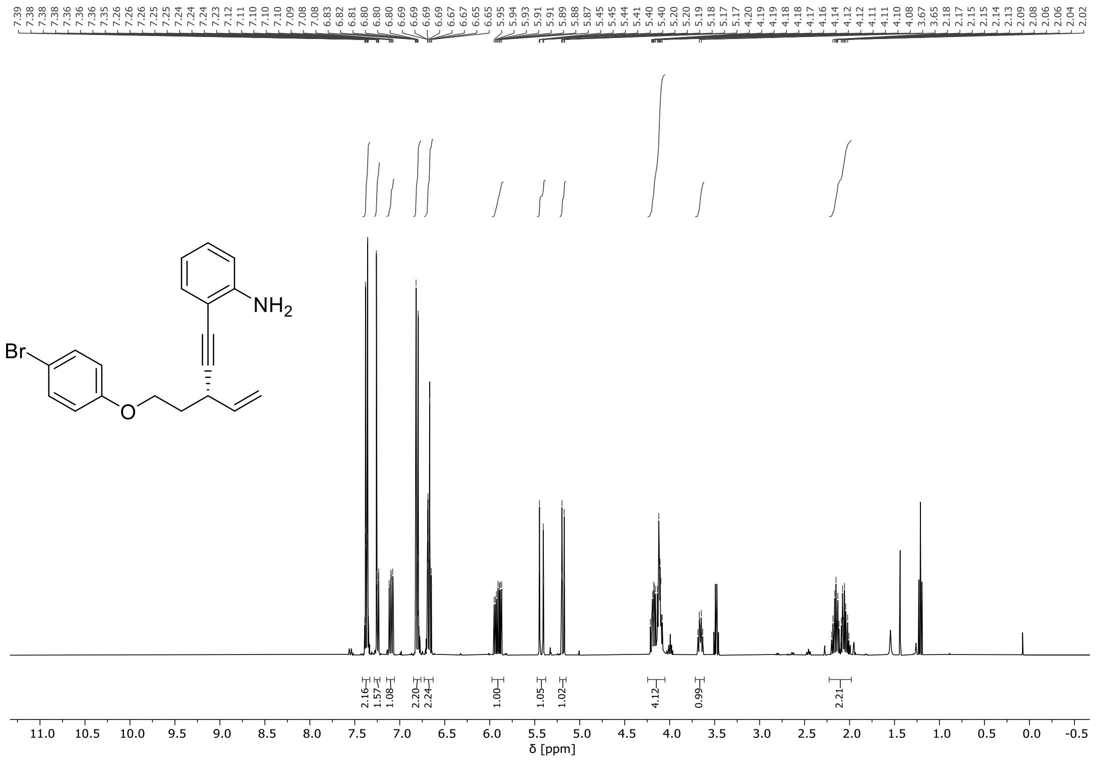

Figure 124: ^{1}H-NMR spectrum of (*S*)-2-(3-(2-(4-bromophenoxy)ethyl)pent-4-en-1-yn-1-yl)aniline ($CDCl_3$, 400 MHz, contains Et_2O).

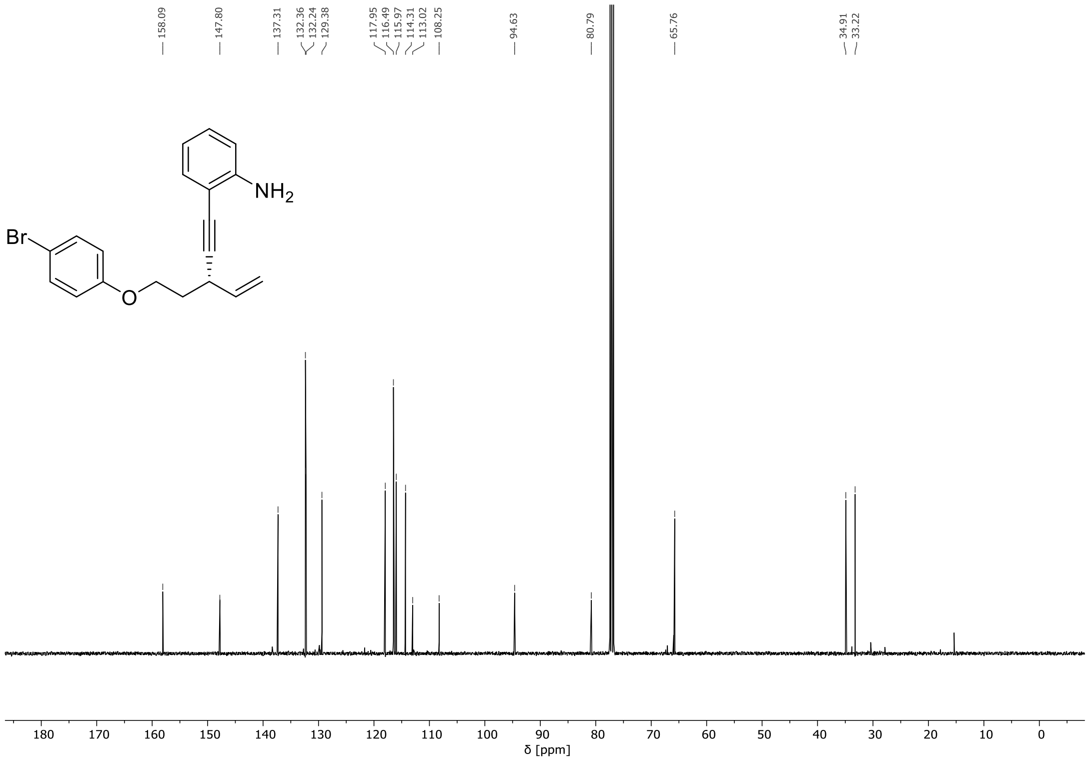

Figure 125: ^{13}C-NMR spectrum of (*S*)-2-(3-(2-(4-bromophenoxy)ethyl)pent-4-en-1-yn-1-yl)aniline ($CDCl_3$, 101 MHz).

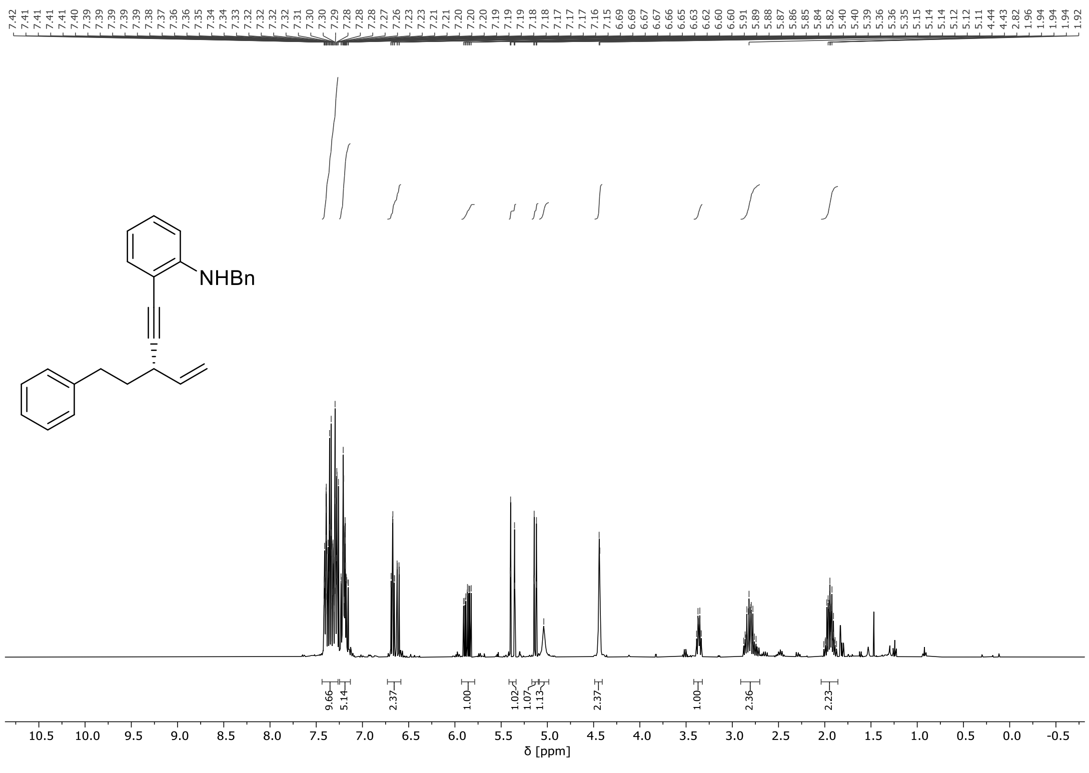

Figure 126: ^{1}H-NMR spectrum of (*S*)-*N*-benzyl-2-(3-phenethylpent-4-en-1-yn-1-yl)aniline ($CDCl_3$, 400 MHz).

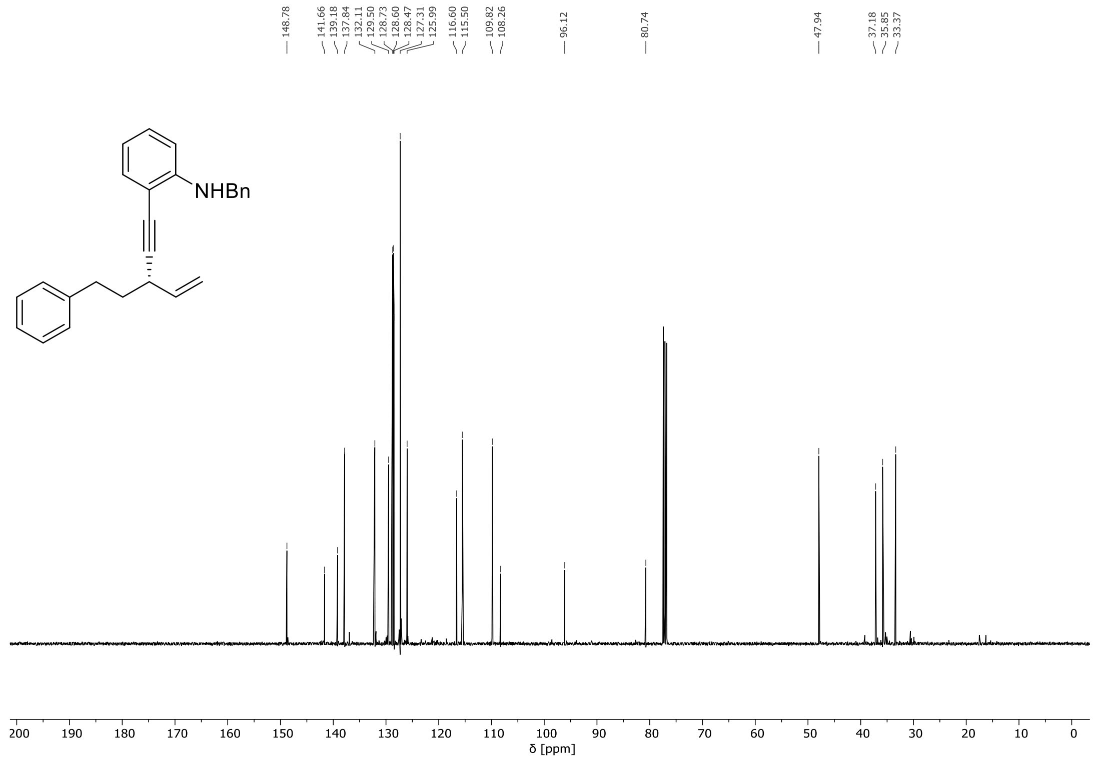

Figure 127: ^{13}C-NMR spectrum of (*S*)-*N*-benzyl-2-(3-phenethylpent-4-en-1-yn-1-yl)aniline ($CDCl_3$, 101 MHz).

k

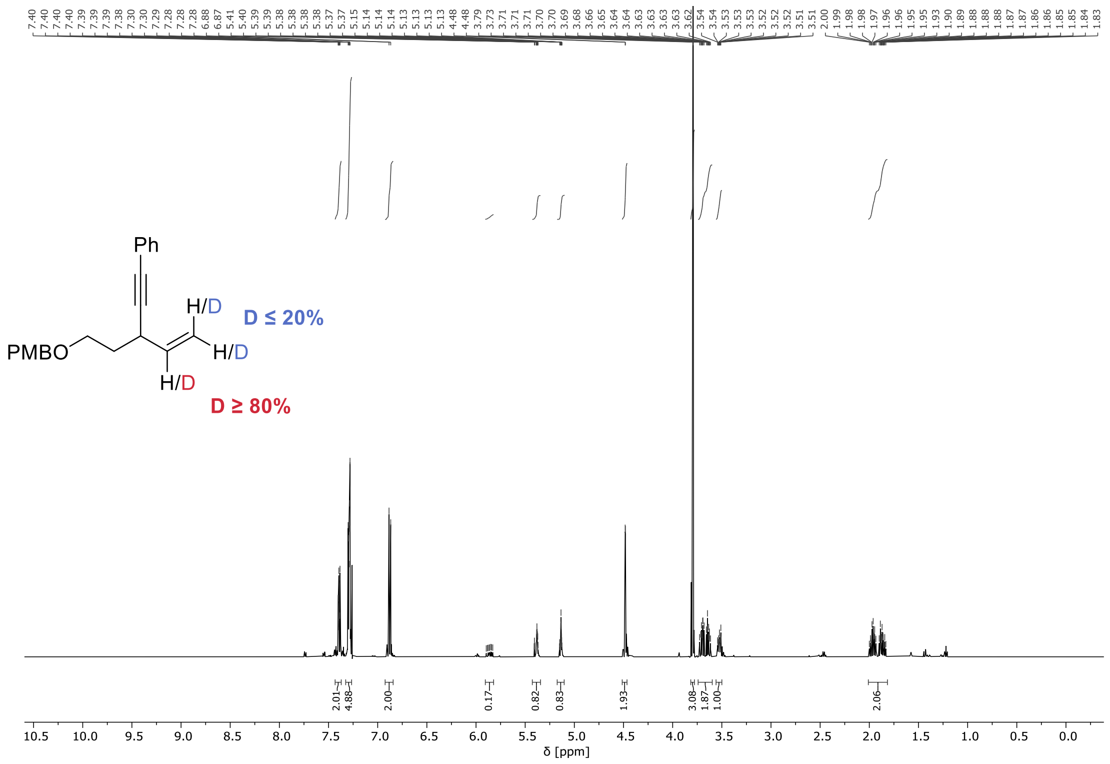

Figure 128: ^{1}H-NMR spectrum of deuterated (*S*)-1-methoxy-4-(((3-(phenylethynyl)pent-4-en-1-yl)oxy)methyl)benzene ($CDCl_3$, 500 MHz).

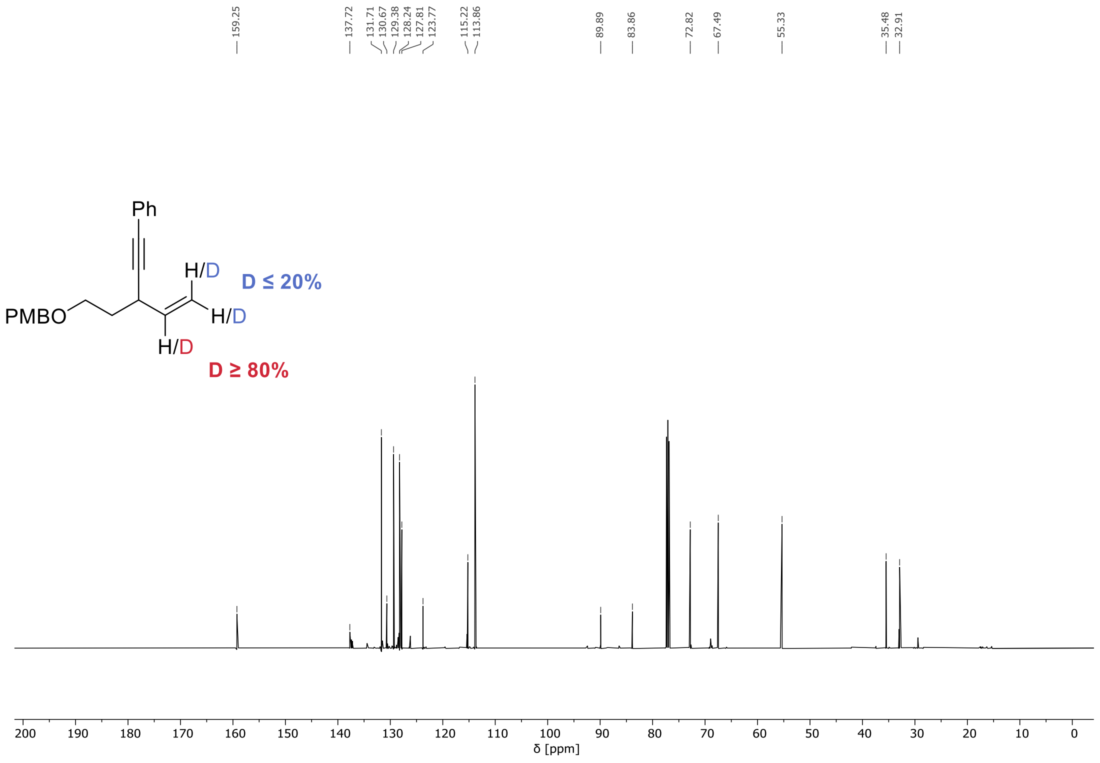

Figure 129: ^{13}C-NMR spectrum of deuterated (*S*)-1-methoxy-4-(((3-(phenylethynyl)pent-4-en-1-yl)oxy)methyl)benzene ($CDCl_3$, 126 MHz).

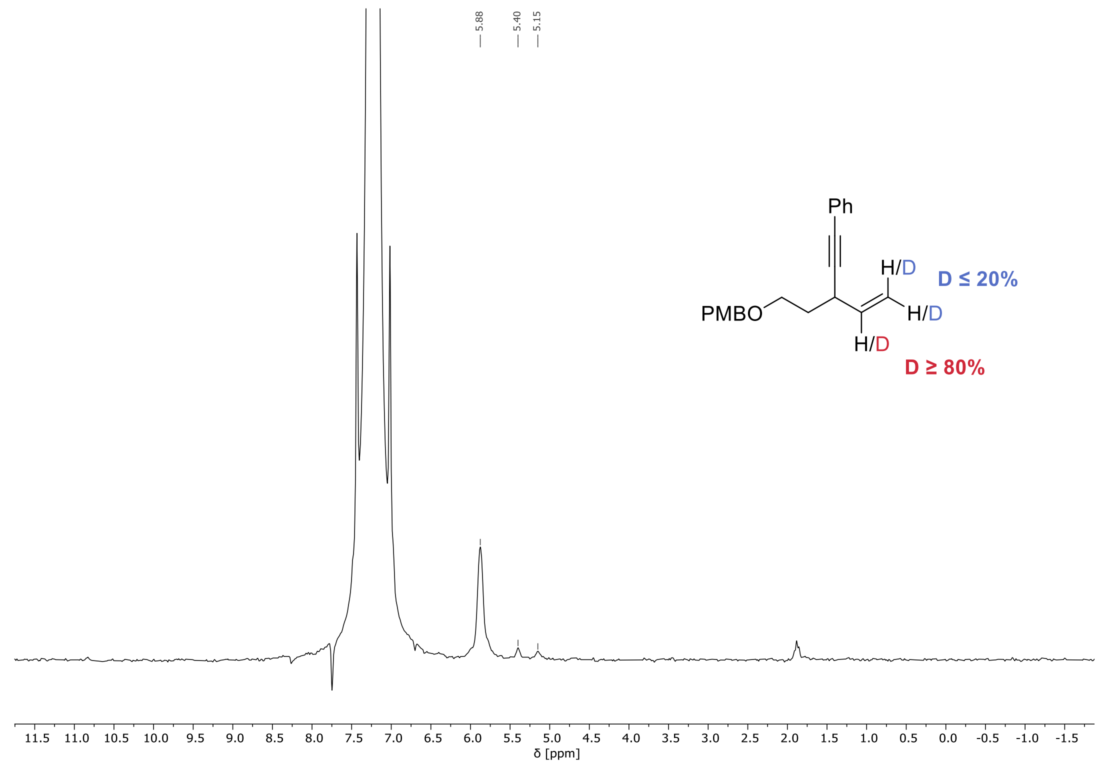

Figure 130: ^{2}H-NMR spectrum of deuterated (*S*)-1-methoxy-4-(((3-(phenylethynyl)pent-4-en-1-yl)oxy)methyl)benzene ($CDCl_3$, 77 MHz).

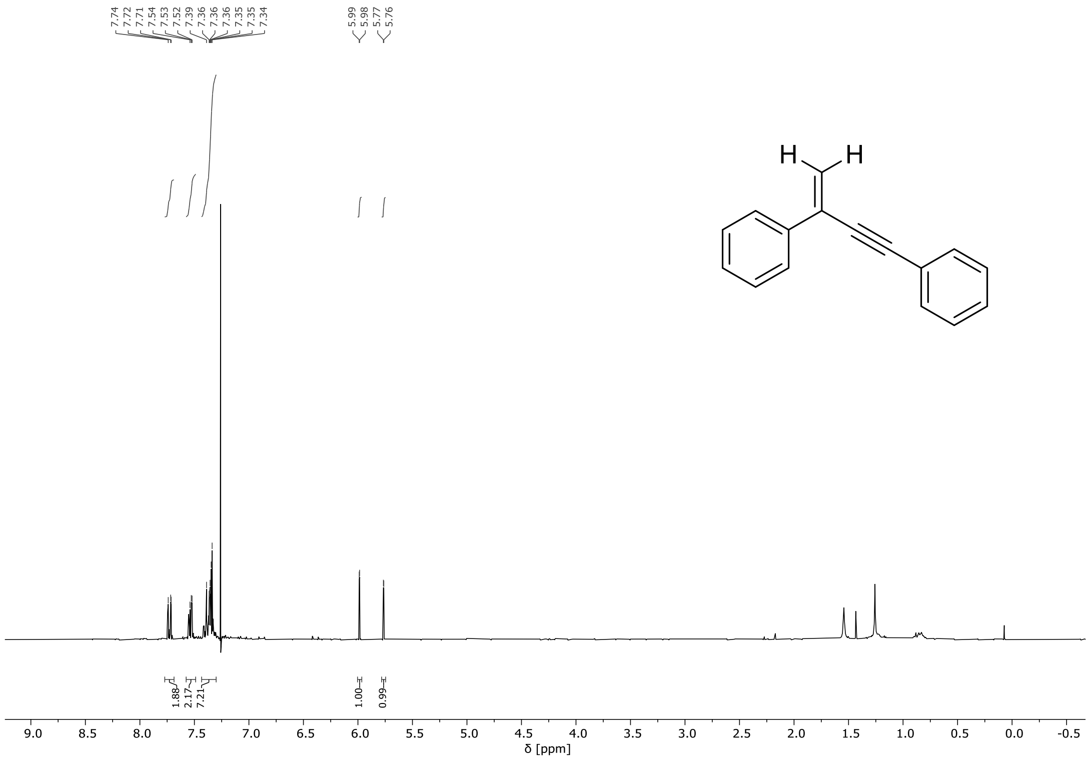

Figure 131: ^{1}H-NMR spectrum of but-3-en-1-yne-1,3-diyldibenzene ($CDCl_3$, 300 MHz).

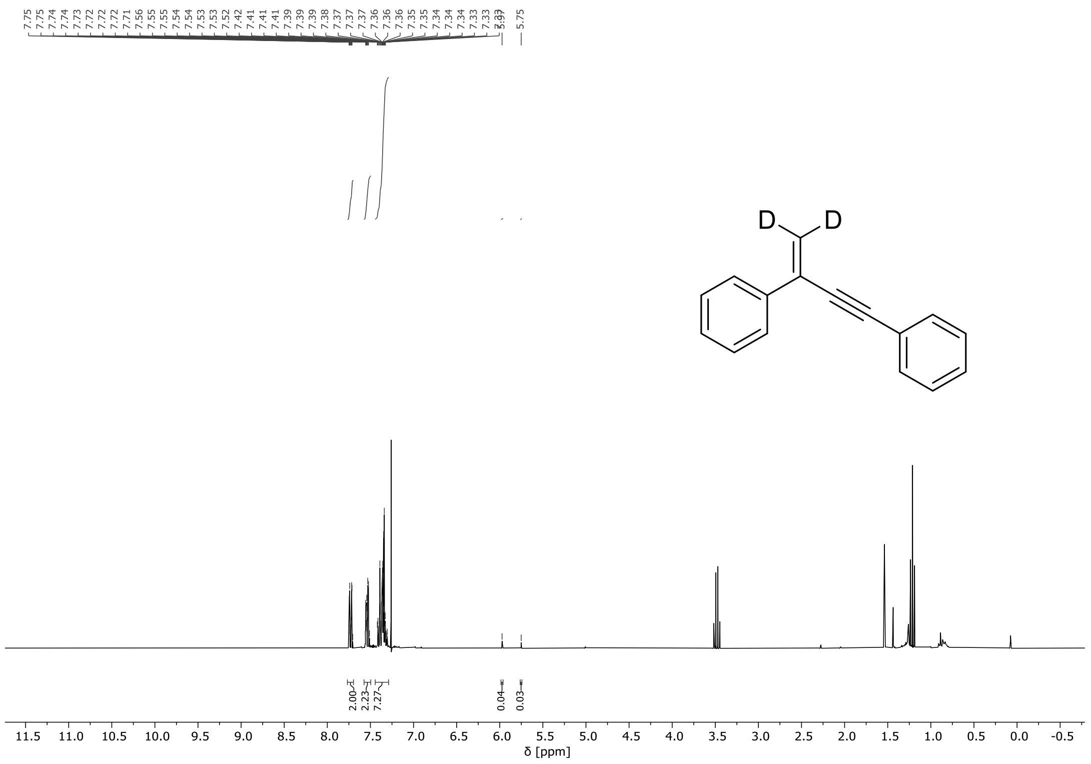

Figure 132: ^{1}H-NMR spectrum of deuterated but-3-en-1-yne-1,3-diyldibenzene ($CDCl_3$, 300 MHz, contains Et_2O).

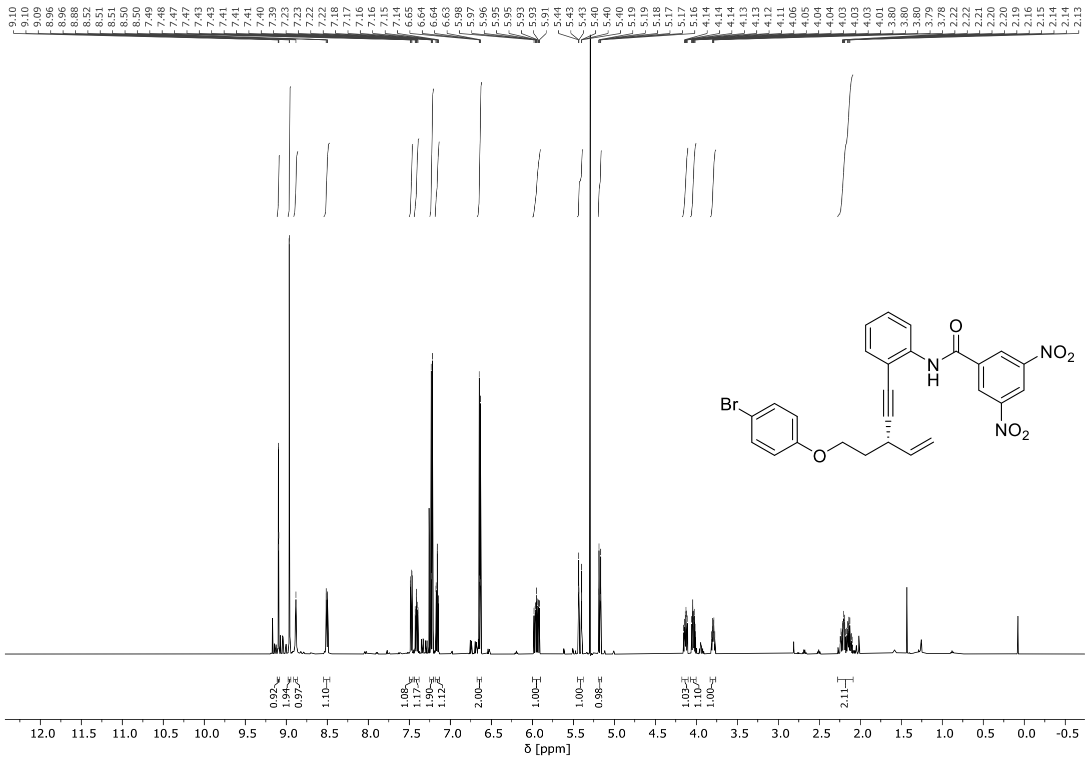

Figure 133: ^{1}H-NMR spectrum of (*S*)-N-(2-(3-(2-(4-bromophenoxy)ethyl)pent-4-en-1-yn-1-yl)phenyl)-3,5-dinitrobenzamide ($CDCl_3$, 500 MHz, contains CH_2Cl_2).

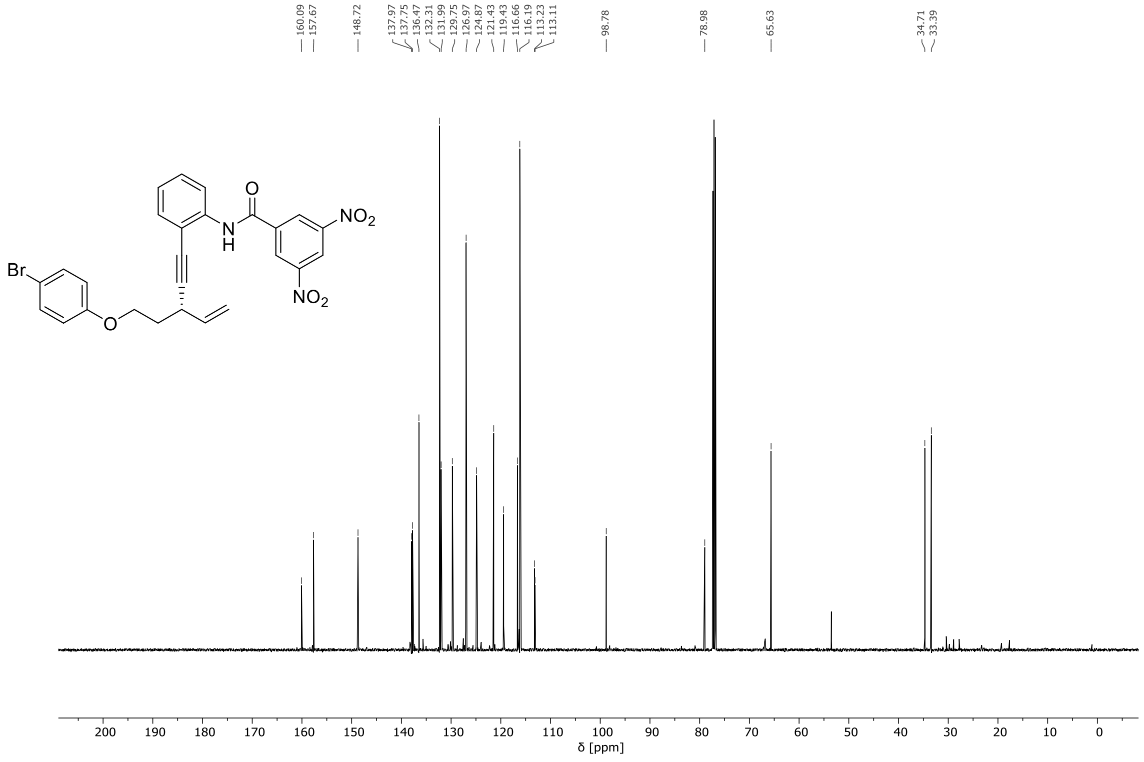

Figure 134: ^{13}C-NMR spectrum of (*S*)-N-(2-(3-(2-(4-bromophenoxy)ethyl)pent-4-en-1-yn-1-yl)phenyl)-3,5-dinitrobenzamide ($CDCl_3$, 126 MHz, contains CH_2Cl_2).

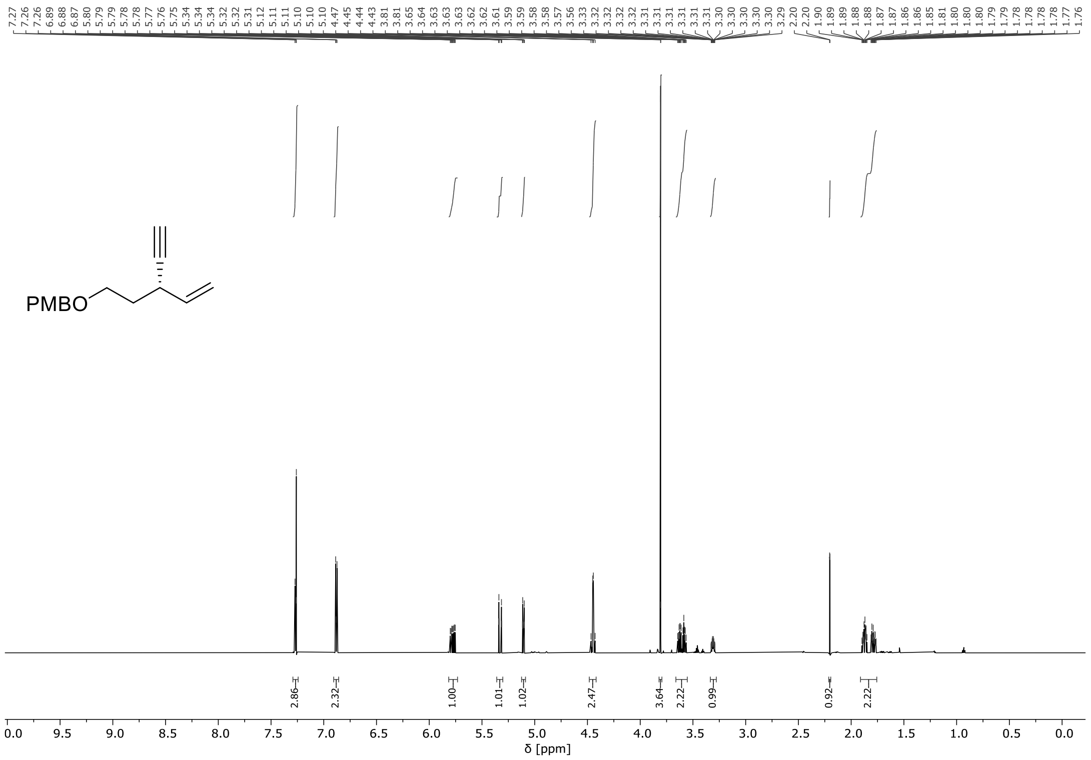

Figure 135: ^{1}H-NMR spectrum of (*S*)-1-(((3-ethynylpent-4-en-1-yl)oxy)methyl)-4-methoxybenzene ($CDCl_3$, 700 MHz).

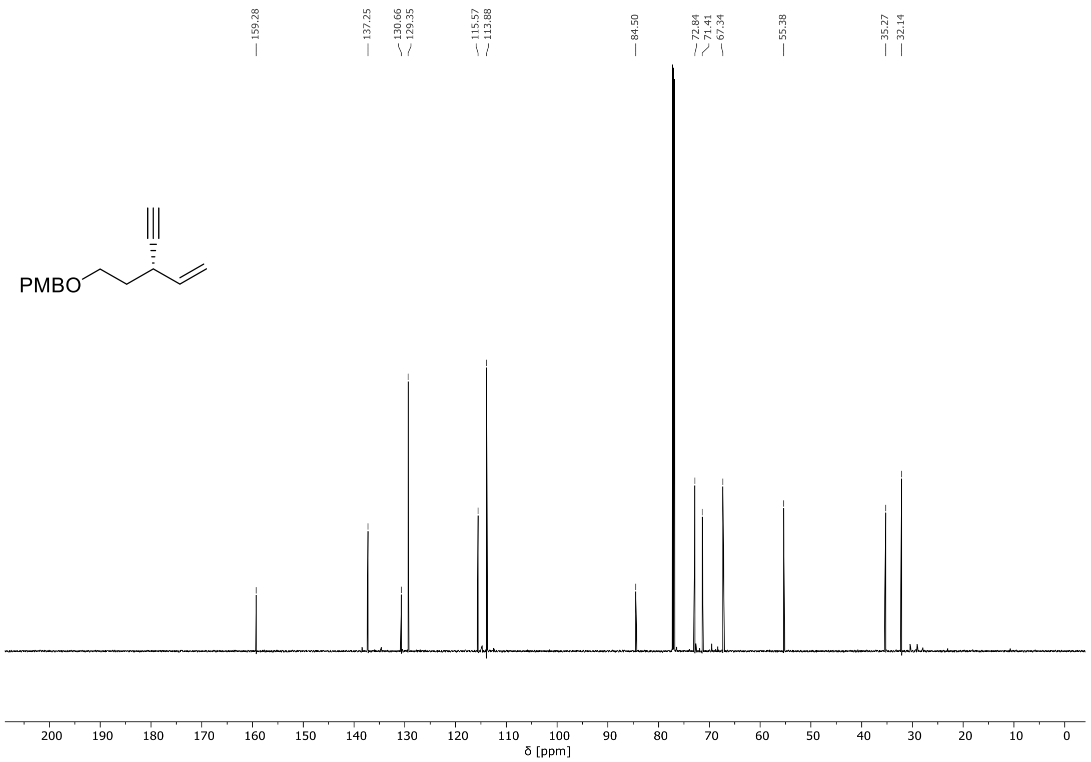

Figure 136: ^{13}C-NMR spectrum of (*S*)-1-(((3-ethynylpent-4-en-1-yl)oxy)methyl)-4-methoxybenzene ($CDCl_3$, 176 MHz).

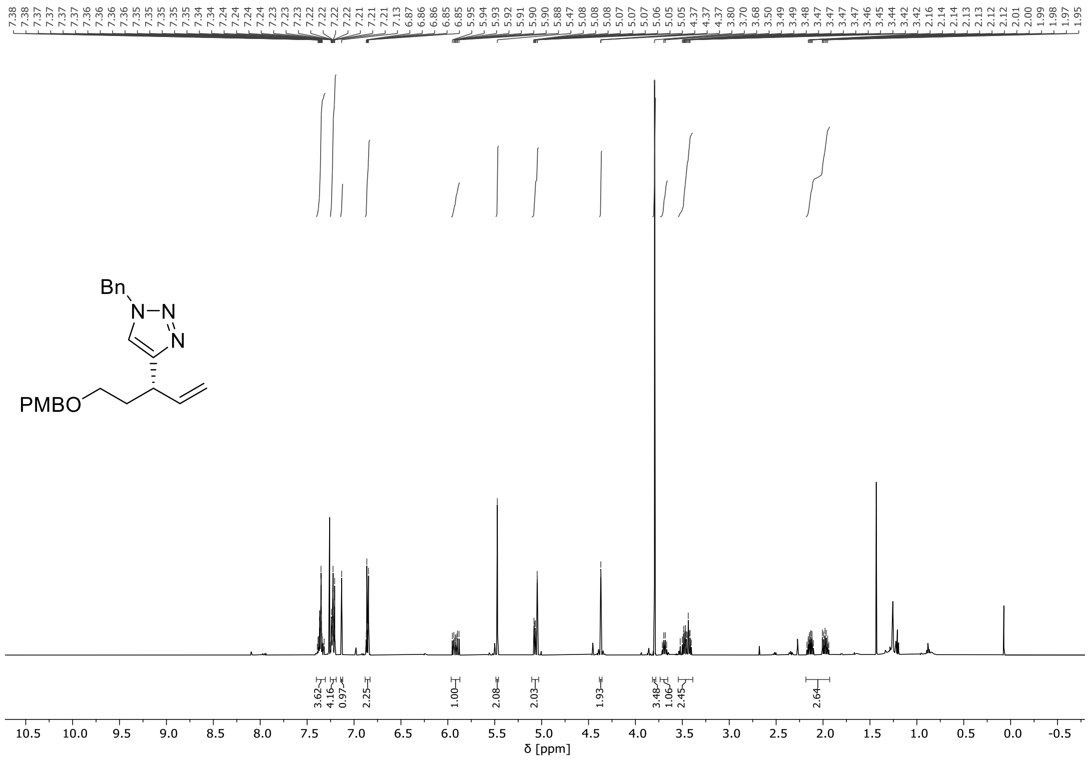

Figure 137: ^{1}H-NMR spectrum of (*S*)-1-benzyl-4-(5-((4-methoxybenzyl)oxy)pent-1-en-3-yl)-1H-1,2,3-triazole ($CDCl_3$, 500 MHz).

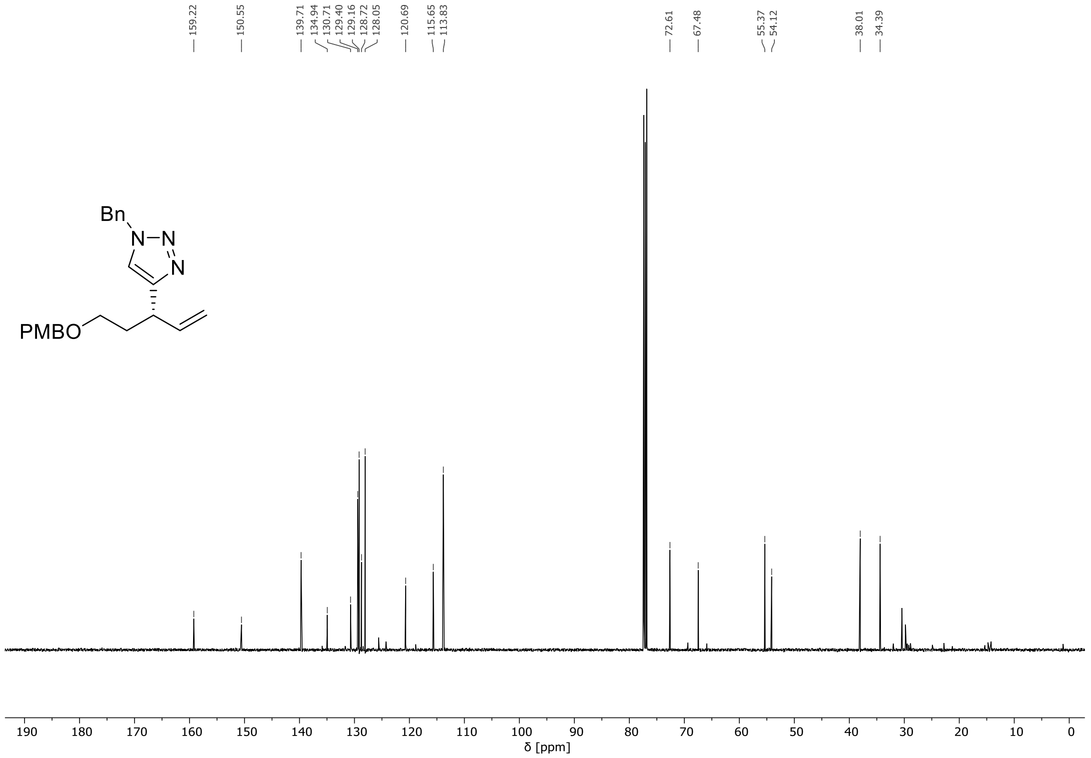

Figure 138: ^{13}C-NMR spectrum of (*S*)-1-benzyl-4-(5-((4-methoxybenzyl)oxy)pent-1-en-3-yl)-1H-1,2,3-triazole ($CDCl_3$, 126 MHz).

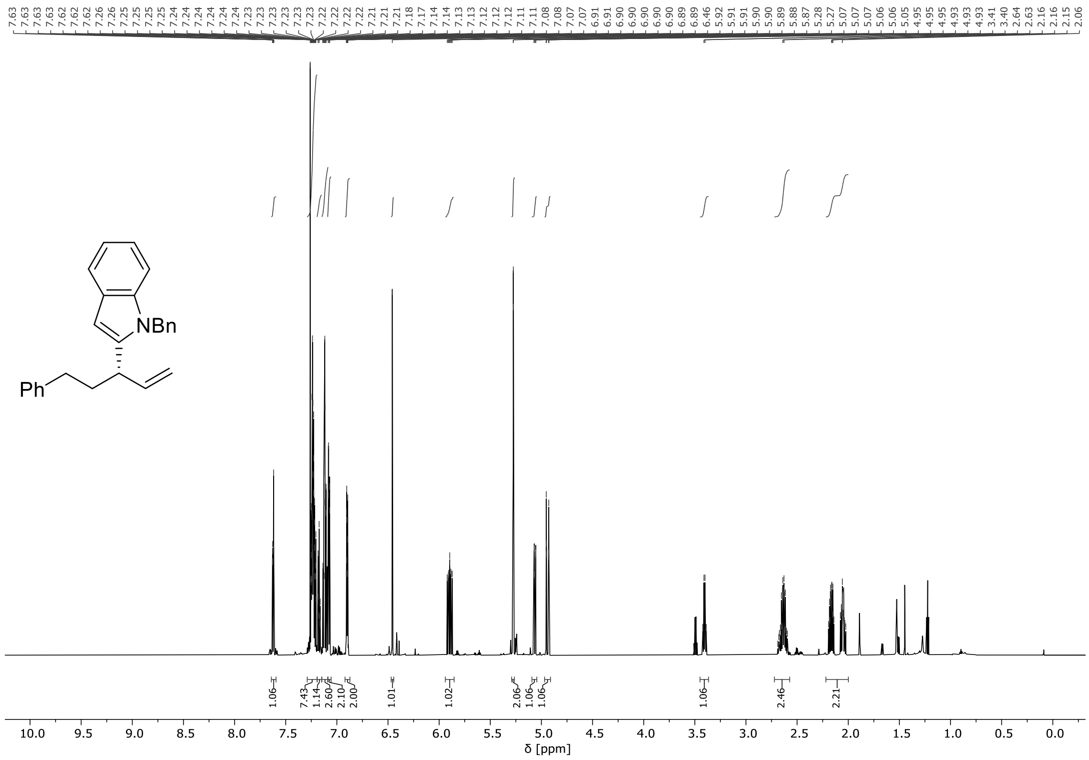

Figure 139: ^{1}H-NMR spectrum of (*S*)-1-benzyl-2-(5-phenylpent-1-en-3-yl)-1H-indole ($CDCl_3$, 700 MHz, contains Et_2O).

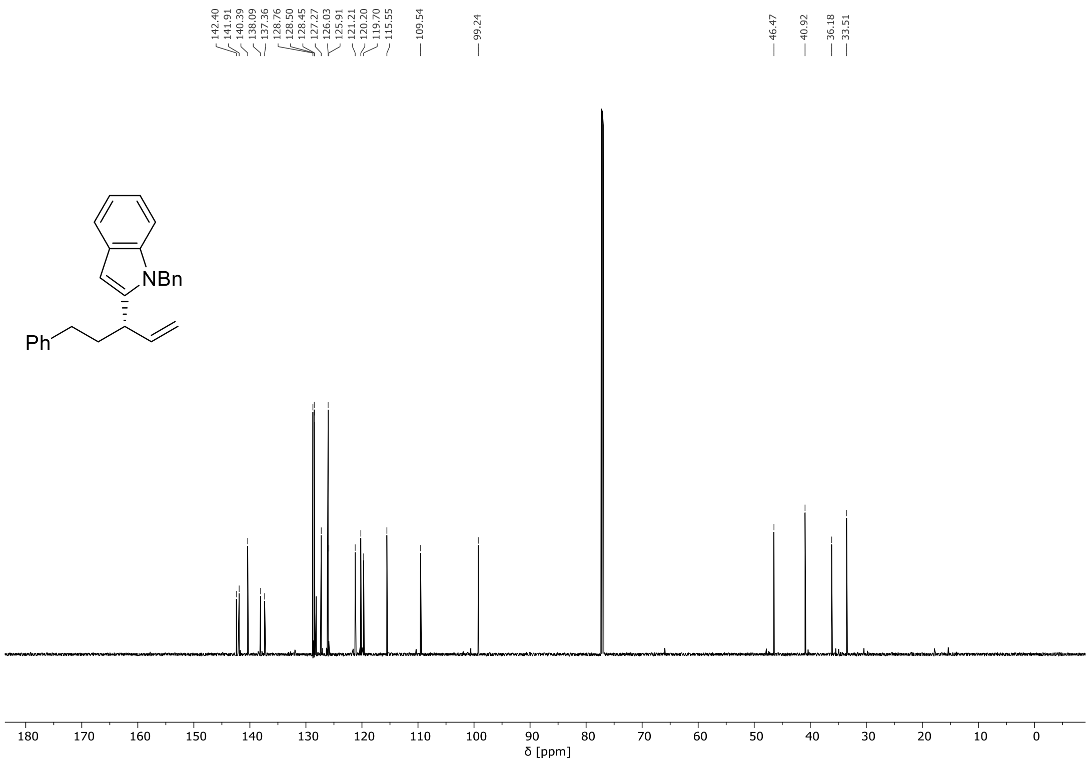

Figure 140: ^{13}C-NMR spectrum of (*S*)-1-benzyl-2-(5-phenylpent-1-en-3-yl)-1H-indole ($CDCl_3$, 176 MHz).

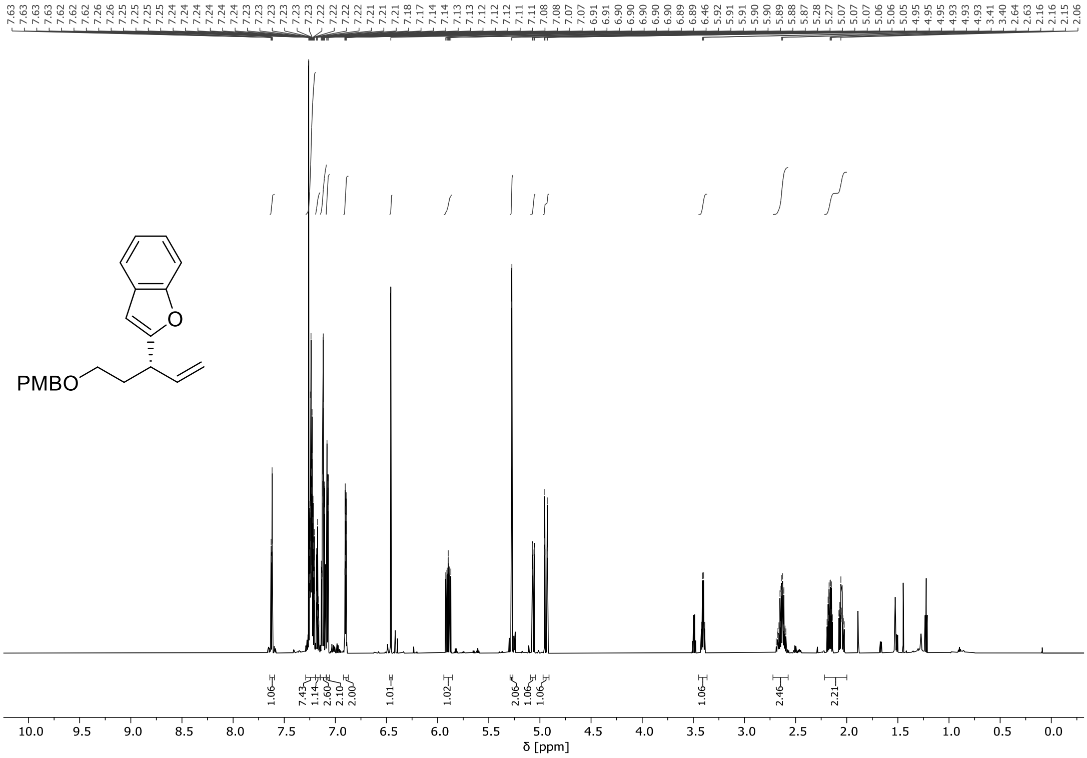

Figure 141: ^{1}H-NMR spectrum of (*S*)-2-(5-((4-methoxybenzyl)oxy)pent-1-en-3-yl)benzofuran ($CDCl_3$, 400 MHz).

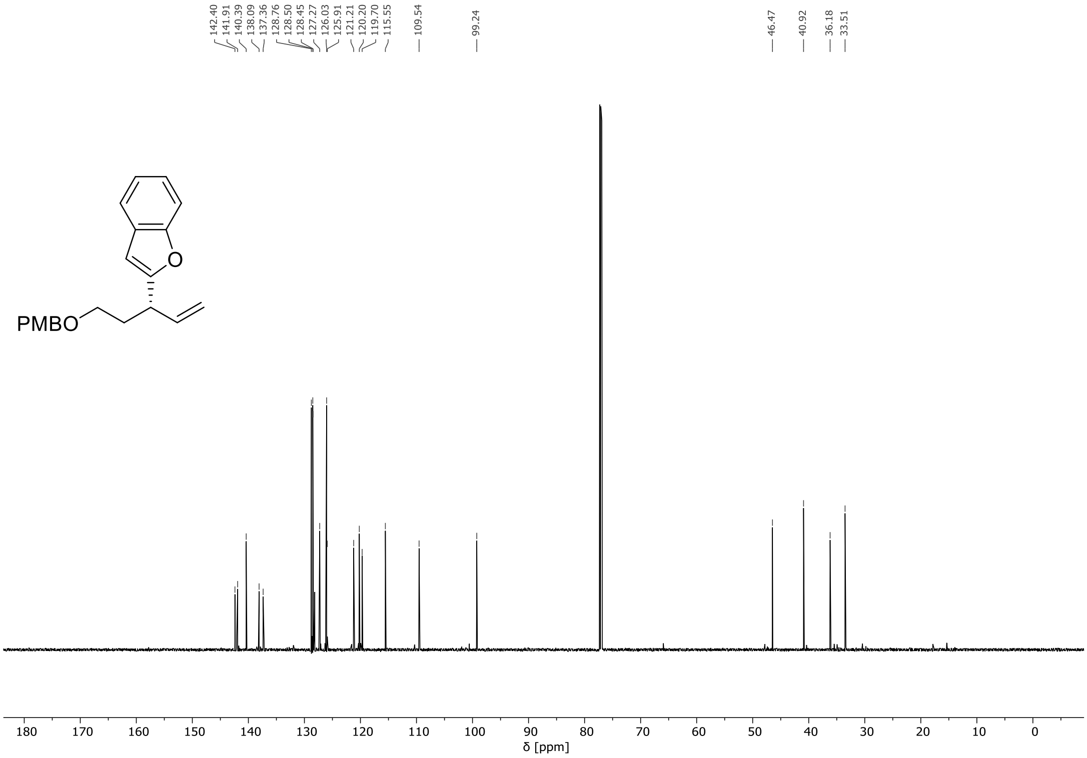

Figure 142: ^{13}C-NMR spectrum of (*S*)-2-(5-((4-methoxybenzyl)oxy)pent-1-en-3-yl)benzofuran ($CDCl_3$, 101 MHz).

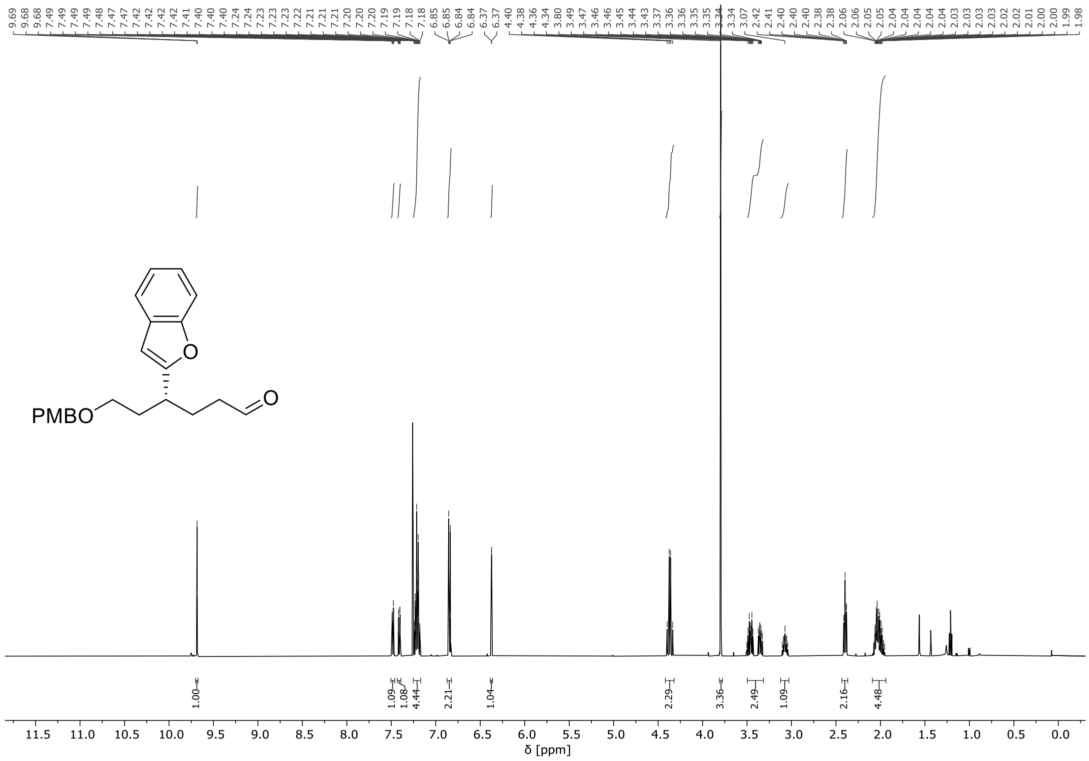

Figure 143: ^{1}H-NMR spectrum of (*R*)-4-(benzofuran-2-yl)-6-((4-methoxybenzyl)oxy)hexanal ($CDCl_3$, 500 MHz).

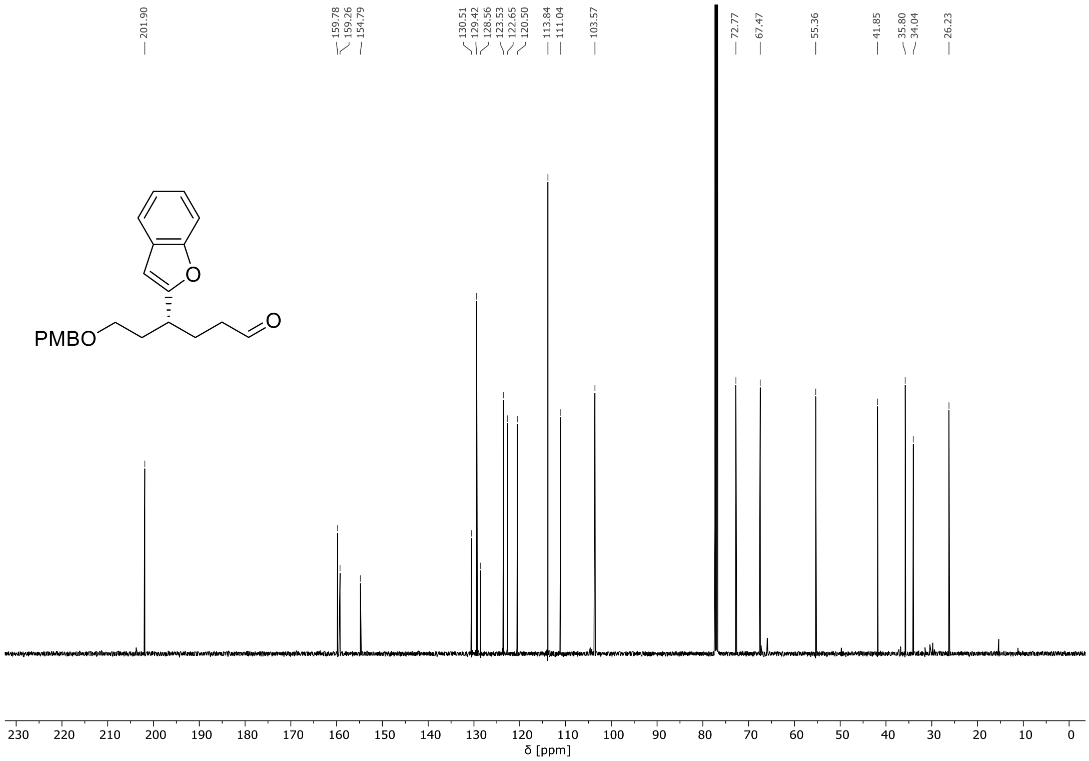

Figure 144: ^{13}C-NMR spectrum of (*R*)-4-(benzofuran-2-yl)-6-((4-methoxybenzyl)oxy)hexanal ($CDCl_3$, 126 MHz).

8.3 HPLC Chromatograms

(*S*)-1-(((3-(Cyclohexylethynyl)pent-4-en-1-yl)oxy)methyl)-4-methoxybenzene

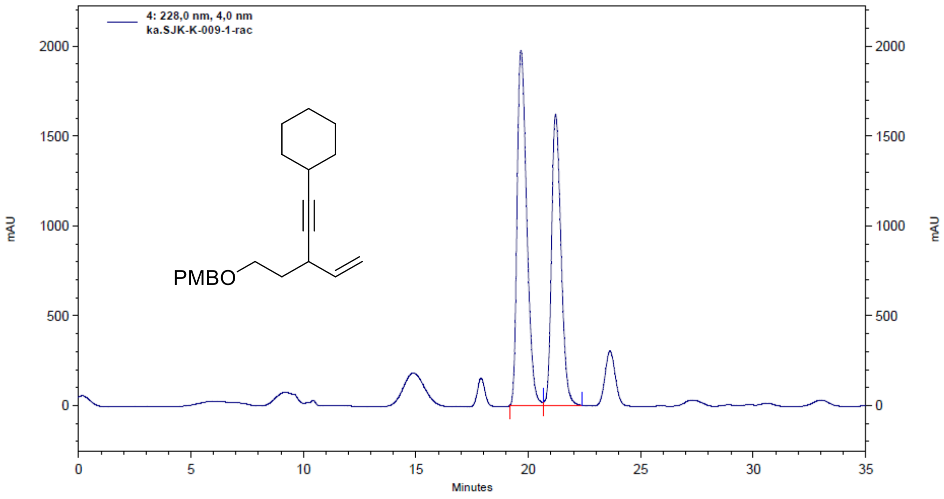

Peak Index	Time / min	Area / %
1	19.7	55.349
2	21.2	44.651

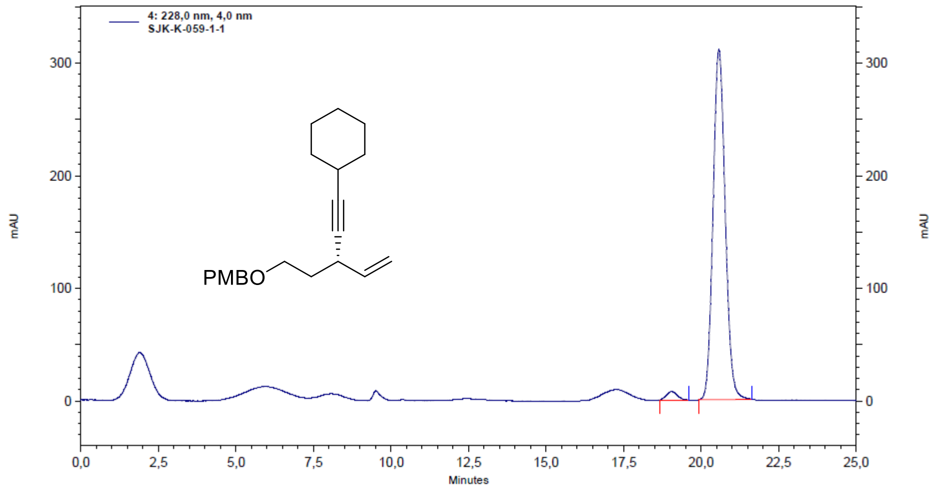

Peak Index	Time / min	Area / %
1	19.1	1.972
2	20.6	98.028

(*S*)-1-Methoxy-4-(((3-(phenylethynyl)pent-4-en-1-yl)oxy)methyl)benzene

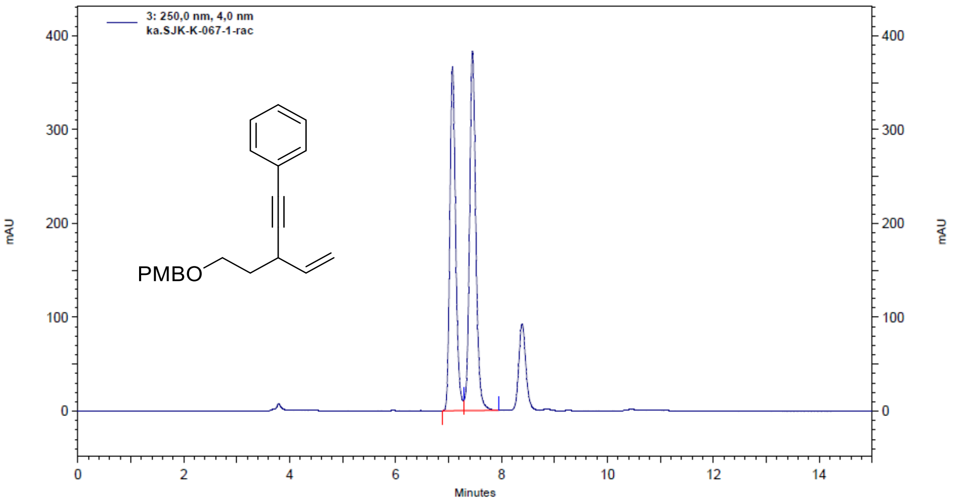

Peak Index	Time / min	Area / %
1	7.1	46.783
2	7.5	53.217

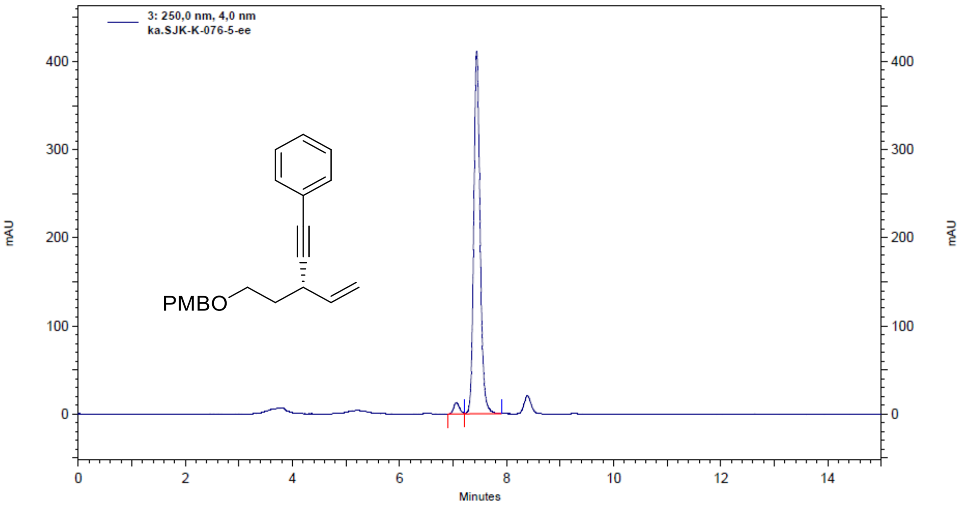

Peak Index	Time / min	Area / %
1	7.1	2.823
2	7.4	97.177

(*S*)-1-Fluoro-4-(3-(2-((4-methoxybenzyl)oxy)ethyl)pent-4-en-1-yn-1-yl)benzene

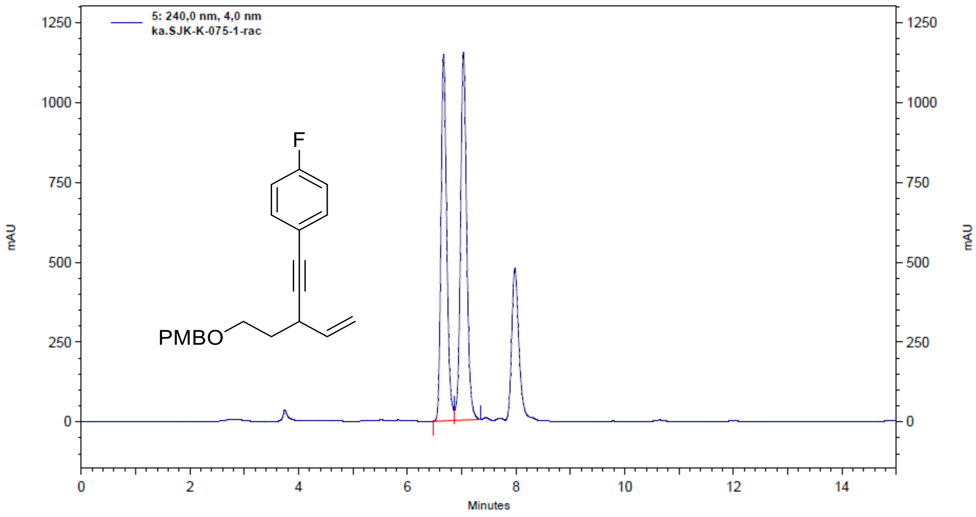

Peak Index	Time / min	Area / %
1	6.7	47.994
2	7.0	52.006

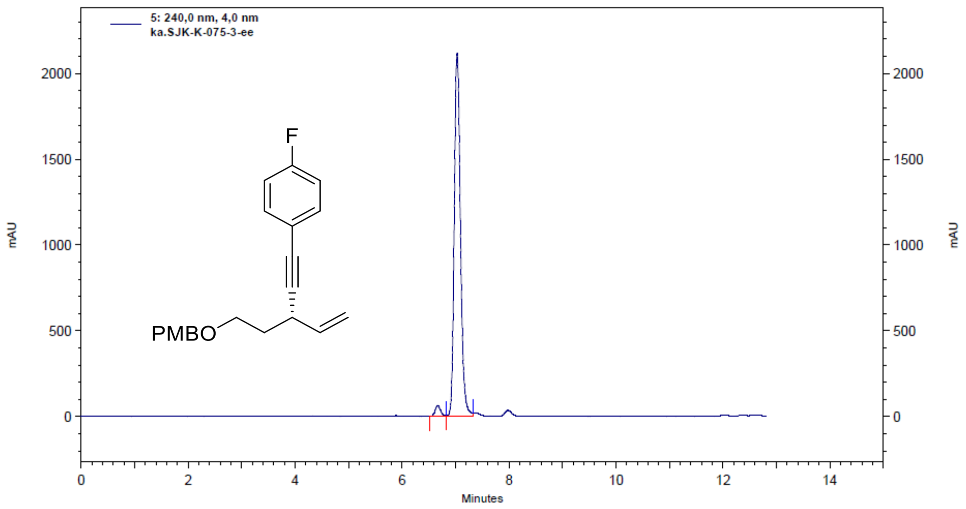

Peak Index	Time / min	Area / %
1	6.7	2.706
2	7.0	97.294

(*S*)-1-Methoxy-4-(3-(2-((4-methoxybenzyl)oxy)ethyl)pent-4-en-1-yn-1-yl)benzene

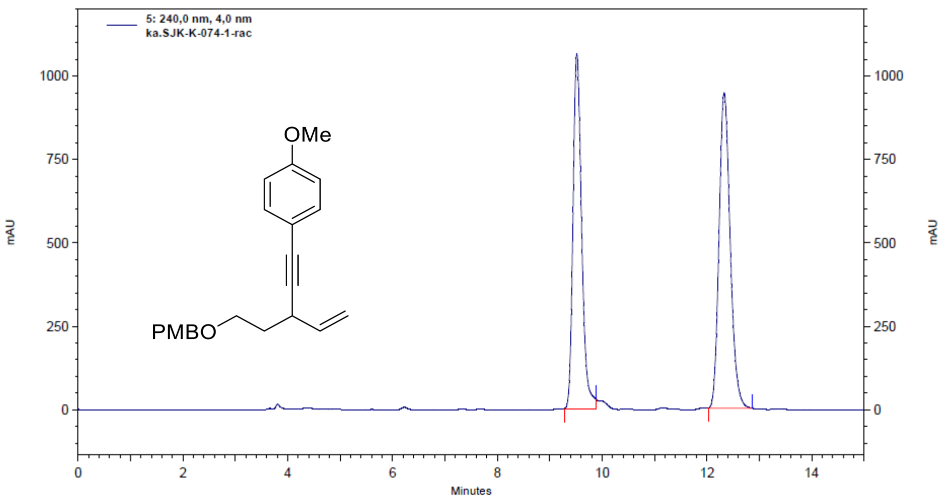

Peak Index	Time / min	Area / %
1	9.5	46.477
2	12.3	53.523

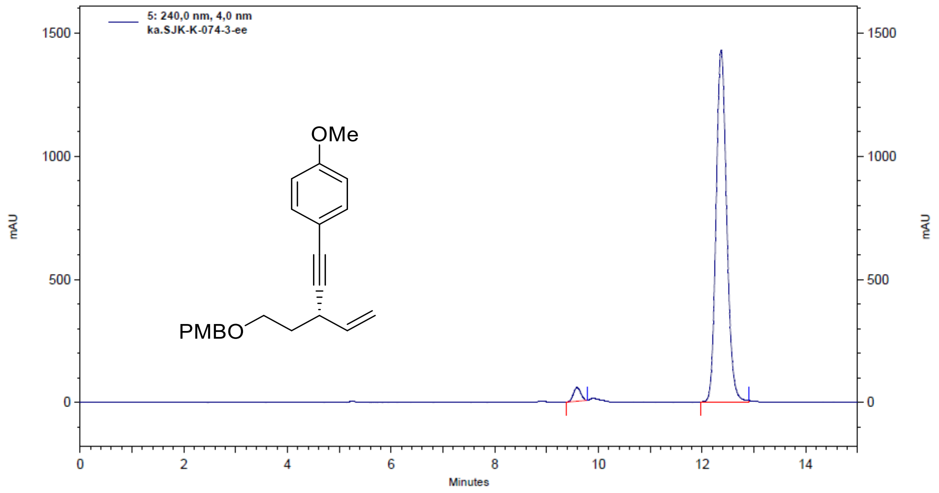

Peak Index	Time / min	Area / %
1	9.6	2.635
2	12.4	97.365

(*S*)-2-(3-(2-((4-Methoxybenzyl)oxy)ethyl)pent-4-en-1-yn-1-yl)phenol

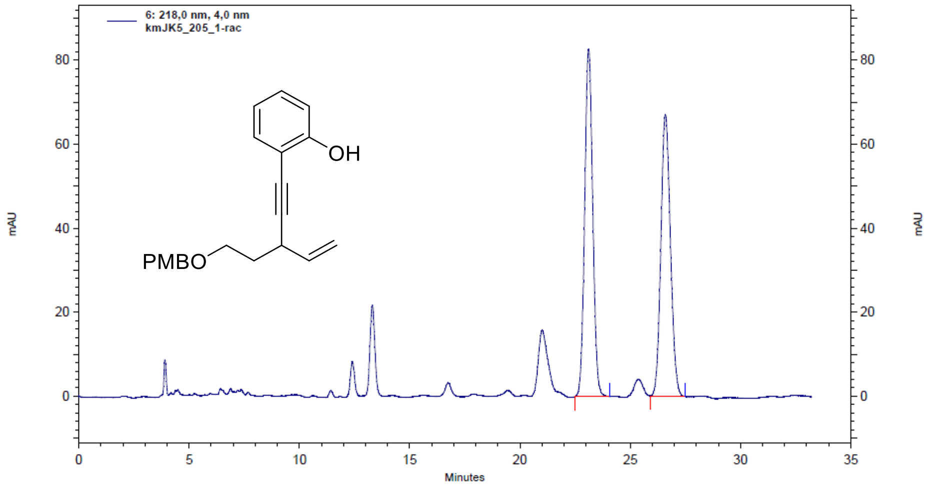

Peak Index	Time / min	Area / %
1	23.1	50.955
2	26.6	49.045

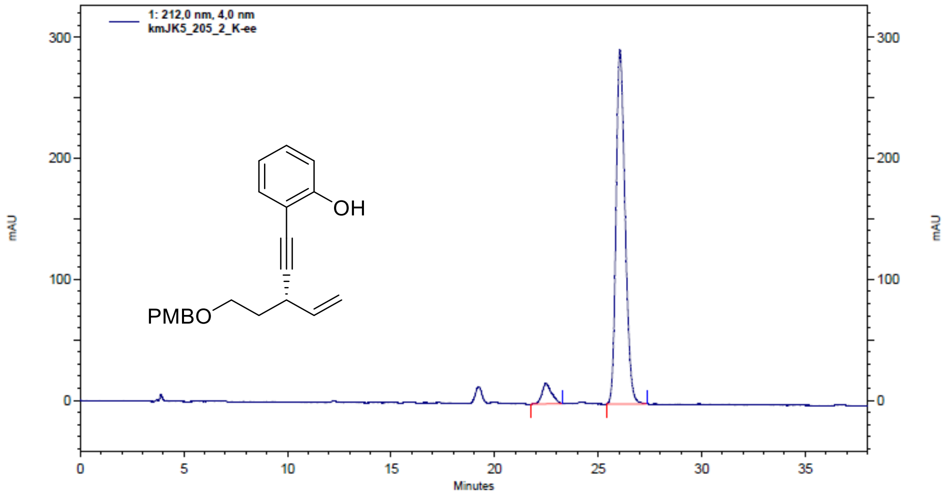

Peak Index	Time / min	Area / %
1	22.5	5.823
2	26.1	94.177

(*S*)-2-(3-(2-((4-Methoxybenzyl)oxy)ethyl)pent-4-en-1-yn-1-yl)aniline

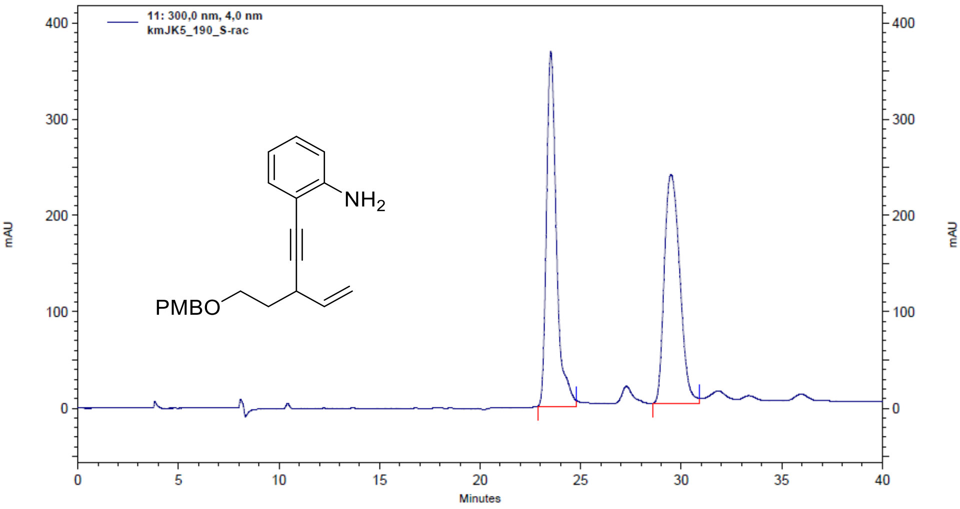

Peak Index	Time / min	Area / %
1	23.5	49.749
2	29.5	50.251

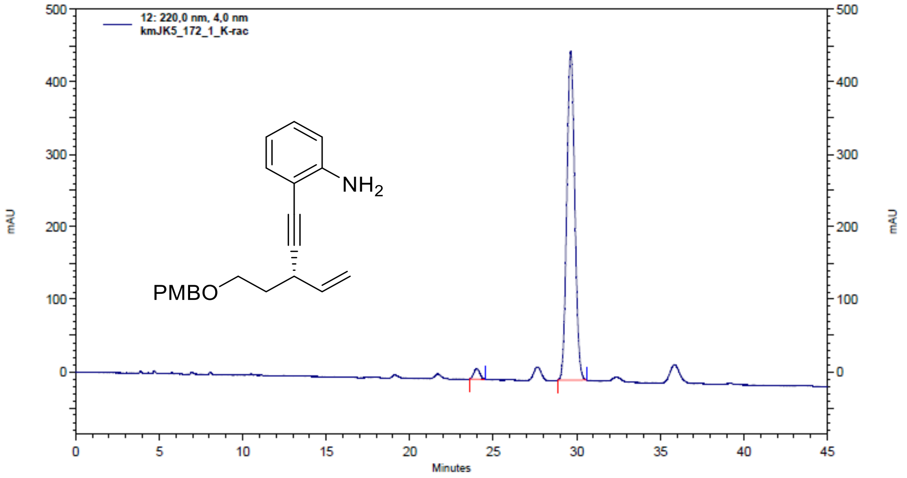

Peak Index	Time / min	Area / %
1	24.0	2.378
2	29.6	97.621

(*S*)-*N*-(2-(3-(2-((4-Methoxybenzyl)oxy)ethyl)pent-4-en-1-yn-1-yl)phenyl)-4-methylbenzenesulfonamide

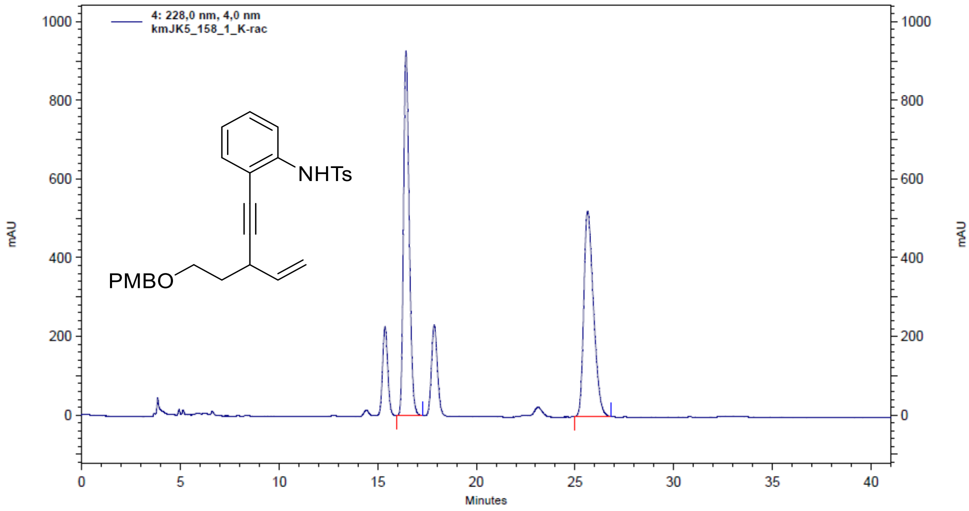

Peak Index	Time / min	Area / %
1	16.4	51.241
2	25.6	48.759

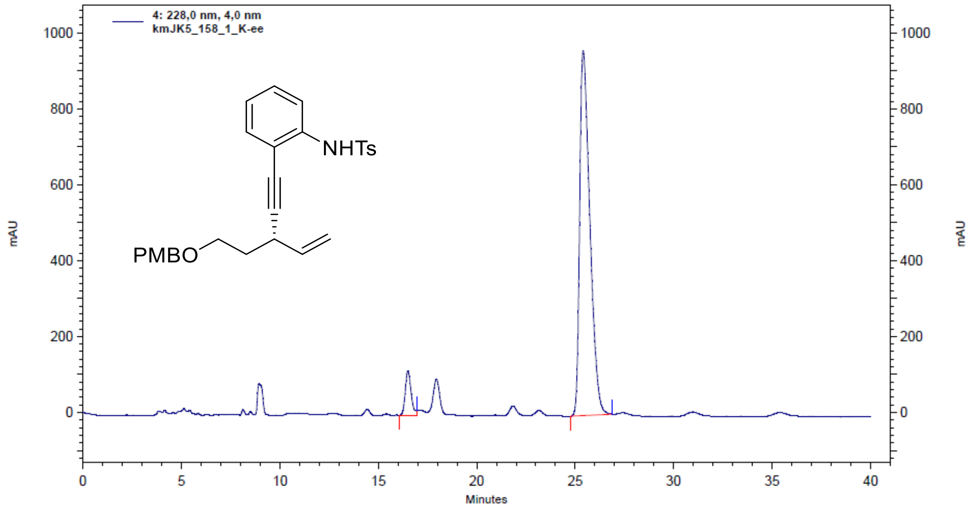

Peak Index	Time / min	Area / %
1	16.5	6.466
2	25.4	93.534

(*S*)-3-(3-(2-((4-Methoxybenzyl)oxy)ethyl)pent-4-en-1-yn-1-yl)aniline

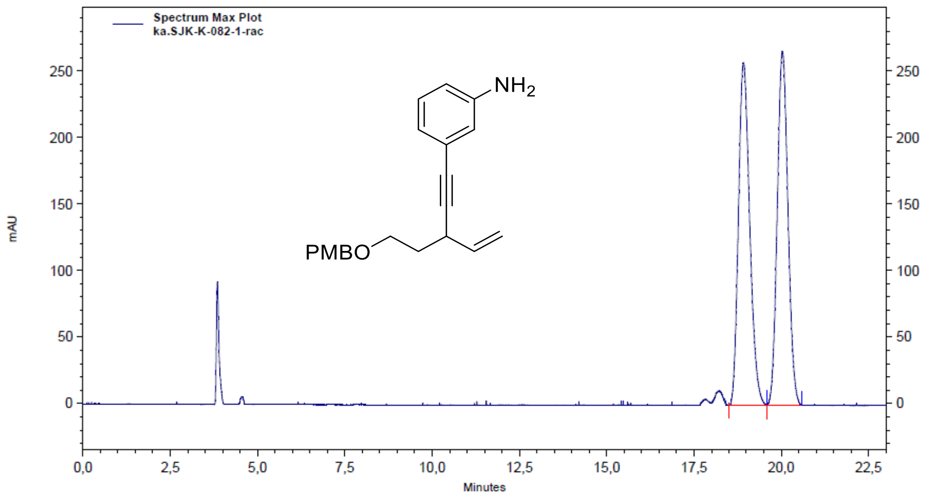

Peak Index	Time / min	Area / %
1	18.9	50.422
2	20.0	49.578

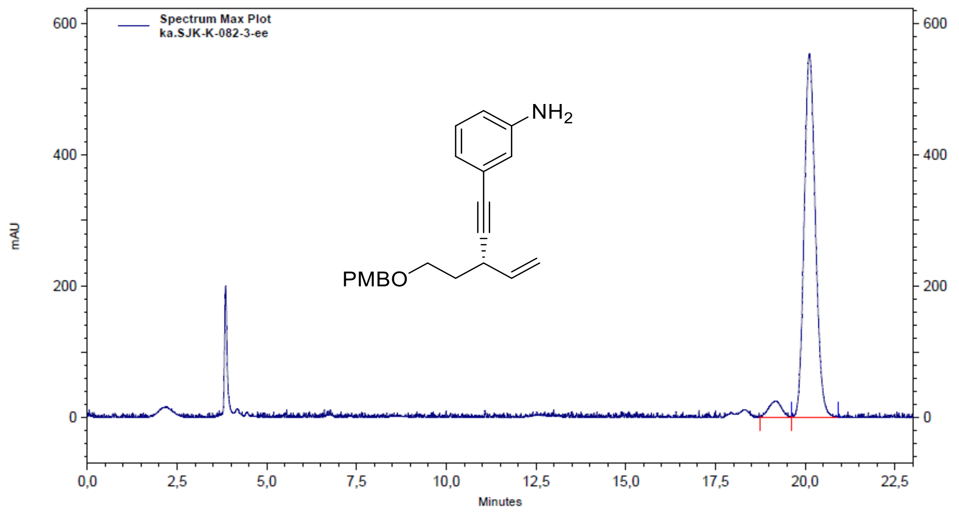

Peak Index	Time / min	Area / %
1	19.2	4.715
2	20.1	95.285

(*S*)-2-(3-(2-((4-Methoxybenzyl)oxy)ethyl)pent-4-en-1-yn-1-yl)thiophene

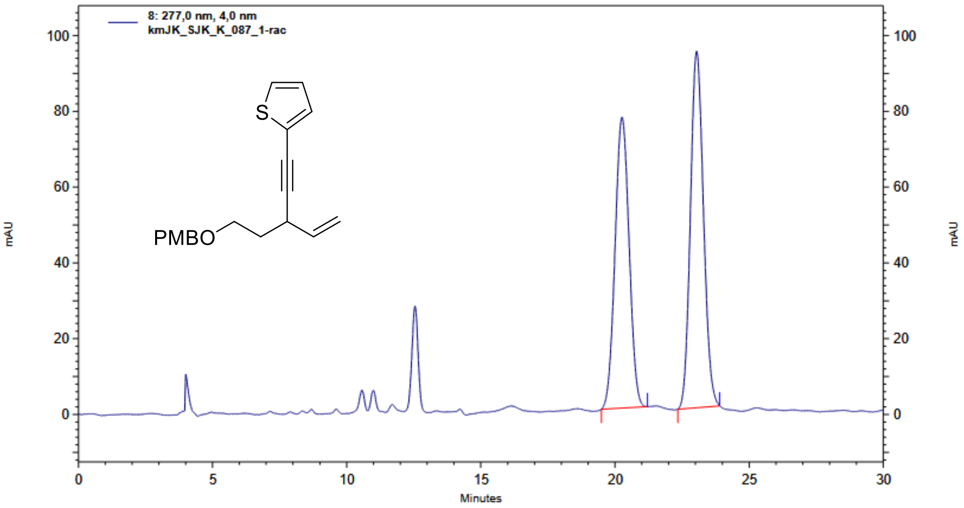

Peak Index	Time / min	Area / %
1	20.3	46.798
2	23.0	53.202

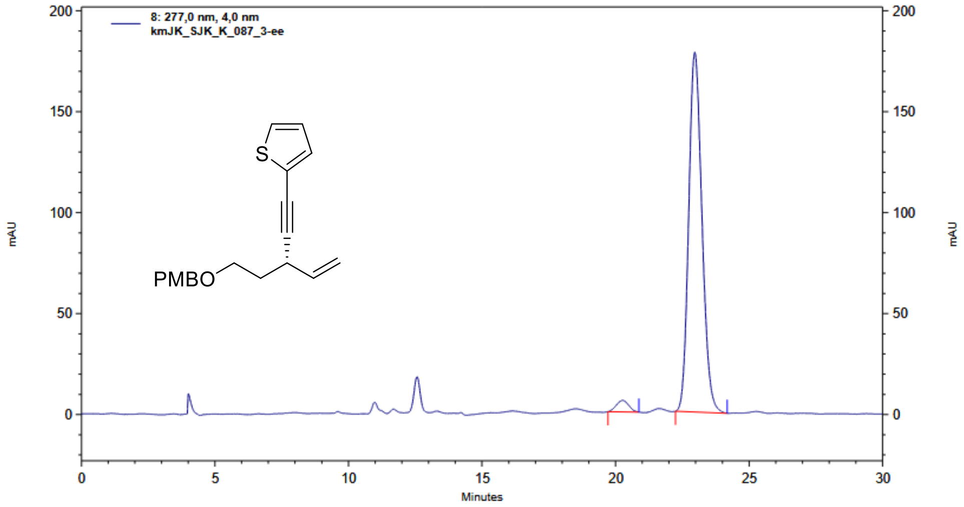

Peak Index	Time / min	Area / %
1	20.3	2.935
2	23.0	97.065

(*S*)-1-(((3-(Cyclopentylethynyl)pent-4-en-1-yl)oxy)methyl)-4-methoxybenzene

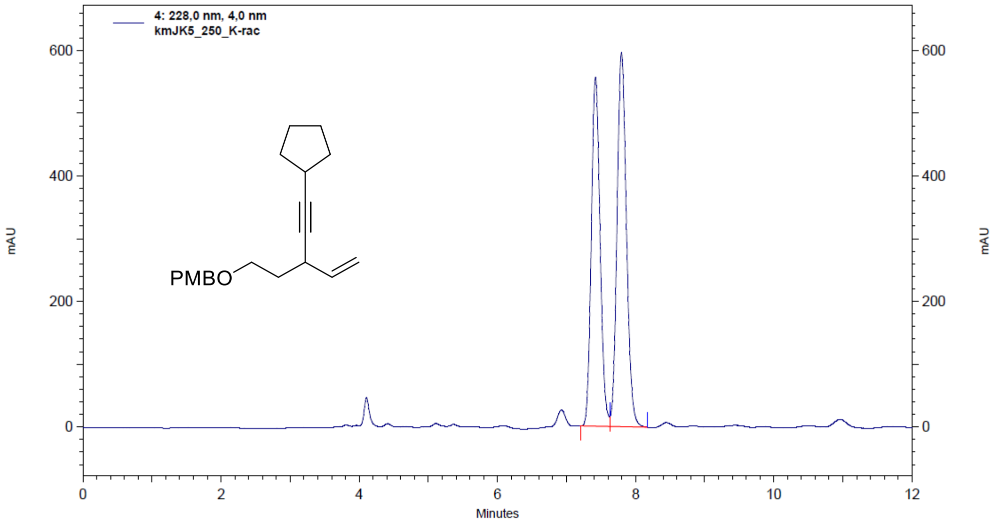

Peak Index	Time / min	Area / %
1	7.4	46.532
2	7.8	53.468

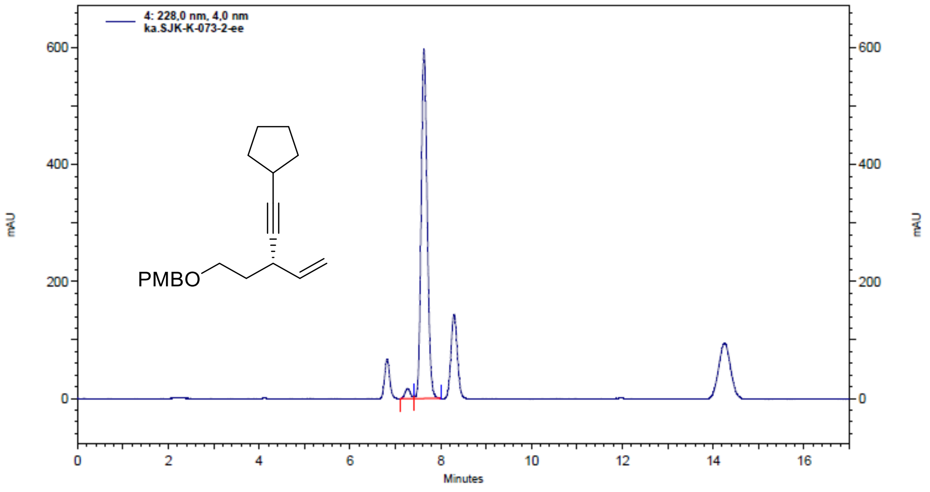

Peak Index	Time / min	Area / %
1	7.3	2.507
2	7.6	97.493

(*S*)-1-(((3-(Cyclopropylethynyl)pent-4-en-1-yl)oxy)methyl)-4-methoxybenzene

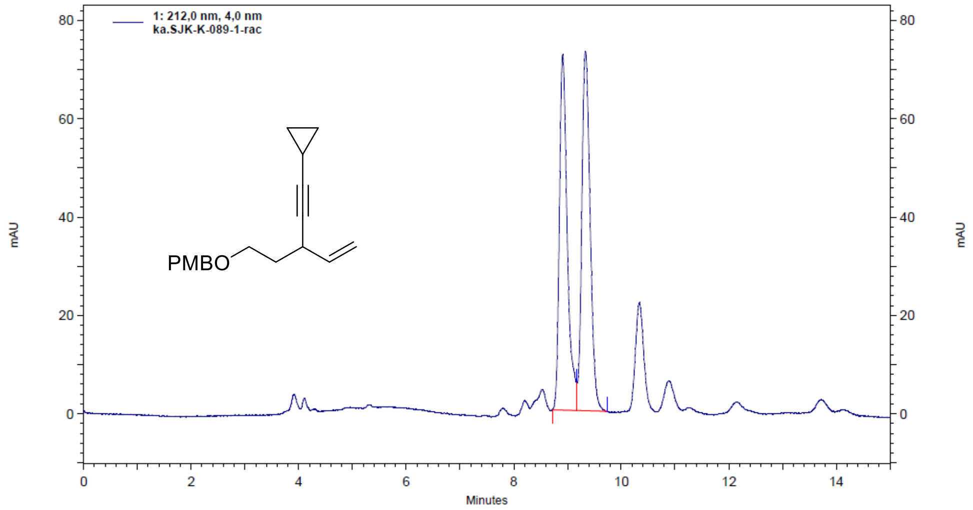

Peak Index	Time / min	Area / %
1	8.9	50.373
2	9.3	49.627

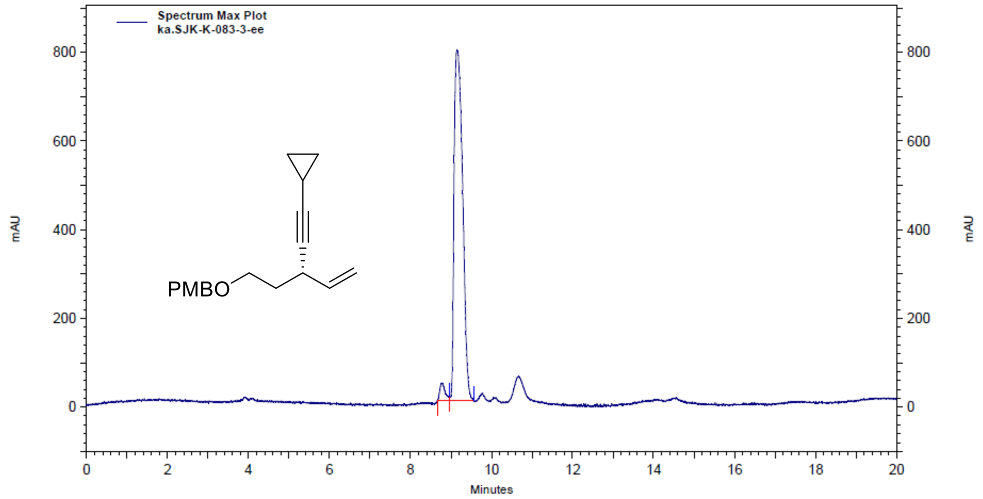

Peak Index	Time / min	Area / %
1	8.8	3.134
2	9.2	96.866

(*S*,*E*)-1-Methoxy-4-(((9-phenyl-3-vinylnon-6-en-4-yn-1-yl)oxy)methyl)benzene

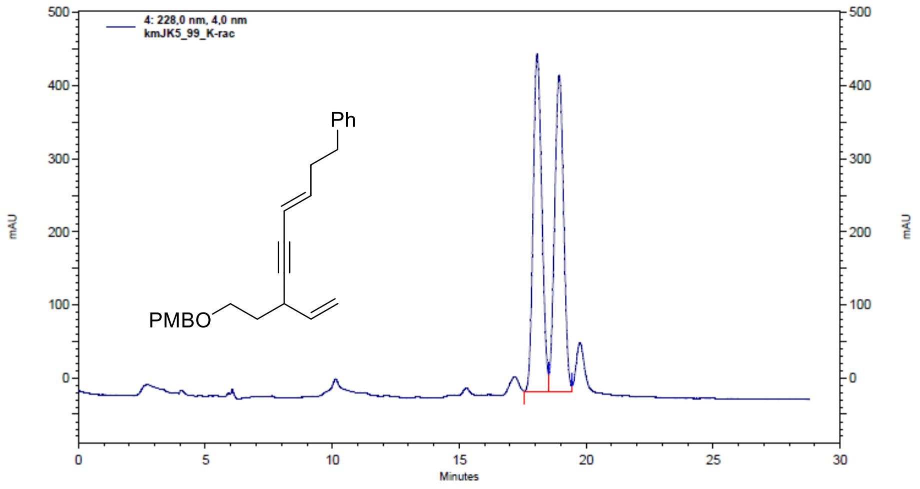

Peak Index	Time / min	Area / %
1	18.1	50.506
2	18.9	49.494

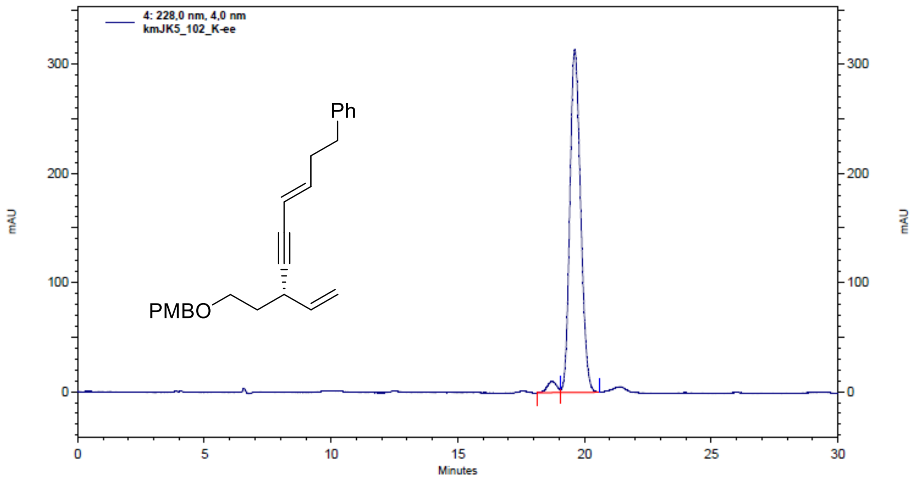

Peak Index	Time / min	Area / %
1	18.7	2.858
2	19.6	97.142

***Tert*-butyl (*S*)-(4-(2-((4-methoxybenzyl)oxy)ethyl)hex-5-en-2-yn-1-yl)carbamate**

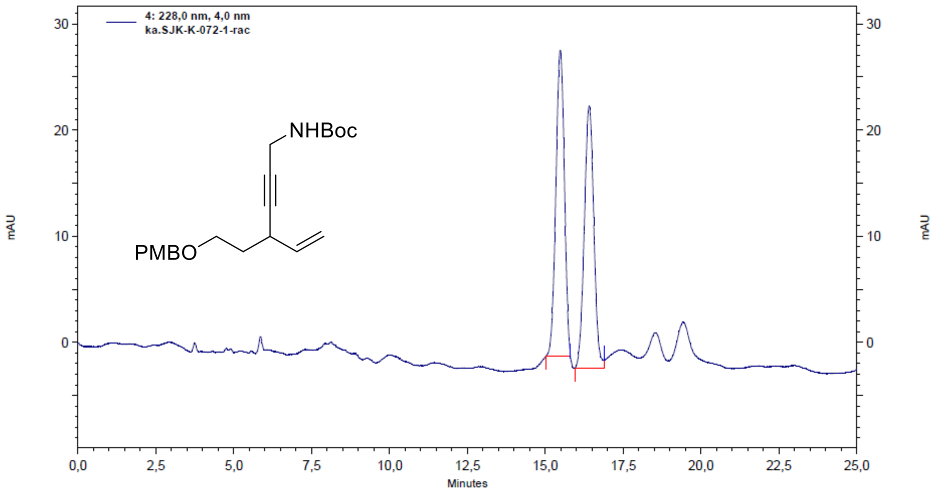

Peak Index	Time / min	Area / %
1	15.5	51.052
2	16.4	48.948

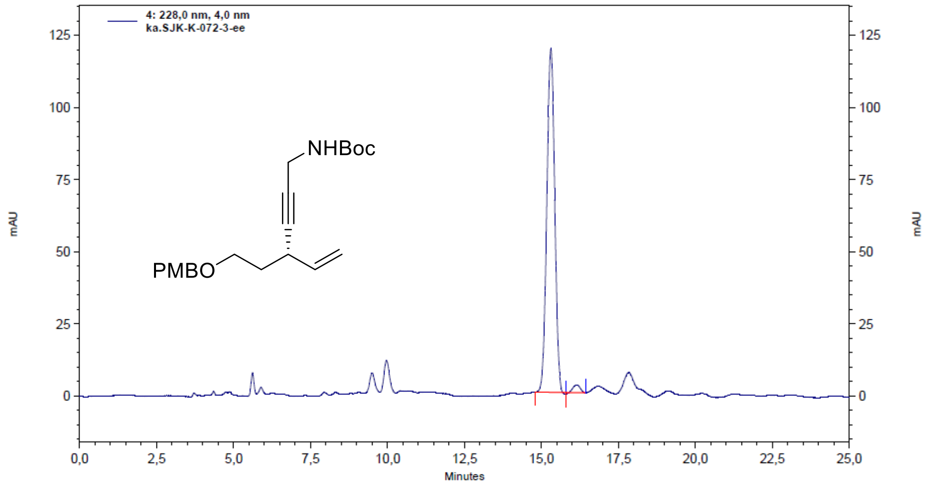

Peak Index	Time / min	Area / %
1	15.3	98.032
2	16.2	1.968

(*S*)-4,4'-(((5-Vinylhept-3-yne-1,7-diyl)bis(oxy))bis(methylene))bis(methoxybenzene)

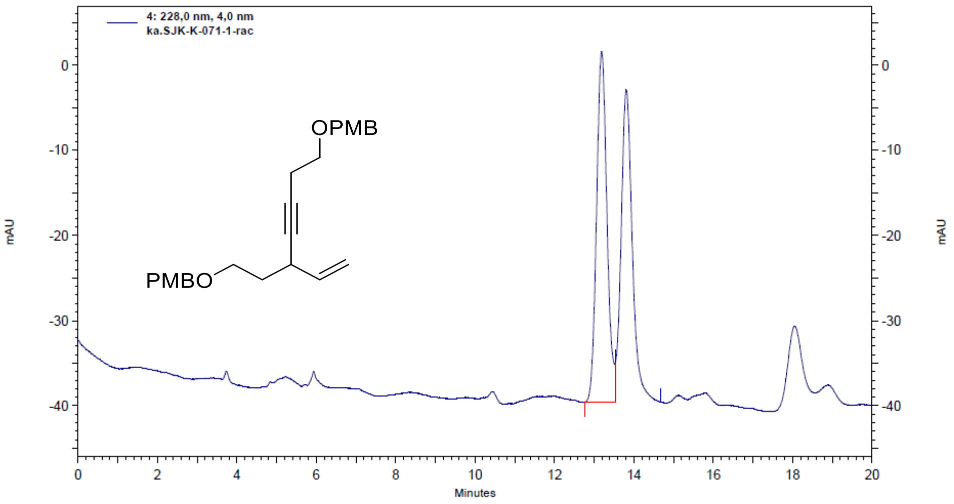

Peak Index	Time / min	Area / %
1	13.2	50.848
2	13.8	49.152

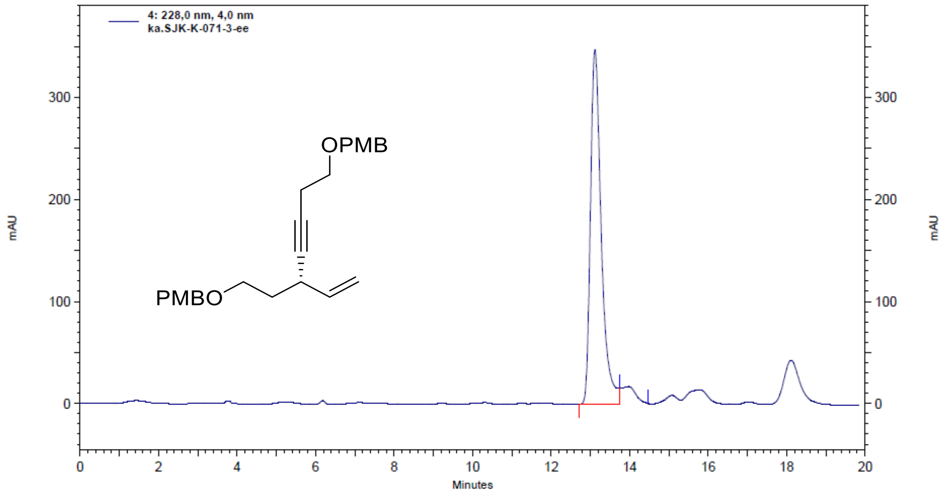

Peak Index	Time / min	Area / %
1	13.1	6.552
2	14.0	93.448

(*S*)-5-(2-((4-Methoxybenzyl)oxy)ethyl)-2-methylhept-6-en-3-yn-2-ol

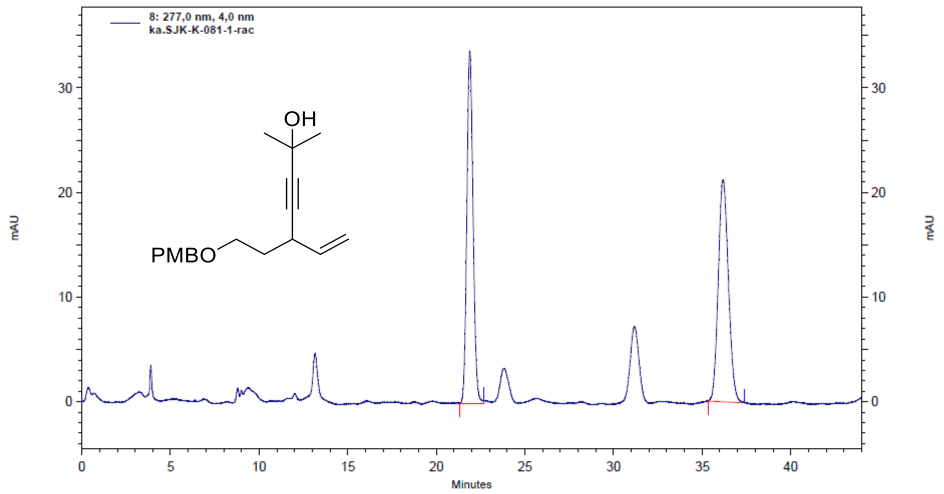

Peak Index	Time / min	Area / %
1	21.9	49.188
2	36.2	50.812

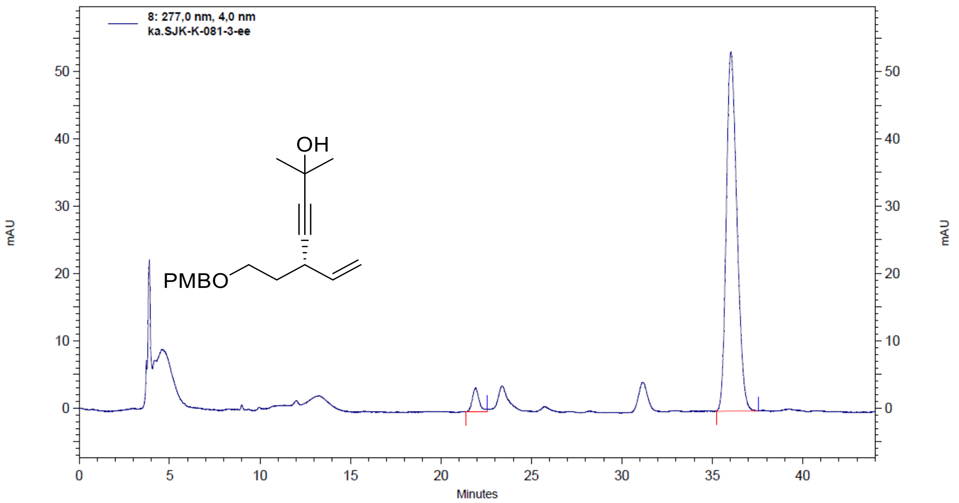

Peak Index	Time / min	Area / %
1	21.9	4.096
2	36.0	95.904

(*S*)-(3-(2-((4-Methoxybenzyl)oxy)ethyl)pent-4-en-1-yn-1-yl)trimethylsilane

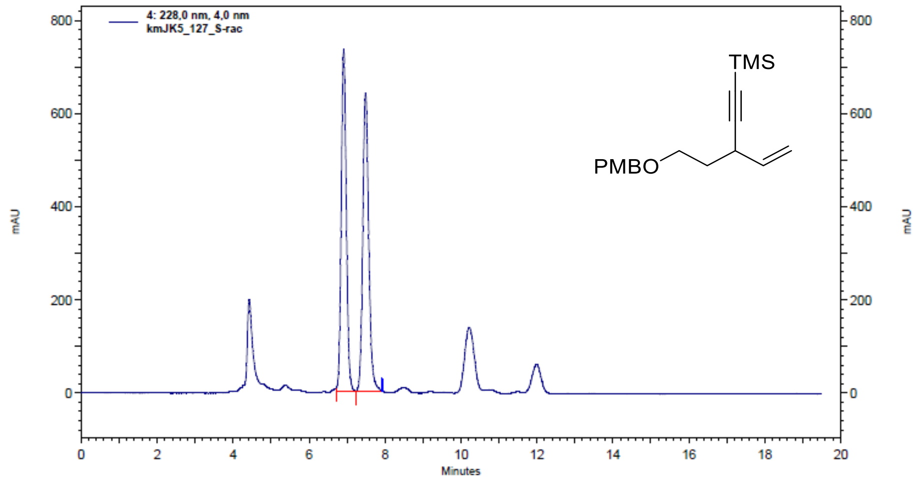

Peak Index	Time / min	Area / %
1	6.9	49.118
2	7.5	50.882

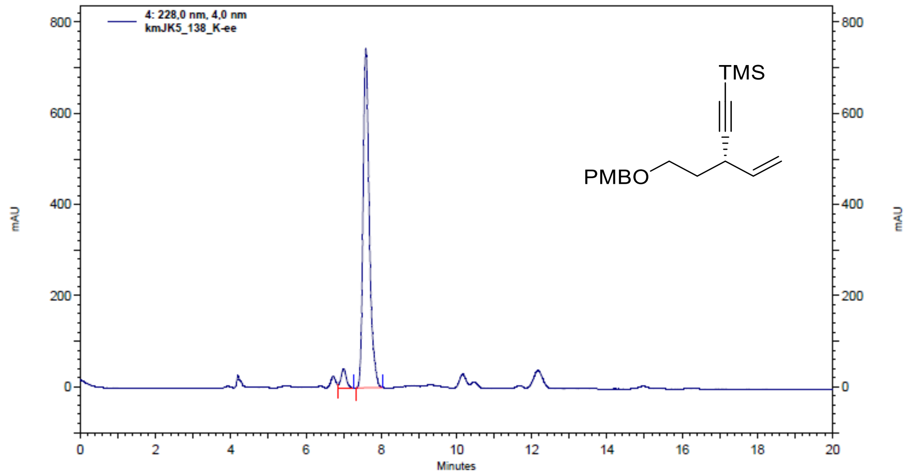

Peak Index	Time / min	Area / %
1	7.0	4.561
2	7.6	95.439

(*S*)-*tert*-Butyldiphenyl((3-(phenylethynyl)pent-4-en-1-yl)oxy)silane

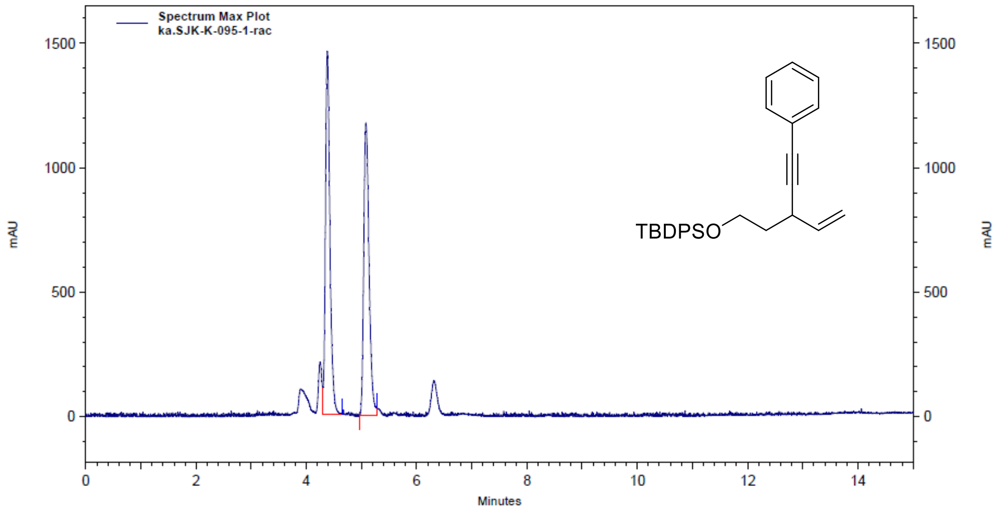

Peak Index	Time / min	Area / %
1	4.4	51.181
2	5.1	48.819

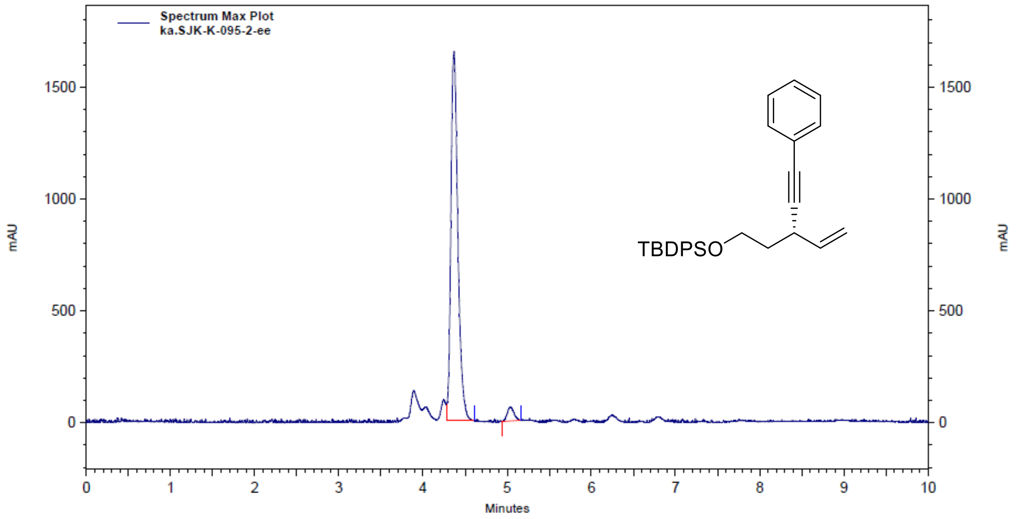

Peak Index	Time / min	Area / %
1	4.4	96.457
2	5.0	3.543

(*S*)-1-Fluoro-4-((3-(phenylethynyl)pent-4-en-1-yl)oxy)benzene

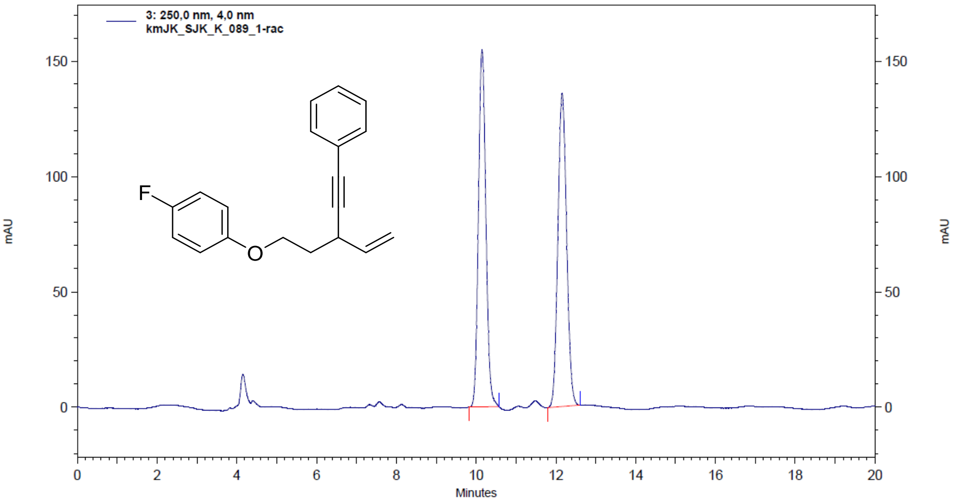

Peak Index	Time / min	Area / %
1	10.1	49.324
2	12.2	50.676

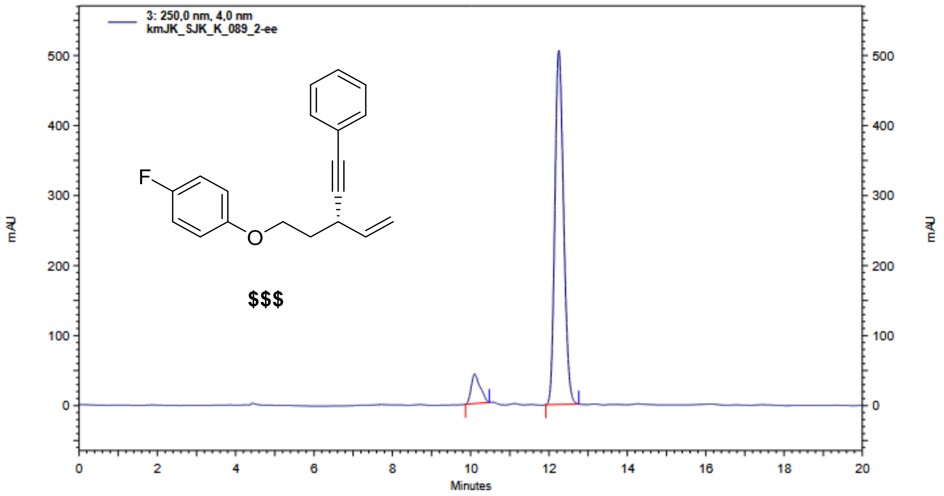

Peak Index	Time / min	Area / %
1	10.1	7.874
2	12.3	92.126

(*S*)-1-Chloro-4-((3-(phenylethynyl)pent-4-en-1-yl)oxy)benzene

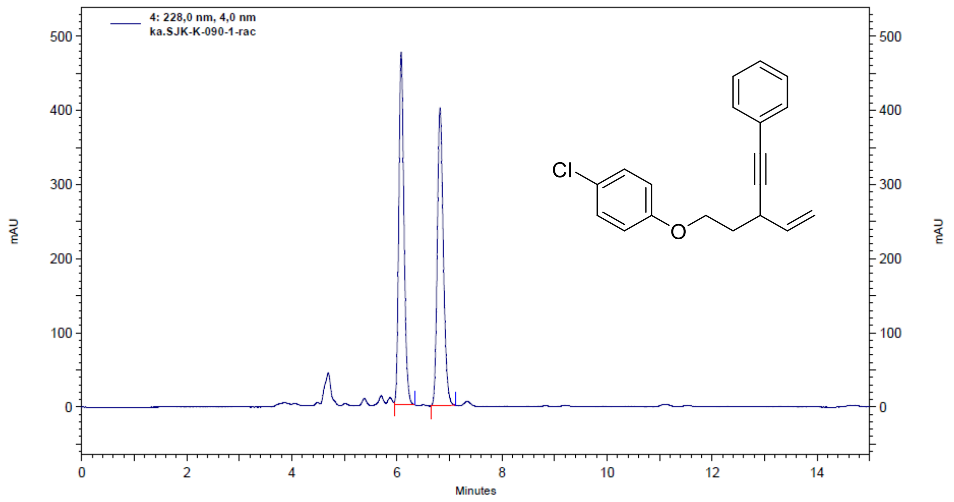

Peak Index	Time / min	Area / %
1	6.1	50.377
2	6.8	49.623

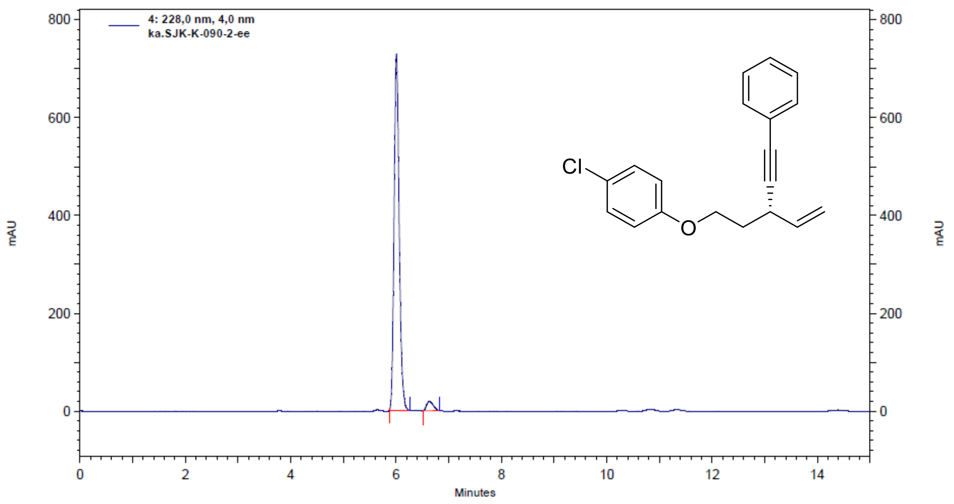

Peak Index	Time / min	Area / %
1	6.0	96.713
2	6.6	3.287

(*S*)-1-Bromo-4-((3-(phenylethynyl)pent-4-en-1-yl)oxy)benzene

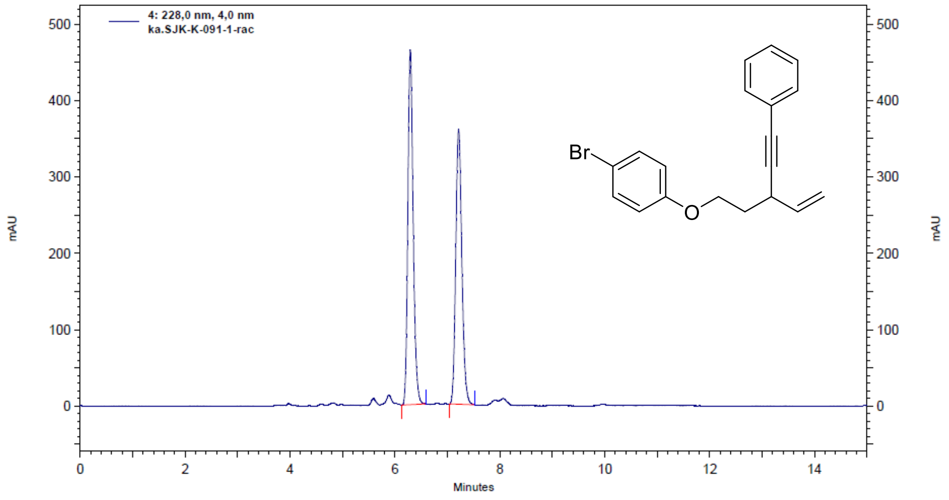

Peak Index	Time / min	Area / %
1	6.3	53.061
2	7.2	46.939

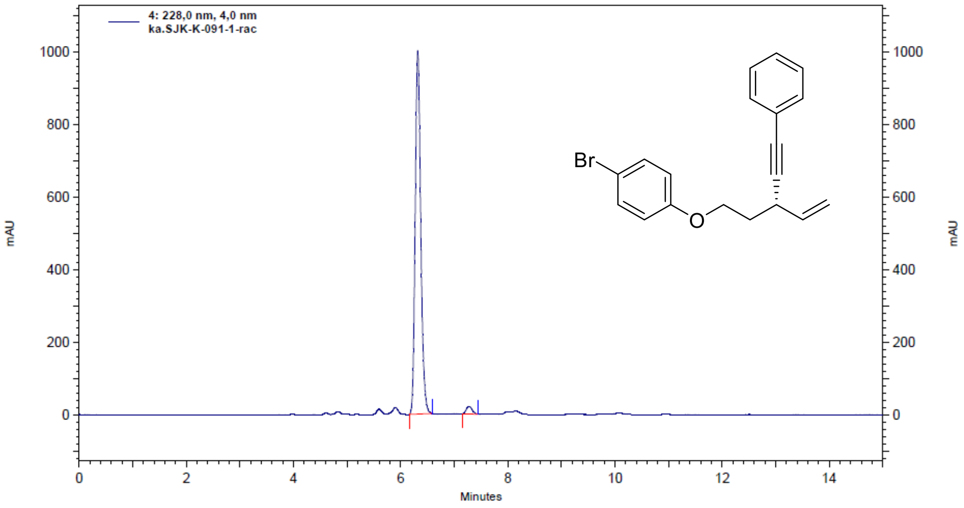

Peak Index	Time / min	Area / %
1	6.3	97.768
2	7.3	2.232

(*S*)-2-((3-(Phenylethynyl)pent-4-en-1-yl)oxy)benzonitrile

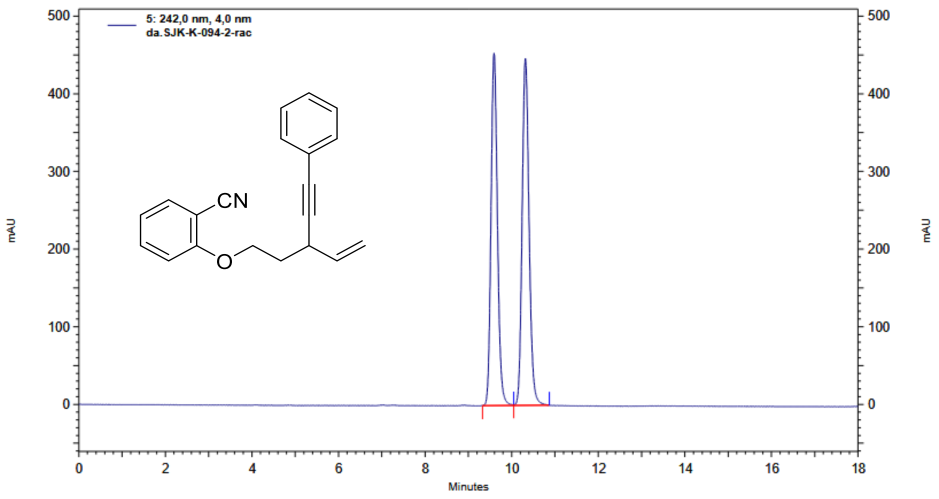

Peak Index	Time / min	Area / %
1	9.6	48.493
2	10.3	51.507

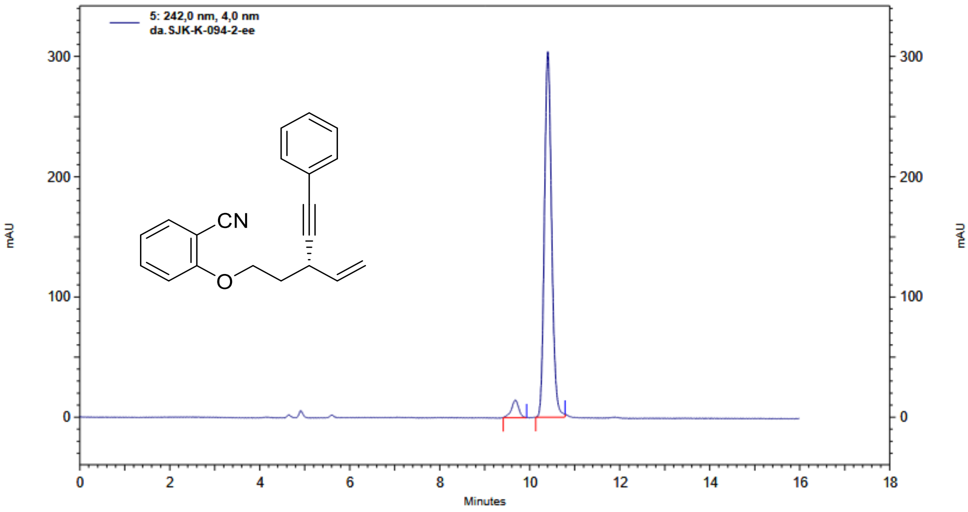

Peak Index	Time / min	Area / %
1	9.7	4.656
2	10.4	95.344

(*S*)-2-(4-(Phenylethynyl)hex-5-en-1-yl)isoindoline-1,3-dione

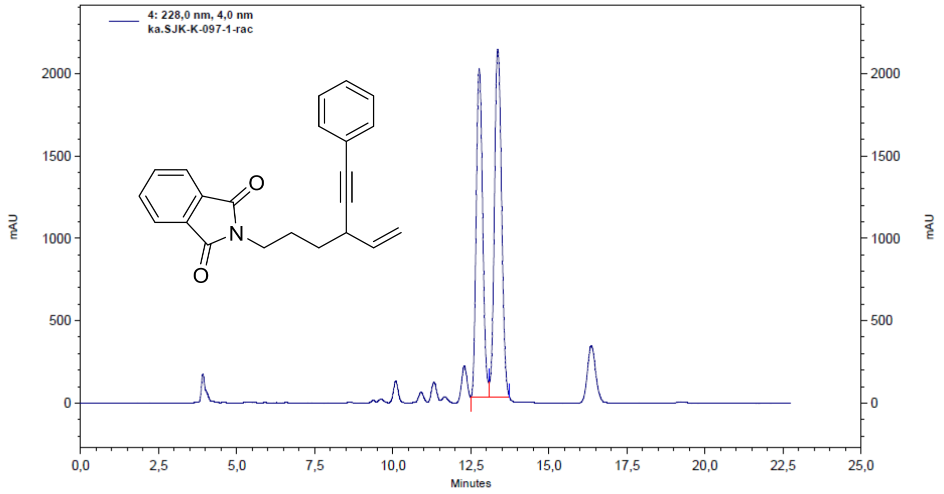

Peak Index	Time / min	Area / %
1	12.8	46.034
2	13.4	53.966

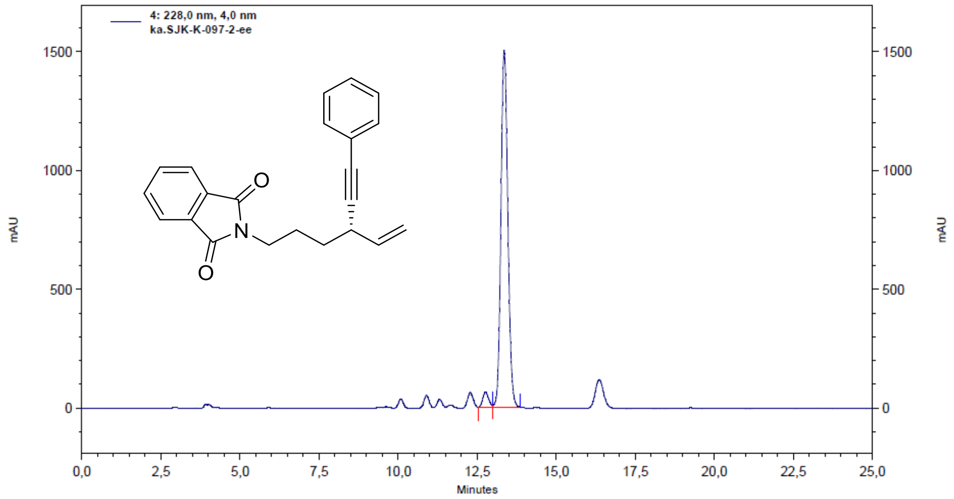

Peak Index	Time / min	Area / %
1	12.8	3.590
2	13.4	96.410

(*S*)-5-Methyl-5-(phenylethynyl)hept-6-en-1-ol

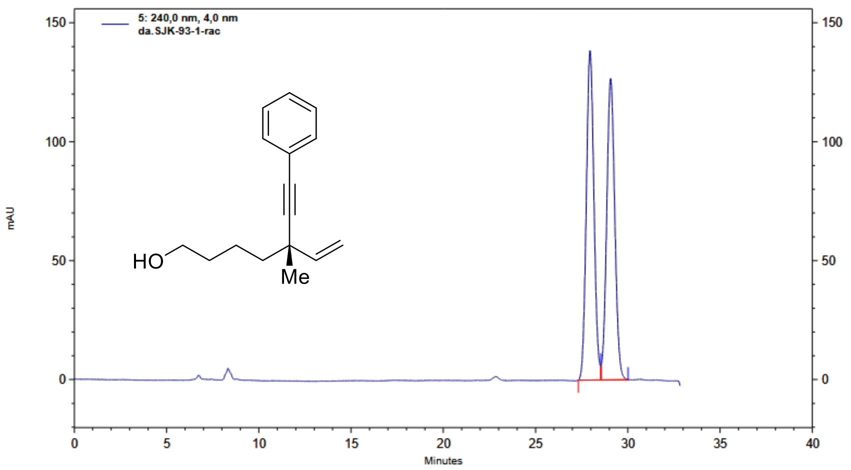

Peak Index	Time / min	Area / %
1	28.0	50.756
2	29.1	49.244

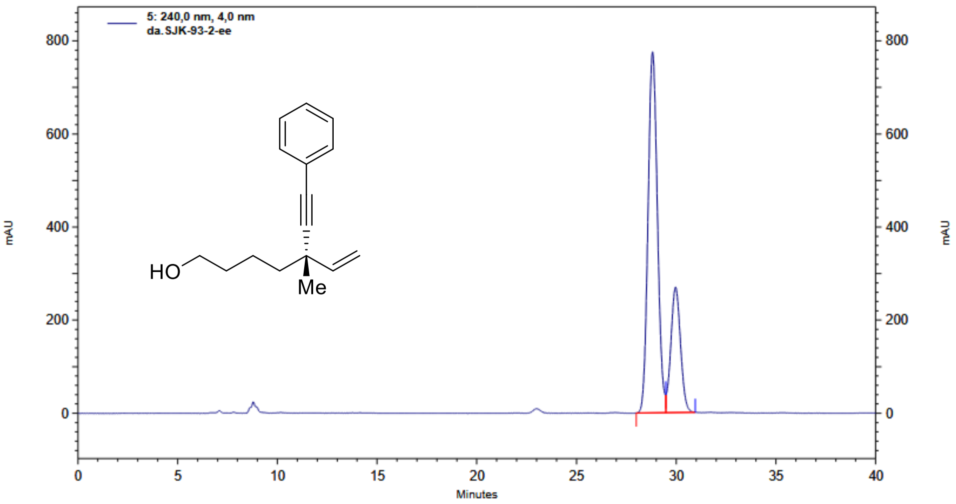

Peak Index	Time / min	Area / %
1	28.8	73.700
2	30.0	26.300

(*S*)-3-(Phenylethynyl)pent-4-en-1-yl 4-(*N*,*N*-dipropylsulfamoyl)benzoate

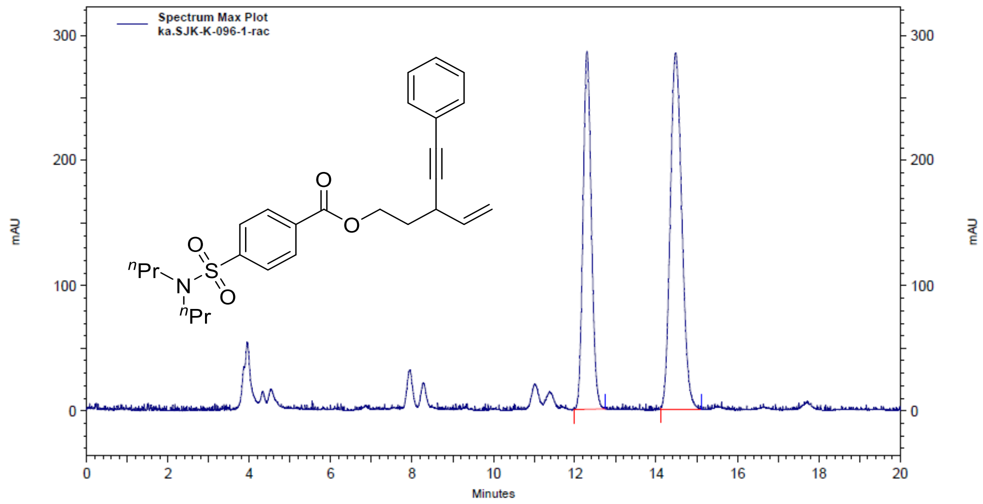

Peak Index	Time / min	Area / %
1	12.3	42.000
2	14.5	58.000

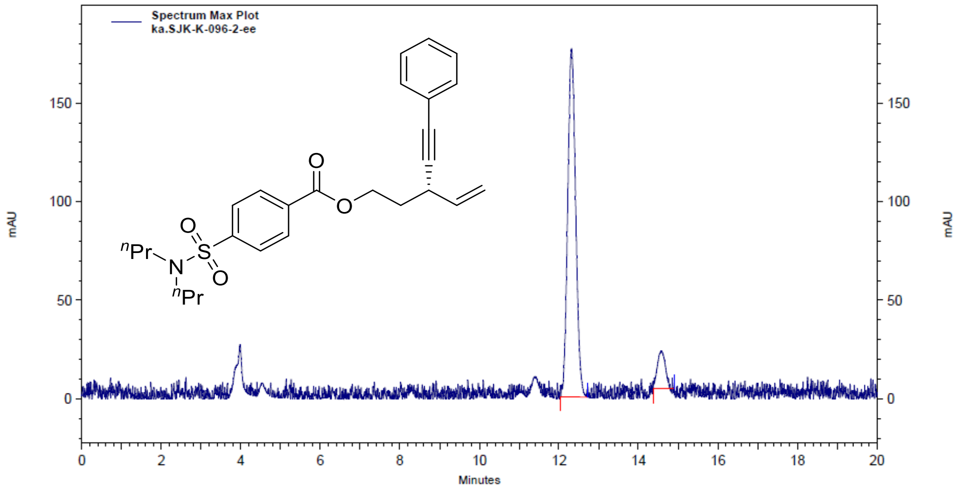

Peak Index	Time / min	Area / %
1	12.3	90.125
2	14.6	9.875

(*S*)-2-(3-(2-(4-Bromophenoxy)ethyl)pent-4-en-1-yn-1-yl)aniline

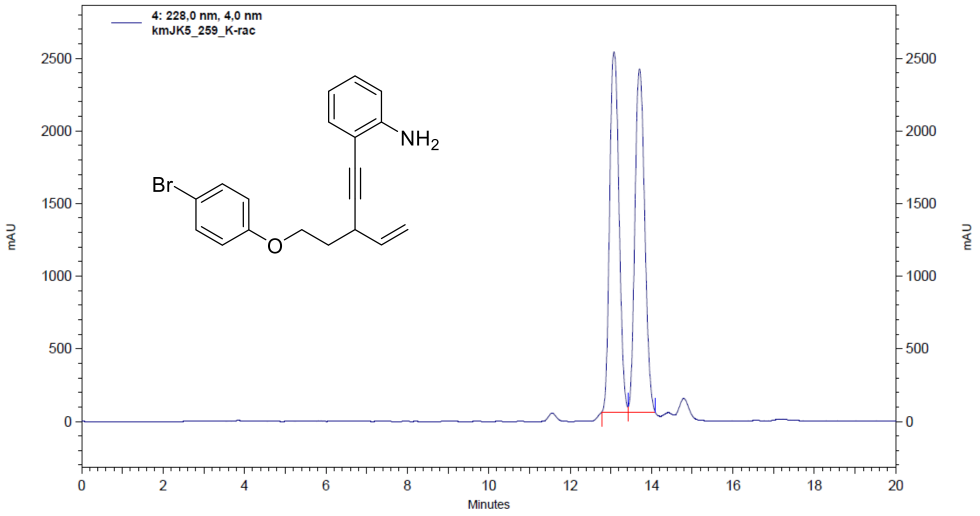

Peak Index	Time / min	Area / %
1	13.1	50.091
2	13.7	49.909

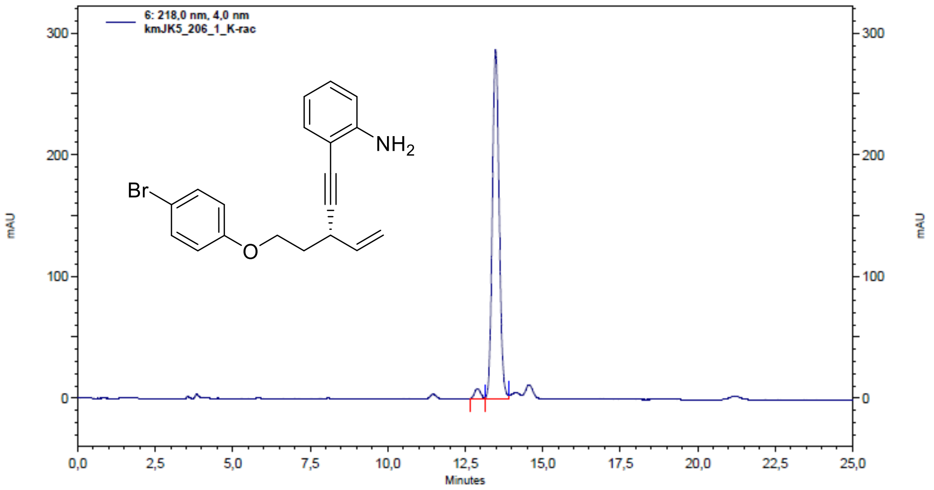

Peak Index	Time / min	Area / %
1	12.9	2.523
2	13.5	97.477

(*S*)-*N*-Benzyl-2-(3-phenethylpent-4-en-1-yn-1-yl)aniline

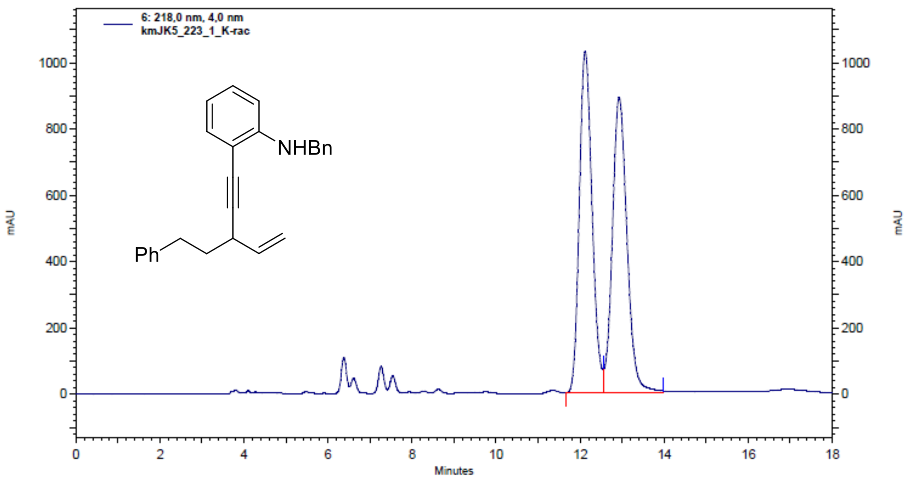

Peak Index	Time / min	Area / %
1	12.1	50.153
2	12.9	49.847

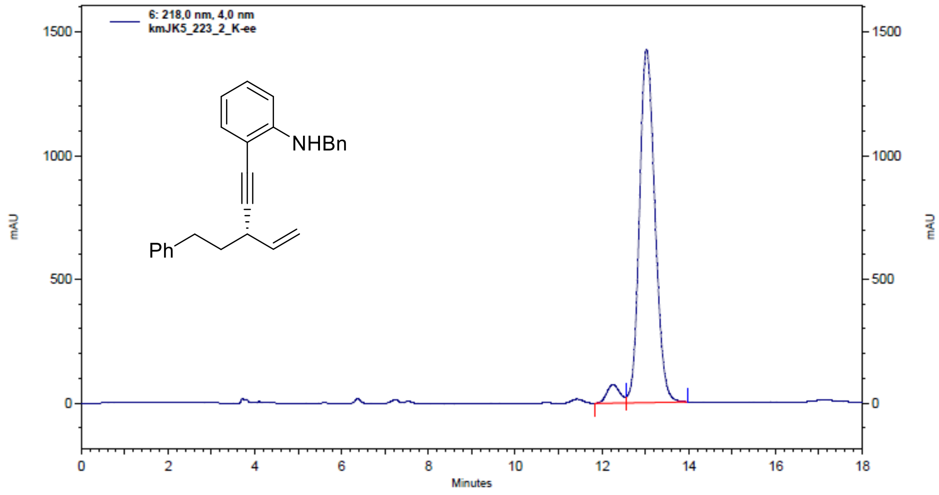

Peak Index	Time / min	Area / %
1	12.3	4.271
2	13.0	95.729

(*R*)-1-Methoxy-4-(((3-(phenylethynyl)pent-4-en-1-yl)oxy)methyl)benzene and deuterated (*S*)-1-methoxy-4-(((3-(phenylethynyl)pent-4-en-1-yl)oxy)methyl)benzene

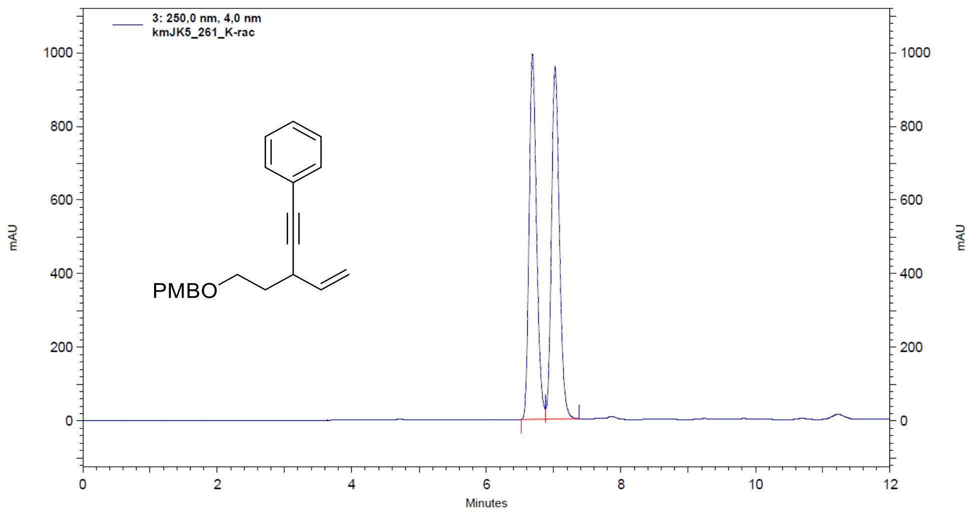

Peak Index	Time / min	Area / %
1	6.7	49.491
2	7.0	50.509

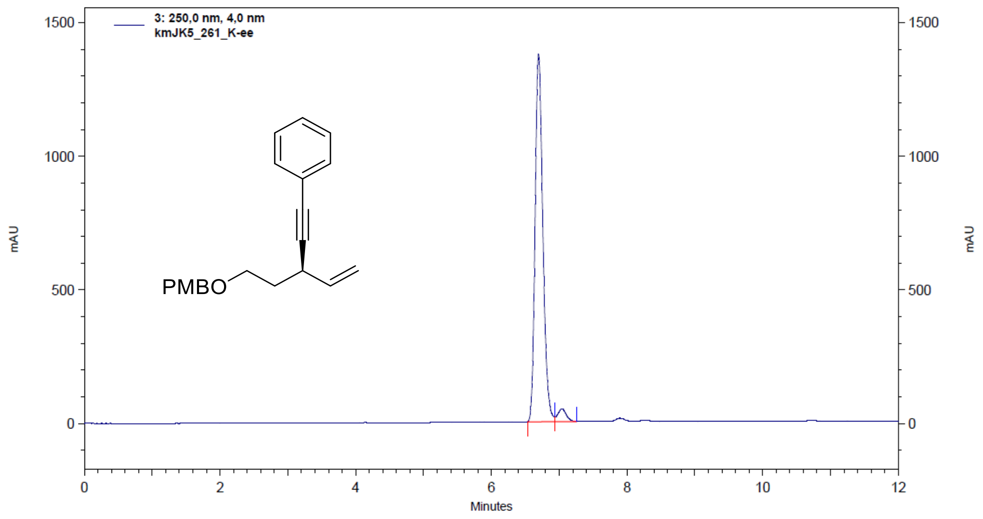

Peak Index	Time / min	Area / %
1	6.7	96.350
2	7.0	3.650

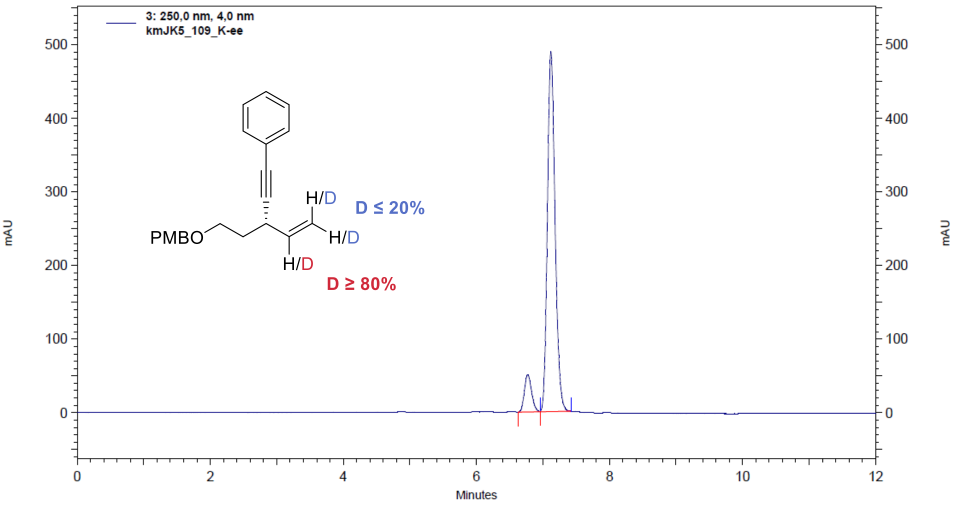

Peak Index	Time / min	Area / %
1	6.8	8.790
2	7.1	91.210

(*S*)-*N*-(2-(3-(2-(4-Bromophenoxy)ethyl)pent-4-en-1-yn-1-yl)phenyl)-3,5-dinitrobenzamide

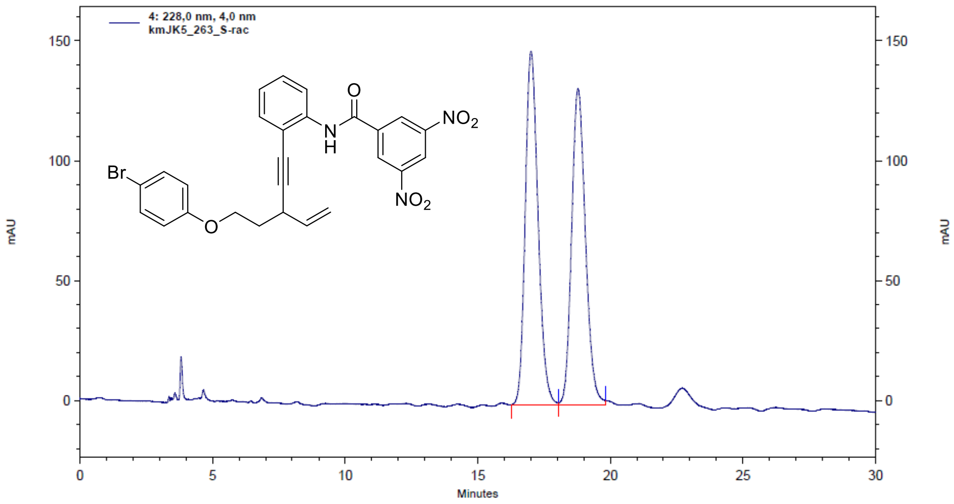

Peak Index	Time / min	Area / %
1	17.0	50.864
2	18.8	49.136

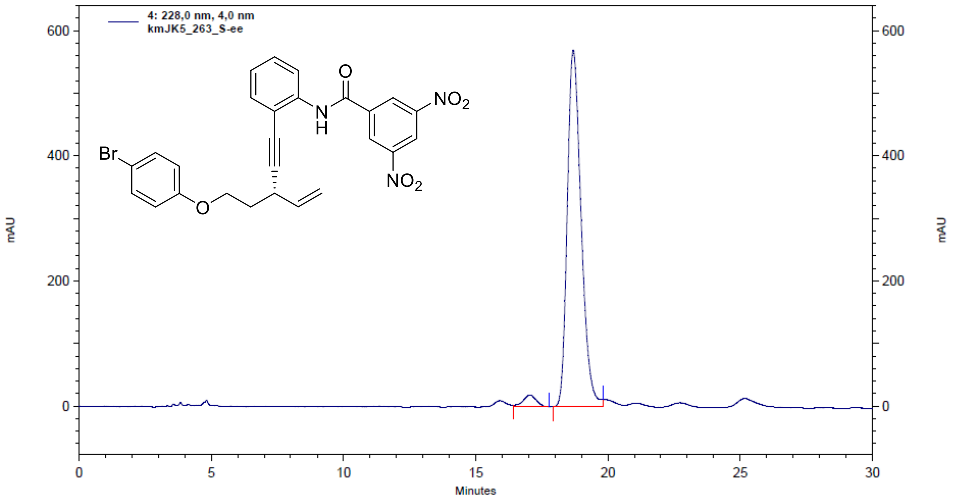

Peak Index	Time / min	Area / %
1	17.1	3.070
2	18.7	96.930

(*S*)-1-(((3-Ethynylpent-4-en-1-yl)oxy)methyl)-4-methoxybenzene

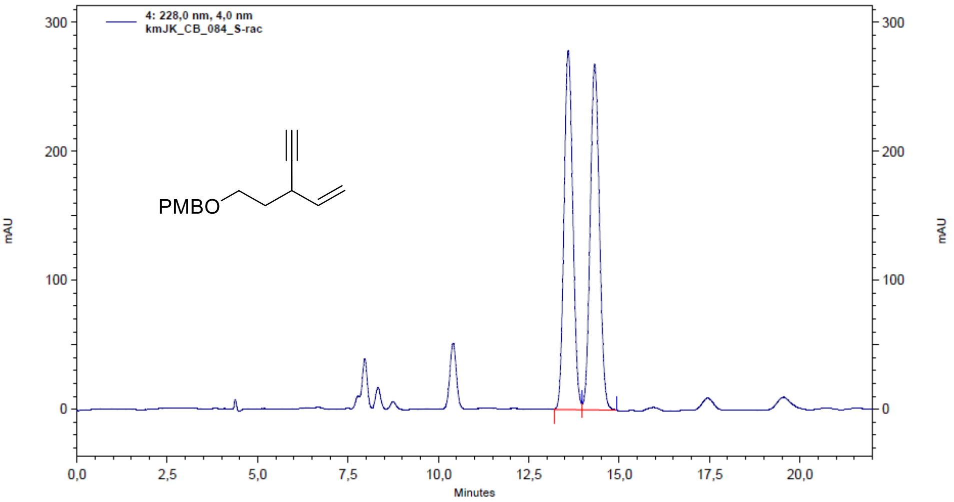

Peak Index	Time / min	Area / %
1	13.6	49.569
2	14.3	50.431

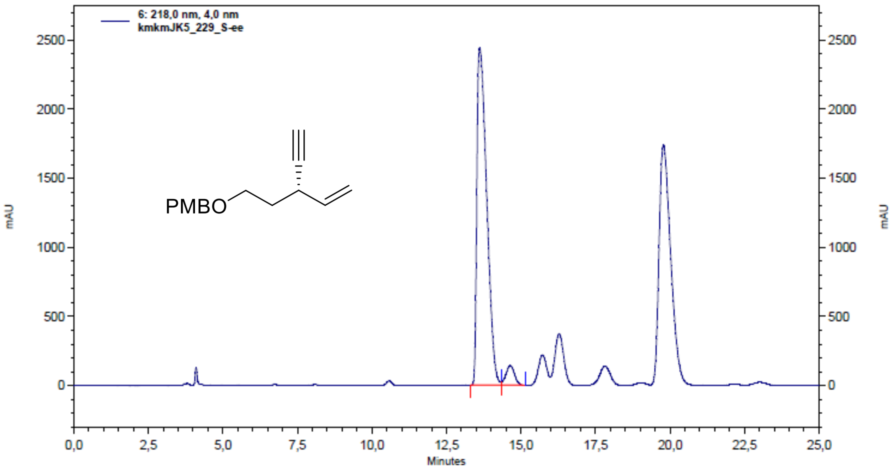

Peak Index	Time / min	Area / %
1	13.6	95.133
2	14.6	4.867

(*S*)-1-Benzyl-4-(5-((4-methoxybenzyl)oxy)pent-1-en-3-yl)-1*H*-1,2,3-triazole

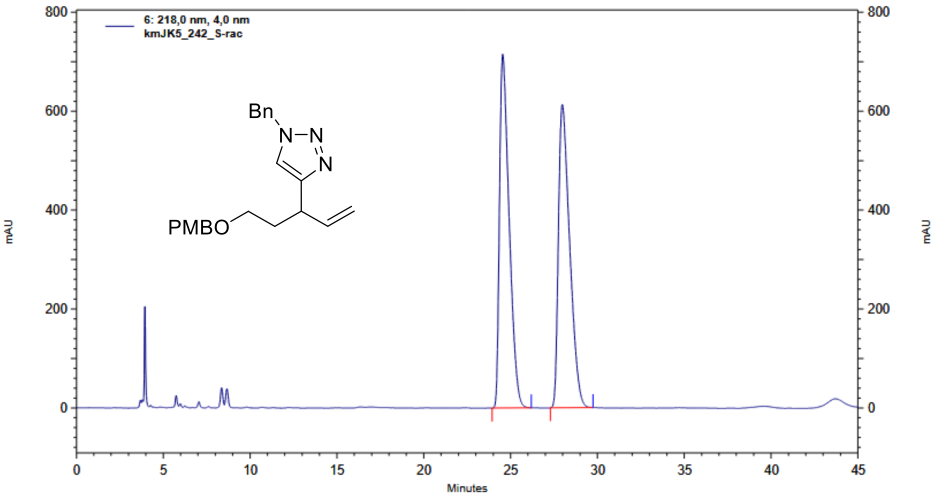

Peak Index	Time / min	Area / %
1	24.6	50.006
2	28.0	49.994

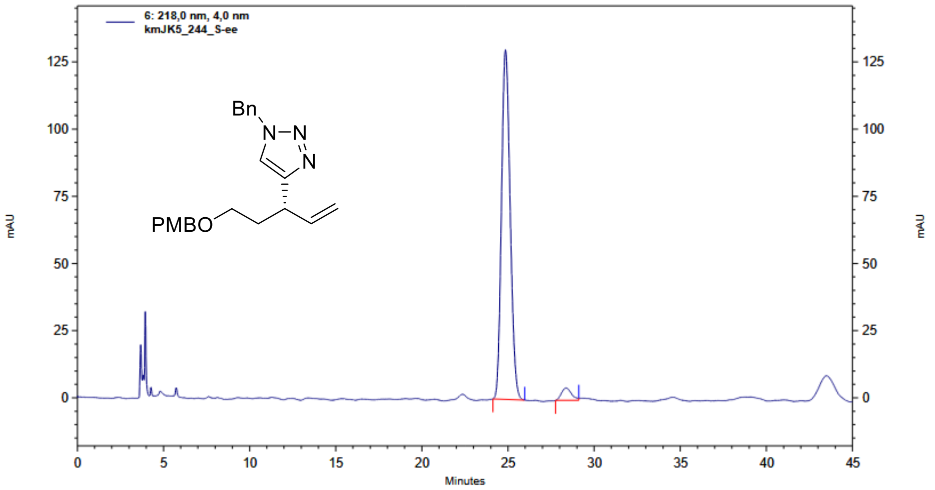

Peak Index	Time / min	Area / %
1	24.855	95.973
2	28.397	4.027

(*S*)-1-Benzyl-2-(5-phenylpent-1-en-3-yl)-1*H*-indole

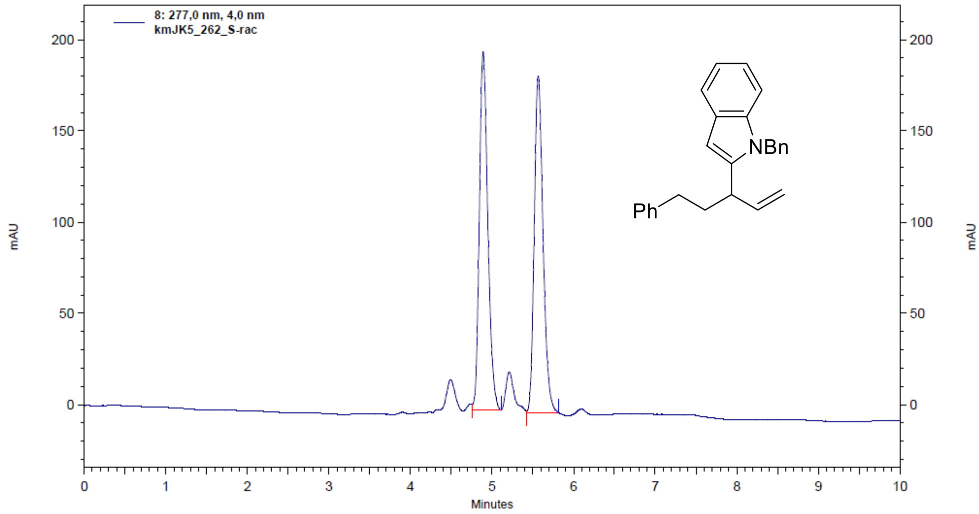

Peak Index	Time / min	Area / %
1	4.9	50.520
2	5.6	49.480

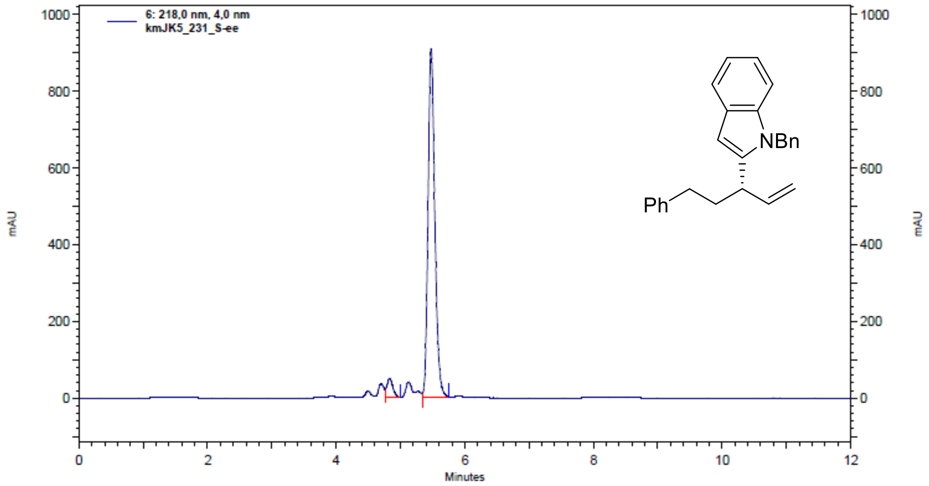

Peak Index	Time / min	Area / %
1	4.8	4.707
2	5.5	95.293

(S)-2-(5-((4-Methoxybenzyl)oxy)pent-1-en-3-yl)benzofuran

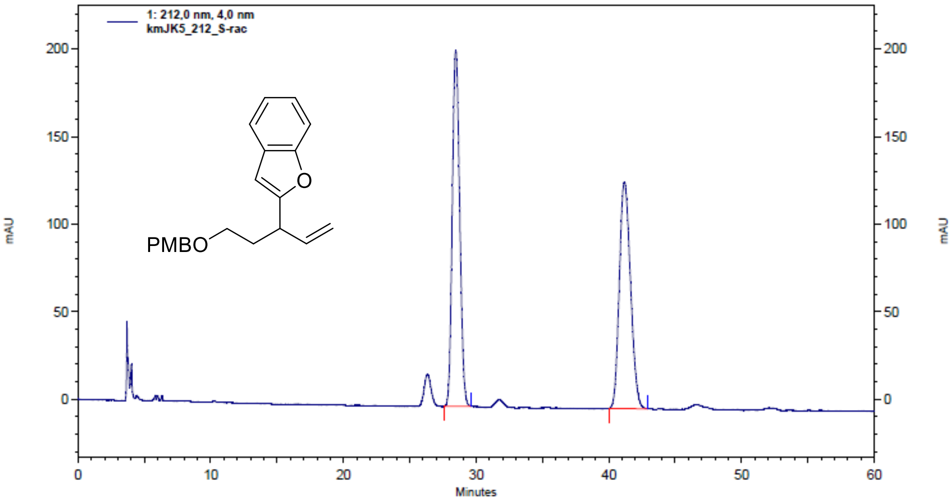

Peak Index	Time / min	Area / %
1	28.5	50.911
2	41.2	49.089

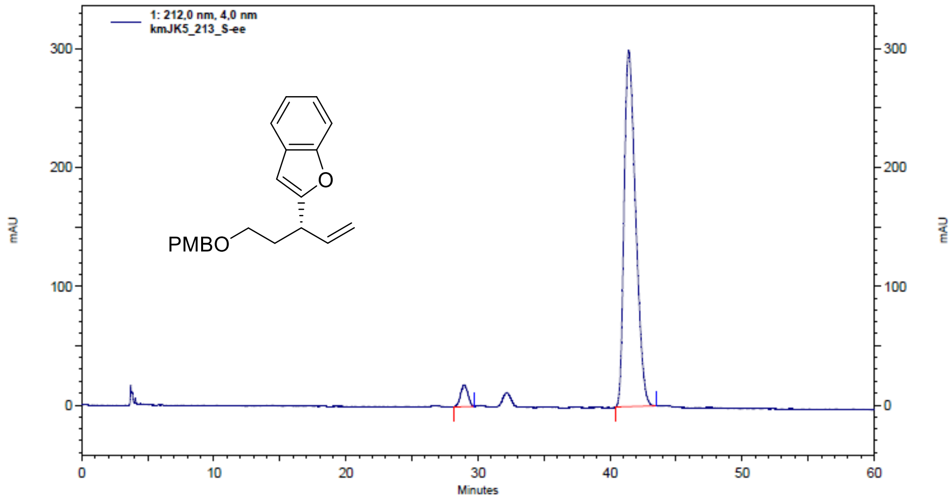

Peak Index	Time / min	Area / %
1	30.0	3.787
2	41.4	96.213

8.4 X-Ray Crystallography Data of 4

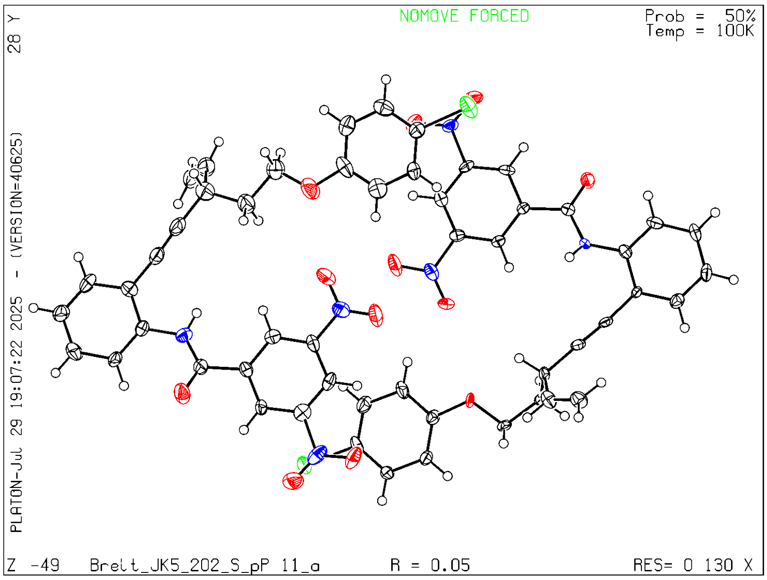

Crystals were obtained at room temperature by slow diffusion of pentane into asolution of the compound dissolved in diethylether by the aid of layering. A colorless, block-shaped crystal was mounted on a MiTeGen micromount with perfluoroether oil. Data for Breit_JK5_202_S_pure1_a were collected from a shock-cooled single crystal at 100(2) K on a Bruker APEX2 QUAZAR three-circle diffractometer with a microfocus sealed X-ray tube using a mirror optics as monochromator and a Bruker APEXII detector. The diffractometer was equipped with an Oxford Cryostream 800 low temperature device and used MoK_{α} radiation (λ = 0.71073 Å). All data were integrated with SAINT V8.41 and a multi-scan absorption correction using SADABS 2016/2 was applied (Bruker, *SAINT*, *V8.41*, Bruker AXS Inc., Madison, Wisconsin, USA).[30] The structure was solved by direct methods with SHELXT and refined by full-matrix least-squares methods against F^2 using SHELXL-2019/2.[31] All non-hydrogen atoms were refined with anisotropic displacement parameters. All hydrogen atoms were refined with isotropic displacement parameters. Some of their coordinates were refined freely and some on calculated positions using a riding model with their U_{iso} values constrained to 1.5 times the U_{eq} of their pivot atoms for terminal sp^3 carbon atoms and 1.2 times for all other carbon atoms. Crystallographic data for the structures reported in this paper have been deposited with the Cambridge Crystallographic Data Centre.[32] CCDC 2476964 contain the supplementary crystallographic data for this paper. These data can be obtained free of charge from The Cambridge Crystallographic Data Centre via www.ccdc.cam.ac.uk/structures. This report and the CIF file were generated using FinalCif (D. Kratzert, *FinalCif*, *V143*, https://dkratzert.de/finalcif.html).

Crystal data and structure refinement for Breit_JK5_202_S_pure1_a

CCDC number	2476964
Empirical formula	$C_{26}H_{20}BrN_3O_6$

Formula weight	550.36
Temperature [K]	100(2)
Crystal system	triclinic
Space group (number)	$P1$ (1)
a [Å]	9.299(5)
b [Å]	11.461(7)
c [Å]	12.110(6)
α [°]	104.98(3)
β [°]	100.080(13)
γ [°]	104.364(10)
Volume [$Å^3$]	1167.6(11)
Z	2
ρ_{calc} [gcm^{-3}]	1.565
μ [mm^{-1}]	1.810
F(000)	560
Crystal size [mm^3]	0.116×0.187×0.250
Crystal color	colorless
Crystal shape	block
Radiation	MoK_α (λ=0.71073 Å)
2θ range [°]	3.60 to 52.94 (0.80 Å)
Index ranges	−11 ≤ h ≤ 11<br>−14 ≤ k ≤ 14<br>−15 ≤ l ≤ 15
Reflections collected	23361
Independent reflections	9623<br>R_{int} = 0.0302<br>R_{sigma} = 0.0489
Completeness to θ = 25.242°	100.0 %
Data / Restraints / Parameters	9623 / 9 / 655
Absorption correction T_{min}/T_{max} (method)	0.6735 / 0.7454 (multi-scan)
Goodness-of-fit on F^2	1.034
Final R indexes [$I \geq 2\sigma(I)$]	R_1 = 0.0524<br>wR_2 = 0.1301
Final R indexes [all data]	R_1 = 0.0670<br>wR_2 = 0.1386
Largest peak/hole [$eÅ^{-3}$]	1.45/−0.57
Flack X parameter	0.019(5)

Atomic coordinates and U_{eq} [Å^2] for Breit_JK5_202_S_pure1_a

Atom	*x*	*y*	*z*	U_{eq}
Br1	1.28377(8)	0.53988(8)	0.69477(7)	0.0471(3)
Br2	−0.23995(7)	0.46095(6)	0.31721(6)	0.0317(2)
O1	0.6528(8)	0.3012(7)	0.7430(7)	0.0514(19)
O2	−0.2569(9)	−0.0081(8)	0.4346(6)	0.056(2)
O3	−0.0428(9)	0.2650(7)	0.0921(6)	0.0428(18)
O4	−0.2556(9)	0.1587(8)	0.1164(6)	0.0466(19)
O5	0.4294(8)	0.3771(7)	0.3931(6)	0.0457(19)
O6	0.4249(8)	0.3144(8)	0.5425(7)	0.051(2)
O7	0.3617(6)	0.6958(5)	0.2166(5)	0.0244(12)
O8	1.2934(8)	0.9814(8)	0.5634(5)	0.055(2)
O9	0.5943(7)	0.6784(7)	0.4497(6)	0.0337(16)
O10	0.5852(8)	0.6174(7)	0.6033(6)	0.0430(18)
O11	1.2646(7)	0.8364(7)	0.8933(5)	0.0409(17)
O12	1.0469(8)	0.7285(7)	0.9106(6)	0.0413(17)
N1	−0.0961(9)	0.0983(8)	0.6133(6)	0.0286(16)
H1N	−0.001(4)	0.132(7)	0.650(5)	0.034
N2	−0.1220(12)	0.2128(8)	0.1466(7)	0.038(2)
N3	0.3629(10)	0.3255(8)	0.4504(7)	0.0328(19)
N4	1.1106(7)	0.8949(7)	0.3892(6)	0.0161(14)
H4N	1.043(6)	0.823(4)	0.352(4)	0.019
N5	0.6530(9)	0.6688(7)	0.5398(7)	0.0299(18)
N6	1.1265(8)	0.7821(8)	0.8565(6)	0.0257(16)
C1	0.3439(12)	0.3677(10)	1.0794(9)	0.039(2)

H1A	0.237199	0.342105	1.042621	0.047
H1B	0.386294	0.435176	1.152006	0.047
C2	0.4328(11)	0.3100(9)	1.0298(8)	0.048(2)
H2	0.538888	0.338340	1.069174	0.058
C3	0.3789(11)	0.2018(9)	0.9146(8)	0.047(2)
H3	0.416944	0.131637	0.929833	0.056
C4	0.4458(10)	0.2373(9)	0.8185(8)	0.046(2)
H4A	0.412268	0.308648	0.803941	0.055
H4B	0.401977	0.164191	0.744886	0.055
C5	0.6221(11)	0.2758(9)	0.8436(9)	0.042(2)
H5A	0.670531	0.352045	0.914186	0.050
H5B	0.660302	0.205863	0.856584	0.050
C6	0.8015(11)	0.3562(9)	0.7416(10)	0.037(2)
C7	0.8189(12)	0.3879(10)	0.6449(10)	0.042(2)
H7	0.730194	0.372376	0.583913	0.050
C8	0.9595(11)	0.4418(9)	0.6310(9)	0.030(2)
H8	0.968271	0.465010	0.562058	0.036
C9	1.0883(11)	0.4623(10)	0.7171(8)	0.032(2)
C10	1.0734(12)	0.4300(10)	0.8166(9)	0.039(2)
H10	1.162546	0.444933	0.876900	0.047
C11	0.9302(12)	0.3760(9)	0.8303(8)	0.037(2)
H11	0.919866	0.352771	0.899000	0.045
C12	0.2064(12)	0.1507(9)	0.8756(8)	0.045(3)
C13	0.0697(11)	0.1070(9)	0.8373(8)	0.033(2)
C14	−0.0936(10)	0.0474(8)	0.7966(8)	0.029(2)

C15	−0.1769(12)	−0.0117(9)	0.8649(8)	0.035(2)
H15	−0.123681	−0.006598	0.941319	0.042
C16	−0.3280(10)	−0.0747(10)	0.8265(8)	0.036(2)
H16	−0.379053	−0.115436	0.874264	0.043
C17	−0.4080(11)	−0.0797(11)	0.7181(9)	0.041(2)
H17	−0.515583	−0.121142	0.692154	0.049
C18	−0.3321(10)	−0.0241(9)	0.6452(8)	0.029(2)
H18	−0.387609	−0.029521	0.569320	0.034
C19	−0.1767(10)	0.0383(8)	0.6842(7)	0.0228(18)
C20	−0.1418(10)	0.0732(10)	0.4967(8)	0.030(2)
C21	−0.0417(10)	0.1493(8)	0.4368(7)	0.0236(18)
C22	−0.1166(10)	0.1514(9)	0.3290(7)	0.0244(18)
H22	−0.223915	0.110420	0.298353	0.029
C23	−0.0328(11)	0.2143(9)	0.2654(8)	0.028(2)
C24	0.1225(11)	0.2725(8)	0.3024(7)	0.027(2)
H24	0.177637	0.312616	0.256255	0.033
C25	0.1950(10)	0.2691(8)	0.4123(8)	0.027(2)
C26	0.1180(11)	0.2108(8)	0.4797(8)	0.0253(19)
H26	0.171506	0.211654	0.554512	0.030
C27	0.6748(11)	0.6245(9)	−0.0787(8)	0.032(2)
H27A	0.776507	0.680154	−0.059590	0.038
H27B	0.633323	0.560301	−0.153479	0.038
C28	0.5907(9)	0.6364(7)	−0.0009(7)	0.0296(17)
H28	0.489879	0.578114	−0.024769	0.035
C29	0.6368(7)	0.7316(7)	0.1208(6)	0.0222(14)

H29	0.626331	0.683028	0.178275	0.027
C30	0.5318(7)	0.8174(6)	0.1361(6)	0.0182(13)
H30A	0.572678	0.881590	0.215623	0.022
H30B	0.537091	0.863414	0.077287	0.022
C31	0.3667(8)	0.7487(7)	0.1222(6)	0.0214(15)
H31A	0.305267	0.808272	0.126099	0.026
H31B	0.324321	0.680615	0.044945	0.026
C32	0.2200(10)	0.6422(9)	0.2331(7)	0.0235(18)
C33	0.0822(10)	0.6184(8)	0.1536(7)	0.0230(18)
H33	0.081122	0.638754	0.082336	0.028
C34	−0.0580(10)	0.5634(8)	0.1783(7)	0.0213(17)
H34	−0.153706	0.546597	0.124330	0.026
C35	−0.0520(10)	0.5351(8)	0.2826(8)	0.025(2)
C36	0.0877(11)	0.5612(10)	0.3636(8)	0.031(2)
H36	0.088781	0.543041	0.435877	0.038
C37	0.2255(10)	0.6137(9)	0.3387(8)	0.0286(19)
H37	0.321124	0.629758	0.392494	0.034
C38	0.8015(8)	0.8119(8)	0.1527(6)	0.0231(16)
C39	0.9332(9)	0.8733(8)	0.1734(6)	0.0192(16)
C40	1.0938(9)	0.9441(7)	0.2073(6)	0.0161(15)
C41	1.1655(9)	1.0015(8)	0.1344(7)	0.0246(18)
H41	1.105689	0.993656	0.059151	0.030
C42	1.3176(10)	1.0684(8)	0.1664(8)	0.0276(19)
H42	1.362300	1.107663	0.115143	0.033
C43	1.4072(10)	1.0782(8)	0.2766(8)	0.0258(19)

H43	1.513088	1.125851	0.301430	0.031
C44	1.3403(10)	1.0182(9)	0.3486(8)	0.029(2)
H44	1.402939	1.022151	0.421225	0.034
C45	1.1848(9)	0.9523(8)	0.3184(7)	0.0198(17)
C46	1.1658(10)	0.9110(9)	0.5054(7)	0.0248(18)
C47	1.0618(9)	0.8394(8)	0.5642(7)	0.0196(17)
C48	0.9051(9)	0.7838(8)	0.5204(7)	0.0214(18)
H48	0.854147	0.784662	0.445578	0.026
C49	0.8227(10)	0.7261(8)	0.5891(7)	0.0213(18)
C50	0.8907(10)	0.7222(8)	0.6955(7)	0.0242(18)
H50	0.832848	0.680367	0.739203	0.029
C51	1.0468(10)	0.7810(9)	0.7387(7)	0.0224(18)
C52	1.1336(9)	0.8393(8)	0.6769(7)	0.0211(17)
H52	1.240850	0.879361	0.709290	0.025

Anisotropic displacement parameters [Å2] for Breit_JK5_202_S_pure1_a. The anisotropic displacement factor exponent takes the form:
−2π2[h2(a*)2U11 + k2(b*)2U22 + … + 2hka*b*U12]

Atom	U_{11}	U_{22}	U_{33}	U_{23}	U_{13}	U_{12}
Br1	0.0318(5)	0.0452(7)	0.0537(7)	0.0080(5)	0.0152(5)	−0.0013(4)
Br2	0.0239(4)	0.0326(5)	0.0341(5)	0.0084(4)	0.0103(3)	0.0012(3)
O1	0.032(4)	0.067(5)	0.057(5)	0.020(4)	0.021(3)	0.013(3)
O2	0.045(4)	0.066(5)	0.027(3)	0.009(3)	0.003(3)	−0.022(4)
O3	0.066(5)	0.040(4)	0.026(3)	0.016(3)	0.019(3)	0.011(3)
O4	0.042(4)	0.061(5)	0.032(4)	0.013(3)	0.003(3)	0.013(4)
O5	0.039(4)	0.044(4)	0.047(4)	0.014(3)	0.020(3)	−0.005(3)

O6	0.024(3)	0.058(5)	0.064(5)	0.027(4)	−0.001(3)	0.000(3)
O7	0.020(3)	0.035(3)	0.027(3)	0.022(2)	0.014(2)	0.007(2)
O8	0.032(4)	0.088(6)	0.023(3)	0.027(3)	−0.005(3)	−0.021(4)
O9	0.020(3)	0.044(4)	0.035(3)	0.021(3)	−0.004(3)	0.006(3)
O10	0.033(4)	0.038(4)	0.042(4)	0.005(3)	0.015(3)	−0.012(3)
O11	0.030(3)	0.067(5)	0.023(3)	0.018(3)	0.000(3)	0.011(3)
O12	0.042(4)	0.059(5)	0.031(3)	0.027(3)	0.013(3)	0.014(3)
N1	0.031(4)	0.033(4)	0.022(3)	0.009(3)	0.006(3)	0.013(3)
N2	0.065(7)	0.031(4)	0.021(4)	0.009(3)	0.012(4)	0.020(4)
N3	0.029(4)	0.031(4)	0.032(4)	0.005(3)	0.003(3)	0.005(3)
N4	0.010(3)	0.019(3)	0.017(3)	0.007(3)	0.001(2)	0.001(2)
N5	0.023(4)	0.025(4)	0.036(4)	0.007(3)	0.007(3)	−0.001(3)
N6	0.026(4)	0.040(4)	0.014(3)	0.009(3)	0.005(3)	0.013(3)
C1	0.040(5)	0.043(6)	0.027(5)	0.011(4)	0.011(4)	0.001(4)
C2	0.046(5)	0.054(6)	0.029(4)	0.012(4)	0.004(4)	−0.006(5)
C3	0.040(5)	0.049(5)	0.043(5)	0.014(4)	0.005(4)	0.004(4)
C4	0.038(5)	0.054(6)	0.034(5)	0.010(4)	0.008(4)	−0.001(4)
C5	0.044(6)	0.037(5)	0.043(5)	0.012(4)	0.014(4)	0.011(4)
C6	0.026(4)	0.024(5)	0.056(6)	0.004(4)	0.016(4)	0.004(4)
C7	0.041(6)	0.037(5)	0.054(6)	0.017(4)	0.016(5)	0.017(4)
C8	0.030(5)	0.023(4)	0.037(5)	0.012(4)	0.011(4)	0.003(4)
C9	0.033(5)	0.037(6)	0.026(4)	0.011(4)	0.007(4)	0.007(4)
C10	0.032(5)	0.032(5)	0.043(5)	0.005(4)	0.001(4)	0.009(4)
C11	0.051(6)	0.031(5)	0.033(5)	0.011(4)	0.017(4)	0.012(4)
C12	0.052(6)	0.051(6)	0.023(4)	0.018(4)	0.003(4)	−0.003(5)

C13	0.042(6)	0.030(5)	0.023(4)	0.011(3)	0.007(4)	0.003(4)
C14	0.027(5)	0.027(4)	0.028(4)	0.003(3)	0.006(3)	0.007(3)
C15	0.052(6)	0.042(5)	0.022(4)	0.016(4)	0.018(4)	0.020(5)
C16	0.034(5)	0.054(6)	0.029(5)	0.019(4)	0.015(4)	0.019(4)
C17	0.022(4)	0.060(7)	0.045(5)	0.020(5)	0.015(4)	0.012(4)
C18	0.029(4)	0.043(5)	0.023(4)	0.018(4)	0.013(3)	0.015(4)
C19	0.030(5)	0.021(4)	0.019(4)	0.006(3)	0.010(3)	0.009(3)
C20	0.028(5)	0.037(5)	0.022(4)	0.010(4)	0.007(4)	0.004(4)
C21	0.022(4)	0.017(4)	0.026(4)	0.003(3)	0.006(3)	0.002(3)
C22	0.022(4)	0.029(4)	0.024(4)	0.011(3)	0.011(3)	0.005(3)
C23	0.036(5)	0.023(4)	0.024(4)	0.001(3)	0.011(4)	0.014(4)
C24	0.044(5)	0.018(4)	0.023(4)	0.009(3)	0.013(4)	0.008(4)
C25	0.021(4)	0.021(4)	0.041(5)	0.012(4)	0.013(4)	0.005(3)
C26	0.030(5)	0.019(4)	0.024(4)	0.005(3)	0.003(4)	0.008(4)
C27	0.029(5)	0.028(5)	0.030(5)	0.002(4)	0.005(4)	0.004(4)
C28	0.023(4)	0.025(4)	0.041(4)	0.009(3)	0.011(3)	0.007(3)
C29	0.017(3)	0.030(4)	0.028(4)	0.017(3)	0.012(3)	0.008(3)
C30	0.020(3)	0.023(3)	0.017(3)	0.011(3)	0.006(3)	0.010(3)
C31	0.021(4)	0.028(4)	0.022(3)	0.015(3)	0.004(3)	0.013(3)
C32	0.026(4)	0.030(4)	0.023(4)	0.013(3)	0.013(3)	0.015(4)
C33	0.026(4)	0.026(4)	0.021(4)	0.012(3)	0.009(3)	0.009(3)
C34	0.018(4)	0.026(4)	0.023(4)	0.009(3)	0.007(3)	0.009(3)
C35	0.019(4)	0.014(4)	0.042(5)	0.004(4)	0.017(4)	0.004(3)
C36	0.037(5)	0.038(5)	0.027(4)	0.016(4)	0.015(4)	0.015(4)
C37	0.020(4)	0.030(5)	0.041(5)	0.015(4)	0.016(4)	0.008(3)

C38	0.020(4)	0.037(4)	0.016(3)	0.012(3)	0.004(3)	0.014(3)
C39	0.021(4)	0.027(4)	0.011(3)	0.008(3)	0.002(3)	0.010(3)
C40	0.016(4)	0.021(4)	0.017(3)	0.012(3)	0.007(3)	0.009(3)
C41	0.028(4)	0.031(4)	0.021(4)	0.011(3)	0.011(3)	0.013(3)
C42	0.028(4)	0.025(4)	0.041(5)	0.021(4)	0.019(4)	0.010(4)
C43	0.019(4)	0.022(4)	0.040(5)	0.012(3)	0.015(3)	0.004(3)
C44	0.019(4)	0.031(5)	0.029(4)	0.007(4)	−0.003(3)	0.005(3)
C45	0.016(4)	0.021(4)	0.024(4)	0.005(3)	0.008(3)	0.007(3)
C46	0.019(4)	0.029(4)	0.024(4)	0.011(3)	0.001(3)	0.002(3)
C47	0.019(4)	0.025(4)	0.014(3)	0.008(3)	0.003(3)	0.005(3)
C48	0.021(4)	0.027(4)	0.016(4)	0.009(3)	0.002(3)	0.007(3)
C49	0.024(4)	0.021(4)	0.016(4)	0.002(3)	0.001(3)	0.007(3)
C50	0.022(4)	0.022(4)	0.027(4)	0.006(3)	0.009(3)	0.005(3)
C51	0.029(4)	0.029(5)	0.012(3)	0.009(3)	0.004(3)	0.012(4)
C52	0.015(4)	0.032(5)	0.015(4)	0.002(3)	0.000(3)	0.011(3)

Bond lengths and angles for Breit_JK5_202_S_pure1_a

**Atom–Atom**	**Length [Å]**
Br1–C9	1.914(10)
Br2–C35	1.912(8)
O1–C6	1.375(12)
O1–C5	1.383(11)
O2–C20	1.200(11)
O3–N2	1.221(11)
O4–N2	1.185(11)

O5–N3	1.181(10)
O6–N3	1.216(11)
O7–C32	1.381(11)
O7–C31	1.428(9)
O8–C46	1.222(10)
O9–N5	1.176(10)
O10–N5	1.247(10)
O11–N6	1.223(10)
O12–N6	1.228(10)
N1–C20	1.334(11)
N1–C19	1.430(11)
N1–H1N	0.86(3)
N2–C23	1.523(12)
N3–C25	1.470(12)
N4–C46	1.358(10)
N4–C45	1.384(11)
N4–H4N	0.85(3)
N5–C49	1.494(11)
N6–C51	1.483(10)
C1–C2	1.311(14)
C1–H1A	0.9500
C1–H1B	0.9500
C2–C3	1.513(13)
C2–H2	0.9500
C3–C12	1.506(13)

C3–C4	1.509(13)
C3–H3	1.0000
C4–C5	1.540(13)
C4–H4A	0.9900
C4–H4B	0.9900
C5–H5A	0.9900
C5–H5B	0.9900
C6–C7	1.337(15)
C6–C11	1.388(14)
C7–C8	1.360(15)
C7–H7	0.9500
C8–C9	1.369(13)
C8–H8	0.9500
C9–C10	1.368(15)
C10–C11	1.381(15)
C10–H10	0.9500
C11–H11	0.9500
C12–C13	1.199(13)
C13–C14	1.437(12)
C14–C19	1.406(12)
C14–C15	1.412(13)
C15–C16	1.345(13)
C15–H15	0.9500
C16–C17	1.370(14)
C16–H16	0.9500

C17–C18	1.404(13)
C17–H17	0.9500
C18–C19	1.377(13)
C18–H18	0.9500
C20–C21	1.520(12)
C21–C22	1.378(12)
C21–C26	1.412(13)
C22–C23	1.392(12)
C22–H22	0.9500
C23–C24	1.369(13)
C24–C25	1.398(13)
C24–H24	0.9500
C25–C26	1.369(13)
C26–H26	0.9500
C27–C28	1.328(12)
C27–H27A	0.9500
C27–H27B	0.9500
C28–C29	1.504(10)
C28–H28	0.9500
C29–C38	1.500(10)
C29–C30	1.549(9)
C29–H29	1.0000
C30–C31	1.499(10)
C30–H30A	0.9900
C30–H30B	0.9900

C31–H31A	0.9900
C31–H31B	0.9900
C32–C33	1.379(12)
C32–C37	1.396(12)
C33–C34	1.419(12)
C33–H33	0.9500
C34–C35	1.378(12)
C34–H34	0.9500
C35–C36	1.396(13)
C36–C37	1.393(13)
C36–H36	0.9500
C37–H37	0.9500
C38–C39	1.196(11)
C39–C40	1.439(11)
C40–C41	1.395(11)
C40–C45	1.425(11)
C41–C42	1.362(11)
C41–H41	0.9500
C42–C43	1.405(12)
C42–H42	0.9500
C43–C44	1.381(14)
C43–H43	0.9500
C44–C45	1.388(11)
C44–H44	0.9500
C46–C47	1.505(12)

C47–C48	1.380(11)
C47–C52	1.410(10)
C48–C49	1.404(12)
C48–H48	0.9500
C49–C50	1.349(12)
C50–C51	1.379(12)
C50–H50	0.9500
C51–C52	1.364(12)
C52–H52	0.9500

**Atom–Atom–Atom**	**Angle [°]**
C6–O1–C5	120.0(8)
C32–O7–C31	118.3(6)
C20–N1–C19	126.4(8)
C20–N1–H1N	120(4)
C19–N1–H1N	110(4)
O4–N2–O3	128.1(9)
O4–N2–C23	117.6(8)
O3–N2–C23	114.3(9)
O5–N3–O6	124.0(9)
O5–N3–C25	120.6(8)
O6–N3–C25	115.4(8)
C46–N4–C45	127.3(7)
C46–N4–H4N	113(4)
C45–N4–H4N	114(4)

O9–N5–O10	125.7(8)
O9–N5–C49	119.6(8)
O10–N5–C49	114.6(7)
O11–N6–O12	124.7(7)
O11–N6–C51	118.6(7)
O12–N6–C51	116.6(7)
C2–C1–H1A	120.0
C2–C1–H1B	120.0
H1A–C1–H1B	120.0
C1–C2–C3	124.9(9)
C1–C2–H2	117.6
C3–C2–H2	117.6
C12–C3–C4	111.6(8)
C12–C3–C2	111.3(8)
C4–C3–C2	112.5(8)
C12–C3–H3	107.1
C4–C3–H3	107.1
C2–C3–H3	107.1
C3–C4–C5	115.9(8)
C3–C4–H4A	108.3
C5–C4–H4A	108.3
C3–C4–H4B	108.3
C5–C4–H4B	108.3
H4A–C4–H4B	107.4
O1–C5–C4	104.3(8)

O1–C5–H5A	110.9
C4–C5–H5A	110.9
O1–C5–H5B	110.9
C4–C5–H5B	110.9
H5A–C5–H5B	108.9
C7–C6–O1	115.8(10)
C7–C6–C11	119.6(9)
O1–C6–C11	124.6(10)
C6–C7–C8	122.1(11)
C6–C7–H7	119.0
C8–C7–H7	119.0
C7–C8–C9	119.5(10)
C7–C8–H8	120.2
C9–C8–H8	120.2
C10–C9–C8	119.5(9)
C10–C9–Br1	122.4(7)
C8–C9–Br1	118.1(7)
C9–C10–C11	120.6(9)
C9–C10–H10	119.7
C11–C10–H10	119.7
C10–C11–C6	118.7(9)
C10–C11–H11	120.7
C6–C11–H11	120.7
C13–C12–C3	175.8(10)
C12–C13–C14	175.3(10)

C19–C14–C15	116.8(8)
C19–C14–C13	121.7(8)
C15–C14–C13	121.4(8)
C16–C15–C14	122.9(9)
C16–C15–H15	118.5
C14–C15–H15	118.5
C15–C16–C17	119.5(10)
C15–C16–H16	120.2
C17–C16–H16	120.2
C16–C17–C18	120.5(9)
C16–C17–H17	119.7
C18–C17–H17	119.7
C19–C18–C17	119.6(8)
C19–C18–H18	120.2
C17–C18–H18	120.2
C18–C19–C14	120.6(8)
C18–C19–N1	121.1(8)
C14–C19–N1	118.2(8)
O2–C20–N1	123.9(9)
O2–C20–C21	117.3(8)
N1–C20–C21	118.8(8)
C22–C21–C26	119.1(8)
C22–C21–C20	115.1(7)
C26–C21–C20	125.8(8)
C21–C22–C23	119.1(8)

C21–C22–H22	120.4
C23–C22–H22	120.4
C24–C23–C22	123.7(8)
C24–C23–N2	119.7(8)
C22–C23–N2	116.6(8)
C23–C24–C25	115.8(8)
C23–C24–H24	122.1
C25–C24–H24	122.1
C26–C25–C24	123.1(9)
C26–C25–N3	120.5(8)
C24–C25–N3	116.3(8)
C25–C26–C21	119.2(8)
C25–C26–H26	120.4
C21–C26–H26	120.4
C28–C27–H27A	120.0
C28–C27–H27B	120.0
H27A–C27–H27B	120.0
C27–C28–C29	127.4(7)
C27–C28–H28	116.3
C29–C28–H28	116.3
C38–C29–C28	111.7(6)
C38–C29–C30	110.0(6)
C28–C29–C30	112.6(6)
C38–C29–H29	107.4
C28–C29–H29	107.4

C30–C29–H29	107.4
C31–C30–C29	114.9(5)
C31–C30–H30A	108.5
C29–C30–H30A	108.5
C31–C30–H30B	108.5
C29–C30–H30B	108.5
H30A–C30–H30B	107.5
O7–C31–C30	107.1(6)
O7–C31–H31A	110.3
C30–C31–H31A	110.3
O7–C31–H31B	110.3
C30–C31–H31B	110.3
H31A–C31–H31B	108.6
C33–C32–O7	124.0(7)
C33–C32–C37	121.4(8)
O7–C32–C37	114.6(7)
C32–C33–C34	120.0(8)
C32–C33–H33	120.0
C34–C33–H33	120.0
C35–C34–C33	118.4(8)
C35–C34–H34	120.8
C33–C34–H34	120.8
C34–C35–C36	121.4(8)
C34–C35–Br2	118.9(7)
C36–C35–Br2	119.7(7)

C37–C36–C35	120.3(8)
C37–C36–H36	119.9
C35–C36–H36	119.9
C36–C37–C32	118.5(8)
C36–C37–H37	120.7
C32–C37–H37	120.7
C39–C38–C29	177.2(8)
C38–C39–C40	175.1(8)
C41–C40–C45	118.6(7)
C41–C40–C39	122.8(7)
C45–C40–C39	118.6(7)
C42–C41–C40	122.8(8)
C42–C41–H41	118.6
C40–C41–H41	118.6
C41–C42–C43	118.8(8)
C41–C42–H42	120.6
C43–C42–H42	120.6
C44–C43–C42	119.5(8)
C44–C43–H43	120.2
C42–C43–H43	120.2
C43–C44–C45	122.3(8)
C43–C44–H44	118.9
C45–C44–H44	118.9
N4–C45–C44	124.7(7)
N4–C45–C40	117.3(7)

C44–C45–C40	117.9(8)
O8–C46–N4	123.1(8)
O8–C46–C47	119.4(7)
N4–C46–C47	117.5(7)
C48–C47–C52	119.4(8)
C48–C47–C46	125.3(7)
C52–C47–C46	115.1(7)
C47–C48–C49	118.5(7)
C47–C48–H48	120.8
C49–C48–H48	120.8
C50–C49–C48	122.7(8)
C50–C49–N5	120.3(8)
C48–C49–N5	117.0(7)
C49–C50–C51	117.8(8)
C49–C50–H50	121.1
C51–C50–H50	121.1
C52–C51–C50	122.5(7)
C52–C51–N6	117.5(7)
C50–C51–N6	120.0(8)
C51–C52–C47	119.1(7)
C51–C52–H52	120.5
C47–C52–H52	120.5

**Atom–Atom–Atom–Atom**	**Torsion Angle [°]**
C1–C2–C3–C12	−11.3(14)

C1–C2–C3–C4	114.7(11)
C12–C3–C4–C5	−173.1(8)
C2–C3–C4–C5	61.0(12)
C6–O1–C5–C4	170.7(8)
C3–C4–C5–O1	179.1(8)
C5–O1–C6–C7	−174.1(9)
C5–O1–C6–C11	8.1(15)
O1–C6–C7–C8	−179.3(9)
C11–C6–C7–C8	−1.3(16)
C6–C7–C8–C9	1.3(16)
C7–C8–C9–C10	−1.0(16)
C7–C8–C9–Br1	−179.4(8)
C8–C9–C10–C11	0.7(16)
Br1–C9–C10–C11	179.0(8)
C9–C10–C11–C6	−0.6(16)
C7–C6–C11–C10	0.9(15)
O1–C6–C11–C10	178.7(9)
C19–C14–C15–C16	0.3(15)
C13–C14–C15–C16	−176.0(9)
C14–C15–C16–C17	−2.1(16)
C15–C16–C17–C18	2.6(16)
C16–C17–C18–C19	−1.4(16)
C17–C18–C19–C14	−0.3(15)
C17–C18–C19–N1	−178.7(9)
C15–C14–C19–C18	0.8(13)

C13–C14–C19–C18	177.2(9)
C15–C14–C19–N1	179.3(8)
C13–C14–C19–N1	−4.4(13)
C20–N1–C19–C18	−20.7(14)
C20–N1–C19–C14	160.9(9)
C19–N1–C20–O2	−2.6(17)
C19–N1–C20–C21	178.7(8)
O2–C20–C21–C22	23.0(14)
N1–C20–C21–C22	−158.2(9)
O2–C20–C21–C26	−154.0(10)
N1–C20–C21–C26	24.8(14)
C26–C21–C22–C23	−0.2(13)
C20–C21–C22–C23	−177.3(8)
C21–C22–C23–C24	1.8(14)
C21–C22–C23–N2	179.8(8)
O4–N2–C23–C24	178.1(9)
O3–N2–C23–C24	0.8(13)
O4–N2–C23–C22	0.1(13)
O3–N2–C23–C22	−177.3(8)
C22–C23–C24–C25	−2.0(14)
N2–C23–C24–C25	−179.9(8)
C23–C24–C25–C26	0.6(14)
C23–C24–C25–N3	177.0(8)
O5–N3–C25–C26	−179.1(9)
O6–N3–C25–C26	1.1(14)

O5–N3–C25–C24	4.4(14)
O6–N3–C25–C24	−175.5(9)
C24–C25–C26–C21	0.9(14)
N3–C25–C26–C21	−175.4(8)
C22–C21–C26–C25	−1.1(14)
C20–C21–C26–C25	175.7(9)
C27–C28–C29–C38	−4.4(11)
C27–C28–C29–C30	120.0(9)
C38–C29–C30–C31	−172.3(6)
C28–C29–C30–C31	62.3(8)
C32–O7–C31–C30	171.3(6)
C29–C30–C31–O7	64.9(7)
C31–O7–C32–C33	10.6(12)
C31–O7–C32–C37	−169.0(7)
O7–C32–C33–C34	−179.7(8)
C37–C32–C33–C34	−0.2(14)
C32–C33–C34–C35	0.0(13)
C33–C34–C35–C36	1.0(13)
C33–C34–C35–Br2	−180.0(6)
C34–C35–C36–C37	−1.8(14)
Br2–C35–C36–C37	179.2(7)
C35–C36–C37–C32	1.6(14)
C33–C32–C37–C36	−0.6(14)
O7–C32–C37–C36	179.0(8)
C45–C40–C41–C42	−2.1(13)

C39–C40–C41–C42	−179.9(8)
C40–C41–C42–C43	1.2(13)
C41–C42–C43–C44	1.3(13)
C42–C43–C44–C45	−2.9(15)
C46–N4–C45–C44	13.0(15)
C46–N4–C45–C40	−166.2(9)
C43–C44–C45–N4	−177.2(8)
C43–C44–C45–C40	2.0(15)
C41–C40–C45–N4	179.7(8)
C39–C40–C45–N4	−2.4(12)
C41–C40–C45–C44	0.5(13)
C39–C40–C45–C44	178.4(8)
C45–N4–C46–O8	0.0(16)
C45–N4–C46–C47	179.0(8)
O8–C46–C47–C48	162.4(10)
N4–C46–C47–C48	−16.6(14)
O8–C46–C47–C52	−12.9(13)
N4–C46–C47–C52	168.1(8)
C52–C47–C48–C49	−1.5(13)
C46–C47–C48–C49	−176.6(8)
C47–C48–C49–C50	−0.4(13)
C47–C48–C49–N5	179.0(8)
O9–N5–C49–C50	176.2(9)
O10–N5–C49–C50	−1.4(12)
O9–N5–C49–C48	−3.2(13)

O10–N5–C49–C48	179.2(8)
C48–C49–C50–C51	1.9(13)
N5–C49–C50–C51	−177.5(8)
C49–C50–C51–C52	−1.5(14)
C49–C50–C51–N6	178.5(8)
O11–N6–C51–C52	1.1(12)
O12–N6–C51–C52	−179.6(9)
O11–N6–C51–C50	−179.0(9)
O12–N6–C51–C50	0.3(13)
C50–C51–C52–C47	−0.3(13)
N6–C51–C52–C47	179.6(8)
C48–C47–C52–C51	1.9(13)
C46–C47–C52–C51	177.4(8)
